# High-Affinity Lectin Ligands Enable the Detection of Pathogenic *Pseudomonas aeruginosa* Biofilms: Implications for Diagnostics and Therapy

**DOI:** 10.1021/jacsau.4c00670

**Published:** 2024-12-03

**Authors:** Eva Zahorska, Lisa Marie Denig, Stefan Lienenklaus, Sakonwan Kuhaudomlarp, Thomas Tschernig, Peter Lipp, Antje Munder, Emilie Gillon, Saverio Minervini, Varvara Verkhova, Anne Imberty, Stefanie Wagner, Alexander Titz

**Affiliations:** † Chemical Biology of Carbohydrates (CBCH), Helmholtz Institute for Pharmaceutical Research Saarland (HIPS), Helmholtz Centre for Infection Research, Saarbrücken D-66123, Germany; ‡ Deutsches Zentrum für Infektionsforschung (DZIF), Standort Hannover-Braunschweig, Braunschweig 38124, Germany; § Department of Chemistry, Saarland University, Saarbrücken D-66123, Germany; ∥ PharmaScienceHub, Saarland University, Saarbrücken D-66123, Germany; ⊥ Hannover Medical School, Institute of Laboratory Animal Science, Hannover 30625, Germany; # Université Grenoble Alpes, CNRS, CERMAV, Grenoble 38000, France; ∇ Department of Biochemistry, Faculty of Science, Mahidol University, Bangkok 10400, Thailand; ○ Center for Excellence in Protein and Enzyme Technology, Faculty of Science, Mahidol University, Bangkok 10400, Thailand; ◆ Medical Faculty of Saarland University, Institute of Anatomy and Cell Biology, Homburg/Saar, D-66421, Germany; ¶ Center for Molecular Signaling (PZMS), Medical Faculty of Saarland University, Homburg/Saar D-66421, Germany; ◮ Department of Pediatric Pneumology, Allergology and Neonatology, Hannover Medical School, Carl Neuberg-Str. 1, Hannover D-30625, Germany; ◭ Biomedical Research in Endstage and Obstructive Lung Disease Hannover (BREATH), Member of the German Center for Lung Research (DZL), Hannover D-30625, Germany

**Keywords:** *Pseudomonas aeruginosa*, biofilm, lectins, imaging agents, glycosylation, glycomimetics

## Abstract

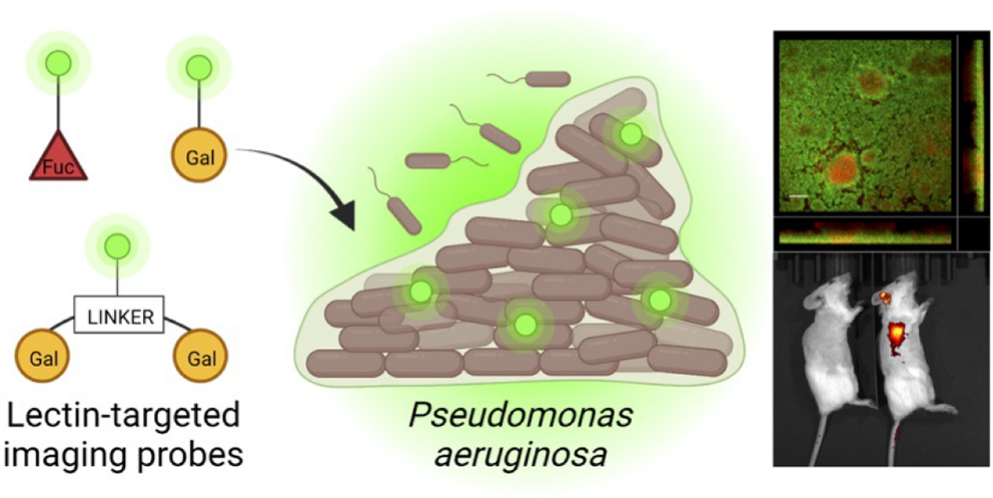

*Pseudomonas aeruginosa* is a critical priority pathogen and causes life-threatening acute and biofilm-associated chronic infections. The choice of suitable treatment for complicated infections requires lengthy culturing for species identification from swabs or an invasive biopsy. To date, no fast, pathogen-specific diagnostic tools for *P. aeruginosa* infections are available. Here, we present the noninvasive pathogen-specific detection of *P. aeruginosa* using novel fluorescent probes that target the bacterial biofilm-associated lectins LecA and LecB. Several glycomimetic probes were developed to target these extracellular lectins and demonstrated to stain *P. aeruginosa* biofilms *in vitro*. Importantly, for the targeting of LecA an activity boost to low-nanomolar affinity could be achieved, which is essential for *in vivo* application. *In vitro*, the nanomolar divalent LecA-targeted imaging probe accumulated effectively in biofilms under flow conditions, independent of the fluorophore identity. Investigation of these glycomimetic imaging probes in a murine lung infection model and fluorescence imaging revealed accumulation at the infection site. These findings demonstrate the use of LecA- and LecB-targeting probes for the imaging of *P. aeruginosa* infections and suggest their potential as pathogen-specific diagnostics to accelerate the start of the appropriate treatment.

## Introduction

Until the 20th century, people often died from simple bacterial infections. In 1928, the discovery of penicillin changed the world and antibiotics became widely used.^1^ However, since 1962, only two new classes of antibiotics have been developed, and bacterial resistance to commonly used antibiotic agents is steadily increasing.^2^ In Germany, 400,000 to 600,000 hospital-acquired infections occur annually, resulting in 10,000 to 15,000 fatalities.^3^ A recent report showed that in 2019, 4.95 million deaths were associated with bacterial antimicrobial resistance (AMR), including 1.27 million deaths directly attributable to AMR.^4^ It is estimated that worldwide, more than 10 million deaths per year will be caused by antimicrobial resistant pathogens by 2050.^5^

The majority of nosocomial infections are caused by six highly virulent and antibiotic-resistant bacteria, known as the ESKAPE pathogens, among which *Pseudomonas aeruginosa* has been recently classified as a priority 1 pathogen.^6^*P. aeruginosa* is able to colonize various tissues and organs, with infections most commonly affecting the respiratory and urinary tracts, wounds, and implanted medical devices.^7^*P. aeruginosa* has developed numerous resistance mechanisms against a wide range of antibiotic agents, leading to multi-drug resistance (MDR), extensive drug resistance (XDR), and total drug resistance (TDR).^8,9^ Furthermore, *P. aeruginosa* forms biofilms, which further increase antimicrobial resistance by 10- to 1000-fold, allowing the bacteria to evade the host immune system.^10−12^ Therefore, biofilm formation often results in untreatable infections and is a hallmark of chronic infections.^12^ The biofilm matrix of *P. aeruginosa* is a complex hydrogel composed of exopolysaccharides, extracellular DNA (eDNA), rhamnolipids, and proteins.^13^

Various adhesive proteins such as the flagellar cap protein FliD, type IV pili, and lectins are important for surface attachment, host recognition, and biofilm formation.^14−16^ The two soluble lectins, LecA and LecB, are present in the cytoplasm as well as on the bacterial outer membrane and play essential roles in the initial attachment to the host, biofilm formation, and stabilization of the biofilm matrix.^17−20^ LecA specifically binds to d-galactosides, while LecB recognizes d-mannosides and l-fucosides.^21,22^ The expression of both lectins is regulated by quorum-sensing and both are also considered to be virulence factors.^23−25^ Therefore, both lectins have emerged as targets for the treatment of *P. aeruginosa* infections.^26−29^

In many, and especially for complicated infections, a prerequisite for successful therapy is the detection and identification of the bacterial pathogen.^30^ This usually involves sampling from the infected tissue, sample preparation, microscopy, and bacterial cultivation on selective growth media for in-depth analysis, including biochemical and serological assays, as well as PCR and sequencing.^12^ This time-consuming conventional diagnostics is responsible for a significant delay of up to 48 h prior to start of therapy.^31^

The diagnosis of etiological agents in chronic infections is more problematic. Accessible infections are sampled with swabs, which only collect bacteria located at the surface of the tissue, possibly leading to a wrong diagnosis,^32^ while pathogens deeply embedded e.g., in a wound may remain undetected.^12^ Tissue biopsies are required for less accessible infection sites in organs or deep wounds. Nevertheless, successful isolation of the causative pathogen is not guaranteed given the heterogeneous distribution of bacteria, e.g., in chronic wounds.^12,33^ Moreover, a biopsy is an invasive procedure with additional stress and risks for the patient.

Noninvasive imaging techniques such as radiography, computed tomography (CT), or magnetic resonance imaging (MRI) can be additionally consulted for infection diagnostics.^34^ Single-photon emission computed tomography (SPECT) or positron emission tomography (PET) combined with the application of radiopharmaceuticals provide insights into metabolic pathways within the human body.^35^ However, these diagnostic methods detect morphological tissue changes or physiological alterations due to inflammation rather than the infection itself.^34^ Therefore, the ability to differentiate between sterile inflammation and infection using imaging techniques is still limited, and none of the current methods are pathogen-specific.^36^

In summary, there is an urgent need to develop new diagnostic tools for bacterial infections, especially for chronic infections of the most problematic pathogens. To date, a few compounds broadly detecting bacterial infections without pathogen specificity are under investigation. First, Locke et al. screened several antimicrobial peptide-based imaging probes for their ability to visualize *P. aeruginosain vitro,*(37) and one probe was effective to detect bacteria in wound infections *in vivo* after intravenous administration. The exact molecular mechanism of action and pathogen spectrum remains elusive. Second, a Trojan horse strategy exploits siderophores, i.e., chelators released by bacteria for iron uptake from the environment.^38^ Conjugation of siderophores with imaging moieties (e.g., fluorophores, PET tracers) was effective to stain ESKAPE bacteria *in vitro* and allowed the imaging of subcutaneously injected bacteria in mice.^39,40^ However, imaging success was dependent on the bacterial iron demand and competition with endogenous siderophores.^40^ In a third approach, the targeting of *P. aeruginosa* biofilms *in vitro* was demonstrated with a polymannosylated 26 kDa polymer labeled with rhodamine. Such polymeric carriers of native carbohydrates are prone to unspecific binding to host lectins of the immune system, e.g., mannose-binding lectin, mannose receptor, DC-SIGN, and others.^41^ Nevertheless, the retention of the mannose polymer at the biofilm *in vitro* was attributed to the two biofilm-resident, mannose-binding proteins, CdrA and LecB.^42^

We have previously reported the first staining of *P. aeruginosa* biofilms *in vitro* using a covalent LecA inhibitor coupled to fluorescein.^43^ This inhibitor carries a reactive epoxygalactoheptoside warhead to target a cysteine residue present in the LecA carbohydrate binding site with only moderate binding affinity in the micromolar range. However, the intrinsic reactivity toward nucleophiles coupled with its moderate affinity to the target lectin precludes its suitability for *in vivo* analysis. We also reported antibiotic-carbohydrate conjugates and glycomimetic-decorated liposomes for targeted drug delivery.^44−46^

In this study, we present the development of novel high-affinity LecA- and LecB-targeted imaging probes, their biophysical evaluation for lectin binding, and their application for *in vitro* staining of *P. aeruginosa* biofilms under static or flow conditions. Two lead compounds with high affinity to LecA or LecB were finally evaluated in a murine lung infection model and demonstrated to accumulate at the infection site after intravenous administration.

## Results and Discussion

### Monovalent LecA- and LecB-Targeting Imaging Probes

**Figure 1:**
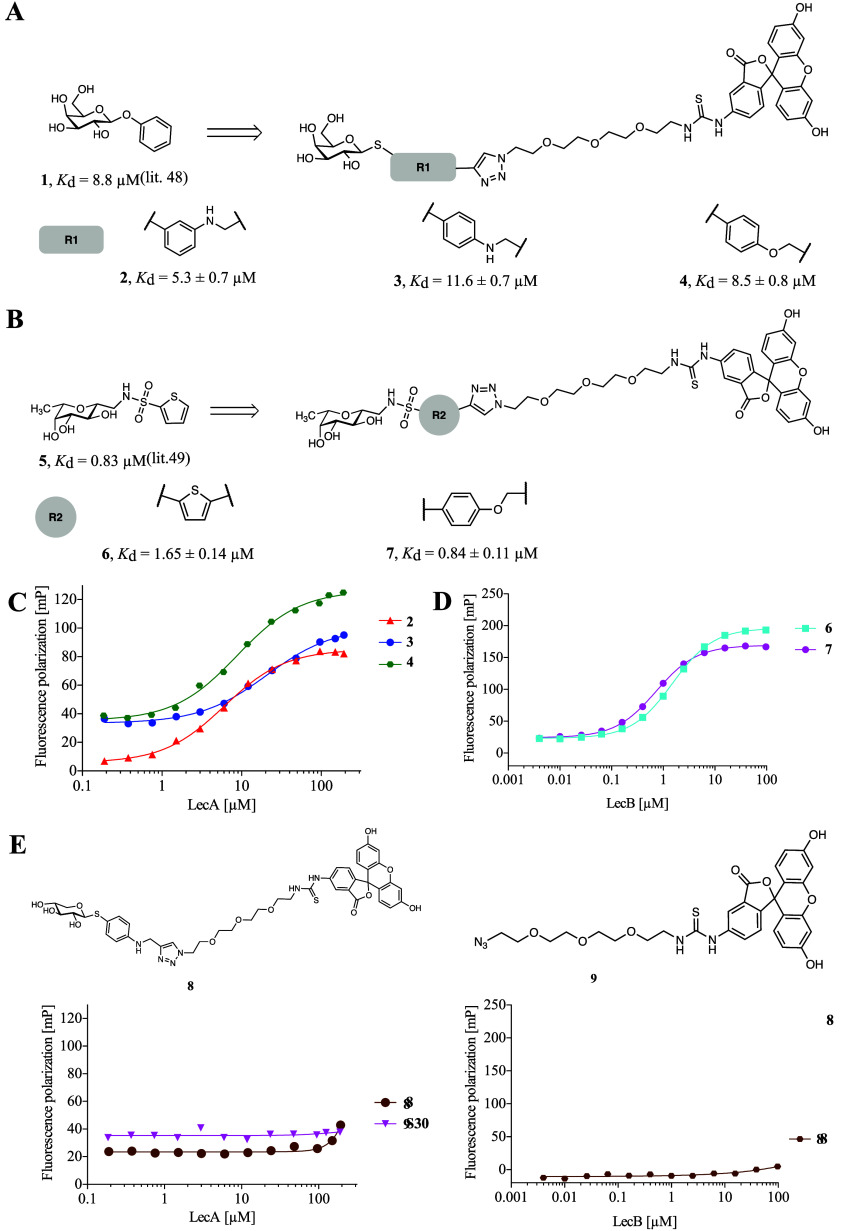
LecA- and LecB-targeted imaging probes and controls binding to the respective lectin determined by fluorescence polarization. (A, C) Monovalent LecA imaging probes **2**–**4** based on phenyl β-d-galactoside (**1**) were titrated with LecA. (B, D) LecB imaging probes **6** and **7** derived from LecB ligand **5** were titrated with LecB. (E) Xyloside-fluorescein conjugate (**8**) and azido fluorescein (**9**) served as negative controls in titrations with LecA and LecB, respectively. Averages and standard deviations were determined from at least three independent titrations of technical triplicates each. One representative titration is shown for each condition.

Due to a possible unspecific reactivity and resulting toxicity of the epoxide-based probe reported by Wagner et al.,^43^ we decided to target LecA with ligands derived from phenyl β-d-galactoside (**1**, Figure 1A). To this end, the phenyl aglycon was equipped with an alkyne handle and conjugated to azide-modified fluorescein in a copper-catalyzed Huisgen-type [3 + 2] cycloaddition to obtain a set of monovalent LecA-targeting imaging probes **2**–**4** (Figure 1A, synthesis described in the Supporting Information, see Schemes S1 and S4). The glycosidase-susceptible O-glycosidic linkage was replaced with a thioglycoside to improve metabolic stability. A small SAR study around the clickable handle was carried out by varying the position and chemical nature of the linker, specifically ether or amine functional groups. The binding affinities of imaging probes **2**–**4** for LecA were determined using a direct binding assay based on fluorescence polarization (FP).^47^ Substitution of the phenyl aglycon with an amine at its *meta* position was found to be the most favorable modification (compound **2**, *K*_d_ = 5.3 ± 0.7 μM). Amine substitution at the *para* position resulted in imaging probe **3** (*K*_d_ = 11.6 ± 0.7 μM), whose affinity could be moderately increased by exchange with an ether linkage in **4** (*K*_d_ = 8.5 ± 0.8 μM). Thus, the modification of the phenyl β-d-galactoside aglycon (**1**, *K*_d_ = 8.8 μM by ITC)^48^ with a fluorescent label was well tolerated by LecA in all cases.

The design of LecB-targeted probes involved conjugating a fluorophore to the optimized C-glycosidic LecB inhibitor **5** (Figure 1B),^49^ which exhibits high binding affinity (LecB_PAO1_*K*_d_ = 0.83 μM by ITC), long receptor residence time, excellent *in vivo* pharmacokinetic properties, and the ability to block *P. aeruginosa* biofilm formation *in vitro*. Similar to the LecA-targeted probes, LecB inhibitor **5** was modified with a terminal alkyne handle for the conjugation to fluorophores resulting in LecB-targeting probe **6** (synthesis described in the Supporting Information, see Schemes S2 and S4). Substitution of the thiophene with a benzene^50^ and incorporation of a short flexible connection to the triazole unit was explored with compound **7**. Both **6** and **7** showed good binding affinity for LecB with benzene **7** twice as active (*K*_d_ = 0.84 ± 0.11 μM) compared to thiophene **6** (*K*_d_ = 1.65 ± 0.14 μM).

Since LecA is specific for d-galactosides^47,48,51,52^ and LecB for l-fucosides/d-mannosides,^21,50,53^d-xyloside was selected as a negative control. To the best of our knowledge, xylose is not known to play any role in *P. aeruginosa* infection process and was unable to prevent *P. aeruginosa* adherence to kidney or lung cells.^54^ Furthermore, xylose upregulated *P. aeruginosa* virulence genes expression to a lesser extent than arabinose when supplemented in the growth media and also indicated to be metabolized at a slower rate than glucose or fructose.^55^ Therefore, xylosyl fluorescein conjugate **8** mimicking the design of LecA-targeted **3** was synthesized (synthesis described in the Supporting Information, see Schemes S3 and S4) and its inability to bind to LecA and LecB was confirmed (Figure 1E). Furthermore, azido-modified fluorescein **9** lacking a carbohydrate targeting moiety was included, and binding to LecA was also not observed. All monovalent glycosides were also tested in competitive binding assays with LecA and LecB prior to coupling to the fluorophores (Figure S2).

### *In Vitro* Biofilm Imaging under Static Conditions

**Figure 2:**
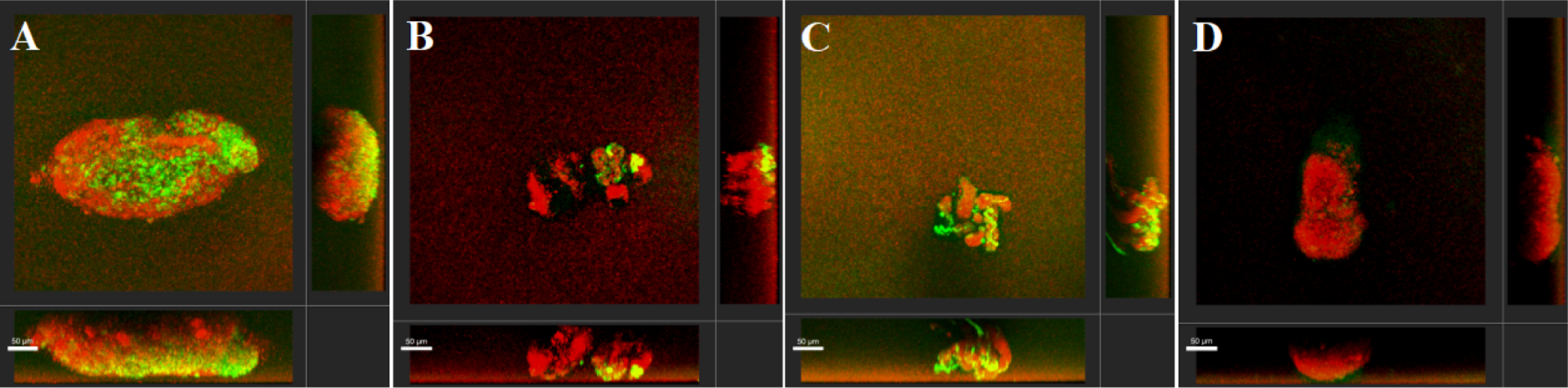
*P. aeruginosa* PAO1 biofilm aggregates stained with imaging probes at 10 μM displayed as three-dimensional maximum intensity projection using Imaris. Staining was observed for imaging probes galactoside **2** targeting LecA (A), C-fucoside **6** targeting LecB (B), and xyloside **8** (C). Azide **9** (D) did not stain *P. aeruginosa* biofilm aggregates and served as a negative control. One representative image of stained aggregates is shown. *P. aeruginosa* expressing mCherry from pMP7605^56^ is displayed in red, and fluorescein conjugates are displayed in green. Scale bar = 50 μm.

The ability of LecA- and LecB-targeting probes to image *P. aeruginosa* biofilms was evaluated *in vitro*. *P. aeruginosa* PAO1, constitutively expressing mCherry from plasmid pMP7605,^56^ was cultivated in microtiter plates under shaking conditions to form biofilm aggregates, as reported previously.^43^ Fluorescein-carbohydrate conjugates were added to the bacterial culture to reach a final concentration of 10 μM and biofilms were analyzed by confocal laser scanning microscopy (CLSM) under static conditions (Figures 2 and S4). Galactosides **2** and **3** targeting LecA showed staining of *P. aeruginosa* biofilm aggregates (Figures 2A and S5). Similarly, C-fucosides **6** and **7** targeting LecB accumulated at the biofilm aggregates, although with weaker staining compared to other probes (Figures 2B and S4). To our surprise, the intended negative control xyloside **8** also stained the *P. aeruginosa* biofilms (Figures 2C and S4). In all cases, the green signal originating from the fluorescein conjugates was not homogeneously spread over the entire structure of the biofilm aggregates, but a certain heterogeneity was observed across biofilm regions as bright spots. Further, the intensity of staining varied between replicates (see the Supporting Information for individual experiments). On the other hand, azido fluorescein **9** devoid of a carbohydrate moiety did not stain the biofilm in contrast to the observed positive staining for all carbohydrate conjugates (Figures 2D and S4). Instead, only a very faint green shadow was detected for **9** indicating its suitability as a negative control. Thus, the observed biofilm staining with imaging probes **2**, **3**, **6**, **7** and also xyloside **8** was carbohydrate dependent and while **2**, **3** and **6**, **7** are ligands of LecA and LecB, respectively, the molecular mechanism of xyloside **8** remains elusive. To enhance the staining, we increased the concentration of the fluorescein-carbohydrate conjugates from 10 μM to 40 μM, which unfortunately did not improve the contrast and, in a few cases, even resulted in unspecific binding of azide **9** to the biofilm (Figure S5, and S6).

### Divalent LecA-Targeting Probes

In order to enhance staining efficiency and specificity, we aimed to increase the compound’s binding potency through multivalent display of the binding epitopes. LecA forms a homotetramer with favorably oriented binding sites for divalent binding.^57^ Therefore, this approach is particularly attractive for LecA, while for LecB this is not suitable due to unfavorable orientation of its binding sites. We have reported low-nanomolar divalent LecA ligands^58^ that could be exploited for biofilm imaging after synthetic modification with fluorophores (Figure S1).

Synthesis of a branched acetal derivative was performed, and its biophysical evaluation demonstrated that the introduction of a fluorescent label to the divalent ligand was tolerated by LecA (Scheme S5 and Figure S7). The resulting first divalent fluorescent LecA ligand retained low-nanomolar binding affinity but unfortunately showed decomposition in aqueous buffers (Figures S8–S12).

**Scheme 1:**
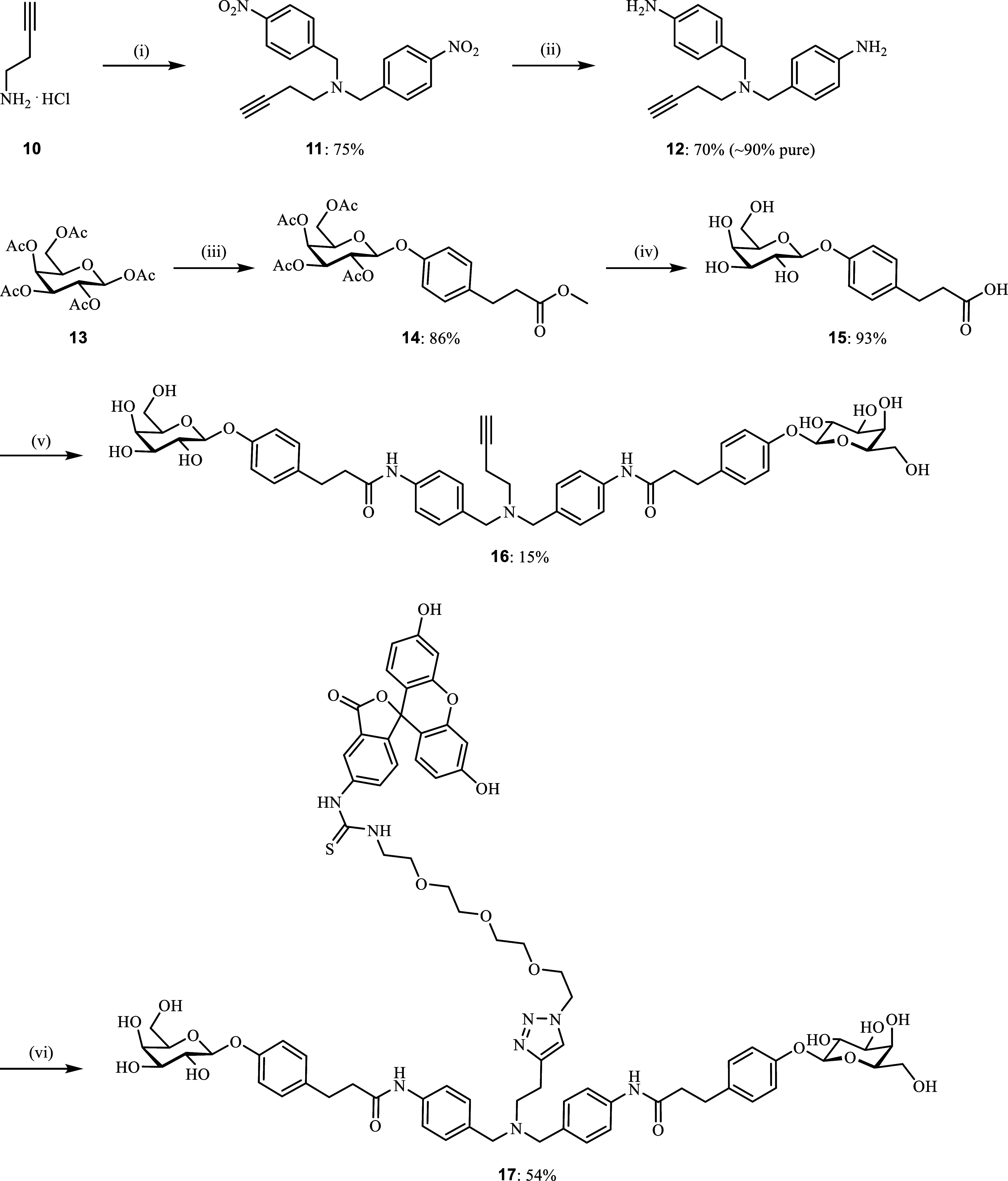
Synthesis of the Optimized Divalent Fluorescent LecA Ligand Reagents and conditions: (i) 4-nitrobenzyl bromide, K_2_CO_3_, r.t., DMF, overnight; (ii) Fe, CaCl_2_, EtOH/H_2_O, 40 °C – r.t., 9 d; (iii) methyl 3-(4-hydroxyphenyl)propanoate, BF_3_**·**Et_2_O, CH_2_Cl_2_, 0 °C – r.t., overnight; (iv) NaOH, H_2_O/MeOH (1:1), r.t., 1 h. (v) **12**, HBTU, DIPEA, DMF, r.t., 2 d; (vi) **9**, CuSO_4_, sodium ascorbate, DMF/H_2_O, r.t.–35 °C, 6 d.

Recently, we reported the optimization of the divalent LecA ligands where the acylhydrazone linker motif was isosterically substituted with amides, improving compound stability.^59^ Therefore, we also optimized the stability of our divalent imaging compound for LecA, replaced the acylhydrazone motif with a more stable amide bond, and introduced a central nitrogen atom in compound **17** to provide a stable tertiary amine as a branching point for fluorophore attachment. The synthesis of divalent imaging probe **17** started with a double alkylation of but-3-yn-1-amine (**10**) with 4-nitrobenzyl bromide (Scheme 1). Selective reduction of the bis-nitro intermediate **11** with iron powder gave the desired bis-aniline linker **12,** which was then coupled to galactosylated acid **15**. The latter was obtained after β-selective glycosylation of methyl 3-(4-hydroxyphenyl)propanoate with β-d-galactose pentaacetate (**13**) under Lewis acid catalysis to give β-galactoside **14** in good yield (86%) followed by saponification with aqueous NaOH (**14** → **15**, 93%). O-glycosidic linkages were used for this ligand since our previous work demonstrated their stability in human and murine plasma and in the presence of liver microsomes from both species.^59^

The assembly of divalent ligand **16** by amide coupling of **12** and **15** was successful, albeit in low yields (15%) due to impure starting material **12**, as well as the side product formation. Despite a slow turnover during the subsequent Huisgen dipolar cycloaddition reaction between divalent ligand **16** and azido fluorescein **9**, possibly due to copper coordination to the reactants, optimized divalent fluorescent ligand **17** was obtained in 54% yield.

**Figure 3:**
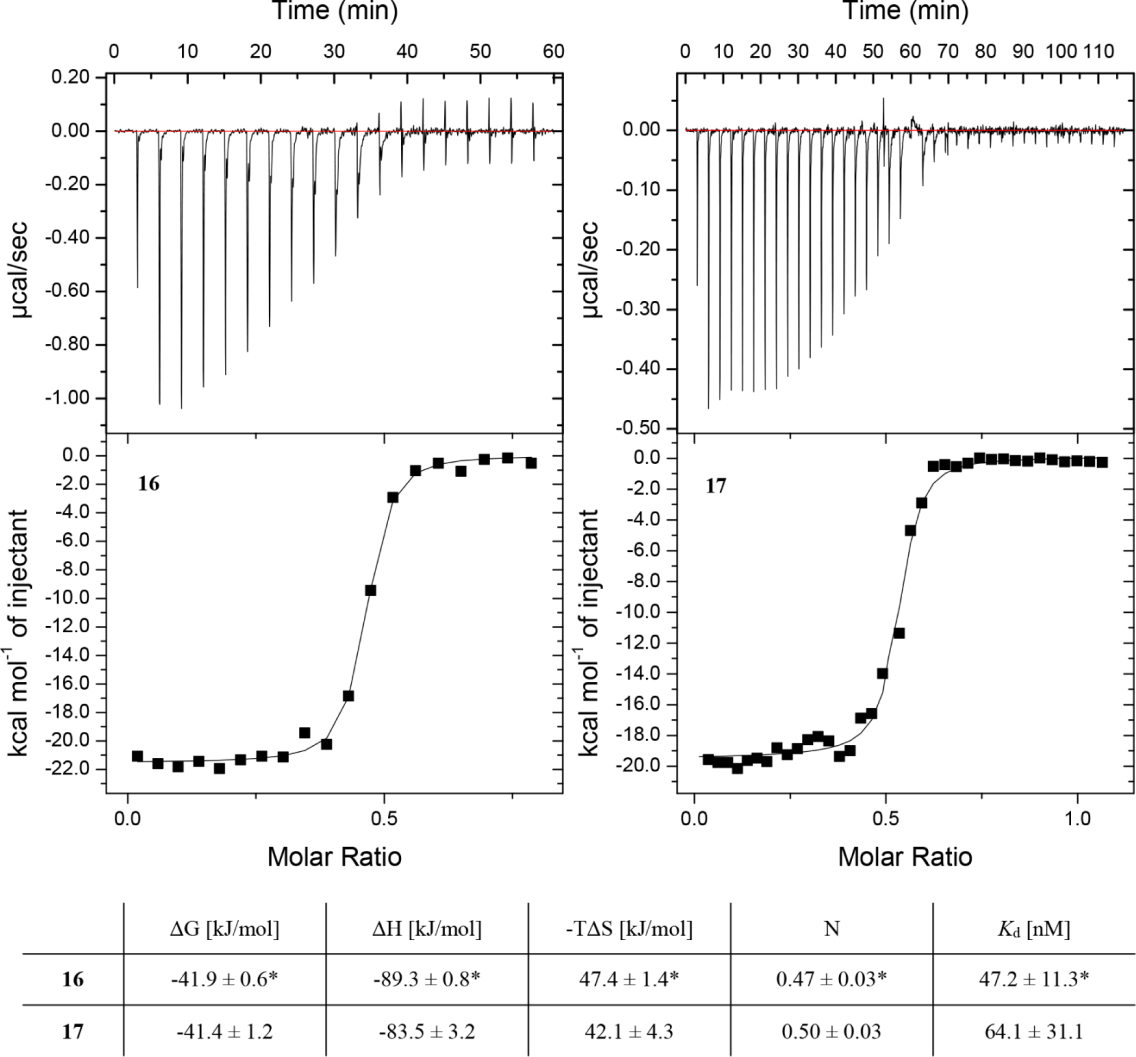
Evaluation of the optimized fluorescent ligand **17** and its synthetic precursor **16** in LecA binding using thermodynamic analysis by ITC. Data for one representative experiment are depicted. Averages and standard deviation from at least three independent experiments (*two independent experiments).

Next, binding of the optimized divalent ligand **17** and its synthetic precursor **16** to LecA was evaluated by fluorescence polarization-based (FP) assays,^47^ SPR, and ITC. In the competitive binding assay by FP, a steep titration slope was observed for the synthetic precursor **16** compared to the assay control *para*-nitrophenyl β-d-galactoside (IC_50_ = 39.7 ± 11.5 μM), indicating that the lower assay limit was reached as reported previously with nanomolar divalent LecA ligands (Figure S13A, top).^58^ For **16**, an IC_50_ value of 5.6 μM was fitted in this assay, which corresponds to the used concentration of LecA (20 μM) and thus a likely lower affinity cannot be reliably determined. Therefore, fluorescent ligand **17** was analyzed in a direct binding experiment by FP. Surprisingly, only a micromolar binding affinity of the divalent fluorescent ligand **17** (*K*_d_ = 1.05 ± 0.12 μM) was observed in this assay (Figure S13A, bottom). These data suggested that the divalent ligand **17** was only 5 to 10 times more potent than the monovalent LecA ligands **2**–**4**. On the other hand, evaluation by direct LecA binding using SPR and ITC revealed that both ligands retained the divalent potency boost and reached nanomolar binding affinities to LecA (Figures 3, S13B and S14–S16). The dissociation constants for **16** were 47.2 ± 11.3 nM (ITC) and 47.8 ± 44.1 nM (steady-state SPR), while for **17,** the values were 64.1 ± 31.1 nM and 79.3 ± 24.9 nM (steady-state SPR). For the SPR experiments, K_d_ was also determined by single-cycle kinetic analysis resulting in approximately five times higher affinity, but the data have limited reliability due to suboptimal fit for the kinetic curves (Figures S13B and S14).^58−60^ Furthermore, these SPR and ITC data demonstrated that the attachment of fluorescein to the branched divalent LecA ligand was well tolerated and did not have significant impact on the thermodynamics of binding (**16**: Δ*H* = −89.3 ± 0.8 kJ/mol vs **17**: Δ*H* = −83.5 ± 3.2 kJ/mol, **16**: -TΔ*S*= 47.4 ± 1.4 kJ/mol vs **17**: -TΔ*S*= 42.1 ± 4.3 kJ/mol). Since SPR and ITC consistently showed low-nanomolar affinities for **17**, it is conceivable that a cross-linking of LecA tetramers and/or their aggregation caused by the divalent display of galactosides in ligand **17** influenced fluorescence polarization and thus a lower binding affinity was observed in FP when compared to SPR and ITC.

**Scheme 2:**
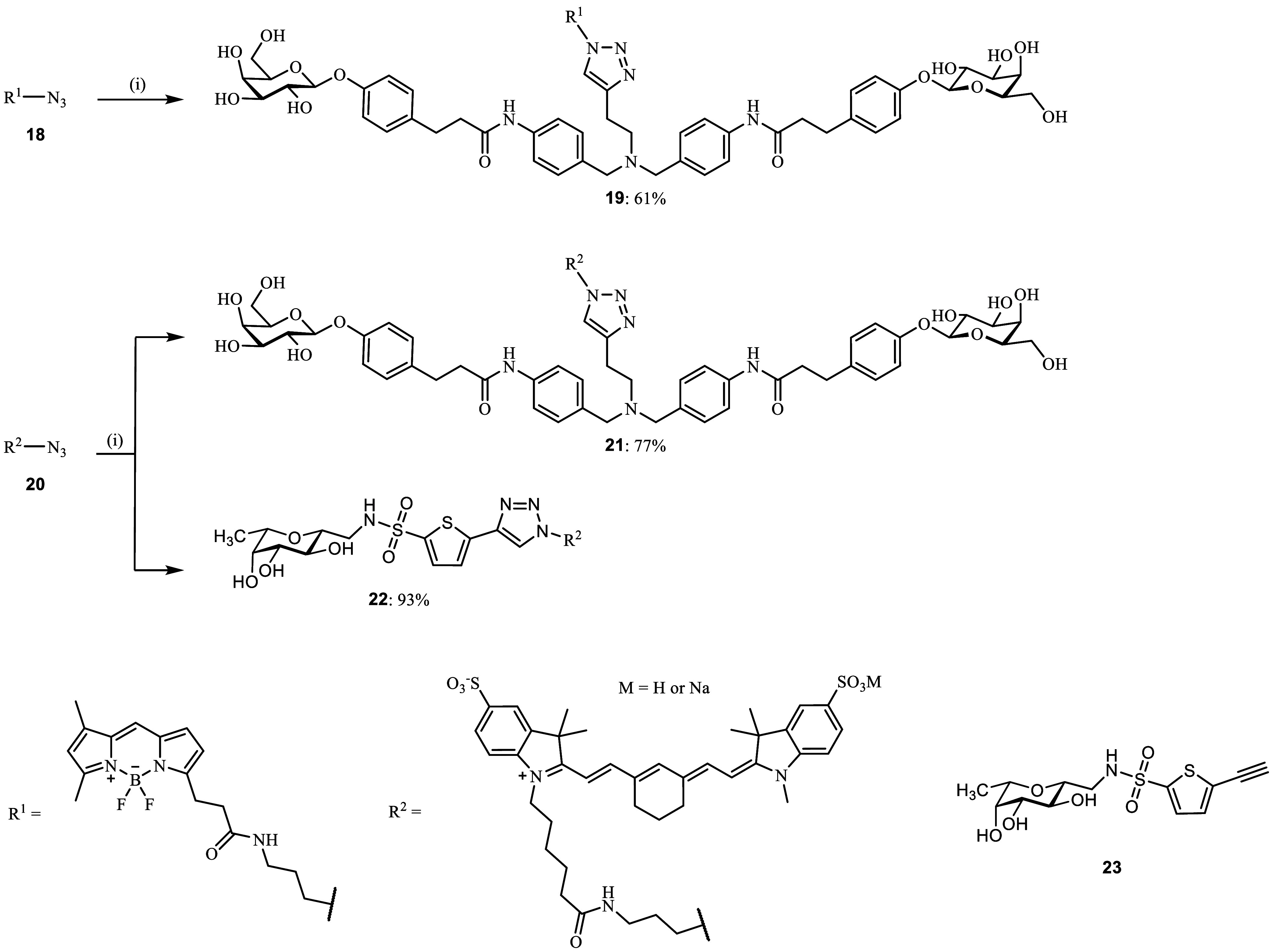
Conjugation of LecA- and LecB-targeting probes with BODIPY and/or sulfo-Cyanine7 dyes Reagents and conditions: (i) **16** or **23**, CuSO_4_, sodium ascorbate, DMF/H_2_O, r.t., 3 h overnight.

The design of divalent LecA ligands allows replacement of the fluorophore moiety with other cargo in the last synthetic step. In order to account for physicochemical properties of the fluorophore and analyze the effect of the identity of the attached dye on the staining of *P. aeruginosa* biofilms, we exchanged the negatively charged fluorescein with a neutral BODIPY dye in compound **19** (Scheme 2). Furthermore, divalent LecA-targeting probe **21** and LecB-targeting probe **22** were synthesized and equipped with the near-infrared dye sulfo-Cyanine7 for *in vivo* experiments (Scheme 2).

### *In Vitro* Biofilm Imaging under Flow Conditions

In a clinical setting, bacteria can adhere to the surface of medical devices or the vasculature of the host, leading to biofilm growth under flow conditions.^61,62^ Reportedly, biofilm formation under flow conditions can alter bacterial metabolism,^63^ gene expression,^64^ morphology,^65^ and growth rate.^63^ To test if our imaging probes can stain biofilms under flow conditions, we established *P. aeruginosa* biofilms using a microfluidics device. Furthermore, the weak staining contrast observed in the static biofilm assay should be overcome by the clearance of unbound dye under flow conditions, whereas the potent LecA-targeting compounds should be retained through binding to their target lectin embedded in the biofilm matrix.

*P. aeruginosa* biofilms were grown under flow conditions and observed by fluorescence microscopy in analogy to Ghanbari et al.^66^ To this end, *P. aeruginosa* PAO1 expressing the red fluorescent protein mCherry^56^ was diluted to an OD_600 nm_ of 0.1 and injected into the microfluidics device. After a 30-min settling period, biofilms were grown under a constant laminar flow rate of 3 mL/h per channel. Following 48 h of incubation at 30 °C, dense and mature *P. aeruginosa* PAO1 biofilms were observed, which were then further used for analysis of the imaging probes.

**Figure 4:**
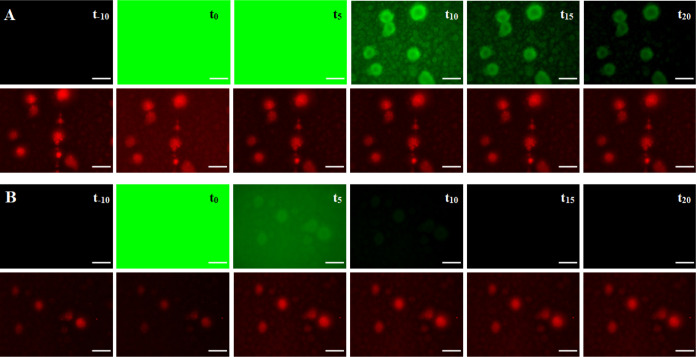
Staining of *P. aeruginosa* PAO1 biofilms under flow following the injection method. Fluorescent compound (stocks of 500 μM **17** or **24**) was injected into the tubings upstream of the flow cell channel, resulting in a final concentration of 17 μM within the channel. Pictures of the same aggregates were recorded prior to compound injection (*t*_−10_), during a 10 min incubation period without flow (*t*_0_) and during the wash period (constant flow, 3 mL/h) at different time points *t*_5_, *t*_10_, *t*_15_, and *t*_20_ (5, 10, 15, and 20 min) using a fluorescence microscope. The green signal corresponds to fluorescently labeled divalent LecA ligand **17** (A) or to the control fluorescein **24** (B). *P. aeruginosa* expressing mCherry from pMP7605^56^ is shown in red. Scale bar = 100 μm.

Two different staining methods were evaluated for the imaging of *P. aeruginosa* PAO1 biofilms: (I) direct injection of the imaging compounds into the flow system or (II) compound supplementation into the medium and gradual accumulation at the biofilm (Figure S18). With the injection method, either bivalent LecA-targeting probe **17** or fluorescein **24** as a control was manually injected into the tubing upstream of the flow cell with biofilms. Final concentrations used for the imaging probes were 17 μM, 8.5 μM, or 3.4 μM inside the flow cell. The flow of compounds **17** and **24** and their arrival in the flow cells were monitored using a fluorescence microscope attached to the flow system. Subsequently, the flow was stopped for an incubation period of 10 min (*t* = 0) and thereafter resumed to clear unbound probes. Biofilm staining was observed for a period of 20 min using the fluorescence microscope. At *t* = 0, divalent LecA-targeting probe **17** and fluorescein control **24** showed strong fluorescence in the flow cell, while after 10 min medium flow clear differences became evident (Figures 4 and S21). The fluorescence originating from control **24** rapidly decreased over time and completely disappeared after 10 min, while the LecA-targeting probe **17** was retained at the *P. aeruginosa* biofilm aggregates and a staining was observed. The larger biofilm aggregates remained stained after 20 min continuous flow, but signal intensity decreased over time due to the flow. Despite these successful results, the direct injection staining method was laborious and technically challenging. The manual injection inevitably perturbed the constant flow, and homogeneous mixing was difficult. Sometimes a heterogeneous fluorescence distribution within the flow cell occurred already during the incubation period, which consequently resulted in unequal staining at different positions within the same channel.

**Figure 5:**
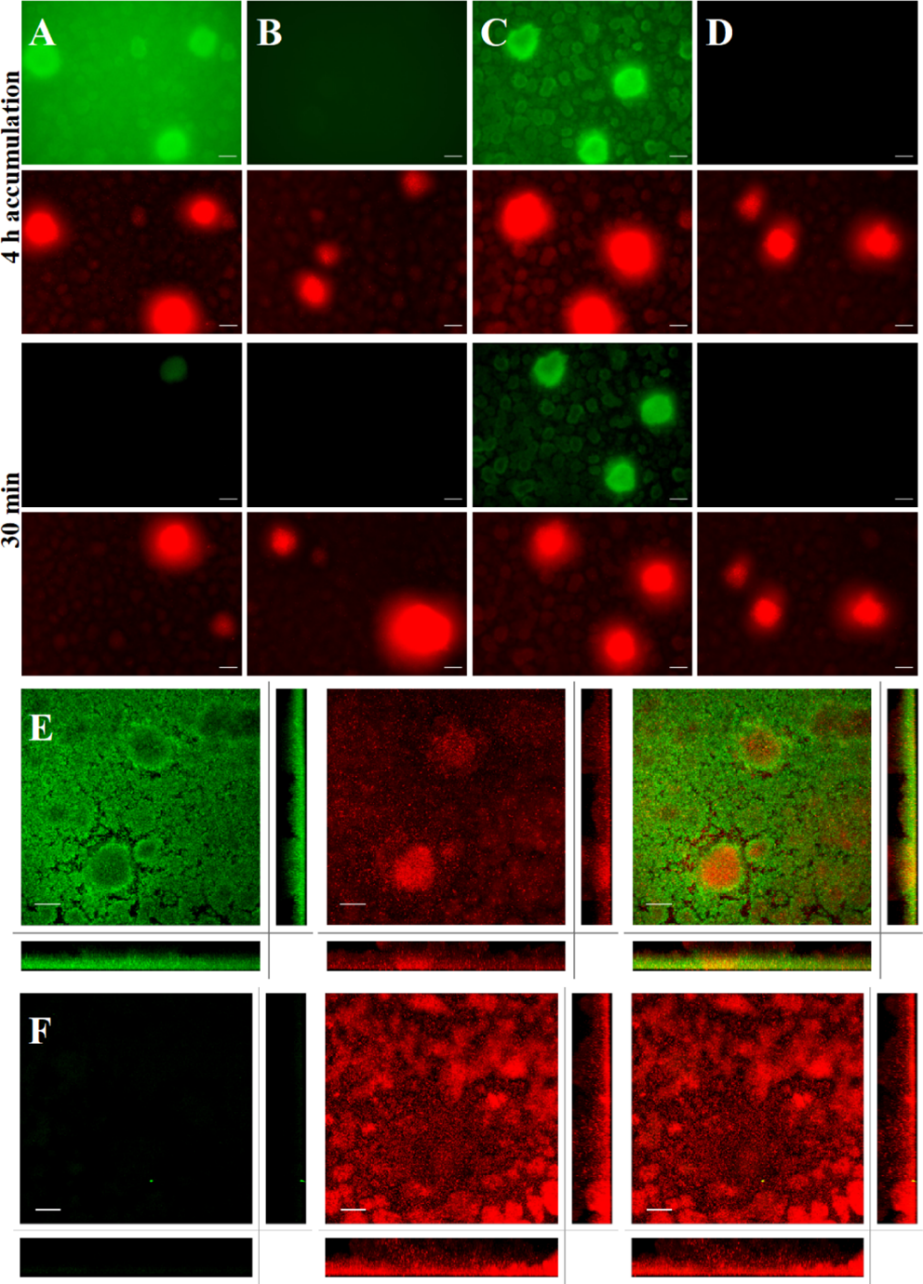
Staining of *P. aeruginosa* PAO1 biofilms grown under flow conditions with compound supplementation to the medium. Biofilm staining with (A) fluorescein divalent probe **17**, (B) fluorescein control **24**, (C) BODIPY divalent probe **19**, and (D) BDP FL azide control **18** was monitored after 4 h of accumulation under constant flow (3 mL/h) of LB media containing 500 nM of respective compound. Retention of imaging compounds is shown after a subsequent wash period for 30 min with medium (3 mL/h, no compound). Images were recorded by using a fluorescence microscope. CLSM was performed for (E) BODIPY divalent probe **19** and the control (F) BDP FL azide **18** after 4 h of accumulation. Green signal originates from fluorescently labeled probes **17** and **19** and controls **24** and **18**. *P. aeruginosa* constitutively expressing mCherry from pMP7605^56^ is shown in red. Z-Stack size, 2 μm. Scale bar = 50 μm.

To circumvent these technical drawbacks, the addition of compounds to the medium reservoir and retention at the biofilm was pursued. Imaging compounds were added to the medium reservoirs at a concentration of 500 nM, which were continuously pumped (3 mL/h) through the flow cell for 4 h. Compound accumulation at mature *P. aeruginosa* biofilms was immediately analyzed using fluorescence microscopy. Afterwards, the medium was exchanged, and the biofilms were washed for 30 min (3 mL/h, no compound) to monitor the retention (Figure 5). Furthermore, an analysis using CLSM was also performed after 4 h of accumulation without washing (Figures 5 and S22).

Divalent LecA-targeting probe **17** successfully stained PAO1 biofilms, as indicated by the observed enrichment at the *P. aeruginosa* biofilm structures and aggregates (Figure 5A) and larger biofilm aggregates were stained intensively. Analogous accumulation experiments using the control fluorescein **24** did not result in any observable staining (Figure 5B). After 30 min of washing, the fluorescence from compound **17** at the biofilm was still visible at a lower intensity, whereas the signal from fluorescein **24** was not detected.

Then, biofilm staining with a divalent LecA probe equipped with the BODIPY fluorophore **19** was analyzed to test whether staining is independent of the fluorophore. In the case of **19**, strong staining was observed after 4 h accumulation at the *P. aeruginosa* biofilm (Figure 5C). Importantly, a high contrast was achieved and even smaller biofilm structures well resolved at the fluorescence microscope. After 30 min of washing, the strong signal was still observed, indicating a good retention of **19** at the *P. aeruginosa* biofilm. In contrast, the appropriate control BDP FL azide **18** did not show any enrichment, which clearly demonstrates that the lectin-targeting moiety is essential for biofilm staining (Figure 5D).

The better staining of the biofilms with BODIPY ligand **19** compared to fluorescein ligand **17** may have resulted from different physicochemical properties of neutral BODIPY and the negatively charged fluorescein moiety. Bacterial biofilms contain polyanions such as extracellular DNA (eDNA) and polysaccharides, e.g., alginate,^67^ which might be responsible for the observed weaker staining by electrostatic repulsion of the negatively charged fluorescein on the divalent LecA ligand **17**. Moreover, BODIPY dyes possess a better photostability compared to fluorescein dyes, which could further account for the more intense staining by BODIPY **19** compared to fluorescein **17**.^68^ However, both LecA-targeting probes showed good efficacy in staining biofilm structures compared to the controls, BDP FL azide (**18**) and fluorescein (**24**), suggesting that chemically diverse fluorophores are acceptable on the divalent LecA-targeting probe.

Considering the results of both staining procedures, supplementation of the probe to the medium offers several advantages over the direct injection method. The addition of the imaging probes to the media is highly reproducible and allows the use of defined concentrations and incubation times and avoids perturbations at the biofilm compared to the laborious and error prone compound injection into the tubing. For the accumulation method, a probe concentration of 500 nM and 4 h of accumulation time gave optimal results. An increased concentration or duration of the experiment may induce morphological changes of the biofilm since the imaging probes **17** and **19** are potent LecA ligands, which could interfere with LecA function, thereby disintegrating the biofilm.

CLSM was used to analyze the staining of *P. aeruginosa* biofilms with the BODIPY imaging probe **19** and the control BDP FL azide (**18)** in more detail (Figures 5E, F and S23). Compounds **18** or **19** were supplemented to the media at 500 nM and after accumulation for 4 h (3 mL/min), the flow cell was disconnected, and the biofilm was analyzed by CLSM without washing. Again, no signal was observed for control BDP FL azide **18**. On the other hand, LecA-targeting probe **19** showed clear and strong staining predominantly at the periphery of large biofilm aggregates, while the smaller biofilm structures were stained homogeneously. These results suggest a low penetration of **19** into the biofilm of large aggregates, possibly as a consequence of slow diffusion. Another explanation may be a heterogeneous distribution of the target LecA within the biofilm aggregates and possibly a localization mainly at the biofilm periphery, as reported for LecB.^69^

### *In Vivo* Imaging of *P. aeruginosa*

Encouraged by the high affinity of the imaging probes and their ability to stain *P. aeruginosa* biofilms *in vitro*, we further investigated the compounds *in vivo* using an acute *P. aeruginosa* airway infection model in mice. Intratracheal administration of *P. aeruginosa* PAO (DSM 1707) results in a local infection that is mainly restricted to the lungs. With the infection dose of 2 × 10^6^ cfu/50 μL chosen here, the number of bacteria peaks at 6 h after infection before the host immune system clears the bacteria.^70^

**Figure 6:**
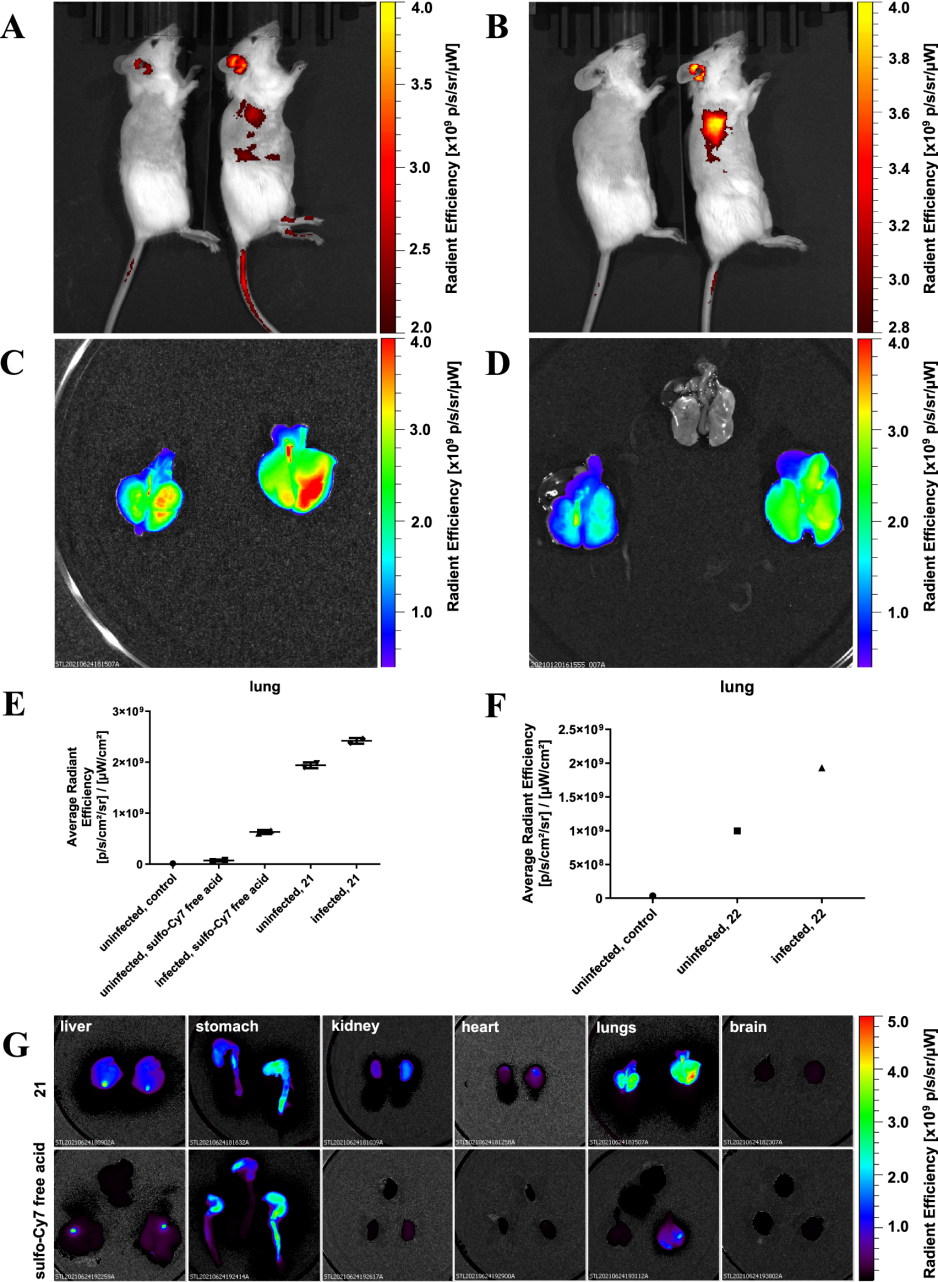
Optical *in vivo* imaging in mice and *ex situ* analysis of organs with *P. aeruginosa* PAO acute lung infection. *In vivo* imaging of mice with *P. aeruginosa* intratracheal infection (right) or uninfected control mice (left) after intravenous administration of imaging probes 2 h post infection: (A) 50 nmol/20 g divalent LecA probe **21** or (B) 100 nmol/20 g LecB probe **22**. Then, explanted lungs were analyzed for fluorescence intensity, shown as a false color scale in (C) divalent LecA probe **21** (left: uninfected, right: infected) and (D) LecB probe **22** (left: uninfected; middle: uninfected control without dye; right: infected). (E) Quantification of the average radiant efficiency of the divalent LecA ligand **21** and the control sulfo-Cy7 free acid in the lungs of uninfected and infected mice. Duplicate data points and the mean are depicted. (F) Average radiant efficiency was quantified for the LecB imaging probe **22** in lungs of infected and uninfected mice. In addition, (G) further mouse organs were explanted after i.v. injection of **21** (50 nmol/20 g) or control sulfo-Cy7 free acid (50 nmol/20 g) and imaged. Organs of uninfected (left), infected (right) mice, and uninfected mice without any dye (upper middle on the sulfo-Cy7 free acid rows) were compared. Fluorescence intensities are shown as false color pictures.

Then, imaging probes, divalent LecA-targeting probe **21**, LecB-targeting probe **22** and the control sulfo-Cy7 (free acid), were administered intravenously to the tail vein 2 h post infection. Optical *in vivo* imaging allowed the detection of elevated Cyanine7 signals in the area of the lung of infected mice compared to uninfected mice for LecA-targeting compound **21** (Figure 6A) and LecB-targeting compound **22** (Figure 6B). This accumulation in the lungs was then confirmed by a subsequent *ex vivo* analysis of the organs. Average fluorescence radiant efficiency for infected mice was elevated compared with uninfected mice (Figure 6C,D). The infection is restricted to the lung, but a marginally increased signal in heart, stomach/gut and kidney of infected mice suggests a reduced clearance of the dye likely due to acute host symptoms of the infection.

Interestingly, when quantifying the fluorescence *ex vivo*, the LecA-targeting compound **21** (Figures 6G and S26) as well as the LecB-targeting compound **22** (Figure S24) showed already a certain accumulation in the lungs of uninfected animals compared to the control sulfo-Cyanine7 (free acid). Further, the kidneys of infected animals that received compound **21** also showed moderately increased signals compared to uninfected animals, although at a much lower fluorescence intensity compared to the lungs (Figures 6G and S25). Further research will be needed to elucidate the basis for these observations.

### *Ex Situ* Lung Imaging of *P. aeruginosa*

**Figure 7:**
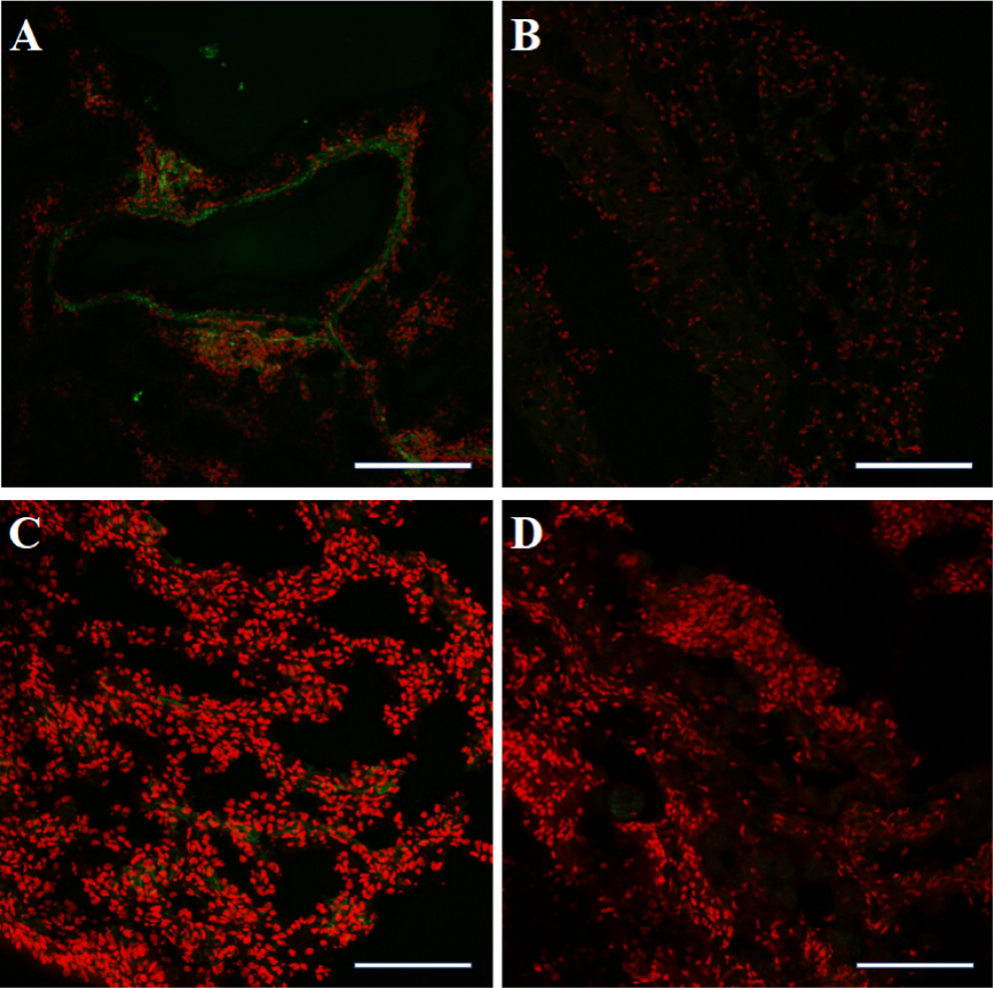
Confocal laser scanning microscopy analysis of *P. aeruginosa* PAO-infected murine lung sections using divalent LecA imaging probe **21**. Sections of cryopreserved murine lungs infected with *P. aeruginosa* were prepared and analyzed by CLSM after intravenous injection of divalent LecA imaging probe **21** (A) or the control Sulfo-Cyanine7 free acid (B). Lung sections of uninfected animals were used as a control after i.v. injection of **21** (C) or control Sulfo-Cyanine7 free acid (D). Cell nuclei are shown in red (DAPI) and green signals correspond to **21** or Sulfo-Cyanine7 free acid, respectively. Green signals were detected only in infected lung sections after injection of divalent LecA ligand **21**. Signals were located in cellular accumulations around medium-sized blood vessels. Scale bar for A and B = 100 μm and for C and D = 50 μM.

For more detailed insights into the localization of divalent LecA ligand **21** and the control Sulfo-Cyanine7, lung sections of infected and uninfected mice were prepared and analyzed by CLSM. Cyanine7 signals were exemplarily visible in infected lung sections from one mouse that received divalent LecA ligand **21** (Figure 7A). Signals corresponding to **21** were localized in cellular accumulations around medium-sized blood vessels. Particle-shaped signals and signals clustered into larger dots were observed. In contrast, no signals were detected in lungs of the uninfected mice that received **21** (Figure 7C), nor in lungs of infected and uninfected animals treated with control Sulfo-Cyanine7 (Figure 7B,D). Possibly, signals detected in lung sections stained with a divalent LecA imaging probe are due to complexes of membrane-bound LecA with **21**. These particle-shaped complexes probably represent the divalent imaging probe **21** bound to bacterial cells. Additionally, it is possible that larger dots are attributed to accumulated bacteria in phagocytes such as neutrophils, monocytes, and dendritic cells. However, intracellular detection using the divalent LecA imaging probe **21** has not yet been analyzed and requires further experiments.

## Conclusion

Clinical identification of the exact site of an infection and the causative bacterial species requires invasive sampling followed by lengthy analysis, which significantly delays the initiation of therapy. The latter often leads to complications. Noninvasive infection imaging is a neglected field, yet it could potentially overcome these hurdles and accelerate diagnostic processes. In this work, we report a set of novel imaging probes for the *in vitro* and *in vivo* imaging of *P. aeruginosavia* targeting its lectins LecA and LecB.

The synthesized monovalent lectin-targeting imaging probes **2**–**4** for LecA and **6**–**7** for LecB showed low micromolar binding affinities for their respective target. When the technique was applied to biofilms of *P. aeruginosa*, a carbohydrate-dependent staining was observed under static conditions *in vitro*. Interestingly, the xylose-based probe **8**, which was initially designed as a negative control, also showed binding to the biofilm despite the absence of binding to purified LecA or LecB proteins. A control molecule without a carbohydrate residue confirmed the carbohydrate dependence of the biofilm staining.

To enhance the binding affinity of the imaging probe into the nanomolar range, a crucial step for potential human applications, LecA-targeting imaging probes were optimized using the concept of multivalency. The divalent fluorescent ligand **17** showed low-nanomolar binding affinity to LecA, as unambiguously determined by SPR and ITC. Using compound **17** for *in vitro* staining of *P. aeruginosa* biofilms under flow conditions demonstrated the suitability of LecA-targeting imaging probes as candidates for diagnostics. Moreover, LecA-dependent biofilm staining under flow conditions was successful independent of the fluorophore moiety, with the BODIPY-conjugated probe **19** showing increased imaging quality when compared to the fluorescein analogue **17**. Therefore, it is conceivable to exchange the fluorophore with a PET tracer or an MRI active agent, enabling future applications in human detection for clinical practice.

Finally, we analyzed the ability of LecA- and LecB-targeting imaging probes to detect *P. aeruginosa* infections in a mouse lung infection model. Intravenous administration of LecB-targeting **22** or divalent LecA ligand **21** resulted in increased fluorescence signals in the lungs of infected mice compared to uninfected mice. Subsequent *ex vivo* examination of lung sections corroborated these observations. This is the first report to show that the detection of *P. aeruginosa* using lectin-targeted imaging probes is also effective *in vivo*.

In summary, lectin-targeting imaging probes have proven to be highly efficient to image *P. aeruginosa* biofilms *in vitro* and are promising leads for the development of fast, noninvasive, and pathogen-specific diagnostic tools for *P. aeruginosa* infections. Future work comprises the analysis of chronic infections *in vivo* in appropriate animal models and their extension to PET and MRI imaging.

## Supporting Information Available

Experimental details, protocols on assays, bacterial cultivation, infection experiments, and imaging. Description of the synthesis of all compounds and analytical data including NMR transcripts. Supplementary figures comprise the biophysical evaluation of mono- and divalent lectin ligands by FP, SPR, and ITC, *in vitro* biofilm static and flow biofilm staining, absorption and emission spectra of fluorescent ligands, as well as additional figures on *in vivo* imaging (PDF)

## Notes

Commercial compounds served as controls: fluorescein 24 (CAS no.: 518-47-8, CarlRoth), sulfoCy7 free acid (CAS no.: 2104632-30-4, Lumiprobe); sulfoCy7 azide 20 (CAS: n.a., Cat. No.: E5330, Lumiprobe).
