# Supplementary material for: High-Affinity Lectin Ligands Enable the Detection of Pathogenic Pseudomonas aeruginosa Biofilms: Implications for Diagnostics and Therapy
Source: JACS Au. 2024 Dec 3;4(12):4715–28. doi: 10.1021/jacsau.4c00670 (PMC11672137; doi:10.1021/jacsau.4c00670)
Supplement: Supplementary file 1 — au4c00670_si_001.pdf [file au4c00670_si_001.pdf]

Supporting Information

High Affinity Lectin-ligands Enable Detection of Pathogenic *Pseudomonas aeruginosa* Biofilms - Implications for Diagnostic and Therapy

Eva Zahorska[1-4$], Lisa Marie Denig[1-4$], Stefan Lienenklaus[5], Sakonwan Kuhaudomlarp[6,7,8], Thomas Tschernig[9], Peter Lipp[10], Antje Munder[11,12], Emilie Gillon[6], Saverio Minervini[1], Varvara Verkhova[1-4], Anne Imberty[6], Stefanie Wagner[1-4], Alexander Titz[1-4*]

[1]Chemical Biology of Carbohydrates (CBCH), Helmholtz Institute for Pharmaceutical Research Saarland (HIPS), Helmholtz Centre for Infection Research, D-66123 Saarbrücken, Germany.
[2]Deutsches Zentrum für Infektionsforschung (DZIF), Standort Hannover-Braunschweig.
[3]Department of Chemistry, Saarland University, D-66123 Saarbrücken, Germany.
[4]PharmaScienceHub, Saarland University, D-66123 Saarbrücken, Germany.
[5]Hannover Medical School, Institute of Laboratory Animal Science, 30625 Hannover, Germany.
[6]Université Grenoble Alpes, CNRS, CERMAV, 38000 Grenoble, France.
[7]Department of Biochemistry, Faculty of Science, Mahidol University, Bangkok 10400, Thailand.
[8]Center for Excellence in Protein and Enzyme Technology, Faculty of Science, Mahidol University, Bangkok 10400, Thailand.
[9]Institute of Anatomy and Cell Biology, Medical Faculty of Saarland University, D-66421 Homburg/Saar, Germany.
[10]Center for Molecular Signaling (PZMS), Medical Faculty of Saarland University, D-66421 Homburg/Saar, Germany.
[11]Department of Pediatric Pneumology, Allergology and Neonatology, Hannover Medical School, Carl-Neuberg-Str. 1, D-30625 Hannover, Germany.
[12]Biomedical Research in Endstage and Obstructive Lung Disease Hannover (BREATH), Member of the German Center for Lung Research (DZL), D-30625 Hannover, Germany

[$]these authors contributed equally
*corresponding author: alexander.titz@helmholtz-hzi.de

Table of contents

Compounds synthesis 3
Chemical synthesis – Experimental details 9
Biophysical evaluation 42
Bacterial cultivation 45
In vitro biofilm staining under static conditions 45
Stability and LecA binding and inhibition of divalent LecA ligands 48
In vitro biofilm staining under flow conditions 57
Analysis of excitation and emission maxima of the divalent LecA targeting imaging probes 58
Determination of fluorescence intensities 59
Lung infection model and *in vivo* imaging of *P. aeruginosa* 62
Lung sections and *ex vivo* imaging of *P. aeruginosa* 65
NMR spectra 66
REFERENCES 115

Compounds synthesis

Synthesis of galactose-based alkyne precursors with amine linker (**S5*m***, **S5*p***, **S6*m***, **S6*p***) were synthesized in four linear steps, whereas their ether analogue (**S11**) in convergent manner (Scheme S1). Lewis acid promoted glycosylation of corresponding thiol acceptors gave glycosides **S1*m***, **S1*p***, **S10** in fair yields (39–60%). Disulfide formation was used as a provisional protecting group for O-alkylation to intermediate **S8** during synthesis of the glycosyl acceptor **S9**. Disulfide reduction conditions using sodium borohydride or triphenyl phosphine were tested, however the reaction with dithiothreitol was the most successful to obtain thiol **S9** (fast and not requiring a chromatographic purification). Palladium catalyzed hydrogenation of the nitro intermediates gave anilines **S2*m*** and **S2*p*** quantitatively. N-alkylations yielding **S3*m***, **S3*p***, **S4*m*** and **S4*p*** had to be closely monitored to minimize the production of undesired tertiary amines (characterized as **S27*m***, **S27*p***, **S28*p***). Furthermore, carbamate side products were detected during the alkylations with 4-bromo-1-butyne (**S29*p***), when potassium carbonate base served as a carbon dioxide source. Deprotection under Zemplén conditions gave the desired terminal alkyne containing precursors for imaging probes, galactosides **S5*m***, **S5*p***, **S6*m***, **S6*p*** and **S11**.

Synthesis of alkyne C-glycoside precursors targeting LecB started from β-C-glycoside **S14**[1] that was coupled to the corresponding sulfonyl chloride, yielding intermediate **S15** and imaging probe precursor **S16** (Scheme S2). Benzenesulfonyl chloride building block **S13** was prepared by chlorosulfonation of propargyl phenyl ether **S12**, whereas thiophene building block was commercially available. Palladium-catalysed Sonogashira cross coupling reaction with **S15** and the protected acetylene followed by desilylation gave imaging probe precursor **25** in 50% yield (over 2 steps).

Synthesis of xylose-based precursors was performed in analogy to galactose-based alkyne precursors (Scheme S3). Peracetylation step of D-xylose (**S17**) yielded β-D-xylopyranose tetraacetate (**S18**, 39%) and was followed by Lewis acid promoted glycosylation of 4-nitrophenyl thiol or thiol **S9** to get xylosides **S19** (44%) and **S23** (12%). The poor isolated yield of **S23** was caused by limited separation of anomers during normal phase chromatographic purification. Palladium-catalyzed hydrogenation of **S19** gave the corresponding aniline **S20** quantitatively. Afterwards, N-alkylation with propargyl bromide yielded **S21** (63%). Finally, deprotection under Zemplén conditions was used to obtain xyloside-based alkyne precursors **S22** and **S24**.

Galactose-based precursors with amine linker

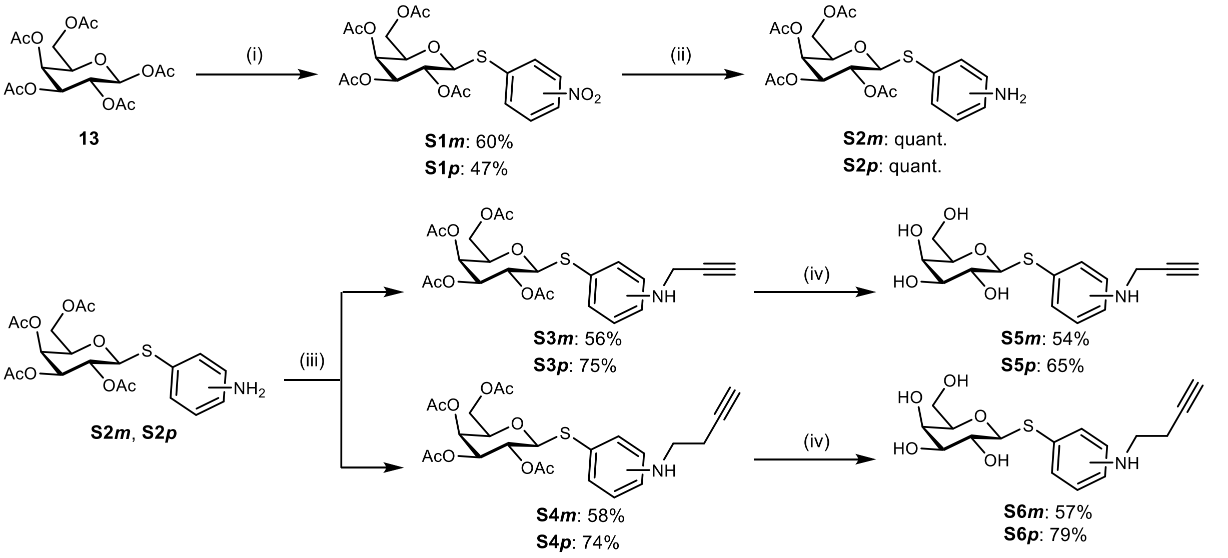

Galactose-based precursors with ether linker

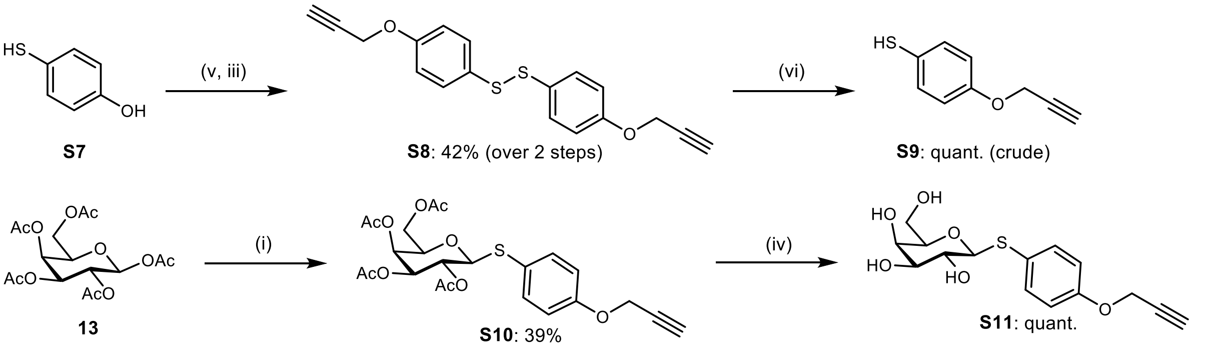

Scheme S1: Synthesis of alkyne precursors for LecA targeting galactose-based imaging probes. Reagents and conditions: (i) *m*-nitrothiophenol/*p*-nitrothiophenol/4-prop-2-ynyloxyphenyl thiol (**S7**), $BF_3{\cdot}Et_2O$, CH_2Cl_2, 0 °C – r.t., o.n.; (ii) H_2, Pd/C, CH_2Cl_2, r.t., o.n.; (iii) propargyl bromide/4-bromo-1-butyne, K_2CO_3, DMF, 0 – 40 °C (for **S4*m*** and **S4*p***: 0 – 70 °C), 6 h – 4 d; (iv) NaOMe, MeOH, r.t., 1 – 2 h; (v) I_2, EtOH, r.t., 5 h; (vi) dithiothreitol, *N*-ethyldiisopropylamine, MeOH, r.t., 3 h.

C-glycoside-based precursors

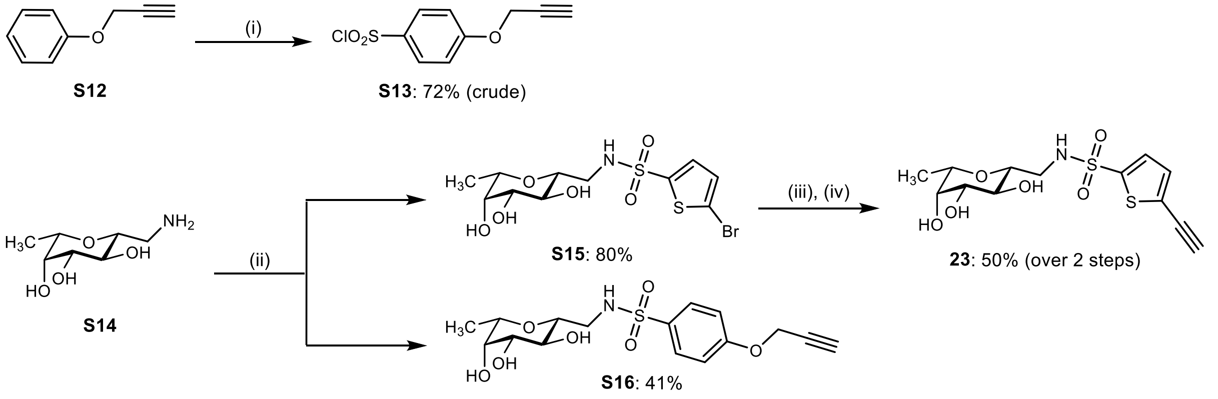

Scheme S2: Synthesis of alkyne precursors for LecB targeting C-glycoside-based imaging probes. Reagents and conditions: (i) $ClSO_3H$, CH_2Cl_2, 0 °C, 2 h; (ii) 5-bromothiophene-2-sulfonylchloride/4-(propargyloxy)benzenesulfonyl chloride (**S13**), Et_3N, DMF, 0 °C – r.t., 2 – 3 h; (iii) trimethylsilyl acetylene, CuI, $PdCl_2(PPh_3)_2$, Et_3N, DMF, 30 °C, 5 h; (iv) K_2CO_3, MeOH, r.t., 3 h.

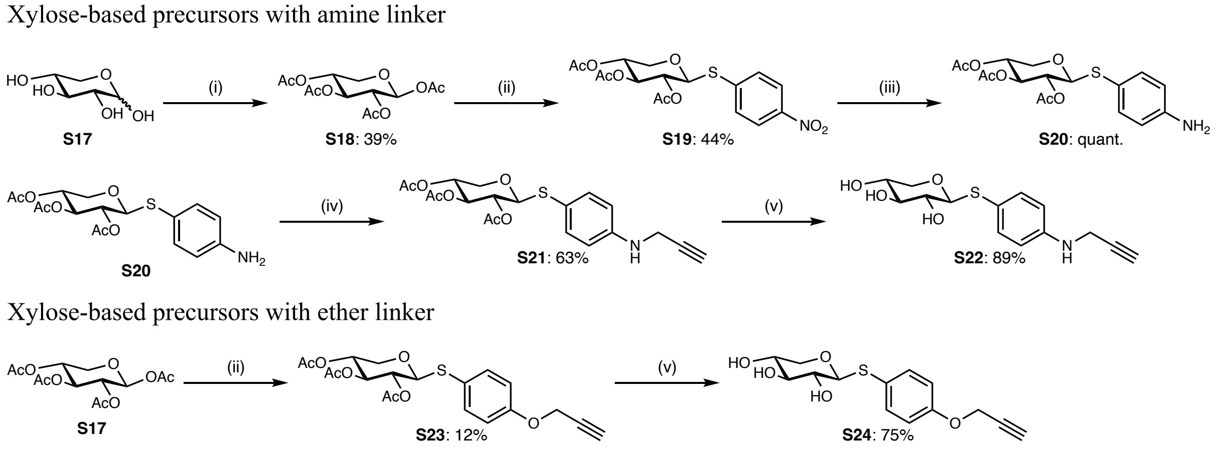

Scheme S3: Synthesis of alkyne precursors for a xylose-based imaging probes. Reagents and conditions: (i) Ac_2O, NaOAc, 100 °C, 20 min; (ii) *p*-nitrothiophenol/4-prop-2-ynyloxyphenyl thiol (**S9**), $BF_3{\cdot}Et_2O$, CH_2Cl_2, 0 °C – r.t., o.n.; (iii) H_2, Pd/C, CH_2Cl_2, r.t., o.n.; (iv) propargyl bromide, K_2CO_3, DMF, 0 – 40 °C, 6 h – o.n.; (v) NaOMe, MeOH, r.t., 1 – 2 h.

Assembly of imaging probes was performed using Huisgen azide-alkyne cycloaddition between alkyne precursors and azide modified fluorescein **9** (Scheme S4). Fluorescein 5-isothiocyanate (**S25**) was coupled to the short flexible polyethylene glycol spacer with terminal azide to give **9** in 59% yield. Based on the results of competitive binding assay showing no difference in activity was for galactosides with extended linker **S6*m*** and **S6*p*** (*vide infra*), only the galactose-based compounds bearing a propargyl handle (**S5*m*** and **S5*p***) were chosen for final conjugation to the fluorophore to get LecA targeting imaging probes. High amounts of copper source were necessary for efficient click reaction turnover, probably due to its coordination to the building blocks. Imaging probes **2**–**4**, **6**–**8** and **S26** were synthesized in fair yields (40-85%).

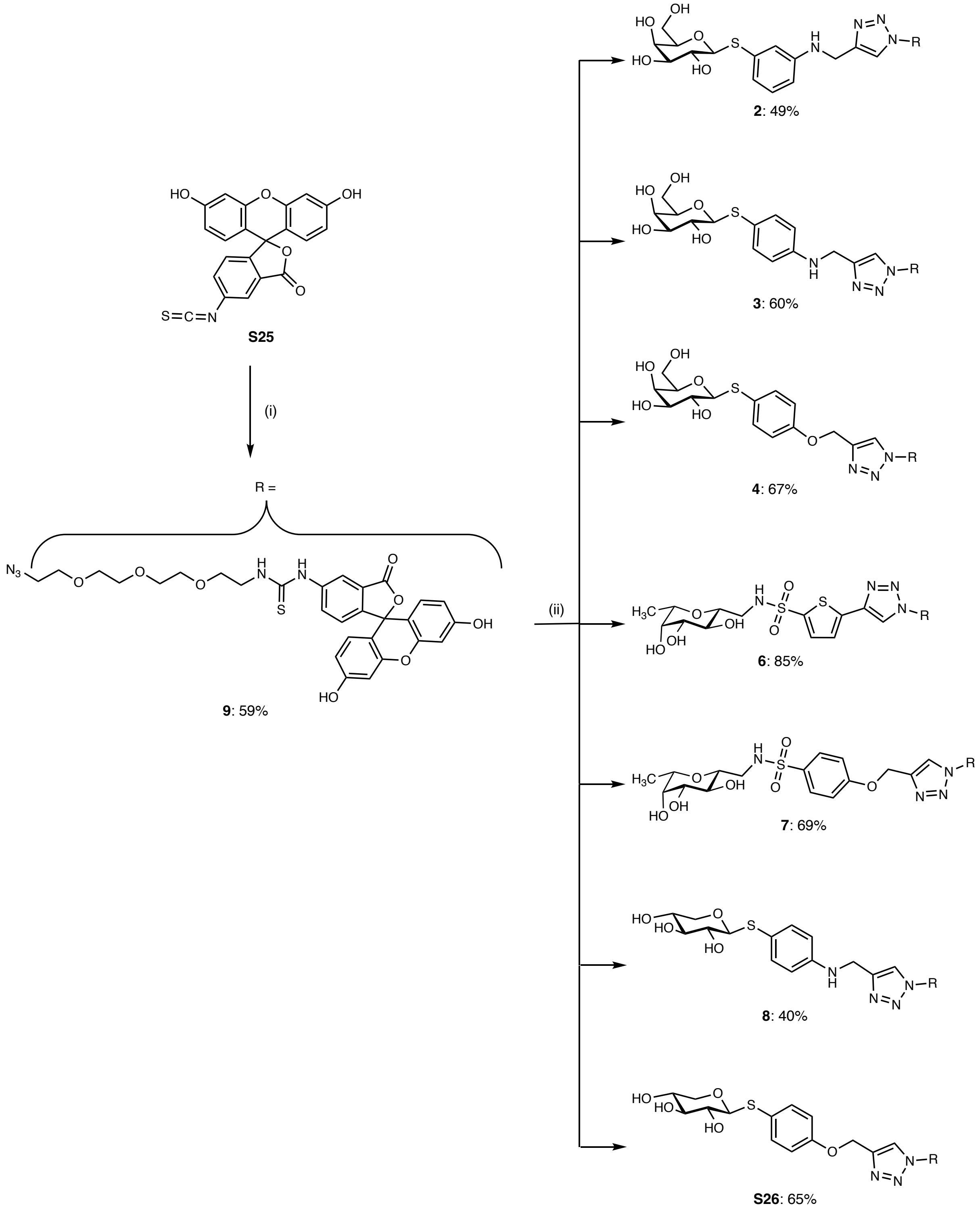

Scheme S4: Assembly of imaging probes. Reagents and conditions: (i) 11-azido-3,6,9-trioxaundecan-1-amine, Et_3N, DMF, 0 °C – r.t., 1 h; (ii) corresponding terminal alkyne (**S5*m***, **S5*p***, **S11**, **25**, **S16**, **S22**, **S24**), $CuSO_4$, sodium ascorbate, DMF/H_2O (4:1), r.t., overnight.

Previous molecular dynamics simulations of acylhydrazone divalent ligand **S30** in complex with LecA showed a possible growth vector for an extension at the center of the ligand (Figure S1). Therefore, compound **S31** was designed and synthesized as the first divalent LecA imaging probe (Scheme S5). Synthesis started with the chlorination of 4-formylbenzoic acid (**S32**) followed by an amide formation with propargyl amine yielding the linker core building block **S33**. A double nucleophilic substitution reaction on **S33** with 4-hydroxybenzaldehyde

gave the new branched linker **S34** in a moderate yield (67%). The bisacylhydrazone formation with the hydrazide bearing galactoside **S35** under acidic conditions yielded divalent ligand **S36**. Huisgen dipolar cycloaddition between crude product **S36** and the azide modified fluorescein **9** was not successful – no product peak was detected by LCMS, despite the consumption of the starting material **S36**. The click reaction was not attempted with purified bis-acylhydrazone **S36**, instead firstly the CuAAC reaction between the azide modified fluorescein **9** and the branched linker **S34** was performed to obtain bis-benzaldehyde fluorescent linker **S37** and then the chemically labile acylhydrazone bonds were formed. Despite nearly quantitative turnover for the condensation reaction between bis-benzaldehyde **S37** and hydrazide bearing galactoside **S35**, divalent fluorescent ligand **S31** was obtained only in 35% yield. The product loss was probably a result of its poor solubility and chemical instability of the acylhydrazone bond. Unfortunately, partial hydrolysis of the acylhydrazone bond was detected after preparative HPLC purification and accounted for approximately 10% impurity in the final compound **S31**.

The binding affinity of **S31** to LecA was determined by SPR (Kd = 37.2 ± 2.9 nM, Figure S7) which showed that introduction of the fluorescence label was well tolerated. Unfortunately, **S31** showed decomposition in aqueous buffers (pH 7.4, Figure S8-S12), likely due to the acetal motif at the branching point and/or hydrolytic lability of the acylhydrazone bond, although compounds **S30** and **S36** were stable under the same conditions.

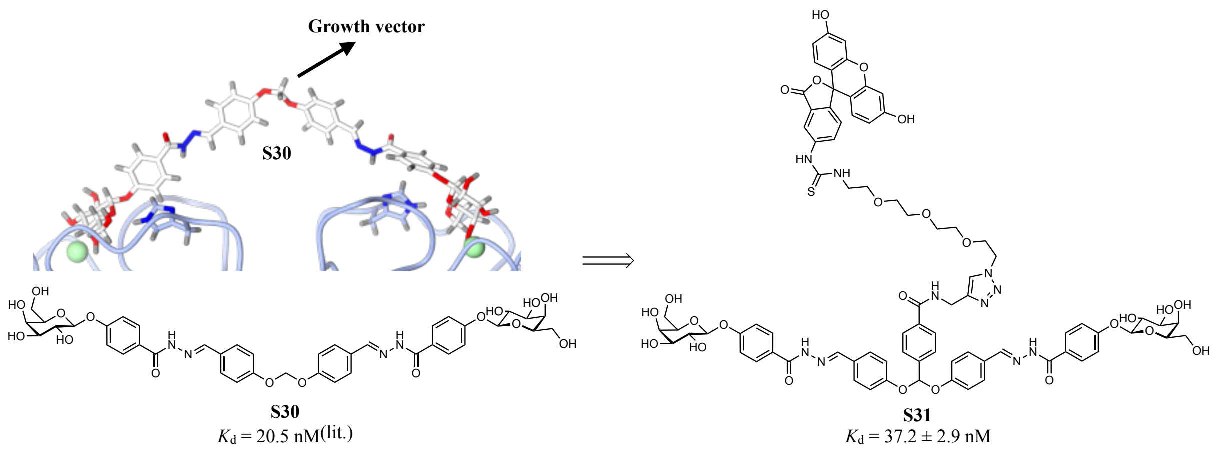

Figure S1: Design of the first generation divalent fluorescent LecA ligand **S31** from published molecular dynamics simulations of compound **S30** in complex with LecA (left, reproduced from Zahorska et. al.)[2] which indicated a possible growth vector for the attachment of imaging agents. Binding affinity of the first divalent fluorescent LecA ligand **S31** (right) was determined by SPR.

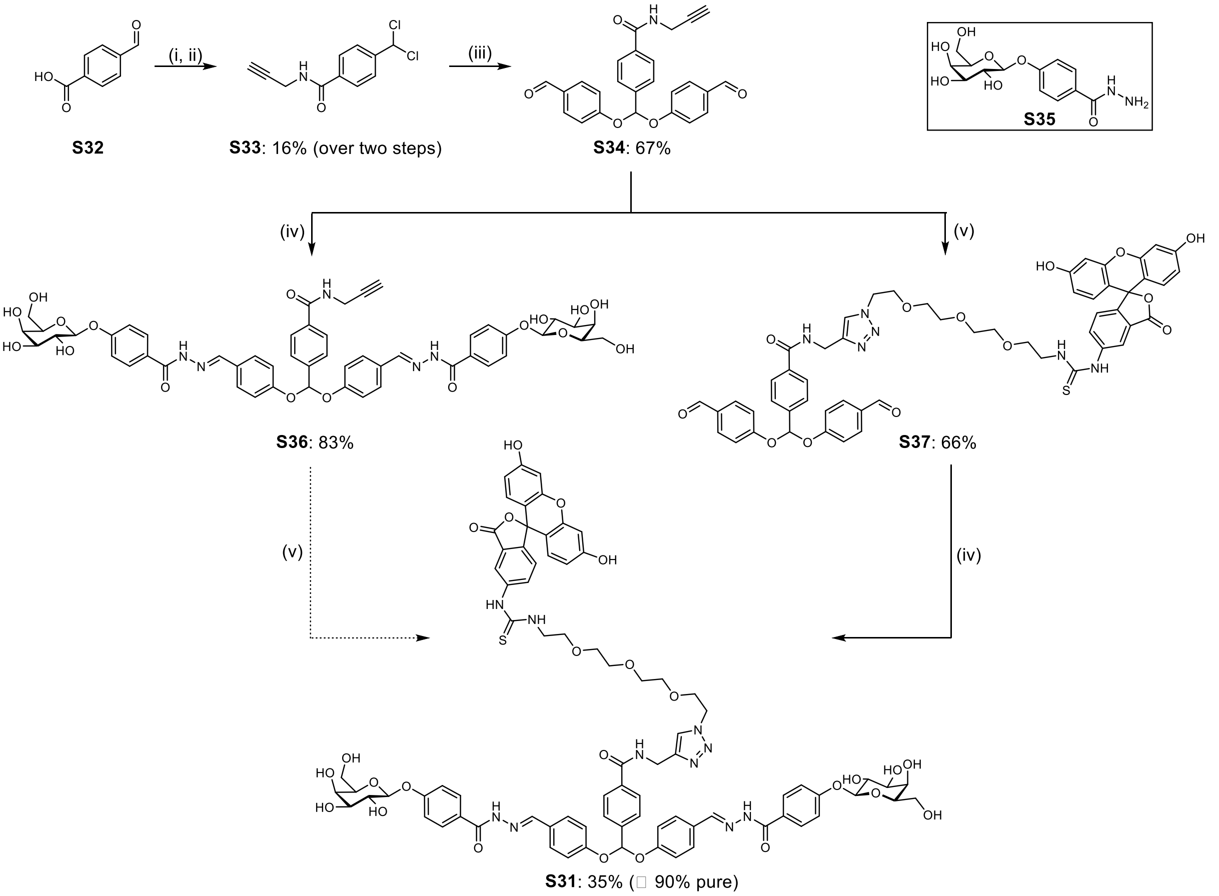

Scheme S5: Synthesis of divalent fluorescent LecA ligand. Reagents and conditions: (i) $SOCl_2$, reflux, 1 d; (ii) propargyl amine, Et_3N, CH_2Cl_2, 0 °C–r.t., 3 h; (iii) 4-hydroxybenzaldehyde, K_2CO_3, DMF, reflux, 2 d; (iv) **S35**[2], formic acid, DMSO, r.t., 2.5 h; (v) **9**, $CuSO_4$, sodium ascorbate, DMF/H_2O/DMSO, r.t., 7.5 h.

Chemical synthesis – Experimental details

Commercial chemicals and solvents were used without further purification.

Thin layer chromatography (TLC) was performed using silica gel 60 aluminum plates containing fluorescence indicator (Merck KGaA, Darmstadt, Germany) and developed under UV light (254 nm) and using a molybdate solution (0.02 M solution of $(NH_4)_4Ce(SO_4)_4 \cdot 2\ H_2O$ and $(NH_4)_6Mo_7O_{24} \cdot 4\ H_2O$ in aqueous 10% H_2SO_4) or a potassium permanganate solution (3 g of $KMnO_4$, 20 g of K_2CO_3 in 5 mL of 5% NaOH and 300 mL of water) with heating.

Medium pressure liquid chromatography (MPLC) was performed on a Teledyne Isco Combiflash Rf200 system using normal phase self-packed silica gel columns (60 Å, 400 mesh particle size, Fluka) or reverse-phase pre-packed silica gel 60 Å columns from Macherey-Nagel (C_{18} ec, endcapped). Preparative high-pressure liquid chromatography (HPLC) was performed on Waters 2545 Binary Gradient Module with a Waters 2489 UV/Vis detector using a RP-18 column (250/21 Nucleodur C18 Gravity SB, 5 µm from Macherey-Nagel, Germany).

Analytical HPLC-MS was performed on a Thermo Dionex Ultimate 3000 HPLC coupled to a Bruker amaZon SL mass spectrometer, with UV detection at 254 nm using a RP-18 column (100/2 Nucleoshell RP18plus, 2.7 µm from Macherey-Nagel, Germany) as stationary phase. High resolution mass spectrometry (HRMS) was performed on an Ultimate 3000 UPLC system coupled to a Q Exactive Focus Orbitrap system with HESI source (Thermo Fisher, Dreieich, Germany). The UPLC was operated with a C18 column (EC 150/2 Nucleodur C18 Pyramid, 3 µm from Macherey-Nagel, Germany).

^{1}H-NMR and ^{13}C-NMR spectra were recorded on a Bruker Avance III 500 UltraShield spectrometer at 500 MHz and 126 MHz. Chemical shifts (δ) are given in ppm and were calibrated on residual solvent peaks: $CDCl_3$ (^{1}H-NMR δ = 7.26 ppm, ^{13}C-NMR δ = 77.0 ppm), MeOH-d_4 (^{1}H-NMR δ = 3.31 ppm, ^{13}C-NMR δ = 49.0 ppm), DMSO-d_6 (^{1}H-NMR δ = 2.50 ppm, ^{13}C-NMR δ = 39.51 ppm), D_2O (^{1}H-NMR δ = 4.79 ppm), acetone-d_6 (^{1}H-NMR δ = 2.05 ppm, ^{13}C-NMR δ = 29.84 ppm, δ = 206.26 ppm).[3] Deuterated solvents were purchased from Eurisotop (Saarbrücken, Germany). Multiplicities are specified as s = singlet, d = doublet, t = triplet, q = quartet, m = multiplet. The spectra were assigned with the help of ^{1}H,^{1}H-COSY; ^{1}H,^{13}C-HSQC and ^{1}H,^{13}C-HMBC experiments.

***m*-Nitrophenyl-thio-β-D-galactopyranose tetraacetate (S1*m*)**

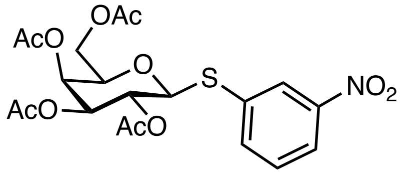

3-Nitrobenzene disulphide (1.28 g, 4.15 mmol) was dissolved in dry tetrahydrofuran (5 mL) and $NaBH_4$ (0.51 g, 13.39 mmol) was added carefully in small portions. The resulting mixture was stirred for 3 h then cooled to 0 ºC. 20 mL of ice-cold water was added ant the resulting mixture was acidified with 1M HCl solution. 3-Nitrophenyl thiol (quant.) was extracted to dichloromethane, washed with water and brine, dried over anhydrous Na_2SO_4, filtered and concentrated *in vacuo*.

β-D-Galactopyranose pentaacetate (**13**, 0.88 g, 2.26 mmol) and freshly prepared 3-nitrophenyl thiol (189 mg, 1.22 mmol) were dissolved in dry dichloromethane (5 mL). Reaction mixture was cooled to 0 ºC and $BF_3 \cdot OEt_2$ (1 mL, 7.96 mmol) was added dropwise. Mixture was allowed to warm to room temperature and stirred overnight. The reaction was poured over ice cold saturated $NaHCO_3$ solution and diluted with dichloromethane. Organic phase was washed with saturated $NaHCO_3$ solution and brine, dried over anhydrous Na_2SO_4, filtered and concentrated *in vacuo*. Purification by normal phase MPLC (toluene/ethyl acetate, 5–20% ethyl acetate) gave compound **S1*m*** (358 mg, 0.74 mmol, 60%). Synthesis of compound **S1*m*** was first reported by Ramos-Soriano *et. al.*.[4]

^{1}H NMR (300 MHz, $CDCl_3$) δ 8.43 (t, *J* = 2.0 Hz, 1H, ArH), 8.15 (ddt, *J* = 8.3, 1.9, 0.9 Hz, 1H, ArH), 7.78 (ddt, *J* = 7.9, 1.8, 0.9 Hz, 1H, ArH), 7.49 (t, *J* = 8.0 Hz, 1H, ArH), 5.45 (dd, *J* = 3.3, 1.0 Hz, 1H, H-4), 5.22 (t, *J* = 9.9 Hz, 1H, H-2), 5.07 (dd, *J* = 10.0, 3.3 Hz, 1H, H-3), 4.78 (d, *J* = 9.8 Hz, 1H, H-1), 4.25 – 4.11 (m, 2H, H-6), 4.05 – 3.97 (m, 1H, H-5), 2.11 (s, 3H, CH_3), 2.09 (s, 3H, CH_3), 2.06 (s, 3H, CH_3), 1.96 (s, 3H, CH_3).

^{13}C NMR (75 MHz, $CDCl_3$) δ 170.58 (1C, C=O), 170.19 (1C, C=O), 170.05 (1C, C=O), 169.46 (1C, C=O), 148.50 (1C, ArC), 137.84 (1C, ArCH), 135.10 (1C, ArC), 129.61 (1C, ArCH), 126.56 (1C, ArCH), 122.90 (1C, ArCH), 85.52 (1C, C-1), 75.01 (1C, C-5), 71.89 (1C, C-3), 67.31 (1C, C-4), 66.89 (1C, C-2), 61.93 (1C, C-6), 20.89 (1C, CH_3), 20.77 (1C, CH_3), 20.67 (2C, CH_3).

HPLC-MS: $[C_{20}H_{23}NO_{11}S + NH_4]^+$ calcd. 503.13, found 503.06.

***p*-Nitrophenyl-thio-β-D-galactopyranose tetraacetate (S1*p*)**

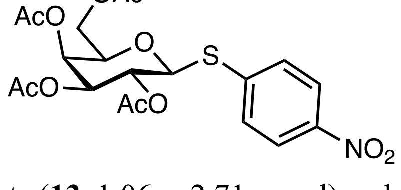

β-D-Galactopyranose pentaacetate (**13**, 1.06 g, 2.71 mmol) and 4-nitrophenyl thiol (1.20 g, 7.75 mmol) were dissolved in dry dichloromethane (10 mL). Reaction mixture was cooled to 0 ºC and $BF_3 \cdot OEt_2$ (1.6 mL, 12.74 mmol) was added dropwise. Mixture was allowed to warm to room temperature and stirred for 8 h. The reaction was poured over ice cold saturated $NaHCO_3$ solution and diluted with dichloromethane. Organic phase was washed with saturated $NaHCO_3$ solution and brine, dried over anhydrous Na_2SO_4, filtered and concentrated *in vacuo*. Purification by normal phase MPLC (toluene/ethyl acetate, 5–20% ethyl acetate) gave

compound **S1*p*** (0.61 g, 1.26 mmol, 47%). The analytical data of **S1*p*** are in agreement with literature.[5]

^{1}H NMR (300 MHz, $CDCl_3$) δ 8.20 – 8.12 (m, 2H, ArH), 7.65 – 7.57 (m, 2H, ArH), 5.47 (dd, *J* = 3.4, 1.0 Hz, 1H, H-4), 5.29 (t, *J* = 9.9 Hz, 1H, H-2), 5.10 (dd, *J* = 9.9, 3.3 Hz, 1H, H-3), 4.86 (d, *J* = 9.9 Hz, 1H, H-1), 4.26 – 4.09 (m, 2H, H-6), 4.08 – 4.00 (m, 1H, H-5), 2.16 (s, 3H, CH_3), 2.08 (s, 3H, CH_3), 2.07 (s, 3H, CH_3), 1.98 (s, 3H, CH_3).

^{13}C NMR (126 MHz, $CDCl_3$) δ 170.46 (1C, C=O), 170.16 (1C, C=O), 170.10 (1C, C=O), 169.51 (1C, C=O), 146.97 (1C, ArC), 142.54 (1C, ArC), 130.54 (2C, ArCH), 123.99 (2C, ArCH), 84.98 (1C, C-1), 74.98 (1C, C-5), 71.86 (1C, C-3), 67.20 (1C, C-4), 66.85 (1C, C-2), 61.81 (1C, C-6), 20.88 (1C, CH_3), 20.84 (1C, CH_3), 20.79 (1C, CH_3), 20.68 (1C, CH_3).

HPLC-MS: $[C_{20}H_{23}NO_{11}S + NH_4]^+$ calcd. 503.13, found 503.06.

***m*-Aminophenyl-thio-β-D-galactopyranose tetraacetate (S2*m*)**

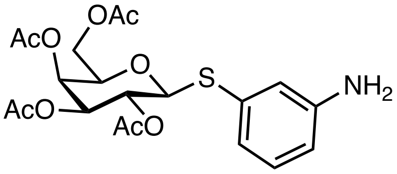

Compound **S1*m*** (1.97 g, 4.06 mmol) was dissolved in dry dichloromethane (40 mL) and 10% Pd/C (100 mg, 0.09 mmol) was added. After three vacuum/H_2 cycles the reaction was stirred under H_2 atmosphere (1 atm) for 2 d. The reaction was filtered over celite and concentrated *in vacuo*. Pure product **S2*m*** was obtained (1.84 g, 4.04 mmol, quant.) without further purification.

^{1}H NMR (300 MHz, DMSO-d_6) δ 6.98 (t, *J* = 7.8 Hz, 1H, ArH), 6.64 (t, *J* = 1.9 Hz, 1H, ArH), 6.59 (ddd, *J* = 7.6, 1.8, 1.0 Hz, 1H, ArH), 6.48 (ddd, *J* = 8.0, 2.2, 0.9 Hz, 1H, ArH), 5.31 (dd, *J* = 3.6, 1.1 Hz, 1H, H-4), 5.26 (dd, *J* = 9.5, 3.5 Hz, 1H, H-3), 5.19 (s, 2H, NH_2), 5.15 – 4.97 (m, 2H, H-1, H-2), 4.29 (t, *J* = 6.5 Hz, 1H, H-5), 4.13 – 3.99 (m, 2H, H-6), 2.13 (s, 3H, CH_3), 2.04 (s, 3H, CH_3), 2.00 (s, 3H, CH_3), 1.92 (s, 3H, CH_3).

^{13}C NMR (75 MHz, DMSO-d_6) δ 169.97 (1C, C=O), 169.87(1C, C=O), 169.43 (1C, C=O), 169.21 (1C, C=O), 149.15 (1C, ArC), 133.12 (1C, ArC), 129.44 (1C, ArCH), 117.59 (1C, ArCH), 115.67 (1C, ArCH), 113.13 (1C, ArCH), 84.80 (1C, C-1), 73.33 (1C, C-5), 71.05 (1C, C-3), 67.51 (1C, C-4), 67.18 (1C, C-2), 61.56 (1C, C-6), 20.54 (1C, CH_3), 20.47 (1C, CH_3), 20.41 (1C, CH_3), 20.34 (1C, CH_3).

HPLC-MS: $[C_{20}H_{25}NO_9S + H]^+$ calcd. 456.13, found 456.03.

HRMS: $[C_{20}H_{25}NO_9S + H]^+$ calcd. 456.1323, found 456.1315.

***p*-Aminophenyl-thio-β-D-galactopyranoside tetraacetate (S2*p*)**

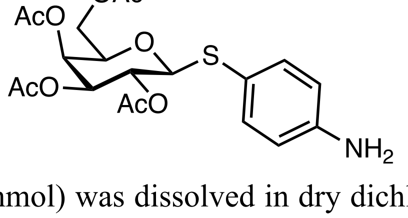

Compound **S1*p*** (0.66 g, 1.36 mmol) was dissolved in dry dichloromethane (20 mL) and 10% Pd/C (75 mg, 0.07 mmol) was added. After three vacuum/H_2 cycles the reaction was stirred under H_2 atmosphere (1 atm) overnight. The reaction was filtered over celite and concentrated *in vacuo*. Pure product **S2*p*** was obtained (0.62 g, 1.36 mmol, quant.) without further purification. The analytical data of **S2*p*** are in agreement with literature.[6]

^{1}H NMR (300 MHz, DMSO-d$_6$) δ 7.18 – 7.09 (m, 2H, ArH), 6.57 – 6.48 (m, 2H, ArH), 5.39 (s, 2H, NH$_2$), 5.25 (dd, *J* = 3.5, 1.0 Hz, 1H, H-4), 5.18 (dd, *J* = 9.7, 3.5 Hz, 1H, H-3), 4.93 (t, *J* = 9.9 Hz, 1H, H-2), 4.78 (d, *J* = 10.0 Hz, 1H, H-1), 4.21 – 4.13 (m, 1H, H-5), 4.12 – 3.94 (m, 2H, H-6), 2.09 (s, 3H, CH$_3$), 2.06 (s, 3H, CH$_3$), 2.00 (s, 3H, CH$_3$), 1.90 (s, 3H, CH$_3$).
^{13}C NMR (75 MHz, DMSO-d$_6$) δ 169.93 (1C, C=O), 169.84 (1C, C=O), 169.46 (1C, C=O), 169.17 (1C, C=O), 149.42 (1C, ArC), 135.15 (2C, ArCH), 115.20 (1C, ArC), 114.07 (2C, ArCH), 86.07 (1C, C-1), 73.25 (1C, C-5), 71.20 (1C, C-3), 67.62 (1C, C-4), 67.24 (1C, C-2), 61.66 (1C, C-6), 20.63 (1C, CH$_3$), 20.51 (1C, CH$_3$), 20.40 (1C, CH$_3$), 20.36 (1C, CH$_3$).
HPLC-MS: $[C_{20}H_{25}NO_9S + H]^+$ calcd. 456.13, found 456.04.

Galactoside S3*m*

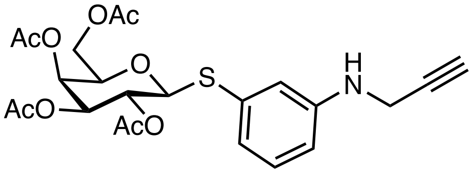

Compound **S2*m*** (308.7 mg, 0.68 mmol) was dissolved in dry dimethylformamide (3 mL) and potassium carbonate (184 mg, 1.33 mmol) was added. The reaction mixture was cooled to 0 ºC and propargyl bromide (100 µL, 1.02 mmol) was added dropwise. The mixture was allowed to warm to room temperature and then heated to 45 ºC for 6.5 h. The reaction was cooled to room temperature, poured over cold water and diluted with dichloromethane. Organic phase was washed with water and brine, dried over anhydrous Na_2SO_4, filtered and concentrated *in vacuo*. Purification by normal phase MPLC (toluene/ethyl acetate + 0.25% Et_3N, 5–30% ethyl acetate) gave compound **S3*m*** (188.3 mg, 0.38 mmol, 56%).
^{1}H NMR (500 MHz, DMSO-d$_6$) δ 7.12 – 7.05 (m, 1H, ArH), 6.71 – 6.65 (m, 2H, ArH), 6.60 – 6.55 (m, 1H, ArH), 6.15 (t, *J* = 6.1 Hz, 1H, NH), 5.32 (dd, *J* = 3.6, 1.2 Hz, 1H, H-4), 5.26 (dd, *J* = 9.8, 3.5 Hz, 1H, H-3), 5.18 (d, *J* = 10.1 Hz, 1H, H-1), 5.02 (t, *J* = 10.0 Hz, 1H, H-2), 4.34 – 4.28 (m, 1H, H-5), 4.11 – 4.01 (m, 2H, H-6), 3.86 (dd, *J* = 6.2, 2.4 Hz, 2H, CH$_2$), 3.08 (t, *J* = 2.4 Hz, 1H, C≡CH), 2.12 (s, 3H, CH$_3$), 2.05 (s, 3H, CH$_3$), 1.99 (s, 3H, CH$_3$), 1.92 (s, 3H, CH$_3$).
^{13}C NMR (126 MHz, DMSO-d$_6$) δ 170.02 (1C, C=O), 169.92 (1C, C=O), 169.50 (1C, C=O), 169.30 (1C, C=O), 148.25 (1C, ArC), 133.45 (1C, ArC), 129.41 (1C, ArCH), 118.27 (1C, ArCH), 114.11 (1C, ArCH), 112.06 (1C, ArCH), 84.65 (1C, C-1), 81.92 (1C, $\underline{C}$≡CH), 73.38 (1C, C-5), 73.15 (1C, C≡$\underline{C}$H), 71.06 (1C, C-3), 67.53 (1C, C-4), 67.11 (1C, C-2), 61.60 (1C, C-6), 31.96 (1C, CH$_2$), 20.59 (1C, CH$_3$), 20.50 (1C, CH$_3$), 20.44 (1C, CH$_3$), 20.38 (1C, CH$_3$).
HPLC-MS: $[C_{23}H_{27}NO_9S + H]^+$ calcd. 494.15, found 494.18.
HRMS: $[C_{23}H_{27}NO_9S + H]^+$ calcd. 494.1479, found 494.1471.

Galactoside S3*p*

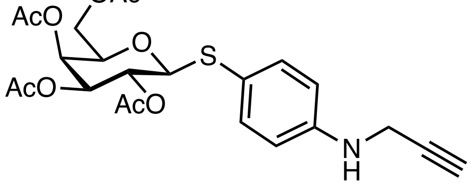

Compound **S2*p*** (105.3 mg, 0.23 mmol) was dissolved in dry dimethylformamide (1 mL) and potassium carbonate (64.3 mg, 0.46 mmol) was added. The reaction mixture was cooled to 0 ºC

and propargyl bromide (29 µL, 0.30 mmol) was added dropwise. The mixture was allowed to warm to room temperature and then heated to 40 ºC. After 20 h, dry dimethylformamide (1 mL), potassium carbonate (81.0 mg, 0.59 mmol) and propargyl bromide (25 µL, 0.26 mmol at 0 ºC) were added and stirred for 6 h at 40 ºC. The reaction was cooled to room temperature, poured over cold water and diluted with dichloromethane. Organic phase was washed with water, dried over anhydrous Na_2SO_4, filtered and concentrated *in vacuo*. Purification by normal phase MPLC (toluene/ethyl acetate + 0.25% Et_3N, 5–30% ethyl acetate) gave compound **S3*p*** (85.8 mg, 0.17 mmol, 75%).

^{1}H NMR (300 MHz, DMSO-d$_6$) δ 7.24 (d, *J* = 8.4 Hz, 2H, ArH), 6.66 – 6.57 (m, 2H, ArH), 6.30 (t, *J* = 6.1 Hz, 1H, NH), 5.26 (d, *J* = 3.5 Hz, 1H, H-4), 5.19 (dd, *J* = 9.6, 3.5 Hz, 1H, H-3), 4.95 (t, *J* = 9.8 Hz, 1H, H-2), 4.84 (d, *J* = 9.9 Hz, 1H, H-1), 4.19 (t, *J* = 6.3 Hz, 1H, H-5), 4.13 – 3.94 (m, 2H, H-6), 3.87 (dd, *J* = 6.2, 2.4 Hz, 2H, CH_2), 3.07 (t, *J* = 2.2 Hz, 1H, C≡CH), 2.09 (s, 3H, CH_3), 2.07 (s, 3H, CH_3), 2.00 (s, 3H, CH_3), 1.90 (s, 3H, CH_3).

^{13}C NMR (75 MHz, DMSO-d$_6$) δ 169.92 (1C, C=O), 169.84 (1C, C=O), 169.44 (1C, C=O), 169.18 (1C, C=O), 148.27 (1C, ArC), 134.84 (2C, ArCH), 116.56 (1C, ArC), 112.93 (2C, ArCH), 85.84 (1C, C-1), 81.87 (1C, <u>C</u>≡CH), 73.25 (1C, C-5), 73.05 (1C, C≡<u>C</u>H), 71.17 (1C, C-3), 67.59 (1C, C-4), 67.25 (1C, C-2), 61.66 (1C, C-6), 31.84 (1C, CH_2), 20.62 (1C, CH_3), 20.53 (1C, CH_3), 20.41 (1C, CH_3), 20.35 (1C, CH_3).

HPLC-MS: $[C_{23}H_{27}NO_9S + H]^+$ calcd. 494.15, found 494.13.

HRMS: $[C_{23}H_{27}NO_9S + H]^+$ calcd. 494.1479 , found 494.1475.

Galactoside S4*m*

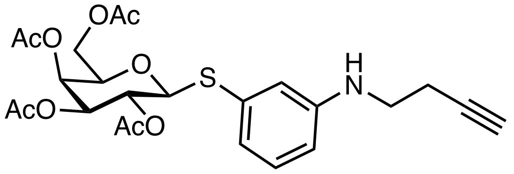

Compound **S2*m*** (337.1 mg, 0.74 mmol) was dissolved in dry dimethylformamide (3 mL) and potassium carbonate (175 mg, 1.26 mmol) was added. The reaction mixture was cooled to 0 ºC and 4-bromo-1-butyne (205 µL, 2.12 mmol) was added dropwise. The mixture was allowed to warm to room temperature and then heated to 70 ºC. After 48 h, dry dimethylformamide (1 mL), potassium carbonate (120 mg, 0.87 mmol) and 4-bromo-1-butyne (100 µL, 1.07 mmol at 0 ºC) were added and stirred for additional 48 h at 70 ºC. The reaction was cooled to room temperature, poured over cold water and diluted with dichloromethane. Organic phase was washed with water, dried over anhydrous Na_2SO_4, filtered and concentrated *in vacuo*. Purification by normal phase MPLC (toluene/ethyl acetate + 0.25% Et_3N, 5–30% ethyl acetate) gave compound **S4*m*** (219.4 mg, 0.43 mmol, 58%).

^{1}H NMR (500 MHz, DMSO-d$_6$) δ 7.087 – 7.01 (m, 1H, ArH), 6.64 – 6.60 (m, 2H, ArH), 6.53 – 6.50 (m, 1H, ArH), 5.88 (t, *J* = 6.0 Hz, 1H, NH), 5.31 (dd, *J* = 3.5, 1.1 Hz, 1H, H-4), 5.27 (dd, *J* = 9.8, 3.6 Hz, 1H, H-3), 5.18 (d, *J* = 10.1 Hz, 1H, H-1), 5.02 (t, *J* = 10.0 Hz, 1H, H-2), 4.31 (td, *J* = 6.8, 6.4, 1.2 Hz, 1H, H-5), 4.10 – 4.01 (m, 2H, H-6), 3.19 (q, *J* = 6.9 Hz, 2H, NHC<u>H</u>$_2$CH$_2$), 2.85 (t, *J* = 2.7 Hz, 1H, C≡CH), 2.39 (td, *J* = 7.1, 2.7 Hz, 2H, NHCH$_2$C<u>H</u>$_2$), 2.12 (s, 3H, CH_3), 2.04 (s, 3H, CH_3), 1.99 (s, 3H, CH_3), 1.92 (s, 3H, CH_3).

^{13}C NMR (126 MHz, DMSO-d$_6$) δ 169.97 (1C, C=O), 169.86 (1C, C=O), 169.45 (1C, C=O), 169.22 (1C, C=O), 148.67 (1C, ArC), 133.42 (1C, ArC), 129.50 (1C, ArCH), 117.71 (1C,

ArCH), 113.26 (1C, ArCH), 111.54 (1C, ArCH), 84.47 (1C, C-1), 82.62 (1C, $\underline{C}\equiv CH$), 73.35 (1C, C-5), 72.21 (1C, $C\equiv\underline{C}H$), 71.04 (1C, C-3), 67.55 (1C, C-4), 67.08 (1C, C-2), 61.62 (1C, C-6), 41.76 (1C, $NH\underline{C}H_2CH_2$), 20.56 (1C, CH_3), 20.49 (1C, CH_3), 20.42 (1C, CH_3), 20.35 (1C, CH_3), 18.35 (1C, $NHCH_2\underline{C}H_2$).
HPLC-MS: $[C_{24}H_{29}NO_9S + H]^+$ calcd. 508.16, found 508.22.

Galactoside S4*p*

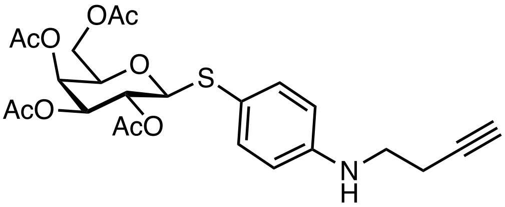

Compound **S2*p*** (315.8 mg, 0.69 mmol) was dissolved in dry dimethylformamide (3 mL) and potassium carbonate (280 mg, 2.03 mmol) was added. The reaction mixture was cooled to 0 ºC and 4-bromo-1-butyne (280 µL, 2.98 mmol) was added dropwise. The mixture was allowed to warm to room temperature and then heated to 65 ºC. After 48 h, dry dimethylformamide (1 mL), potassium carbonate (88.7 mg, 0.64 mmol) and 4-bromo-1-butyne (100 µL, 1.07 mmol at 0 ºC) were added and stirred for additional 48 h at 65 ºC. The reaction was cooled to room temperature, poured over cold water and diluted with dichloromethane. Organic phase was washed with water, dried over anhydrous Na_2SO_4, filtered and concentrated *in vacuo*. Purification by normal phase MPLC (toluene/ethyl acetate + 0.25% Et_3N, 5–30% ethyl acetate) gave compound **S4*p*** (259.3 mg, 0.51 mmol, 74%).
^{1}H NMR (300 MHz, DMSO-d$_6$) δ 7.25 – 7.14 (m, 2H, ArH), 6.60 – 6.50 (m, 2H, ArH), 6.05 (t, *J* = 6.0 Hz, 1H, NH), 5.26 (dd, *J* = 3.5, 1.0 Hz, 1H, H-4), 5.19 (dd, *J* = 9.7, 3.5 Hz, 1H, H-3), 4.94 (t, *J* = 9.8 Hz, 1H, H-2), 4.80 (d, *J* = 10.0 Hz, 1H, H-1), 4.21 – 4.12 (m, 1H, H-5), 4.12 – 3.92 (m, 2H, H-6), 3.19 (q, *J* = 6.8 Hz, 2H, $NHC\underline{H}_2CH_2$), 2.85 (t, *J* = 2.6 Hz, 1H, $C\equiv CH$), 2.38 (td, *J* = 7.0, 2.7 Hz, 2H, $NHCH_2C\underline{H}_2$), 2.08 (s, 3H, CH_3), 2.06 (s, 3H, CH_3), 2.00 (s, 3H, CH_3), 1.90 (s, 3H, CH_3).
^{13}C NMR (126 MHz, DMSO-d$_6$) δ 169.97 (1C, C=O), 169.90 (1C, C=O), 169.51 (1C, C=O), 169.23 (1C, C=O), 148.84 (1C, ArC), 135.28 (2C, ArCH), 115.54 (1C, ArC), 112.26 (2C, ArCH), 86.04 (1C, C-1), 82.64 (1C, $\underline{C}\equiv CH$), 73.25 (1C, C-5), 72.29 (1C, $C\equiv\underline{C}H$), 71.19 (1C, C-3), 67.60 (1C, C-4), 67.24 (1C, C-2), 61.67 (1C, C-6), 41.69 (1C, $NH\underline{C}H_2CH_2$), 20.67 (1C, CH_3), 20.56 (1C, CH_3), 20.44 (1C, CH_3), 20.40 (1C, CH_3), 18.37 (1C, $NHCH_2\underline{C}H_2$).
HPLC-MS: $[C_{24}H_{29}NO_9S + H]^+$ calcd. 508.16, found 508.18.
HRMS: $C_{24}H_{29}NO_9S + H]^+$ calcd. 508.1636, found 508.1633.

Galactoside precursor S5*m*

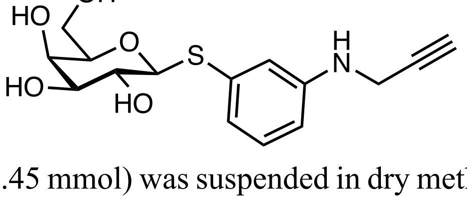

Compound **S3*m*** (224.5 mg, 0.45 mmol) was suspended in dry methanol (4 mL) and 1M sodium methoxide in methanol (cat.) was added and stirred overnight. The reaction mixture was neutralized with 1M HCl solution and the solvent was removed *in vacuo*. Purification by normal

phase MPLC (dichloromethane/ethanol + 1% NH_4OH, 1–20% ethanol) gave compound **S5*m*** (79.7 mg, 0.24 mmol, 54%).

^{1}H NMR (500 MHz, MeOH-d$_4$) δ 7.05 (t, *J* = 7.9 Hz, 1H, ArH), 6.94 (t, *J* = 2.0 Hz, 1H, ArH), 6.86 – 6.81 (m, 1H, ArH), 6.58 (dd, *J* = 8.0, 2.1 Hz, 1H, ArH), 4.60 (d, *J* = 9.8 Hz, 1H, H-1), 3.92 – 3.88 (m, 3H, H-4, CH_2), 3.82 – 3.69 (m, 2H, H-6), 3.65 – 3.57 (m, 2H, H-2, H-5), 3.50 (dd, *J* = 9.2, 3.3 Hz, 1H, H-3), 2.51 (t, *J* = 2.4 Hz, 1H, C≡CH).

^{13}C NMR (126 MHz, MeOH-d$_4$) δ 149.63 (1C, ArC), 136.71 (1C, ArC), 130.19 (1C, ArCH), 120.81 (1C, ArCH), 116.03 (1C, ArCH), 113.46 (1C, ArCH), 90.37 (1C, C-1), 82.40 (1C, <u>C</u>≡CH), 80.58 (1C, C-5), 76.37 (1C, C-3), 71.93 (1C, C≡<u>C</u>H), 70.96 (1C, C-2), 70.44 (1C, C-4), 62.65 (1C, C-6), 33.73 (1C, CH_2).

HPLC-MS: $[C_{15}H_{19}NO_5S + H]^+$ calcd. 326.11, found 326.20.

HRMS: $[C_{15}H_{19}NO_5S + H]$+ calcd. 326.1057, found 326.1056.

Galactoside precursor S5*p*

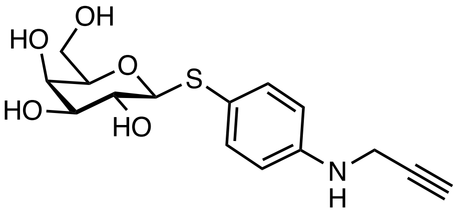

Compound **S3*p*** (148.1 mg, 0.30 mmol) was suspended in dry methanol (9 mL) and 1M sodium methoxide in methanol (cat.) was added. After 4 h, the reaction mixture was neutralized with 1M HCl solution. The solvent was removed *in vacuo*. Purification by C18 column HPLC chromatography (water/acetonitrile + 0.1% formic acid, 5–40% acetonitrile) gave compound **S5*p*** (63.5 mg, 0.20 mmol, 65%).

^{1}H NMR (500 MHz, MeOH-d$_4$) δ 7.43 – 7.37 (m, 2H, ArH), 6.68 – 6.61 (m, 2H, ArH), 4.32 (d, *J* = 9.5 Hz, 1H, H-1), 3.88 (d, *J* = 2.4 Hz, 2H, CH_2), 3.86 (d, *J* = 3.1 Hz, 1H, H-4), 3.76 – 3.67 (m, 2H, H-6), 3.54 – 3.43 (m, 3H, H-2, H-3, H-5), 2.50 (t, *J* = 2.4 Hz, 1H, C≡CH).

^{13}C NMR (126 MHz, MeOH-d$_4$) δ 149.45 (1C, ArC), 136.20 (2C, ArCH), 120.79 (1C, ArC), 114.59 (2C, ArCH), 91.65 (1C, C-1), 82.21 (1C, <u>C</u>≡CH), 80.46 (1C, C-5), 76.37 (1C, C-3), 71.86 (1C, C≡<u>C</u>H), 70.97 (1C, C-2), 70.42 (1C, C-4), 62.54 (1C, C-6), 33.56 (1C, CH_2).

HPLC-MS: $[C_{15}H_{19}NO_5S + H]^+$ calcd. 326.11, found 326.21.

HRMS: $[C_{15}H_{19}NO_5S + Na]$+ calcd. 348.0876, found 348.0875.

Galactoside precursor S6*m*

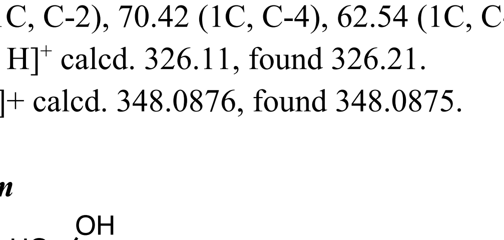

Compound **S4*m*** (214.1 mg, 0.42 mmol) was suspended in dry methanol (4 mL) and 1M sodium methoxide in methanol (cat.) was added and stirred overnight. The reaction mixture was neutralized with 1M HCl solution and the solvent was removed *in vacuo*. Purification by normal phase MPLC (dichloromethane/ethanol + 1% NH_4OH, 1–20% ethanol) gave compound **S6*m*** (81.4 mg, 0.24 mmol, 57%).

^{1}H NMR (500 MHz, MeOH-d$_4$) δ 7.03 (t, *J* = 7.9 Hz, 1H, ArH), 6.87 (t, *J* = 1.9 Hz, 1H), 6.81 – 6.78 (m, 1H, ArH), 6.52 (dd, *J* = 8.0, 1.9 Hz, 1H, ArH), 4.58 (d, *J* = 9.7 Hz, 1H, H-1), 3.90 (d, *J* = 3.1 Hz, 1H, H-4), 3.82 – 3.68 (m, 2H, H-6), 3.64 – 3.55 (m, 2H, H-2, H-5), 3.50 (dd, *J* = 9.2, 3.3 Hz, 1H, H-3), 3.28 (t, *J* = 7.0 Hz, 2H, NHC$\underline{H}_2$CH$_2$), 2.44 (td, *J* = 7.0, 2.6 Hz, 2H, NHCH$_2$C$\underline{H}_2$), 2.30 (t, *J* = 2.6 Hz, 1H, C≡CH).
^{13}C NMR (126 MHz, MeOH-d$_4$) δ 150.01 (1C, ArC), 136.63 (1C, ArC), 130.38 (1C, ArCH), 120.43 (1C, ArCH), 115.72 (1C, ArCH), 113.01 (1C, ArCH), 90.37 (1C, C-1), 82.82 (1C, $\underline{C}$≡CH), 80.59 (1C, C-5), 76.36 (1C, C-3), 70.96 (1C, C≡$\underline{C}$H), 70.78 (1C, C-2), 70.44 (1C, C-4), 62.67 (1C, C-6), 43.67 (1C, NH$\underline{C}$H$_2$CH$_2$), 19.62 (1C, NHCH$_2\underline{C}$H$_2$).
HPLC-MS: [$C_{16}H_{21}NO_5S$ + H]$^+$ calcd. 340.12, found 340.22.
HRMS: [$C_{16}H_{21}NO_5S$ + H]+ calcd. 340.1213, found 340.1215.

Galactoside precursor S6*p*

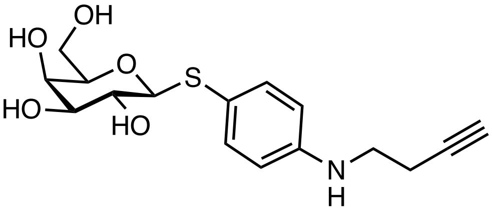

Compound **S4*p*** (232.4 mg, 0.46 mmol) was suspended in dry methanol (5 mL) and 1M sodium methoxide in methanol (cat.) was added and stirred overnight. The reaction mixture was neutralized with 1M HCl solution and the solvent was removed *in vacuo*. Purification by normal phase MPLC (dichloromethane/ethanol + 1% NH_4OH, 1–20% ethanol) gave compound **S6*p*** (118.2 mg, 0.36 mmol, 79%).
^{1}H NMR (500 MHz, MeOH-d$_4$) δ 7.41 – 7.35 (m, 2H, ArH), 6.60 – 6.53 (m, 2H, ArH), 4.30 (d, *J* = 9.5 Hz, 1H, H-1), 3.86 (dd, *J* = 3.3, 1.1 Hz, 1H, H-4), 3.77 – 3.65 (m, 2H, H-6), 3.54 – 3.43 (m, 3H, H-2, H-3, H-5), 3.27 (t, *J* = 7.1 Hz, 2H, NHC$\underline{H}_2$CH$_2$), 2.43 (td, *J* = 7.1, 2.7 Hz, 2H, NHCH$_2$C$\underline{H}_2$), 2.30 (t, *J* = 2.7 Hz, 1H, C≡CH).
^{13}C NMR (126 MHz, MeOH-d$_4$) δ 149.88 (1C, ArC), 136.58 (2C, ArCH), 119.77 (1C, ArC), 113.94 (2C, ArCH), 91.64 (1C, C-1), 82.71 (1C, $\underline{C}$≡CH), 80.45 (1C, C-5), 76.37 (1C, C-3), 70.93 (1C, C-2), 70.76 (1C, C≡$\underline{C}$H), 70.42 (1C, C-4), 62.54 (1C, C-6), 43.57 (1C, NH$\underline{C}$H$_2$CH$_2$), 19.61 (1C, NHCH$_2\underline{C}$H$_2$).
HPLC-MS: [$C_{16}H_{21}NO_5S$ + H]$^+$ calcd. 340.12, found 340.21.
HRMS: [$C_{16}H_{21}NO_5S$ + H]+ calcd. 340.1213, found 340.1215.

Bis(4-prop-2-ynyloxyphenyl) disulphide (S8)

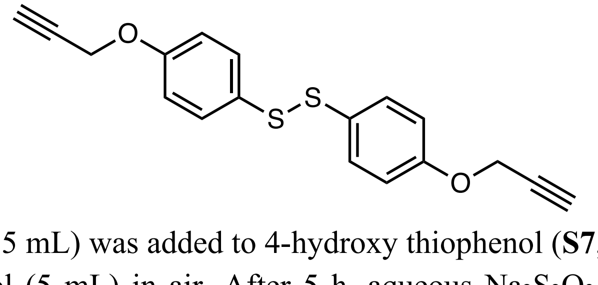

Iodine solution (satd., 0.5 mL) was added to 4-hydroxy thiophenol (**S7**, 187.4 mg, 1.49 mmol) and dissolved in ethanol (5 mL) in air. After 5 h, aqueous $Na_2S_2O_3$ solution was added to remove the residual iodine. Product was extracted to ethyl acetate, organic phase was washed with aqueous $Na_2S_2O_3$ solution and half satd. brine, dried over anhydrous Na_2SO_4, filtered and

concentrated *in vacuo*. Crude bis(4-hydroxyphenyl) disulfide was purified by crystallization from chloroform (93.6 mg, 0.37 mmol, 50%). The analytical data matched the literature.[7]
Bis(4-hydroxyphenyl) disulfide (80.4 mg, 0.32 mmol) was dissolved in dry dimethylformamide (3 mL) and potassium carbonate (227.5 mg, 1.65 mmol) was added. Propargyl bromide (185 µL, 1.89 mmol) was added dropwise and stirred overnight. The reaction was poured over cold water and diluted with dichloromethane. Organic phase was washed half satd. brine, dried over anhydrous Na_2SO_4, filtered and concentrated *in vacuo*. Purification by normal phase MPLC (petrol ether/ethyl acetate, 5–20% ethyl acetate) gave compound **S8** (87.3 mg, 0.27 mmol, 83%). The analytical data match the literature.[8]
^{1}H NMR (500 MHz, $CDCl_3$) δ 7.44 – 7.39 (m, 4H, ArH), 6.94 – 6.89 (m, 4H, ArH), 4.68 (d, *J* = 2.4 Hz, 4H, CH_2), 2.53 (t, *J* = 2.4 Hz, 2H, C≡CH).
^{13}C NMR (126 MHz, $CDCl_3$) δ 157.85 (2C, ArC), 132.15 (4C, ArCH), 129.56 (2C, ArC), 115.72 (4C, ArCH), 78.30 (2C, $\underline{C}$≡CH), 75.99 (2C, C≡$\underline{C}$H), 56.04 (2C, CH_2).

4-prop-2-ynyloxyphenyl thiol (S9)

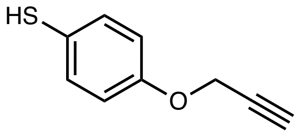

Disulfide **S8** (726 mg, 2.22 mmol) was dissolved in dry methanol (30 mL). 1,4-Dithio-threitol (1.72 g, 11.1 mmol) and DIPEA (1 mL, 5. 74 mmol) were added. The reaction was stirred for 3 h at r.t., then concentrated and diluted with EtOAc. The organic phase was washed with 1M HCl solution, half satd. brine, dried over anhydrous Na_2SO_4, filtered and concentrated *in vacuo*. Crude 4-prop-2-ynyloxyphenyl thiol (**S9**, quant., with ≈ 1 eq. of 1,2-dithiane-4,5-diol impurity) was used without further purification.
^{1}H NMR (500 MHz, MeOH-d_4) δ 7.26 – 7.21 (m, 2H, ArH), 6.90 – 6.86 (m, 2H, ArH), 4.69 (d, *J* = 2.4 Hz, 2H, CH_2), 3.49 (d, *J* = 8.7 Hz, 1H, impurity), 3.03 (d, *J* = 12.1 Hz, 1H, impurity), 2.93 (t, *J* = 2.3 Hz, 1H, C≡CH), 2.88 (dd, *J* = 12.9, 9.5 Hz, 1H, impurity).

Galactoside S10

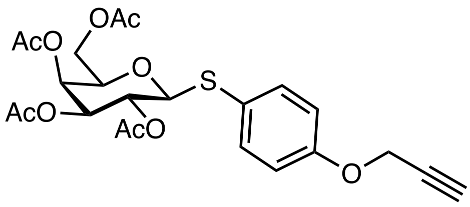

β-D-Galactopyranose pentaacetate (**13**, 1.49 g, 3.83 mmol) and 4-prop-2-ynyloxyphenyl thiol (**S9**, 365 mg, 2.22 mmol) were dissolved in mixture of dry dichloromethane (20 mL) and toluene (15 mL). Reaction mixture was cooled to 0 ºC and $BF_3 \cdot OEt_2$ (1 mL, 7.96 mmol) was added dropwise. Mixture was allowed to warm to room temperature and stirred overnight. The reaction was poured over ice cold satd. $NaHCO_3$ solution and diluted with dichloromethane. Organic phase was washed with satd. $NaHCO_3$ solution and brine, dried over anhydrous Na_2SO_4, filtered and concentrated *in vacuo*. Purification by normal phase MPLC (toluene/ethyl acetate, 5–25% ethyl acetate) gave compound **S10** (736 mg, 1.49 mmol, 39%).
^{1}H NMR (500 MHz, $CDCl_3$) δ 7.48 (d, *J* = 7.8 Hz, 2H, ArH), 6.93 (d, *J* = 7.9 Hz, 2H, ArH), 5.38 (s, 1H, H-4), 5.17 (t, *J* = 9.9 Hz, 1H, H-2), 5.05 – 4.98 (m, 1H, H-3), 4.69 (s, 2H, CH_2),

4.58 (d, J = 9.9 Hz, 1H, H-1), 4.21 – 4.05 (m, 2H, H-6), 3.89 (t, J = 6.5 Hz, 1H, H-5), 2.53 (s, 2H, C≡CH), 2.10 (s, 3H, CH_3), 2.09 (s, 3H, CH_3) 2.03 (s, 3H, CH_3), 1.96 (s, 3H, CH_3).
^{13}C NMR (126 MHz, $CDCl_3$) δ 170.52 (1C, C=O), 170.32 (1C, C=O), 170.22 (1C, C=O), 169.58 (1C, C=O), 158.23 (1C, ArC), 135.79 (2C, ArCH), 123.43 (1C, ArC), 115.38 (2C, ArCH), 87.01 (1C, C-1), 78.26 (1C, C≡CH), 76.00 (1C, C≡CH), 74.42 (1C, C-5), 72.15 (1C, C-3), 67.40 (1C, C-2), 67.30 (1C, C-4), 61.66 (1C, C-5), 55.93 (1C, CH_2), 21.03 (1C, CH_3), 20.83 (1C, CH_3), 20.78 (1C, CH_3), 20.73 (1C, CH_3).
HPLC-MS: $[C_{23}H_{26}O_{10}S + NH_4]^+$ calcd. 512.16, found 512.15.
HRMS: $[C_{23}H_{26}O_{10}S + Na]^+$ calcd. 517.1139, found 517.1134.

Galactoside precursor S11

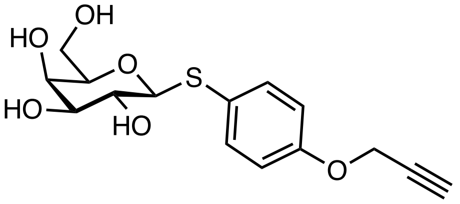

Compound **S10** (736 mg, 1.49 mmol) was dissolved in dry methanol (30 mL) and 1M sodium methoxide in methanol (cat.) was added. After 2h, the reaction mixture was neutralized with Amberlite IR 120/H$^+$. The solvent was removed *in vacuo*. Purification by normal phase MPLC (dichloromethane/methanol, 1–20% methanol) gave compound **S11** (485 mg, 1.49 mmol, quant.).
^{1}H NMR (500 MHz, MeOH-d$_4$) δ 7.54 (d, J = 7.6 Hz, 2H, ArH), 6.93 (d, J = 7.6 Hz, 2H, ArH), 4.72 (s, 2H, CH_2), 4.42 (d, J = 9.5 Hz, 1H, H-1), 3.88 (s, 1H, H-4), 3.80 – 3.66 (m, 2H, H-6), 3.58 – 3.45 (m, 3H, H-2, H-3, H-5), 2.95 (d, J = 2.1 Hz, 1H, C≡CH).
^{13}C NMR (126 MHz, MeOH-d$_4$) δ 159.05 (1C, ArC), 135.47 (2C, ArCH), 126.56 (1C, ArC), 116.37 (2C, ArCH), 91.05 (1C, C-1), 80.47 (1C, C-5), 79.61 (1C, C≡CH), 76.90 (1C, C≡CH), 76.31 (1C, C-3), 70.94 (1C, C-2), 70.46 (1C, C-4), 62.62 (1C, C-6), 56.63 (1C, CH_2).
HPLC-MS: $[C_{15}H_{18}O_6S + NH_4]^+$ calcd. 344.12, found 344.11.
HRMS: $[C_{15}H_{18}O_6S + Na]^+$ calcd. 349.0716, found 349.0714.

4-(Propargyloxy)benzenesulfonyl chloride (S13)

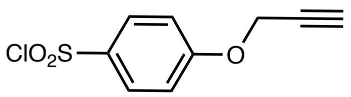

Propargyl phenyl ether (**S12**, 212.5 mg, 1.61 mmol**)** was dissolved in dichloromethane (8 mL) and cooled to 0 ºC. Chlorosulfonic acid (300 µL, 4.51 mmol) was added dropwise. The reaction was stirred for 2 h at 0 ºC and then poured over ice. The product was extracted to chloroform. Organic phase was washed with water and half satd. brine, dried over anhydrous Na_2SO_4, filtered and concentrated *in vacuo*. Crude compound **S13** (266.6 mg, 1.56 mmol, 72%) was used without further purification. Synthesis of compound **S13** was first reported by Inkster *et. al.*.[9]

C-glycoside precursor S16

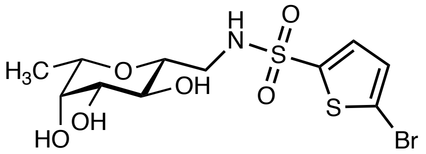

(1-Aminomethyl)-1-deoxy-β-L-fucopyranoside[1] (**S14**, 350.5 mg, 1.98 mmol) was dissolved in dry dimethylformamide (20 mL) and triethyl amine (350 µL, 2.51 mmol) was added. The reaction was cooled to 0 °C in an ice bath and 5-bromothiophene-2-sulfonyl chloride (481.2, 1.84 mmol) in dry dimethylformamide (15 mL) was added slowly. Mixture was allowed to warm to room temperature and stirred for 2 h. The reaction was concentrated *in vacuo*, diluted with ethyl acetate and satd. ammonium chloride solution. Phases were separated, aqueous phase was re-extracted with ethyl acetate. Combined organic phases were washed with half satd. brine, dried over anhydrous Na_2SO_4, filtered and concentrated *in vacuo*. Purification by normal phase MPLC (dichloromethane/methanol, 1–20% methanol) gave compound **S16** (635 mg, 1.58 mmol, 80%). The analytical data match the literature.[10]
^{1}H NMR (500 MHz, MeOH-d$_4$) δ 7.40 (d, J = 4.0 Hz, 1H, ArH), 7.19 (d, J = 4.0 Hz, 1H, ArH), 3.61 (d, J = 1.6 Hz, 1H, H-4), 3.54 – 3.48 (m, 1H, H-5), 3.41 – 3.37 (m, 3H, H-1, H-3, $CH_{2,a}$), 3.22 – 3.15 (m, 1H, H-2), 3.08 (dd, J = 12.9, 7.2 Hz, 1H, $CH_{2,b}$), 1.20 (d, J = 6.5 Hz, 3H, CH_3).
^{13}C NMR (126 MHz, MeOH-d$_4$) δ 144.15 (1C, ArC), 133.19 (1C, ArCH), 131.91 (1C, ArCH), 119.81 (1C, ArC), 79.48 (1C, C-2), 76.31 (1C, C-3), 75.54 (1C, C-5), 73.56 (1C, C-4), 69.67 (1C, C-1), 45.73 (1C, CH_2), 17.06 (1C, CH_3).
HPLC-MS: $[C_{11}H_{16}BrNO_6S_2 + H]^+$ calcd. 401.97, found 401.95.

C-glycoside 23

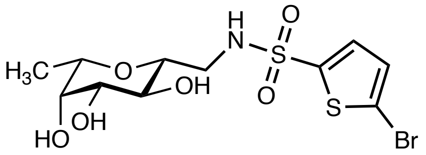

Compound **S15** (112.7 mg, 0.28 mmol) was dissolved in dry degassed dimethylformamide (3 mL). CuI (5.1 mg, 0.03 mmol), $PdCl_2(PPh_3)_2$ (8.9 mg, 0.01 mmol), triethyl amine (100 µL, 0.72 mmol) and trimethylsilyl acetylene (350 µL, 2.53 mmol) were added at r.t.. The reaction was stirred at 30 °C for 5 h, then cooled down to r.t. and diluted with ethyl acetate. Organic phase was washed with satd. ammonium chloride solution and brine, dried over anhydrous Na_2SO_4, filtered and concentrated *in vacuo*. Silyl protected intermediate was purified by normal phase MPLC (dichloromethane/ethanol, 1–15% ethanol), then dissolved in methanol (10 mL) and powder potassium carbonate (79.4 mg, 57 mmol) was added. After 1 h, the reaction was acidified with 1M HCl solution and concentrated *in vacuo*. The reaction mixture was diluted with ethyl acetate and satd. ammonium chloride solution. Phases were separated, the organic phase was washed with satd. ammonium chloride solution and brine, dried over anhydrous Na_2SO_4, filtered and concentrated *in vacuo*. Purification by reverse phase MPLC (water/acetonitrile with 1% formic acid, 10–20% acetonitrile) gave compound **23** (48.5 mg, 0.14 mmol, 50% over two steps).
^{1}H NMR (500 MHz, DMSO-d$_6$) δ 7.96 (s, 1H, NH), 7.49 (d, J = 3.9 Hz, 1H, ArH), 7.40 (d, J = 3.9 Hz, 1H, ArH), 4.83 (s, 2H, C≡CH, OH-2), 4.62 (s, 1H, OH-3), 4.28 (d, J = 3.1 Hz, 1H, OH-4), 3.42 – 3.38 (m, 2H, H-4, H-5), 3.29 (dd, J = 13.1, 2.0 Hz, 1H, $CH_{2,a}$), 3.22 (dd, J = 9.1,

2.8 Hz, 1H, H-3), 3.15 (t, *J* = 9.2 Hz, 1H, H-1), 3.03 (td, *J* = 8.9, 2.1 Hz, 1H, H-2), 2.81 (dd, *J* = 13.1, 8.4 Hz, 1H, $CH_{2,b}$), 1.06 (d, *J* = 6.4 Hz, 3H, CH_3).
^{13}C NMR (126 MHz, DMSO-d_6) δ 142.73 (1C, ArC), 133.48 (1C, ArCH), 131.17 (1C, ArCH), 126.30 (1C, ArC), 87.95 (1C, C≡$\underline{C}$H), 78.26 (1C, C-2), 75.40 (1C, $\underline{C}$≡CH), 74.65 (1C, C-3), 73.69 (1C, C-5), 71.60 (1C, C-4), 68.26 (1C, C-1), 44.80 (1C, CH_2), 16.93 (1C, CH_3).
HPLC-MS: $[C_{13}H_{17}NO_6S_2 + H]^+$ calcd. 348.06, found 348.06.
HRMS: $[C_{13}H_{17}NO_6S_2 + Na]^+$ calcd. 370.0389, found 370.0387.

C-glycoside precursor S16

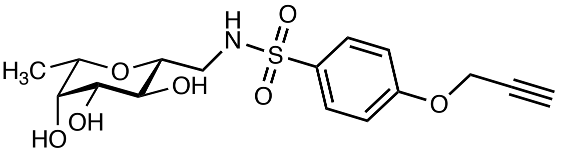

(1-Aminomethyl)-1-deoxy-β-L-fucopyranoside[1] (**S14,** 194.6 mg, 0.91 mmol) was dissolved in dry dimethylformamide (12 mL) and triethyl amine (190 µL, 1.36 mmol) was added. The reaction was cooled to 0 °C in an ice bath and compound **S13** (266.6 mg, 1.56 mmol) in dry dimethylformamide (10 mL) was added slowly. Mixture was allowed to warm to room temperature and stirred for 3 h. The reaction was concentrated *in vacuo*, diluted with ethyl acetate and satd. ammonium chloride solution. Phases were separated, aqueous phase was re-extracted with ethyl acetate. Combined organic phases were washed with half satd. brine, dried over anhydrous Na_2SO_4, filtered and concentrated *in vacuo*. Purification by reverse phase MPLC (water/acetonitrile supplemented with 1% formic acid, 10–20% acetonitrile) gave compound **S16** (137.7 mg, 0.37 mmol, 34%).
^1H NMR (500 MHz, MeOH-d_4) δ 7.85 – 7.78 (m, 2H, ArH), 7.17 – 7.10 (m, 2H, ArH), 4.84 (d, *J* = 2.4 Hz, 2H, $C\underline{H_2}$C≡CH), 3.61 – 3.57 (m, 1H, H-4), 3.47 – 3.42 (m, 1H, H-5), 3.41 – 3.34 (m, 2H, H-1, H-3), 3.32 – 3.26 (m, 1H, $C\underline{H}_{2,a}$NH), 3.11 (td, *J* = 9.0, 8.4, 2.5 Hz, 1H, H-2), 3.02 (t, *J* = 2.4 Hz, 1H, C≡CH), 2.98 (dd, *J* = 13.0, 7.3 Hz, 1H, $C\underline{H}_{2,b}$NH), 1.18 (d, *J* = 6.5 Hz, 3H,CH_3).
^{13}C NMR (126 MHz, MeOH-d_4) δ 162.17 (1C, ArC), 134.28 (1C, ArC), 130.10 (2C, ArCH), 116.18 (2C, ArCH), 79.52 (1C, C-2), 79.01 (1C, $\underline{C}$≡CH), 77.51 (1C, C≡$\underline{C}$H), 76.31 (1C, C-5), 75.51 (1C, C-3), 73.56 (1C, C-4), 69.75 (1C, C-1), 56.88 (1C, $\underline{C}H_2$C≡CH), 45.55 (1C, CH_2NH), 17.06 (1C, CH_3).
HPLC-MS: $[C_{16}H_{21}NO_7S + H]^+$ calcd. 372.11, found 372.11.
HRMS: $[C_{16}H_{21}NO_7S + H]^+$ calcd. 372.1111, found 372.1111.

β-D-Xylopyranose tetraacetate (S18)

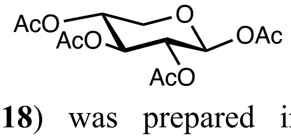

β-D-Xylopyranose tetraacetate (**S18**) was prepared in analogy to synthesis of β-D-galactopyranose pentaacetate (**13**) reported by Cohen *et al.*[11] D-xylose (**S17**, 1.20 g, 7.99 mmol), acetic anhydride (9.4 mL, 99.4 mmol) and anhydrous sodium acetate (0.85 g, 10.4 mmol) were heated to 100 °C for 20 minutes. The mixture was allowed to cooled down to room temperature, poured over 50 mL of ice water and stirred for 1 hour. The product was extracted with dichloromethane, washed with water, dried over anhydrous Na_2SO_4, filtered and evaporated to

a syrup. Crude product was purified by crystallisation from ethanol (1.00 g, 3.15 mmol, 39%). Compound **S18** was first reported by Hudson and Johnson[12] and the analytical data match the literature.[13]

^{1}H NMR (500 MHz, $CDCl_3$) δ 5.71 (d, *J* = 6.8 Hz, 1H, H-1), 5.20 (t, *J* = 8.3 Hz, 1H, H-3), 5.03 (dd, *J* = 8.4, 6.9 Hz, 1H, H-2), 4.97 (td, *J* = 8.3, 5.0 Hz, 1H, H-4), 4.14 (dd, *J* = 12.0, 5.0 Hz, 1H, Heq-5), 3.52 (dd, *J* = 12.0, 8.4 Hz, 1H, Hax-5), 2.10 (s, 3H, CH_3), 2.05 (s, 3H, CH_3), 2.05 (s, 6H, CH_3).

^{13}C NMR (126 MHz, $CDCl_3$) δ 169.96 (2C, C=O), 169.45 (1C, C=O), 169.18 (1C, C=O), 92.17 (1C, C-1), 71.13 (1C, C-3), 69.61 (1C, C-2), 68.44 (1C, C-4), 62.94 (1C, C-5), 20.95 (1C, CH_3), 20.86 (1C, CH_3), 20.80 (1C, CH_3), 20.74 (1C, CH_3).

HPLC-MS: $[C_{13}H_{18}O_9 + Na]^+$ calcd. 341.08, found 341.16.

***p*-Nitrophenyl-thio-β-D-xylopyranose triacetate (S19)**

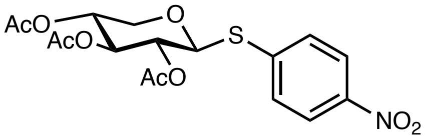

β-D-Xylopyranose tetraaacetate (**S18**, 1.00 g, 3.15 mmol) and 4-nitrophenyl thiol (1.33 g, 8.55 mmol) were dissolved in dry dichloromethane (12 mL). Reaction mixture was cooled to 0 ºC and $BF_3 \cdot OEt_2$ (1.5 mL, 9.45 mmol) was added dropwise. Mixture was allowed to warm to room temperature and stirred for 5 h. The reaction was poured over ice cold saturated $NaHCO_3$ solution and diluted with dichloromethane. Organic phase was washed with saturated $NaHCO_3$ solution and brine, dried over anhydrous Na_2SO_4, filtered and concentrated *in vacuo*. Purification by normal phase MPLC (toluene/ethyl acetate, 5–20% ethyl acetate) gave compound **S19** (573 mg, 1.39 mmol, 44%). The analytical data match the literature.[14]

^{1}H NMR (500 MHz, MeOH-d_4) δ 8.19 – 8.13 (m, 2H, ArH), 7.60 – 7.53 (m, 2H, ArH), 5.20 (t, *J* = 7.4 Hz, 1H, H-3), 5.07 (d, *J* = 7.3 Hz, 1H, H-1), 4.99 (t, *J* = 7.3 Hz, 1H, H-2), 4.94 (td, *J* = 7.6, 4.5 Hz, 1H, H-4), 4.35 (dd, *J* = 12.1, 4.5 Hz, 1H, Heq-5), 3.56 (dd, *J* = 12.0, 7.7 Hz, 1H, Hax-5), 2.10 (s, 3H, CH_3), 2.09 (s, 3H, CH_3), 2.08 (s, 3H, CH_3).

HPLC-MS: $[C_{17}H_{19}NO_9S + Na]^+$ calcd. 436.07, found 436.11.

***p*-Aminophenyl-thio-β-D-xylopyranose triacetate (S20)**

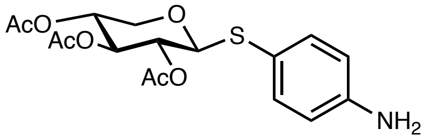

Compound **S19** (553 mg, 1.34 mmol) was dissolved in dry dichloromethane (15 mL) and 10% Pd/C (54.7 mg, 0.05 mmol) was added. After three vacuum/H_2 cycles the reaction was stirred under H_2 atmosphere (1 atm) overnight. The reaction was filtered over celite and concentrated *in vacuo*. Pure product **S20** was obtained (517 mg, 1.34 mmol, quant.) without further purification.

^{1}H NMR (500 MHz, DMSO-d_6) δ 7.11 – 7.06 (m, 2H, ArH), 6.54 – 6.49 (m, 2H, ArH), 5.37 (s, 2H, NH_2), 5.19 (t, *J* = 8.9 Hz, 1H, H-3), 4.79 – 4.69 (m, 2H, H-1, H-4), 4.67 (t, *J* = 9.0 Hz, 1H, H-2), 3.99 (dd, *J* = 11.3, 5.3 Hz, 1H, Heq-5), 3.49 (dd, *J* = 11.3, 9.9 Hz, 1H, Hax-5), 2.04 (s, 3H, CH_3), 1.97 (s, 3H, CH_3), 1.95 (s, 3H, CH_3).

^{13}C NMR (126 MHz, DMSO-d$_6$) δ 169.45 (2C, C=O), 168.92 (1C, C=O), 149.68 (1C, ArC), 135.89 (2C, ArCH), 114.09 (2C, ArCH), 113.87 (1C, ArC), 85.60 (1C, C-1), 72.39 (1C, C-3), 69.67 (1C, C-2), 68.32 (1C, C-4), 64.84 (1C, C-5), 20.51 (1C, CH_3), 20.44 (1C, CH_3), 20.35 (1C, CH_3).
HPLC-MS: $[C_{17}H_{21}NO_7S + H]^+$ calcd. 384.11, found 384.16.
HRMS: $[C_{17}H_{21}NO_7S + H]^+$ calcd. 384.1111, found 384.1105.

Xyloside S21

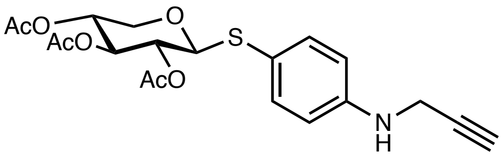

Compound **S20** (221 mg, 0.58 mmol) was dissolved in dry dimethylformamide (2 mL) and potassium carbonate (159 mg, 1.15 mmol) was added. The reaction mixture was cooled to 0 ºC and propargyl bromide (125 µL, 1.28 mmol) was added dropwise. The mixture was allowed to warm to room temperature and then heated to 40 ºC. After 6 h, the reaction was cooled to room temperature, poured over cold water and diluted with dichloromethane. Organic phase was washed with water, dried over anhydrous Na_2SO_4, filtered and concentrated *in vacuo*. Purification by normal phase MPLC (toluene/ethyl acetate + 0.25% Et_3N, 5–20% ethyl acetate) gave compound **S21** (152 mg, 0.36 mmol, 63%).
^1H NMR (500 MHz, DMSO-d$_6$) δ 7.23 – 7.16 (m, 2H, ArH), 6.66 – 6.55 (m, 2H, ArH), 6.33 (t, *J* = 6.1 Hz, 1H, NH), 5.20 (t, *J* = 9.0 Hz, 1H, H-3), 4.82 (d, *J* = 9.3 Hz, 1H, H-1), 4.77 – 4.66 (m, 2H, H-4, H-2), 3.99 (dd, *J* = 11.3, 5.3 Hz, 1H, Heq.-5), 3.87 (dd, *J* = 6.1, 2.5 Hz, 2H, CH_2), 3.51 (dd, *J* = 11.3, 9.9 Hz, 1H, Hax-5), 3.10 – 3.05 (m, 1H, C≡CH), 2.05 (s, 3H, CH_3), 1.97 (s, 3H, CH_3), 1.95 (s, 3H, CH_3).
^{13}C NMR (126 MHz, DMSO-d$_6$) δ 169.52 (1C, C=O), 169.51 (1C, C=O), 169.03 (1C, C=O), 148.47 (1C, ArC), 135.61 (2C, ArCH), 115.42 (1C, ArC), 113.02 (2C, ArCH), 85.54 (1C, C-1), 81.87 (1C, C≡CH), 73.16 (1C, C≡CH), 72.34 (1C, C-3), 69.73 (1C, C-2), 68.34 (1C, C-4), 64.85 (1C, C-5), 31.84 (1C, CH_2), 20.56 (1C, CH_3), 20.49 (1C, CH_3), 20.40 (1C, CH_3).
HPLC-MS: $[C_{20}H_{23}NO_7S + H]^+$ calcd. 422.13, found 422.14.
HRMS: $[C_{20}H_{23}NO_7S + H]^+$ calcd. 422.1268, found 422.1261.

Xyloside precursor S22

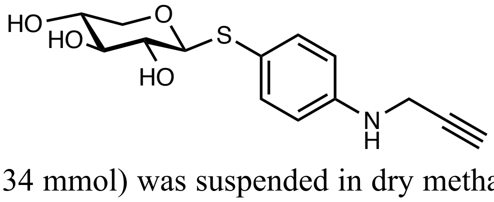

Compound **S21** (142 mg, 0.34 mmol) was suspended in dry methanol (4 mL) and 1M sodium methoxide in methanol (cat.) was added and stirred overnight. The reaction mixture was neutralized with 1M HCl solution and the solvent was removed *in vacuo*. Purification by normal phase MPLC (dichloromethane/ethanol + 1% NH_4OH, 1–20% ethanol) gave compound **S22** (88.5 mg, 0.30 mmol, 89%).
^1H NMR 1H NMR (500 MHz, MeOH-d$_4$) δ 7.40 – 7.32 (m, 2H, ArH), 6.68 – 6.59 (m, 2H, ArH), 4.26 (d, *J* = 9.4 Hz, 1H, H-1), 3.92 – 3.86 (m, 3H, Heq-5, CH_2), 3.43 – 3.37 (m, 1H, H-4), 3.31 – 3.28 (m, 1H, H-3), 3.18 – 3.08 (m, 2H, H-2, Hax-5), 2.51 (t, *J* = 2.4 Hz, 1H, C≡CH).

^{13}C NMR (126 MHz, MeOH-d$_4$) δ 149.75 (1C, ArC), 137.06 (2C, ArCH), 119.17 (1C, ArC), 114.49 (2C, ArCH), 90.91 (1C, C-1), 82.15 (1C, C≡CH), 79.42 (1C, C-3), 73.36 (1C, C-2), 71.88 (1C, C≡CH), 70.94 (1C, C-4), 70.62 (1C, C-5), 33.51 (1C, CH$_2$).
HPLC-MS: [$C_{14}H_{17}NO_4S$ + H]$^+$ calcd. 296.10, found 296.14.
HRMS: [$C_{14}H_{17}NO_4S$ + H]+ calcd. 296.0951, found 296.0949.

Xyloside S23

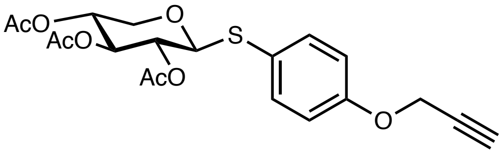

β-D-Xylopyranose tetraacetate (**S18**, 1.21 g, 3.81 mmol) and 4-prop-2-ynyloxyphenyl thiol (**S9**, 365 mg, 2.22 mmol) were dissolved in mixture of dry dichloromethane (20 mL) and toluene (15 mL). Reaction mixture was cooled to 0 °C and $BF_3 \cdot OEt_2$ (1 mL, 7.96 mmol) was added dropwise. Mixture was allowed to warm to room temperature and stirred for 5 h. The reaction was poured over ice cold saturated $NaHCO_3$ solution and diluted with dichloromethane. Organic phase was washed with saturated $NaHCO_3$ solution and brine, dried over anhydrous Na_2SO_4, filtered and concentrated *in vacuo*. Purification by normal phase MPLC (toluene/ethyl acetate, 5–20% ethyl acetate) gave compound **S23** (176 mg, 0.42 mmol, 12%).
^{1}H NMR (500 MHz, CDCl$_3$) δ 7.43 (d, J = 8.5 Hz, 2H, ArH), 6.93 (d, J = 8.5 Hz, 2H, ArH), 5.16 (t, J = 8.4 Hz, 1H, H-3), 4.92 – 4.83 (m, 2H, H-2, H-4), 4.69 (s, 2H, CH$_2$), 4.63 (d, J = 8.6 Hz, 1H, H-1), 4.23 (dd, J = 11.7, 5.0 Hz, 1H, H-5eq.), 3.41 – 3.33 (m, 1H, H-5ax.), 2.54 (s, 1H, C≡CH), 2.10 (s, 3H, CH$_3$), 2.03 (s, 6H, CH$_3$).
^{13}C NMR (126 MHz, CDCl$_3$) δ 170.12 (1C, C=O), 169.92 (1C, C=O), 169.49 (1C, C=O), 158.31 (1C, ArC), 136.03 (2C, ArCH), 122.94 (1C, ArC), 115.55 (2C, ArCH), 86.53 (1C, C-1), 78.27 (1C, C≡CH), 76.01 (1C, C≡CH), 72.48 (1C, C-3), 69.95 (1C, C-2), 68.59 (1C, C-4), 65.61 (1C, C-5), 55.95 (1C, CH$_2$), 20.97 (1C, CH$_3$), 20.85 (2C, CH$_3$).
HPLC-MS: [$C_{20}H_{22}O_8S$ + NH_4]$^+$ calcd. 440.14, found 440.12.
HRMS: [$C_{20}H_{22}O_8S$ + Na]$^+$ calcd. 445.0928, found 445.0918.

Xyloside precursor (S24)

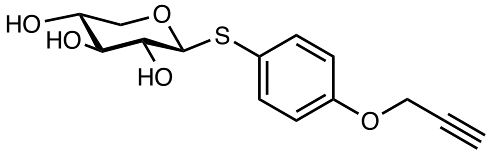

Compound **S23** (157.3 mg, 0.37 mmol) was dissolved in dry methanol (10 mL) and 1M sodium methoxide in methanol (cat.) was added. After 5h, the reaction mixture was neutralized with Amberlite IR 120/H$^+$. The solvent was removed *in vacuo*. Purification by normal phase MPLC (dichloromethane/methanol, 1–20% methanol) gave compound **S24** (83.0 mg, 0.28 mmol, 75%).
^{1}H NMR (500 MHz, MeOH-d$_4$) δ 7.53 – 7.46 (m, 2H, ArH), 6.97 – 6.92 (m, 2H, ArH), 4.73 (d, J = 2.4 Hz, 2H, CH$_2$), 4.37 (d, J = 9.4 Hz, 1H, H-1), 3.91 (dd, J = 11.3, 5.2 Hz, 1H, H-5eq.), 3.46 – 3.37 (m, 1H, H-4), 3.34 – 3.29 (m, 1H, H-3), 3.22 – 3.08 (m, 2H, H-2, H-5ax.), 2.96 (t, J = 2.4 Hz, 1H, C≡CH).

^{13}C NMR (126 MHz, MeOH-d$_4$) δ 159.41 (1C, ArC), 136.53 (2C, ArCH), 125.15 (1C, ArC), 116.36 (2C, ArCH), 90.56 (1C, C-1), 79.55 (1C, C̲≡CH), 79.31 (1C, C-3), 76.95 (1C, C≡C̲H), 73.50 (1C, C-2), 70.91 (1C, C-4), 70.56 (1C, C-5), 56.64 (1C, CH_2).
HPLC-MS: $[C_{14}H_{16}O_6S + NH_4]^+$ calcd. 314.11, found 314.06.
HRMS: $[C_{14}H_{16}O_6S + Na]^+$ calcd. 319.0610, found 319.0610.

5-(3-(2-(2-(2-(2-azidoethoxy)ethoxy)ethoxy)ethyl)thioureido)-2-(6-hydroxy-3-oxo-3H-xanthen-9-yl)benzoic acid (S29)

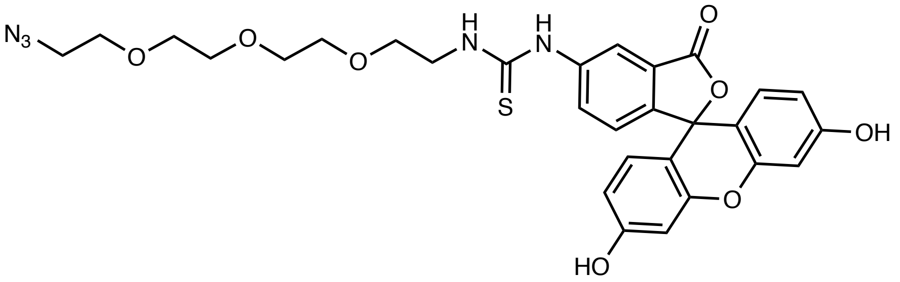

Fluorescein 5-isothiocyanate (**S25**, 109.1 mg, 0.25 mmol) was dissolved in dimethylformamide (1 mL) and triethylamine (100 µL, 0.72 mmol) was added. The reaction mixture was cooled to 0 °C and a solution of 11-azido-3,6,9-trioxaundecan-1-amine (68 µL, 0.34 mmol) in dimethylformamide (0.5 mL) was added. The mixture was allowed to warm to r.t. and stirred for 1 h. The reaction mixture was dried *in vacuo*. Purification by normal phase MPLC (dichloromethane/methanol with 1% formic acid, 1–20% methanol) gave the azide modified fluorescein **9** (97.9 mg, 0.16 mmol, 59%). Synthesis of **9** was first described by Loison *et. al.*.[15]
^1H NMR (500 MHz, MeOH-d$_4$) δ 8.18 (d, J = 1.7 Hz, 1H, ArH), 7.80 (d, J = 7.9 Hz, 1H, ArH), 7.17 (d, J = 8.2 Hz, 1H, ArH), 6.73 – 6.68 (m, 4H, ArH), 6.57 (dd, J = 8.7, 2.4 Hz, 2H, ArH), 3.82 (s, 2H, NHCSNHC$\underline{H_2}$), 3.71 (t, J = 5.1 Hz, 2H, NHCSNHCH$_2$C$\underline{H_2}$), 3.68 (s, 4H, CH_2), 3.66 – 3.59 (m, 6H, CH_2), 3.36 – 3.32 (m, 2H, CH_2N_3).
^{13}C NMR (126 MHz, MeOH-d$_4$) δ 182.90 (1C, C=S), 171.01 (1C, C=O), 161.74 (2C, ArCOH), 154.39 (2C, ArC), 142.53 (1C, ArCNH), 131.61 (1C, ArCH), 130.46 (2C, ArCH), 129.06 (1C, ArC), 125.88 (1C, ArCH), 119.90 (1C, ArCH), 113.85 (2C, ArCH), 111.71 (2C, ArC), 103.51 (2C, ArCH), 71.65 (1C, CH_2), 71.56 (1C, CH_2), 71.49 (1C, CH_2), 71.33 (1C, CH_2), 71.09 (1C, CH_2), 70.20 (1C, CH_2), 51.73 (1C, CH_2N_3), 45.51 (1C, NHCSNHC̲H$_2$).
HPLC-MS: $[C_{29}H_{29}N_5O_8S + H]^+$ calcd. 608.18, found. 608.21.
HRMS: $[C_{29}H_{29}N_5O_8S + H]^+$ calcd. 608.1810, found 608.1803.

Imaging probe 2

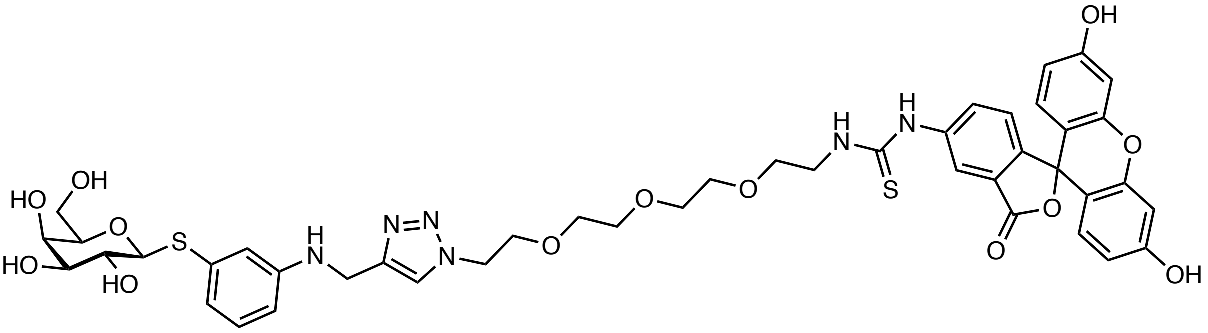

Azide modified fluorescein (**9**, 21.0 mg, 34.7 µmol) and galactoside **S5*m*** (16.3 mg, 50.1 µmol) were dissolved in dimethylformamide (2 mL). $CuSO_4$ solution (315 µL, 100 mM in water,

32 µmol) and sodium ascorbate solution (630 µL, 100 mM in water, 63 µmol) were added. The reaction was stirred at r.t. overnight. After lyophilization, product was purified by preparative HPLC (water/acetonitrile with 0.1% formic acid, 20–35% acetonitrile). Compound **2** was obtained as yellow solid (15.8 mg, 16.9 µmol, 49%).

^{1}H NMR (500 MHz, DMSO-d_6) δ 10.11 (s, 2H, OH-fluorescein), 8.28 (s, 1H, ArH-fluorescein), 8.17 (s, 1H, NH-thiourea), 7.91 (s, 1H, CH-triazole), 7.74 (d, *J* = 7.6 Hz, 1H, ArH-fluorescein), 7.17 (d, *J* = 8.3 Hz, 1H, ArH-fluorescein), 6.97 (t, *J* = 7.9 Hz, 1H, ArH), 6.74 (s, 1H, ArH), 6.67 (d, *J* = 2.2 Hz, 2H, ArH-fluorescein), 6.63 – 6.58 (m, 3H, ArH, ArH-fluorescein), 6.57 (d, *J* = 2.2 Hz, 1H, ArH-fluorescein), 6.55 (d, *J* = 2.3 Hz, 1H, ArH-fluorescein), 6.48 (dd, *J* = 8.1, 1.7 Hz, 1H, ArH), 6.08 (t, *J* = 5.8 Hz, 1H, NH), 5.08 (s, 1H, OH-2), 4.86 (s, 1H, OH-3), 4.62 (s, 1H, OH-6), 4.53 – 4.44 (m, 4H, H-1, CH_2, OH-4), 4.27 (d, *J* = 5.8 Hz, 2H, $NHCH_2$), 3.78 (t, *J* = 5.2 Hz, 2H, CH_2), 3.71 (s, 1H, H-4), 3.67 (s, 2H, CH_2), 3.58 (t, *J* = 5.4 Hz, 2H, CH_2), 3.56 – 3.38 (m, 13H, H-2, H-3, H-5, H-6, CH_2).

^{13}C NMR (126 MHz, DMSO-d_6) δ 180.56 (1C, C=S), 168.57 (1C, C=O), 159.68 (2C, ArC-fluorescein), 151.95 (2C, ArC-fluorescein), 148.69 (1C, ArC), 146.92 (2C, ArC-fluorescein), 145.44 (1C, C=CH-triazole), 141.36 (1C, ArC-fluorescein), 136.03 (1C, ArC), 129.35 (4C, ArCH-fluorescein), 129.09 (1C, ArCH), 126.74 (1C, ArCH-fluorescein), 124.14 (1C, ArCH-fluorescein), 123.11 (1C, C=CH-triazole), 116.74 (1C, ArCH), 116.47 (1C, ArCH-fluorescein), 112.72 (1C, ArCH-fluorescein), 112.49 (1C, ArCH), 110.60 (1C, ArCH), 109.80 (2C, ArC-fluorescein), 102.26 (2C, ArCH-fluorescein), 88.01 (1C, C-1), 79.03 (1C, C-5), 74.78 (1C, C-3), 69.75 (1C, CH_2), 69.65 (2C, CH_2), 69.58 (1C, CH_2), 69.28 (1C, CH_2), 68.81 (1C, C-2), 68.42 (1C, CH_2), 68.33 (1C, C-4), 60.54 (1C, C-6), 49.33 (1C, CH_2), 43.70 (1C, CH_2), 38.40 (1C, $NHCH_2$).

HPLC-MS: $[C_{44}H_{48}N_6O_{13}S_2 + H]^+$ calcd. 933.28, found. 933.35.

HRMS: $[C_{44}H_{48}N_6O_{13}S_2 + H]^+$ calcd. 933.2794, found 933.2769.

Imaging probe 3

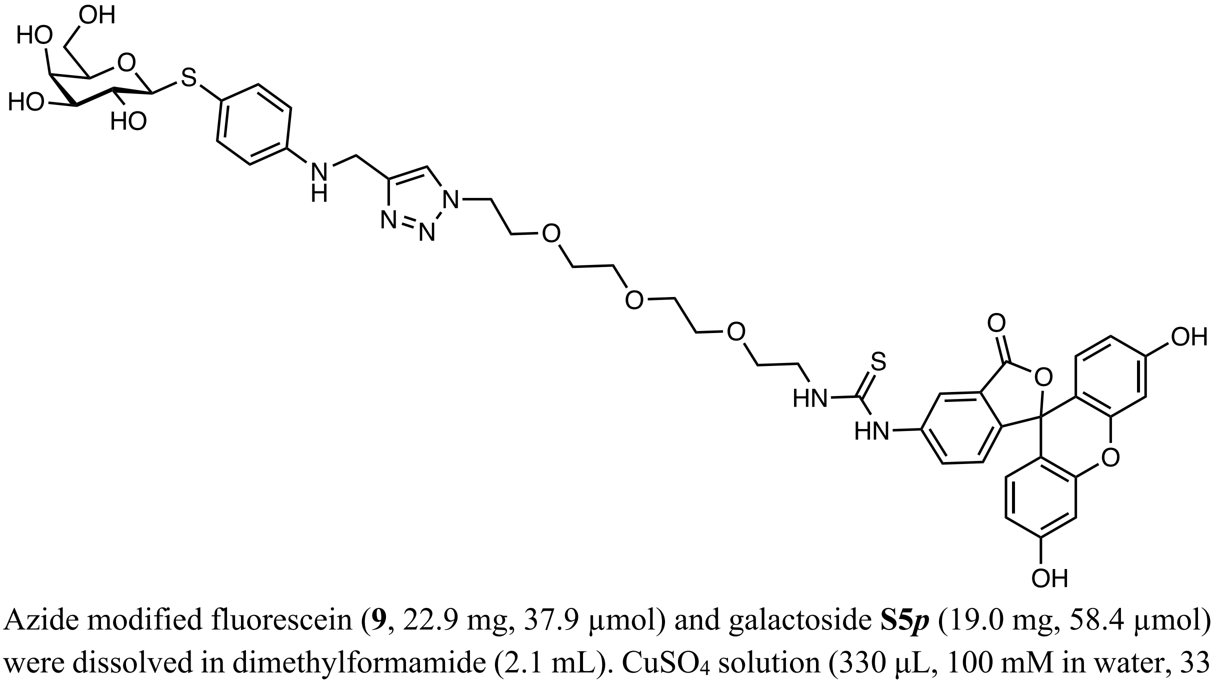

Azide modified fluorescein (**9**, 22.9 mg, 37.9 µmol) and galactoside **S5*p*** (19.0 mg, 58.4 µmol) were dissolved in dimethylformamide (2.1 mL). $CuSO_4$ solution (330 µL, 100 mM in water, 33 µmol) and sodium ascorbate solution (660 µL, 100 mM in water, 66 µmol) were added. The

reaction was stirred at r.t. overnight. After lyophilization, product was purified by preparative HPLC (water/acetonitrile with 0.1% formic acid, 20–35% acetonitrile). Compound **3** was obtained as yellow solid (21 mg, 22.5 µmol, 60%).

^{1}H NMR (500 MHz, DMSO-d$_6$) δ 10.09 (s, 2H, OH-fluorescein), 8.26 (s, 1H, ArH-fluorescein), 8.15 (s, 1H, NH-thiourea), 7.92 (s, 1H, CH-triazole), 7.73 (d, J = 7.2 Hz, 1H, ArH-fluorescein), 7.23 (d, J = 8.6 Hz, 2H, ArH), 7.16 (d, J = 8.3 Hz, 1H, ArH-fluorescein), 6.68 – 6.52 (m, 8H, ArH, ArH-fluorescein), 6.23 (t, J = 5.7 Hz, 1H, NH), 4.93 (s, 1H, OH-2), 4.78 (s, 1H, OH-3), 4.57 (s, 1H, OH-6), 4.47 (t, J = 5.1 Hz, 2H, CH$_2$), 4.38 – 4.31 (m, 1H, OH-4), 4.27 (d, J = 5.7 Hz, 2H, NHCH$_2$), 4.20 (d, J = 8.9 Hz, 1H, H-1), 3.77 (t, J = 5.2 Hz, 2H, CH$_2$), 3.73 – 3.61 (m, 3H, H-4, CH$_2$), 3.58 (t, J = 5.3 Hz, 2H, CH$_2$), 3.56 – 3.52 (m, 2H, CH$_2$), 3.52 – 3.41 (m, 8H, H-6, CH$_2$), 3.32 – 3.24 (m, 3H, H-2, H-3, H-5).

^{13}C NMR (126 MHz, DMSO-d$_6$) δ 180.56 (1C, C=S), 168.59 (1C, C=O), 160.05 (2C, ArC-fluorescein), 152.18 (2C, ArC-fluorescein), 148.15 (1C, ArC), 145.39 (1C, C=CH-triazole), 141.29 (1C, ArC-fluorescein), 134.18 (2C, ArCH) 129.17 (4C, ArC-fluorescein), 124.38 (1C, ArCH-fluorescein), 123.17 (1C, C=CH-triazole), 118.31 (1C, ArC), 116.88 (1C, ArC-fluorescein), 113.09 (1C, ArC-fluorescein), 112.58 (2C, ArCH), 109.97 (2C, ArC-fluorescein), 102.29 (2C, ArCH-fluorescein), 89.71 (1C, C-1), 79.01 (1C, C-5), 74.75 (1C, C-3), 69.76 (1C, CH$_2$), 69.66 (2C, CH$_2$), 69.58 (1C, CH$_2$), 69.28 (1C, CH$_2$), 68.80 (1C, C-2), 68.42 (1C, CH$_2$), 68.29 (1C, C-4), 60.51 (1C, C-6), 49.34 (1C, CH$_2$), 43.71 (1C, CH$_2$), 38.42 (1C, NHCH$_2$).

HPLC-MS: [$C_{44}H_{48}N_6O_{13}S_2$ + H]$^+$ calcd. 933.28, found. 933.34.

HRMS: [$C_{44}H_{48}N_6O_{13}S_2$ + H]$^+$ calcd. 933.2794, found 933.2777.

Imaging probe 4

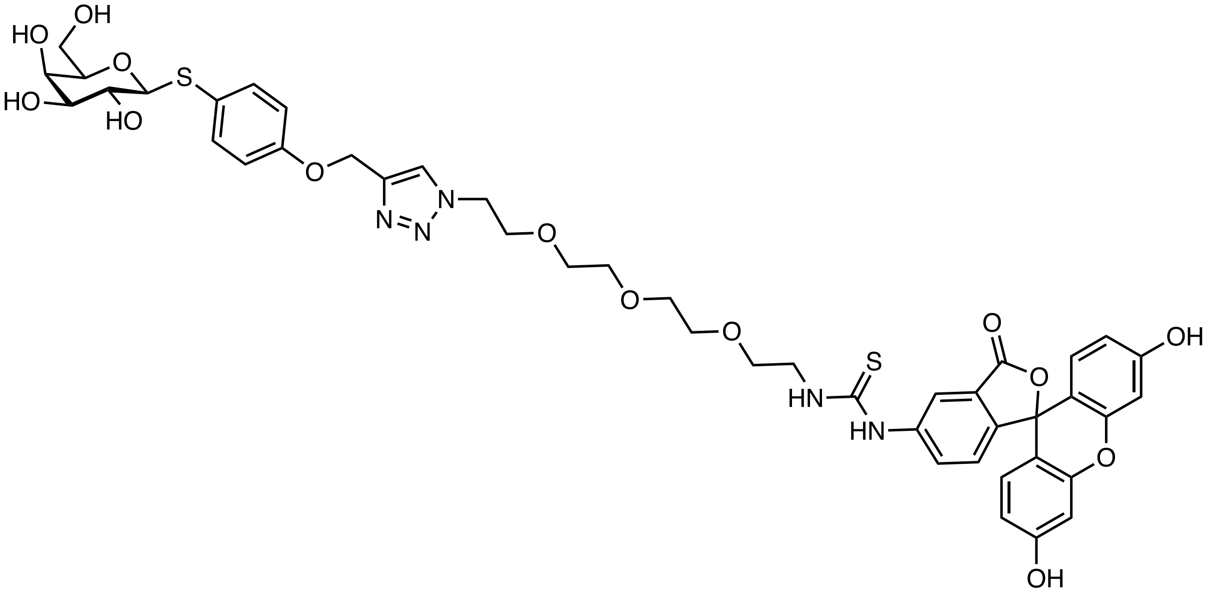

Azide modified fluorescein (**9**, 15.4 mg, 25.3 µmol) and galactoside **S11** (11.0 mg, 33.7 µmol) were dissolved in dimethylformamide (1.5 mL). $CuSO_4$ solution (125 µL, 100 mM in water, 12.5 µmol) and sodium ascorbate solution (125 µL, 100 mM in water, 12.5 µmol) were added. The reaction was stirred at r.t. overnight. After lyophilization, product was purified by preparative HPLC (water/acetonitrile with 0.1% formic acid, 20–40% acetonitrile). Compound **4** was obtained as yellow solid (15.9 mg, 17.0 µmol, 67%).

^{1}H NMR (500 MHz, MeOH-d_4) δ 8.13 (s, 1H, ArH-fluorescein), 8.10 (s, 1H, CH-triazole), 7.77 (d, *J* = 7.3 Hz, 1H, ArH-fluorescein), 7.57 – 7.49 (m, 2H, ArH), 7.16 (d, *J* = 8.2 Hz, 1H, ArH-fluorescein), 6.99 – 6.93 (m, 2H, ArH), 6.75 (d, *J* = 8.7 Hz, 2H, ArH-fluorescein), 6.69 (d, *J* = 2.3 Hz, 2H, ArH-fluorescein), 6.58 (d, *J* = 2.3 Hz, 1H, ArH-fluorescein), 6.56 (d, *J* = 2.4 Hz, 1H, ArH-fluorescein), 6.57 (dd, *J* = 8.8, 2.4 Hz, 2H, ArH-fluorescein), 5.15 (s, 2H, CH_2), 4.57 (t, *J* = 4.9 Hz, 2H, CH_2), 4.44 (d, *J* = 9.5 Hz, 1H, H-1), 3.91 – 3.85 (m, 3H, H-4, CH_2), 3.81 (s, 2H, CH_2), 3.79 – 3.70 (m, 2H, H-6), 3.70 – 3.66 (m, 2H, CH_2), 3.65 – 3.48 (m, 11H, H-2, H-3, H-5, CH_2).
^{13}C NMR (126 MHz, MeOH-d_4) δ 182.73 (1C, C=S), 171.37 (1C, C=O), 159.60 (1C, ArC), 154.76 (2C, ArC-fluorescein), 144.62 (1C, $\underline{C}$=CH-triazole), 135.59 (2C, ArCH), 130.66 (4C, ArCH-fluorescein), 126.36 (1C, C=$\underline{C}$H-triazole), 126.24 (1C, ArC), 120.33(1C, ArCH-fluorescein), 116.35 (2C, ArCH), 114.58 (1C, ArCH-fluorescein), 112.05 (1C, ArC-fluorescein), 103.58 (2C, ArCH-fluorescein), 90.99 (1C, C-1), 80.53 (1C, C-5), 76.31 (1C, C-3), 71.55 (1C, CH_2), 71.42 (1C, CH_2), 71.40 (1C, CH_2), 71.32 (1C, CH_2), 70.96 (1C, C-2), 70.43 (1C, C-4), 70.26 (2C, CH_2), 62.61 (1C, C-6), 62.42 (1C, CH_2), 51.47 (1C, CH_2), 45.48 (1C, CH_2).
HPLC-MS: $[C_{44}H_{47}N_5O_{14}S_2 + H]^+$ calcd. 934.26, found. 934.34.
HRMS: $[C_{44}H_{47}N_5O_{14}S_2 + H]^+$ calcd. 934.2634, found 934.2622.

Imaging probe 6

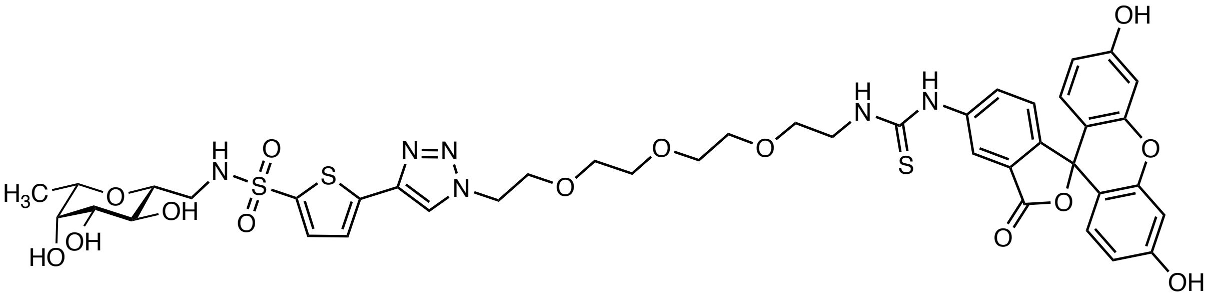

Azide modified fluorescein (**9**, 21.8 mg, 35.9 µmol) and C-fucoside **25** (15.7 mg, 45.2 µmol) were dissolved in dimethylformamide (2.2 mL). $CuSO_4$ solution (35 µL, 100 mM in water, 3.5 µmol) and sodium ascorbate solution (85 µL, 100 mM in water, 8.5 µmol) were added. The reaction was stirred at r.t. overnight. After lyophilization, product was purified by preparative HPLC (water/acetonitrile with 0.1% formic acid, 20–40% acetonitrile). Compound **6** was obtained as yellow solid (29.0 mg, 16.8 µmol, 85%).
^{1}H NMR (500 MHz, DMSO-d_6) δ 10.03 (s, 2H, OH-fluorescein), 8.61 (s, 1H, CH-triazole), 8.27 (s, 1H, ArH-fluorescein), 8.09 (s, 1H, NH-thiourea), 7.73 (d, *J* = 7.5 Hz, 1H, ArH-fluorescein), 7.56 (d, *J* = 3.9 Hz, 1H, ArH), 7.46 (d, *J* = 3.8 Hz, 1H, ArH), 7.17 (d, *J* = 8.3 Hz, 1H, ArH-fluorescein), 6.67 (d, *J* = 2.2 Hz, 2H, ArH-fluorescein), 6.63 – 6.53 (m, 4H, ArH-fluorescein), 4.83 (s, 1H, OH-2), 4.61 (s, 1H, OH-3), 4.58 (t, *J* = 5.1 Hz, 2H, CH_2), 4.28 (d, *J* = 5.2 Hz, 1H, OH-4), 3.85 (t, *J* = 5.1 Hz, 2H, CH_2), 3.67 (s, 2H, CH_2), 3.58 (t, *J* = 5.4 Hz, 2H, CH_2), 3.56 – 3.48 (m, 8H, CH_2), 3.42 – 3.38 (m, 2H, H-4, H-5), 3.31 – 3.28 (m, 1H, $C\underline{H}_{2,a}NHSO_2$), 3.89 – 3.80 (m, 1H, H-3), 3.16 (t, *J* = 9.2 Hz, 1H, H-1), 3.06 (td, *J* = 8.9, 2.0 Hz, 1H, H-2), 2.82 (dd, *J* = 13.0, 8.4 Hz, 1H, $C\underline{H}_{2,b}NHSO_2$), 1.07 (d, *J* = 6.4 Hz, 3H, CH_3).
^{13}C NMR (126 MHz, DMSO-d_6) δ 180.55 (1C, C=S), 168.55 (1C, C=O), 159.66 (2C, ArC-fluorescein), 151.94 (2C, ArC-fluorescein), 146.95 (1C, ArC-fluorescein), 141.32 (1C, ArC-

fluorescein), 140.31 (1C, ArC), 139.78 (1C, $\underline{C}$=CH-triazole), 138.55 (1C, ArC), 132.08 (1C, ArCH), 129.37 (1C, ArCH-fluorescein), 129.08 (4C, ArCH-fluorescein), 126.73 (1C, ArC-fluorescein), 124.15 (1C, ArCH-fluorescein), 123.80 (1C, ArCH), 122.44 (1C, C=$\underline{C}$H-triazole), 116.49 (1C, ArCH-fluorescein), 112.70 (1C, ArCH-fluorescein), 109.79 (2C, ArC-fluorescein), 102.26 (2C, ArCH-fluorescein), 78.31 (1C, C-2), 74.66 (1C, C-3), 73.67 (1C, C-5), 71.60 (1C, C-4), 69.74 (1C, CH_2), 69.62 (3C, CH_2), 68.52 (1C, CH_2), 68.42 (1C, CH_2), 68.30 (1C, C-1), 49.87 (1C, CH_2), 44.81 (1C, CH_2NHSO_2), 43.69 (1C, CH_2), 16.94 (1C, CH_3).
HPLC-MS: $[C_{42}H_{46}N_6O_{14}S_3 + H]^+$ calcd. 955.23, found. 955.30.
HRMS: $[C_{42}H_{46}N_6O_{14}S_3 + H]^+$ calcd. 955.2307, found 955.2295.

Imaging probe 7

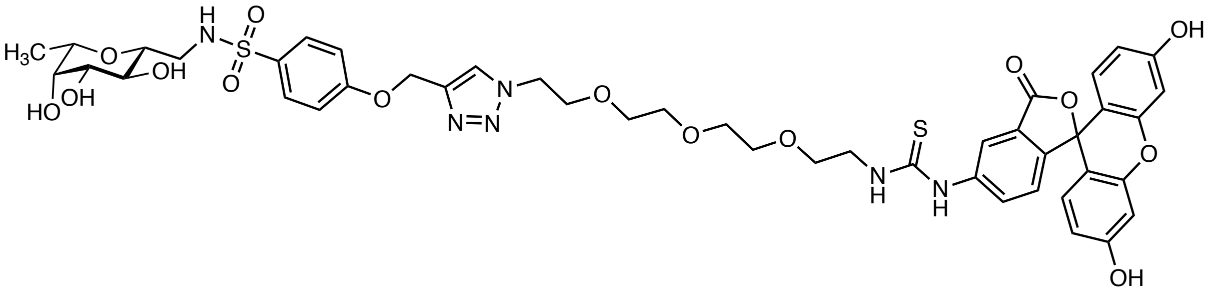

Azide modified fluorescein (**9**, 16.3 mg, 26.8 μmol) and C-fucoside **S24** (13.1 mg, 35.3 μmol) were dissolved in dimethylformamide (2 mL). $CuSO_4$ solution (45 μL, 100 mM in water, 4.5 μmol) and sodium ascorbate solution (95 μL, 100 mM in water, 9.5 μmol) were added. The reaction was stirred at r.t. overnight. After lyophilization, product was purified by preparative HPLC (water/acetonitrile with 0.1% formic acid, 20–40% acetonitrile). Compound **7** was obtained as yellow solid (18.1 mg, 18.5 μmol, 69%).
^{1}H NMR (500 MHz, DMSO-d_6) δ 10.06 (s, 2H, OH-fluorescein), 8.26 (s, 1H, ArH-fluorescein), 8.22 (s, 1H, CH-triazole), 8.12 (s, 1H, NH-thiourea), 7.73 (d, J = 8.9 Hz, 3H, ArH, ArH-fluorescein), 7.37 (s, 1H, NH-thiourea), 7.20 (d, J = 8.9 Hz, 2H, ArH), 7.17 (d, J = 8.3 Hz, 1H, ArH-fluorescein), 6.67 (d, J = 2.2 Hz, 2H, ArH-fluorescein), 6.62 – 6.54 (m, 4H, ArH-fluorescein), 5.23 (s, 2H, $PhOCH_2$), 4.77 (s, 1H, OH-2), 4.59 (s, 1H, OH-3), 4.53 (t, J = 5.2 Hz, 2H, CH_2), 4.27 (d, J = 4.9 Hz, 1H, OH-4), 3.82 (t, J = 5.2 Hz, 2H, CH_2), 3.67 (s, 2H, CH_2), 3.58 (t, J = 5.4 Hz, 2H, CH_2), 3.57 – 3.47 (m, 8H, CH_2), 3.36 (m, 42H, H-4, H-5), 3.23 – 3.08 (m, 3H, H-3, H-1, $C\underline{H}_{2,a}NHSO_2$), 2.98 (td, J = 8.9, 2.1 Hz, 1H, H-2), 2.69 – 2.62 (m, 1H, $C\underline{H}_{2,b}NHSO_2$), 1.06 (d, J = 6.4 Hz, 3H, CH_3).
^{13}C NMR (126 MHz, DMSO-d_6) δ 180.56 (1C, C=S), 168.57 (1C, C=O), 160.77 (1C, ArC), 159.74 (2C, ArC-fluorescein), 151.98 (2C, ArC-fluorescein), 146.85 (1C, ArC-fluorescein), 141.97 (1C, $\underline{C}$=CH-triazole), 141.31 (1C, ArC-fluorescein), 132.79 (1C, ArC), 129.34 (1C, ArCH-fluorescein), 129.10 (4C, ArCH-fluorescein), 128.65 (2C, ArCH), 126.83 (1C, ArC-fluorescein), 125.19 (1C, C=$\underline{C}$H-triazole), 124.19 (1C, ArCH-fluorescein), 116.52 (1C, ArCH-fluorescein), 114.89 (2C, ArCH), 112.75 (1C, ArCH-fluorescein), 109.82 (2C, ArC-fluorescein), 102.27 (2C, ArCH-fluorescein), 78.34 (1C, C-2), 74.66 (1C, C-3), 73.63 (1C, C-5), 71.59 (1C, C-4), 69.76 (1C, CH_2), 69.68 (1C, CH_2), 69.65 (3C, CH_2), 69.56 (1C, CH_2), 68.68 (1C, CH_2), 68.42 (1C, CH_2), 68.32 (1C, C-1), 61.44 (1C, $PhOCH_2$), 49.48 (1C, CH_2), 44.54 (1C, CH_2NHSO_2), 43.71 (1C, CH_2), 16.94 (1C, CH_3).
HPLC-MS: $[C_{45}H_{50}N_6O_{15}S_2 + H]^+$ calcd. 979.28, found. 979.36.
HRMS: $[C_{45}H_{50}N_6O_{15}S_2 + H]^+$ calcd. 979.2849, found 979.2826.

Imaging probe 8

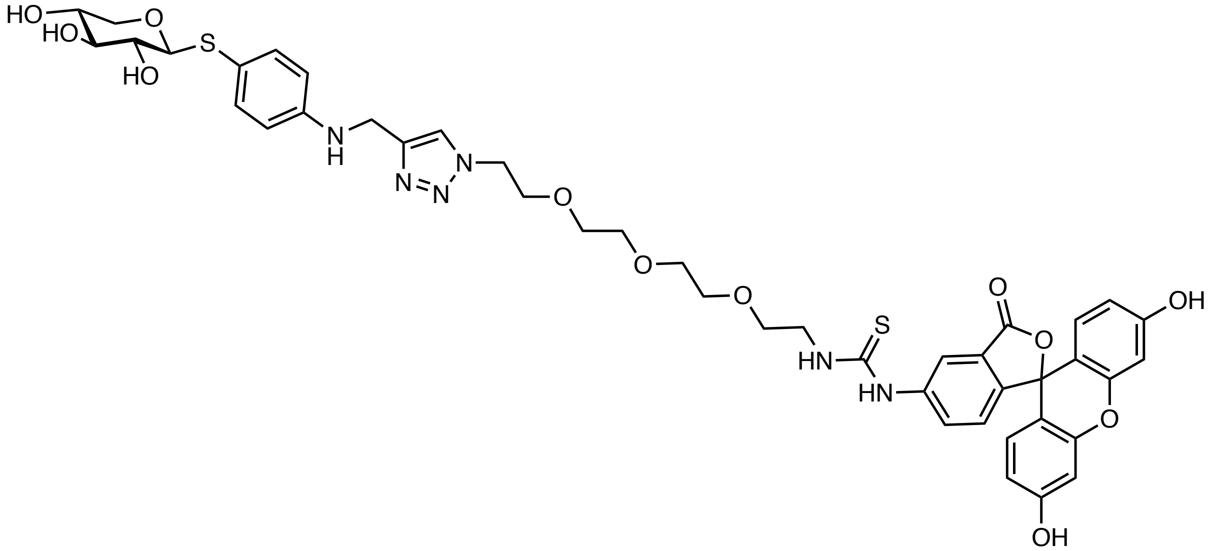

Azide modified fluorescein (**9**, 20.02 mg, 32.9 µmol) and xyloside **S22** (15.7 mg, 53.2 µmol) were dissolved in dimethylformamide (2 mL). $CuSO_4$ solution (330 µL, 100 mM in water, 33 µmol) and sodium ascorbate solution (660 µL, 100 mM in water, 66 µmol) were added. The reaction was stirred at r.t. for 2h. After lyophilization, product was purified by preparative HPLC (water/acetonitrile with 0.1% formic acid, 20–35% acetonitrile). Compound **8** was obtained as yellow solid (11.9 mg, 17.0 µmol, 40%).

^{1}H NMR (500 MHz, DMSO-d$_6$) δ 10.16 (s, 2H, OH-fluorescein), 8.27 (s, 1H, ArH-fluorescein), 8.21 (s, 1H, NH-thiourea), 7.93 (s, 1H, CH-triazole), 7.74 (d, *J* = 7.1 Hz, 1H, ArH-fluorescein), 7.20 (d, *J* = 8.5 Hz, 2H, ArH), 7.16 (d, *J* = 8.3 Hz, 1H, ArH-fluorescein), 6.66 (s, 2H, ArH-fluorescein), 6.63 – 6.52 (m, 7H, ArH, ArH-fluorescein), 6.30 (t, *J* = 5.7 Hz, 1H, NH), 5.11 (s, 1H, OH-2), 5.05 (s, 1H, OH-3), 4.95 (s, 1H, OH-4), 4.47 (t, *J* = 5.1 Hz, 2H, CH_2), 4.27 (d, *J* = 5.7 Hz, 2H, $NHCH_2$), 4.20 (d, *J* = 9.2 Hz, 1H, H-1), 3.78 (t, *J* = 5.1 Hz, 2H, CH_2), 3.74 – 3.62 (m, 3H, H-5eq., CH_2), 3.58 (t, *J* = 5.2 Hz, 2H, CH_2), 3.56 – 3.52 (m, 2H, CH_2), 3.52 – 3.44 (m, 8H, CH_2), 3.23 – 3.14 (m, 2H, H-4), 3.11 (t, *J* = 8.4 Hz, 1H, H-3), 2.99 (t, *J* = 10.6 Hz, 1H, H-5ax.), 2.95 – 2.88 (m, 1H, H-2).

^{13}C NMR (126 MHz, DMSO-d$_6$) δ 180.58 (1C, C=S), 168.60 (1C, C=O), 160.05 (2C, ArC-fluorescein), 152.08 (2C, ArC-fluorescein), 148.54 (1C, ArC), 145.32 (1C, <u>C</u>=CH-triazole), 141.36 (1C, ArC-fluorescein), 135.37 (2C, ArCH), 129.14 (4C, ArCH-fluorescein), 127.10 (1C, ArC-fluorescein), 124.27 (1C, ArCH-fluorescein), 123.18 (1C, C=<u>C</u>H-triazole), 116.58 (1C, ArC), 112.94 (1C, ArCH-fluorescein), 112.54 (2C, ArCH), 109.90 (2C, ArC-fluorescein), 102.28 (2C, ArCH-fluorescein), 89.17 (1C, C-1), 77.84 (1C, C-3), 72.03 (1C, C-2), 69.76 (1C, CH_2), 69.66 (2C, CH_2), 69.59 (1C, CH_2), 69.40 (1C, C-4), 69.25 (1C, C-5), 68.80 (1C, CH_2), 68.42 (1C, CH_2), 49.36 (1C, CH_2), 43.70 (1C, CH_2), 38.35 (1C, $NHCH_2$).

HPLC-MS: $[C_{43}H_{46}N_6O_{12}S_2 + H]^+$ calcd. 903.27, found. 903.33.

HRMS: $[C_{43}H_{46}N_6O_{12}S_2 + 2H]^{2+}$ calcd. 452.1381, found 452.1375.

Imaging probe S25

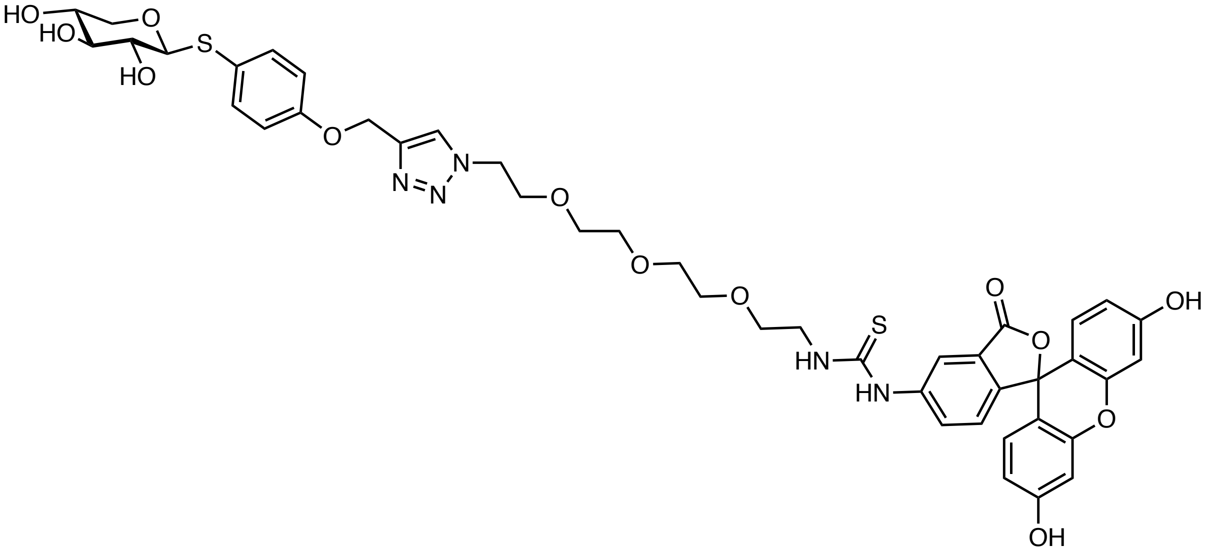

Azide modified fluorescein (**9**, 15.7 mg, 25.8 µmol) and xyloside **S24** (10.1 mg, 34.1 µmol) were dissolved in dimethylformamide (1.5 mL). $CuSO_4$ solution (125 µL, 100 mM in water, 12.5 µmol) and sodium ascorbate solution (125 µL, 100 mM in water, 12.5 µmol) were added. The reaction was stirred at r.t. for 2 days. After lyophilization, product was purified by preparative HPLC (water/acetonitrile with 0.1% formic acid, 20–40% acetonitrile). Compound **S25** was obtained as yellow solid (15.7 mg, 16.8 µmol, 65%).

^{1}H NMR (500 MHz, MeOH-d_4) δ 8.12 (s, 1H, ArH-fluorescein), 8.08 (s, 1H, CH-triazole), 7.74 (d, J = 6.5 Hz, 1H, ArH-fluorescein), 7.47 (d, J = 8.7 Hz, 2H, ArH), 7.14 (d, J = 8.2 Hz, 1H, ArH-fluorescein), 6.95 (d, J = 8.7 Hz, 2H, ArH), 6.73 – 6.64 (m, 4H, ArH-fluorescein), 6.55 (d, J = 2.1 Hz, 1H, ArH-fluorescein), 6.53 (d, J = 2.2 Hz, 1H, ArH-fluorescein), 5.14 (s, 2H, CH_2), 4.55 (t, J = 4.7 Hz, 2H, CH_2), 4.37 (d, J = 9.4 Hz, 1H, H-1), 3.93 – 3.73 (m, 5H, H-5eq, CH_2), 3.70 – 3.52 (m, 10H, CH_2), 4.37 (d, J = 9.4 Hz, 1H, H-4), 3.36 – 3.31 (m, 1H, H-3), 3.20 – 3.09 (m, 2H, H-2, H-5ax.).

^{13}C NMR (126 MHz, MeOH-d_4) δ 182.77 (1C, C=S), 171.28 (1C, C=O), 159.96 (1C, ArC), 154.52 (2C, ArC-fluorescein), 144.58 (1C, <u>C</u>=CH-triazole), 142.29 (1C, ArC-fluorescein), 136.60 (2C, ArCH), 130.54 (4C, ArCH-fluorescein), 126.24 (1C, C=<u>C</u>H-triazole), 126.15 (1C, ArCH-fluorescein), 125.01 (1C, ArC), 120.14 (1C, ArCH-fluorescein), 116.34 (2C, ArCH), 114.20 (1C, ArCH-fluorescein), 111.84 (1C, ArC-fluorescein), 103.56 (2C, ArCH-fluorescein), 90.53 (1C, C-1), 79.30 (1C, C-3), 73.52 (1C, C-2), 71.55 (1C, CH_2), 71.42 (2C, CH_2), 71.32 (1C, CH_2), 70.92 (1C, C-4), 70.56 (1C, C-), 70.27 (1C, CH_2), 62.42 (1C, CH_2), 51.48 (1C, CH_2), 45.49 (1C, CH_2).

HPLC-MS: $[C_{43}H_{44}N_5O_{13}S_2 + H]^+$ calcd. 904.25, found. 904.33.

HRMS: $[C_{43}H_{44}N_5O_{13}S_2 + H]^+$ calcd. 904.2529, found 904.2519.

Galactoside side product S27*m*

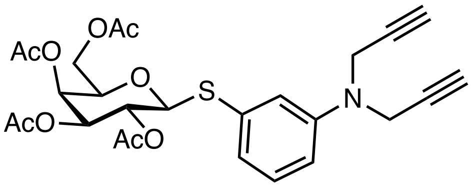

Compound **S27*m*** (19.2 mg, 36.1 µmol, 5%) was isolated as side product during the synthesis of compound **S3*m***.

^{1}H NMR (300 MHz, DMSO-d$_6$) δ 7.22 (t, *J* = 8.0 Hz, 1H, ArH), 6.94 (t, *J* = 2.0 Hz, 1H, ArH), 6.91 – 6.82 (m, 2H, ArH), 5.36 – 5.31 (m, 1H, H-4), 5.30 – 5.22 (m, 2H, H-1, H-3), 5.04 (t, *J* = 9.9 Hz, 1H, H-2), 4.39 – 4.30 (m, 1H, H-5), 4.16 (d, *J* = 2.4 Hz, 4H, CH$_2$), 4.07 (d, *J* = 6.3 Hz, 2H, H-6), 3.19 – 3.15 (m, 2H, C≡CH), 2.12 (s, 3H, CH$_3$), 2.05 (s, 3H, CH$_3$), 1.99 (s, 3H, CH$_3$), 1.92 (s, 3H, CH$_3$).

^{13}C NMR (75 MHz, DMSO-d$_6$) δ 169.96 (1C, C=O), 169.87 (1C, C=O), 169.46 (1C, C=O), 169.29 (1C, C=O), 147.61 (1C, ArC), 133.77 (1C, ArC), 129.40 (1C, ArCH), 120.00 (1C, ArCH), 115.99 (1C, ArCH), 113.94 (1C, ArCH), 84.36 (1C, C-1), 79.65, 75.16, 73.43 (1C, C-5), 71.02 (1C, C-3), 67.54 (1C, C-2), 67.00 (1C, C-4), 61.64 (1C, C-6), 20.57 (1C, CH$_3$), 20.49 (1C, CH$_3$), 20.40 (1C, CH$_3$), 20.34 (1C, CH$_3$).

HPLC-MS: [$C_{26}H_{29}NO_9S$ + H]$^+$ calcd. 532.16, found. 532.19.

HRMS: [$C_{26}H_{29}NO_9S$ + H]$^+$ calcd. 532.1636, found. 532.1628.

Galactoside side product S27*p*

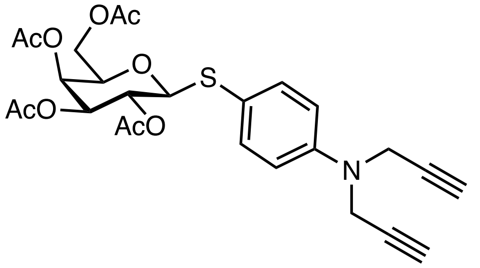

Compound **S27*p*** (16.5 mg, 31.0 µmol, 13%) was isolated as side product during the synthesis of compound **S3*p***.

^{1}H NMR (300 MHz, DMSO-d$_6$) δ 7.39 – 7.31 (m, 2H ArH), 6.93 – 6.86 (m, 2H, ArH), 5.27 (d, *J* = 3.4 Hz, 1H, H-4), 5.18 (tt, *J* = 7.8, 3.4 Hz, 1H, H-3), 5.03 – 4.90 (m, 2H, H-1, H-2), 4.28 – 4.13 (m, 5H, H-5, CH$_2$), 4.13 – 3.95 (m, 2H, H-6), 3.18 – 3.14 (m, 2H, C≡CH), 2.09 (s, 3H, CH$_3$), 2.07 (s, 3H, CH$_3$), 2.01 (s, 3H, CH$_3$), 1.91 (s, 3H, CH$_3$).

^{13}C NMR (75 MHz, DMSO-d$_6$) δ 169.94 (1C, C=O), 169.88 (1C, C=O), 169.44 (1C, C=O), 169.23 (1C, C=O), 147.19 (1C, ArC), 133.97 (2C, ArCH), 119.44 (1C, ArC), 114.85 (2C, ArCH), 85.37 (1C, C-1), 79.72 (2C, <u>C</u>≡CH), 75.03 (2C, C≡<u>C</u>H), 73.31 (1C, C-5), 71.13 (1C, C-3), 67.58 (1C, C-2), 67.22 (1C, C-4), 61.70 (1C, C-6), 20.60 (2C, CH$_3$), 20.44 (1C, CH$_3$), 20.35 (1C, CH$_3$).

HPLC-MS: [$C_{26}H_{29}NO_9S$ + H]$^+$ calcd. 532.16, found. 532.17.

HRMS: [$C_{26}H_{29}NO_9S$ + H]$^+$ calcd. 532.1636, found. 532.1632.

Galactoside side product S28*p*

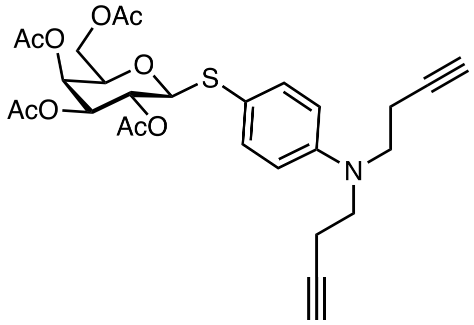

Compound **S28*p*** (23.3 mg, 41.6 µmol, 6%) was isolated as side product during the synthesis of compound **S4*p***.

^{1}H NMR (300 MHz, DMSO-d$_6$) δ 7.35 – 7.25 (m, 2H ArH), 6.71 – 6.60 (m, 2H, ArH), 5.26 (dd, *J* = 3.4, 1.0 Hz, 1H, H-4), 5.19 (dd, *J* = 9.7, 3.5 Hz, 1H, H-3), 4.96 (t, *J* = 9.8 Hz, 1H, H-2), 4.84 (d, *J* = 10.0 Hz, 1H, H-1), 4.23 – 4.14 (m, 1H, H-5), 4.12 – 3.95 (m, 2H, H-6), 3.54 (t, *J* = 7.1 Hz, 4H, NHC$\underline{H}_2$CH$_2$), 2.87 (t, *J* = 2.6 Hz, 2H, C≡CH), 2.40 (td, *J* = 7.1, 2.6 Hz, 4H, NHCH$_2$C$\underline{H}_2$), 2.08 (s, 3H, CH$_3$), 2.07 (s, 3H, CH$_3$), 2.00 (s, 3H, CH$_3$), 1.91 (s, 3H, CH$_3$).

^{13}C NMR (126 MHz, DMSO-d$_6$) δ 169.96 (1C, C=O), 169.89 (1C, C=O), 169.51 (1C, C=O), 169.26 (1C, C=O), 146.80 (1C, ArC), 135.32 (2C, ArCH), 116.19 (1C, ArC), 111.85 (2C, ArCH), 85.91 (1C, C-1), 82.35 (2C, $\underline{C}$≡CH), 73.23 (1C, C-5), 72.61 (2C, C≡$\underline{C}$H), 71.15 (1C, C-3), 67.57 (1C, C-2), 67.26 (1C, C-4), 61.64 (1C, C-6), 49.23 (2C, NH$\underline{C}$H$_2$CH$_2$), 20.67 (1C, CH$_3$), 20.54 (1C, CH$_3$), 20.42 (1C, CH$_3$), 20.40 (1C, CH$_3$), 16.50 (2C, NHCH$_2\underline{C}$H$_2$).

HPLC-MS: [$C_{28}H_{33}NO_9S$ + H]$^+$ calcd. 560.19, found. 560.24.

HRMS: [$C_{28}H_{33}NO_9S$ + H]$^+$ calcd. 560.1949, found. 560.1945.

Galactoside side product S29*p*

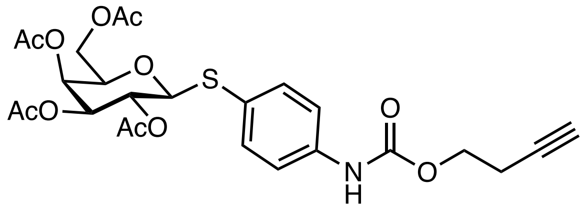

Compound **S29*p*** (15.3 mg, 27.7 µmol, 4%) was isolated as side product during the synthesis of compound **S4*p***.

^{1}H NMR (300 MHz, DMSO-d$_6$) δ 9.87 (s, 1H, NH), 7.51 – 7.44 (m, 2H, ArH), 7.42 – 7.35 (m, 2H, ArH), 5.29 (dd, *J* = 3.5, 1.0 Hz, 1H, H-4), 5.22 (dd, *J* = 9.4, 3.5 Hz, 1H, H-3), 5.08 – 4.92 (m, 2H, H-1, H-2), 4.29 – 4.21 (m, 1H, H-5), 4.15 (t, *J* = 6.4 Hz, 2H, NH$\underline{C}$H$_2$CH$_2$), 4.11 – 3.97 (m, 2H, H-6), 2.90 (t, *J* = 2.6 Hz, 1H, C≡CH), 2.56 (td, *J* = 6.4, 2.7 Hz, 2H, NHCH$_2$C$\underline{H}_2$), 2.10 (s, 3H, CH$_3$), 2.05 (s, 3H, CH$_3$), 2.00 (s, 3H, CH$_3$), 1.91 (s, 3H, CH$_3$).

^{13}C NMR (126 MHz, DMSO-d$_6$) δ 169.95 (1C, C=O), 169.88 (1C, C=O), 169.49 (1C, C=O), 169.26 (1C, C=O), 153.17 (1C, NHC=O), 139.13 (1C, ArC), 132.81 (2C, ArCH), 124.80 (1C, ArC), 118.53 (2C, ArCH), 84.73 (1C, C-1), 81.17 (1C, $\underline{C}$≡CH), 73.44 (1C, C-5), 72.67 (1C, C≡$\underline{C}$H), 71.10 (1C, C-3), 67.59 (1C, C-2), 67.04 (1C, C-4), 62.42 (1C, COO$\underline{C}$H$_2$CH$_2$), 61.78 (1C, C-5), 20.60 (1C, CH$_3$), 20.53 (1C, CH$_3$), 20.42 (1C, CH$_3$), 20.38 (1C, CH$_3$), 18.71 (1C, COOCH$_2\underline{C}$H$_2$).

HPLC-MS: [$C_{25}H_{29}NO_{11}S$ + H]$^+$ calcd. 552.15, found. 552.08.

HRMS: [$C_{25}H_{29}NO_{11}S$ + NH_4]$^+$ calcd. 569.1800, found. 569.1795.

4-(Dichloromethyl)-N-(prop-2-yn-1-yl)benzamide (S33)

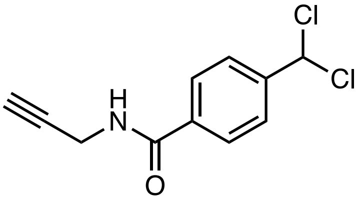

4-Formylbenzoic acid (**S32**, 283.0 mg, 1.89 mmol) was dissolved in thionyl chloride (6 mL, 82.21 mmol). The reaction was stirred at 80 °C for 20 h. The solvent was removed *in vacuo*. Crude 4-(dichloromethyl)benzoyl chloride intermediate (98.0 mg) was dissolved in dichloromethane (2 mL). The reaction was cooled to 0 °C and triethyl amine (183 µL, 1.32 mmol) and propargyl amine (56 µL, 0.88 mmol) were added dropwise. The mixture was allowed to warm to room temperature and stirred for 3 h. The reaction was poured over water and extracted with dichloromethane. Combined organic phases were washed with satd. $NaHCO_3$ and brine, dried over anhydrous Na_2SO_4, filtered and concentrated *in vacuo*. The product was purified by normal phase MPLC (petrol ether/ethyl acetate, 5–60% ethyl acetate). Compound **S33** was obtained as a white solid (17.2 mg, 0.07 mmol, 16% over two steps).
^{1}H NMR (500 MHz, $CDCl_3$) δ 7.82 (d, *J* = 8.1 Hz, 2H, ArH), 7.65 (d, *J* = 8.1 Hz, 2H, ArH), 6.73 (s, 1H, $CHCl_2$), 6.33 (s, 1H, NH), 4.27 (dd, *J* = 5.0, 2.3, 2H, CH_2), 2.30 (s, 1H, C≡CH).
^{13}C NMR (126 MHz, $CDCl_3$) δ 166.21 (C=O), 143.69 (ArC), 135.22 (ArC), 127.74 (2C, ArCH), 126.69 (2C, ArCH), 79.30 (<u>C</u>≡CH), 72.31 (C≡<u>C</u>H), 70.89 ($CHCl_2$), 30.04 (CH_2).
HPLC-MS: $[C_{11}H_9Cl_2NO + H]^+$ calcd. 242.01, found 241.89.

4-(Bis(4-formylphenoxy)methyl)-N-(prop-2-yn-1-yl)benzamide (S34)

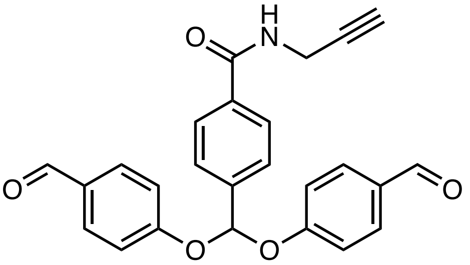

Compound **S33** (35.9 mg, 0.15 mmol), 4-hydroxybenzaldehyde (106 mg, 1.59 mmol) and potassium carbonate (91.5 mg, 0.66 mmol) were dissolved in dry dimethylformamide (5 mL), heated to 80 °C and stirred for 44 h. After cooling down to room temperature, the 4-hydroxybenzaldehyde (72.5 mg, 0.65 mmol) and potassium carbonate (92.1 mg, 0.67 mmol) were added. The reaction was heated to 80 °C and stirred for additional 24 h. After cooling down to room temperature, the reaction was diluted with ethyl acetate and washed with water, satd. $NaHCO_3$ and brine, dried over anhydrous Na_2SO_4, filtered and concentrated *in vacuo*. The product was purified by normal phase MPLC (petrol ether/ethyl acetate, 10–70% ethyl acetate). **S34** was obtained as a white solid (41.2 mg, 0.10 mmol, 67%).
^{1}H NMR (500 MHz, $CDCl_3$) δ 9.89 (s, 2H, CHO), 7.86 (d, *J* = 7.8 Hz, 2H, ArH), 7.81 (d, *J* = 8.4 Hz, 4H, ArH), 7.70 (d, *J* = 8.0 Hz, 2H, ArH), 7.13 (d, *J* = 8.3 Hz, 4H, ArH), 6.93 (s, 1H, CH), 6.41 (s, 1H, NH), 4.25 (dd, *J* = 4.6, 2.1 Hz, 2H, CH_2), 2.28 (s, 1H, C≡CH).

^{13}C NMR (126 MHz, $CDCl_3$) δ 190.87 (O=CH), 166.51 (C=O), 160.20 (ArC), 139.22 (ArC), 135.24 (ArC), 132.05 (4C, ArCH), 131.74 (ArC), 127.85 (2C, ArCH), 127.16 (2C, ArCH), 117.58 (4C, ArCH), 98.92 (CH), 79.26 (C≡CH), 72.29 (C≡CH), 30.04 (CH_2).
HPLC-MS: $[C_{25}H_{19}NO_5 + H]^+$ calcd. 414.13, found 414.10.
HRMS: $[C_{25}H_{19}NO_5 + H]^+$ calcd. 414.1336, found 414.1333.

Synthesis of galactoside building block **S35** is described in Zahorska *et. al.*[2]

Divalent ligand S36

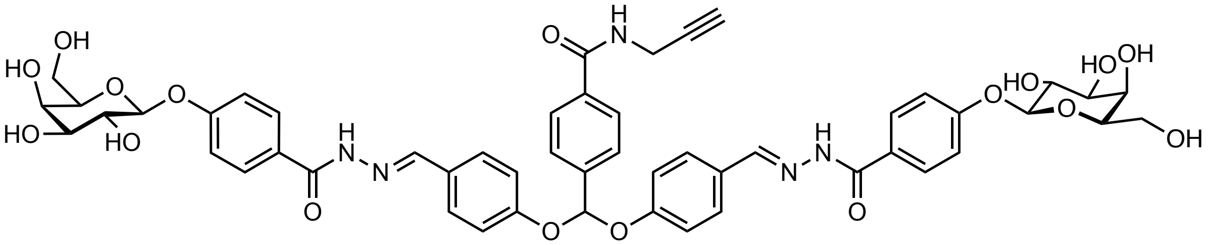

Compound **S34** (15.1 mg, 36.5 µmol) and hydrazide **S35**[2] (51.6 mg, 164 µmol) were dissolved in 1.5 mL dimethyl sulfoxide and 20 µL of formic acid was added. After 2.5 h, the reaction was lyophilized. The compound **S36** was purified by preparative HPLC (water/acetonitrile, gradient of 15–40% acetonitrile). Compound **S36** (32.3 mg, 32.1 µmol, 83%) was obtained as a white solid.
^1H NMR (500 MHz, DMSO-d_6) δ 11.70 (s, 2H, N-NH), 9.03 (t, J = 5.6 Hz, 1H, CONH), 8.38 (s, 2H, N=CH), 7.94 (d, J = 8.1 Hz, 2H, ArH), 7.88 (d, J = 8.4 Hz, 4H, ArH), 7.80 (d, J = 8.0 Hz, 2H, ArH), 7.68 (d, J = 8.3 Hz, 4H, ArH), 7.37 (s, 1H, CH), 7.23 – 7.03 (m, 8H, ArH), 5.25 (s, 2H, OH-2), 5.03 – 4.85 (m, 4H, H-1, OH-3), 4.70 (s, 2H, OH-6), 4.57 (s, 2H, OH-4), 4.06 (dd, J = 5.6, 2.6 Hz, 2H, CH_2), 3.72 (d, J = 3.3 Hz, 2H, H-4), 3.67 – 3.47 (m, 8H, H-2, H-5, H-6), 3.47 – 3.41 (m, 2H, H-3), 3.14 (t, J = 2.5 Hz, 1H, C≡CH).
^{13}C NMR (126 MHz, DMSO-d_6) δ 165.59 (1C, C=O), 162.48 (2C, C=O), 160.09 (2C, ArC), 156.85 (2C, ArC), 146.82 (2C, N=CH), 139.73 (1C, ArC), 134.93 (1C, ArC), 129.40 (4C, ArCH), 128.92 (2C, ArC), 128.71 (4C, ArCH), 127.75 (2C, ArCH), 127.01 (2C, ArCH), 126.56 (2C, ArC), 117.26 (4C, ArCH), 115.81 (4C, ArCH), 100.45 (2C, C-1), 98.09 (1C, CH), 81.27 (1C, C≡CH), 75.65 (2C, C-5), 73.32 (2C, C-3), 73.06 (1C, C≡CH), 70.25 (2C, C-2), 68.18 (2C, C-4), 60.40 (2C, C-6), 28.61 (1C, CH_2).
HPLC-MS: $[C_{51}H_{51}N_5O_{17} + H]^+$ calcd. 1006.34, found 1006.44.
HRMS: $[C_{51}H_{51}N_5O_{17} + H]^+$ calcd. 1006.3353, found 1006.3359.

Bis-benzaldehyde fluorescent linker S37

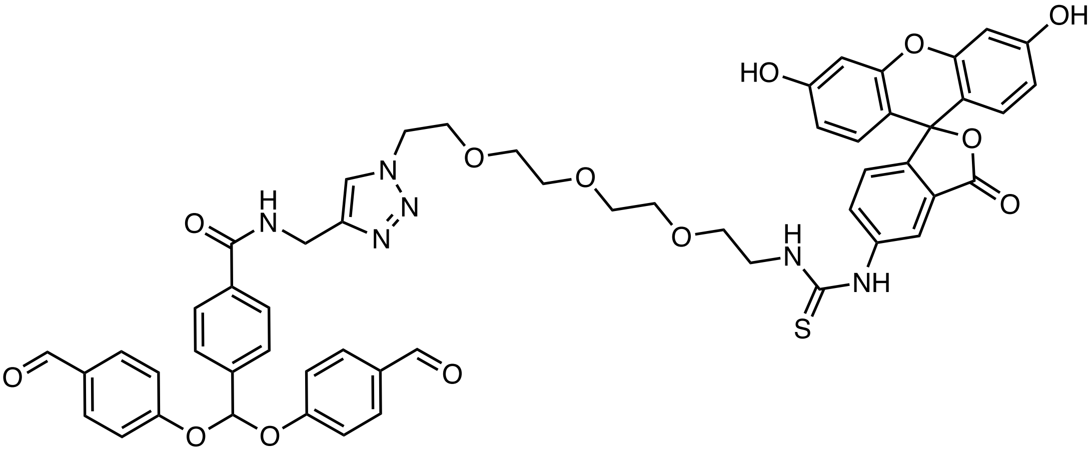

Azide modified fluorescein (**9**, 16.9 mg, 27.8 µmol) and bis-benzaldehyde **S34** (10.3 mg, 24.9 µmol) were dissolved in dimethylformamide (1.5 mL). $CuSO_4$ solution (75 µL, 100 mM in water, 7.5 µmol) and sodium ascorbate solution (75 µL, 100 mM in water, 7.5 µmol) were added. The reaction was stirred at r.t. for 2 days and then dried *in vacuo*. The product **S37** was purified by preparative HPLC (water/acetonitrile with 0.1% formic acid, gradient of 30–60% acetonitrile). Compound **S37** was obtained as a yellow solid (16.7 mg, 16.4 µmol, 66%).

^{1}H NMR (500 MHz, DMSO-d$_6$) δ 10.14 (s, 2H, OH-fluorescein), 10.05 (s, 1H, NH-thiourea), 9.87 (s, 2H, CHO), 9.11 (t, *J* = 5.7 Hz, 1H, NH-thiourea), 8.27 (s, 1H, ArH-fluorescein), 8.10 (s, 1H, CONH), 7.98 – 7.91 (m, 3H, ArH, CH-triazole), 7.87 (d, *J* = 8.7 Hz, 4H, ArH), 7.79 (d, *J* = 8.3 Hz, 2H, ArH), 7.73 (d, *J* = 7.8 Hz, 1H, ArH-fluorescein), 7.57 (s, 1H, CH), 7.25 (d, *J* = 8.7 Hz, 4H, ArH), 7.17 (d, *J* = 8.3 Hz, 1H, ArH-fluorescein), 6.67 (d, *J* = 2.2 Hz, 2H, ArH-fluorescein), 6.62 – 6.53 (m, 4H, ArH-fluorescein), 4.53 – 4.44 (m, 4H, CH_2), 3.78 (t, *J* = 5.2 Hz, 2H, CH_2), 3.67 (s, 2H, CH_2), 3.57 (t, *J* = 5.4 Hz, 2H, CH_2), 3.54 – 3.44 (m, 8H, CH_2).

^{13}C NMR (126 MHz, DMSO-d$_6$) δ 191.50 (2C, O=CH), 180.53 (1C, C=S), 168.55 (1C, C=O, fluorescein), 165.60 (1C, C=O), 159.96 (2C, ArC), 159.51 (2C, ArC-fluorescein), 151.90 (2C, ArC-fluorescein), 147.16 (2C, ArC-fluorescein), 144.86 (1C, <u>C</u>=CH-triazole), 141.33 (1C, ArC-fluorescein), 138.66 (1C, ArC), 135.48 (1C, ArC), 131.81 (4C, ArCH), 131.10 (2C, ArC) 129.44 (1C, ArC-fluorescein), 129.08 (4C, ArCH-fluorescein), 127.81 (2C, ArCH), 126.91 (2C, ArCH), 126.58 (1C, ArC-fluorescein), 124.11 (1C, ArCH-fluorescein), 123.33 (1C, C=<u>C</u>H-triazole), 117.01 (4C, ArCH), 116.37 (1C, ArCH-fluorescein), 112.61 (1C, ArCH-fluorescein), 109.72 (2C, ArC-fluorescein), 102.25 (2C, ArCH-fluorescein), 97.59 (1C, CH), 69.73 (1C, CH_2), 69.64 (2C, CH_2), 69.54 (1C, CH_2), 68.75 (1C, CH_2), 68.41 (1C, CH_2), 49.29 (1C, CH_2), 43.71 (1C, CH_2), 34.90 (1C, CH_2).

HPLC-MS: $[C_{54}H_{48}N_6O_{13}S + 2H]^{2+}$ calcd. 511.16, found 511.15.

HRMS: $[C_{54}H_{48}N_6O_{13}S + 2H]^{2+}$ calcd. 511.1573, found 511.1572.

Divalent fluorescent ligand S31

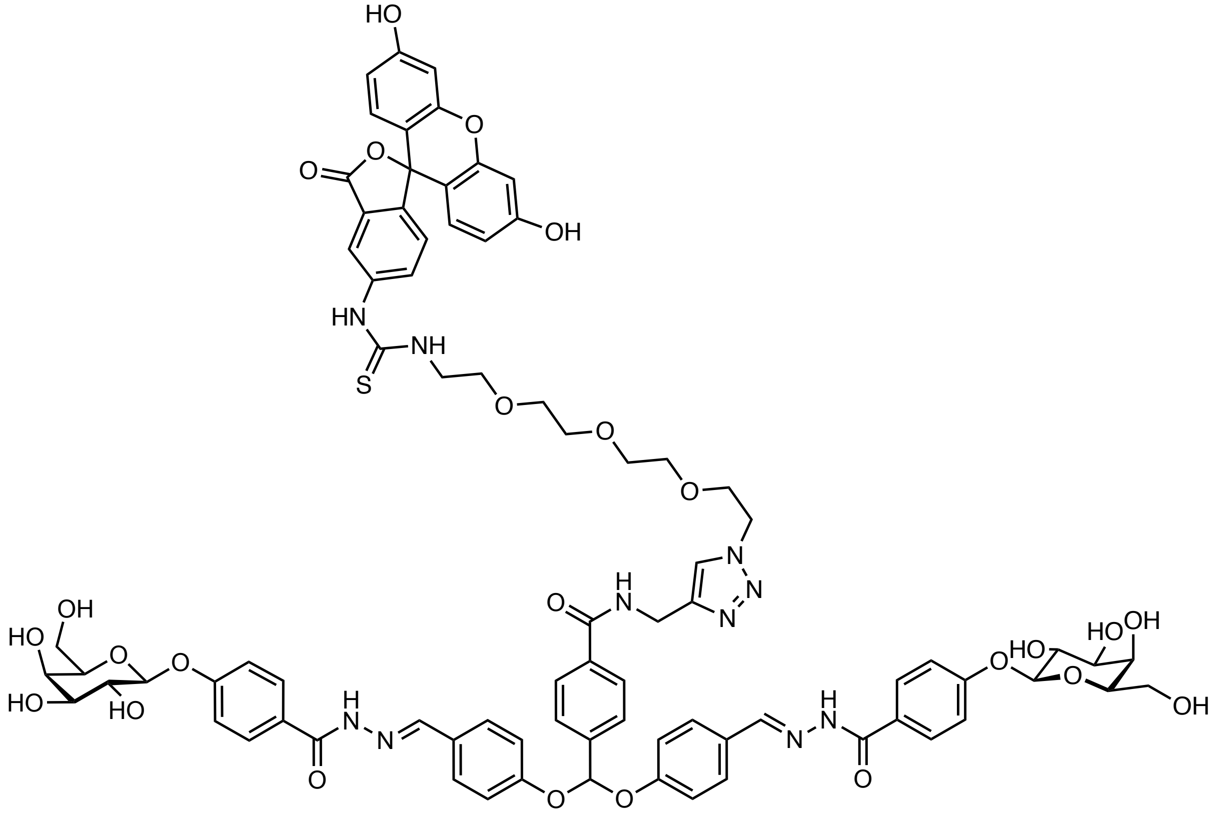

Compound **S37** (4.4 mg, 4.3 µmol) and hydrazide **S35**[2] (5.8 mg, 18.5 µmol) were dissolved in 450 µL dimethyl sulfoxide and 40 µL of formic acid was added. After 7.5 h, the reaction was diluted with water and lyophilized. Purification by preparative HPLC (water/acetonitrile with 0.1% formic acid, gradient of 15–40% acetonitrile) gave product **S31** (2.9 mg, 1.5 µmol, 35%, ≈90% purity) as a yellow powder.
HPLC-MS: $[C_{80}H_{80}N_{10}O_{25}S + 2H]^{2+}$ calcd. 807.26, found 807.78.
HRMS: $[C_{80}H_{80}N_{10}O_{25}S + 2H]^{2+}$ calcd. 807.2581, found 807.2589.
Monohydrolyzed product (impurity) HPLC-MS: $[C_{67}H_{64}N_8O_{19}S + 2H]^{2+}$ calcd. 659.21, found 659.30.

***N*,*N*-bis(4-nitrobenzyl)but-3-yn-1-amine (11)**

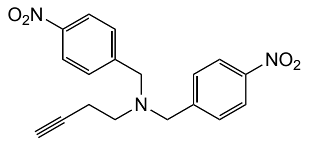

But-3-yn-1-amine hydrochloride (**10**, 55.2 mg, 0.55 mmol), 4-nitrobenzyl bromide (327 mg, 1.52 mmol) and potassium carbonate (264 mg, 1.91 mmol) were suspended in DMF (4 mL) and stirred at room temperature overnight. The reaction was diluted with dichloromethane and water, organic phase was washed with satd. aqueous $NaHCO_3$ and half satd. brine, dried over anhydrous Na_2SO_4, filtered and concentrated *in vacuo*. The product **11** (134 mg, 0.39 mmol, 75%) was purified by normal phase MPLC (petrol ether/ethyl acetate, 5-35% ethyl acetate).
^{1}H NMR (500 MHz, Acetone-d_6) δ 8.24 – 8.16 (m, 4H, ArCH), 7.76 (d, J = 8.7 Hz, 4H, ArCH), 3.85 (s, 4H, Ar-CH$_2$), 2.73 (td, J = 7.2, 1.4 Hz, 2H, CH$_2$C$\underline{H}_2$N), 2.52 – 2.45 (m, 2H, C$\underline{H}_2$CH$_2$N), 2.43 (t, J = 2.7 Hz, 1H, C≡CH).

^{13}C NMR (126 MHz, Acetone-d_6) δ 148.49 (2C, ArC), 148.07 (2C, ArC), 130.45 (4C, ArCH), 124.19 (4C, ArCH), 83.26 (1C, $\underline{C}$≡CH), 71.06 (1C, C≡$\underline{C}$H), 58.04 (2C, Ar-CH_2), 53.19 (1C, $CH_2\underline{C}H_2N$), 17.40 (1C, $\underline{C}H_2CH_2N$).
HPLC-MS: $[C_{18}H_{17}N_3O_4 + H]^+$ calcd. 340.13, found 340.15.
HRMS: $[C_{18}H_{17}N_3O_4 + H]^+$ calcd. 340.1292, found 340.1289.

***N,N*-bis(4-aminobenzyl)but-3-yn-1-amine (12)**

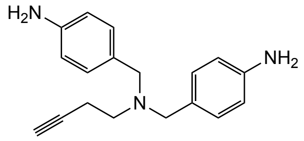

N,N-bis(4-nitrobenzyl)but-3-yn-1-amine (**11**, 21.8 mg, 64.2 µmol), iron powder (17.7 mg, 317 µmol) and $CaCl_2$ (22.4 mg, 202 µmol) were suspended in EtOH/H_2O mixture (0.8 mL : 0.2 mL) under argon atmosphere. The reaction was stirred at r.t. for 1 day, heated to 40 °C for 2 days and then stirred at r.t. for 1 week. The iron was filtered off and the reaction was dried *in vacuo*. The product **12** was purified by normal phase MPLC (petrol ether/ethanol with 2% NH_3OH, 10-50% ethanol). The product **12** (12.5 mg, 44.7 µmol, 70%, ~90% pure) was obtained as yellow powder.
^{1}H NMR (500 MHz, MeOD) δ 7.08 (d, J = 8.0 Hz, 4H, ArH), 6.70 (d, J = 7.9 Hz, 4H, ArH), 3.47 (s, 4H, Ar-CH_2N), 2.60 (t, J = 7.6 Hz, 2H, CH≡$CCH_2C\underline{H_2}N$), 2.33 – 2.27 (m, 2H, CH≡$C\underline{CH_2}CH_2N$), 2.19 (t, J = 2.7 Hz, 1H, CH≡C).
^{13}C NMR (126 MHz, MeOD) δ 147.61 (2C, ArC), 131.12 (2C, ArCH), 129.44 (2C, ArC), 116.56 (2C, ArCH), 83.46 (1C, CH≡$\underline{C}$), 70.27 (1C, $\underline{C}$H≡C), 58.38 (2C, Ar-$\underline{C}H_2N$), 52.61 (1C, CH≡$CCH_2\underline{C}H_2N$), 17.13 (1C, CH≡$C\underline{C}H_2CH_2N$).
HPLC-MS: $[C_{18}H_{21}N_3 + H]^+$ calcd. 280.18, found 280.13.
HRMS: $[C_{18}H_{21}N_3 + H]^+$ calcd. 280.1808, found 280.1804.

Compounds **14** and **15** were synthesised as reported in Zahorska *et. al.*.[16]

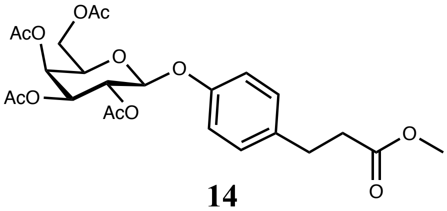

14

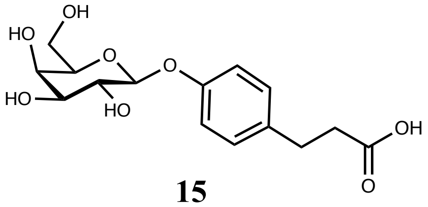

15

Divalent ligand 16

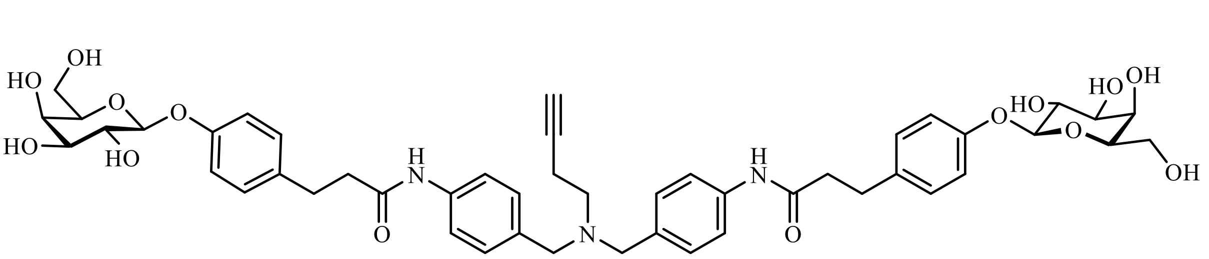

Bis-aniline linker **12** (26.7 mg, 95.6 µmol), galactoside **15** (71.7 mg, 218 µmol, in two portion over 2 days), HBTU (89.9 mg, 237 µmol, in two portion over 2 days) were dissolved in dimethylformamide (2 mL) and DIPEA (70 µL, 402 µmol) was added. Reaction was stirred at r.t. for 2 days, then dried *in vacuo*. The product **16** was purified by C18 column preparative

HPLC chromatography (water/acetonitrile supplemented with 1% formic acid, gradient of 10-40% acetonitrile). Compound **16** (14.6 mg, 16.2 µmol, 17%) was obtained as a white solid.
^{1}H NMR (500 MHz, DMSO) δ 9.90 (s, 2H, NH), 7.52 (d, *J* = 8.5 Hz, 4H, ArH), 7.26 (d, *J* = 8.4 Hz, 4H, ArH), 7.18 – 7.12 (m, 4H, ArH), 6.97 – 6.90 (m, 4H, ArH), 5.15 (s, 2H, OH-2), 4.87 (s, 2H, OH-3), 4.76 (d, *J* = 7.7 Hz, 2H, H-1), 4.66 (s, 2H, OH-6), 4.50 (s, 2H, OH-4), 3.67 (s, 2H, H-4), 3.58 – 3.41 (m, 14H, H-2, H-3, H-5, H-6, Ar-CH_2N), 2.84 (t, *J* = 7.7 Hz, 4H C<u>H</u>$_2$CH$_2$CONH), 2.77 (t, *J* = 2.6 Hz, 1H, CH≡C), 2.57 (t, *J* = 7.7 Hz, 4H, CH$_2$C<u>H</u>$_2$CONH), 2.53 – 2.51 (m, 2H, CH≡CC<u>H</u>$_2$CH$_2$N), 2.35 – 2.29 (m, *J* = 7.5, 2.7 Hz, 2H, CH≡CC<u>H</u>$_2$CH$_2$N).
^{13}C NMR (126 MHz, DMSO) δ 170.39 (2C, C=O), 155.88 (2C, ArC), 138.07 (2C, ArC), 134.38 (2C, ArC), 133.75 (2C, ArC), 129.12 (4C, ArCH), 128.92 (4C, ArCH), 118.95 (4C, ArCH), 116.21 (4C, ArCH), 101.12 (2C, C-1), 83.26 (1C, CH≡<u>C</u>), 75.48 (2C, C-5), 73.33 (2C, C-3), 71.99 (1C, <u>C</u>H≡C), 70.32 (2C, C-2), 68.19 (2C, C-4), 60.42 (2C, C-6), 56.51 (2C, Ar-<u>C</u>H$_2$N), 51.10 (1C, CH≡CCH$_2$<u>C</u>H$_2$N), 38.29 (2C, CH$_2$<u>C</u>H$_2$CONH), 30.12 (2C, <u>C</u>H$_2$CH$_2$CONH), 15.95 (1C, CH≡C<u>C</u>H$_2$CH$_2$N).
HPLC-MS: $[C_{48}H_{57}N_3O_{14} + H]^+$ calcd. 900.39, found 900.34.
HRMS: $[C_{48}H_{57}N_3O_{14} + H]^+$ calcd. 900.3913, found 900.3911.

Divalent fluorescent ligand 17

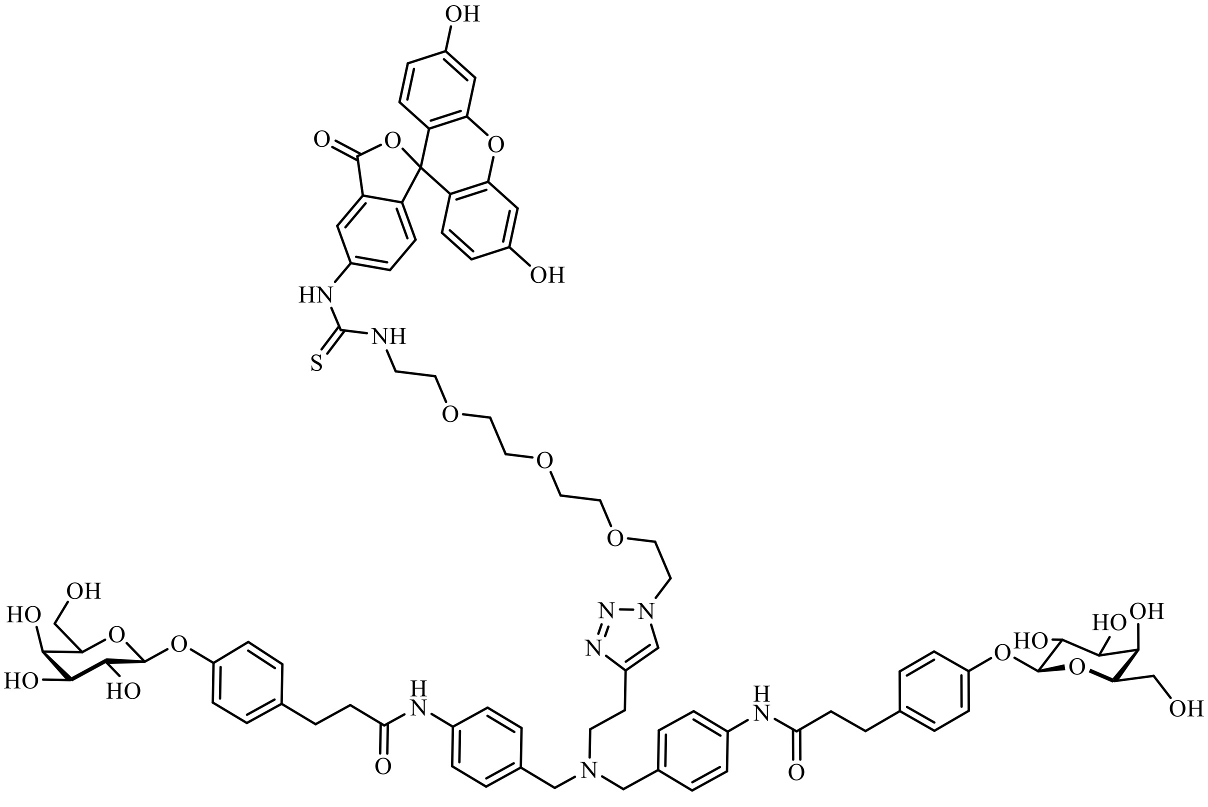

Azide modified FITC (**9**, 4.6 mg, 7.6 µmol) and divalent alkyne precursor **16** (4.44 mg, 4.9 µmol) were dissolved in DMF (500 µL). $CuSO_4$ solution (30 µL, 100 mM in H_2O, 3 µmol) and sodium ascorbate solution (100 µL, 100 mM in H_2O, 10 µmol) were added. The reaction was stirred at r.t. for 5 days then warmed up to 35 °C for one day. After lyophilization, the product was purified by preparative HPLC (water/acetonitrile with 1% formic acid, 20-40% acetonitrile). The product **16** was obtained as yellow solid (4 mg, 2.7 µmol, 54%).
^{1}H NMR (500 MHz, DMSO) δ 10.21 (s, 2H, NH-thiourea), 9.86 (s, 2H, NHCO), 8.37 – 8.21 (m, 2H, ArH-fluorescein, NH-thiourea), 7.75 (d, *J* = 8.3 Hz, 1H, ArH-fluorescein), 7.70 (s, 1H,

CH-triazole), 7.51 (d, J = 8.3 Hz, 4H. ArH), 7.21 (d, J = 8.2 Hz, 4H, ArH), 7.17 (s, 1H, ArH-fluorescein), 7.14 (d, J = 8.8 Hz, 4H, ArH), 6.96 – 6.90 (m, 4H ArH), 6.67 (d, J = 2.3 Hz, 2H, ArH-fluorescein), 6.62 – 6.53 (m, 4H, ArH-fluorescein), 5.11 (s, 2H OH-2), 4.83 (s, 2H, OH-3), 4.75 (d, J = 7.6 Hz, 2H, H-1), 4.63 (s, 2H, OH-6), 4.52 – 4.45 (m, 2H OH-4), 4.43 (t, J = 5.3 Hz, 2H, CH_2-PEG), 3.76 (t, J = 5.3 Hz, 2H, CH_2-PEG), 3.72 – 3.64 (s, 4H, H-4, CH_2-PEG), 3.61 – 3.38 (m, 31H, H-2, H-3, H-5, H-6, Ar-$\underline{C}H_2N$, CH_2-PEG), 2.88 – 2.74 (m, 6H, $C\underline{H}_2CH_2CONH$, triazole-$C\underline{H}_2CH_2N$), 2.62 – 2.53 (m, 6H, $CH_2C\underline{H}_2CONH$, triazole-$CH_2C\underline{H}_2N$). ^{13}C NMR (126 MHz, DMSO) δ 180.56(1C, C=S), 170.30 (2C, NHC=O), 168.55 (1C, C=O), 159.57 (2C, ArC-fluorescein), 155.84 (2C, ArC), 151.91 (2C, ArC-fluorescein), 144.97 (1C, $\underline{C}$=CH-triazole), 141.43 (1C, ArC-fluorescein), 137.96 (2C, ArC), 134.35 (2C, ArC), 133.81 (2C, ArC), 129.04 (6C, ArCH, ArCH-fluorescein), 128.85 (4C, ArCH), 124.06 (1C, ArCH-fluorescein), 122.38 (1C, C=$\underline{C}$H-triazole), 118.87 (4C, ArCH), 116.19 (5C, ArCH, ArC-fluorescein), 112.64 (1C, ArCH-fluorescein), 109.74 (1C, ArC-fluorescein), 102.24 (2C, ArCH-fluorescein), 101.13 (2C, C-1), 75.44 (2C, C-5), 73.31 (2C, C-3), 70.30 (2C, C-2), 69.72 (1C, CH_2-PEG), 69.64 (2C, CH_2-PEG), 69.55 (1C, CH_2-PEG), 68.81 (1C, CH_2-PEG), 68.41 (1C, CH_2-PEG), 68.15 (2C, C-4), 60.39 (2C, C-6), 56.74 (2C, Ar-$\underline{C}H_2N$), 52.40 (1C, triazole-$CH_2\underline{C}H_2N$), 49.16 (1C, CH_2-PEG), 43.67 (1C, CH_2-PEG), 38.24 (2C, $CH_2\underline{C}H_2CONH$), 30.08 (2C, $\underline{C}H_2CH_2CONH$), 22.87 (1C, triazole-$CH_2\underline{C}H_2N$).
HPLC-MS: $[C_{77}H_{86}N_8O_{22}S + 2H]^{2+}$ calcd. 754.2861, found 754.72.
HRMS: $[C_{77}H_{86}N_8O_{22}S + 2H]^{2+}$ calcd. 754.2861, found 754.2836.

Divalent fluorescent ligand 19

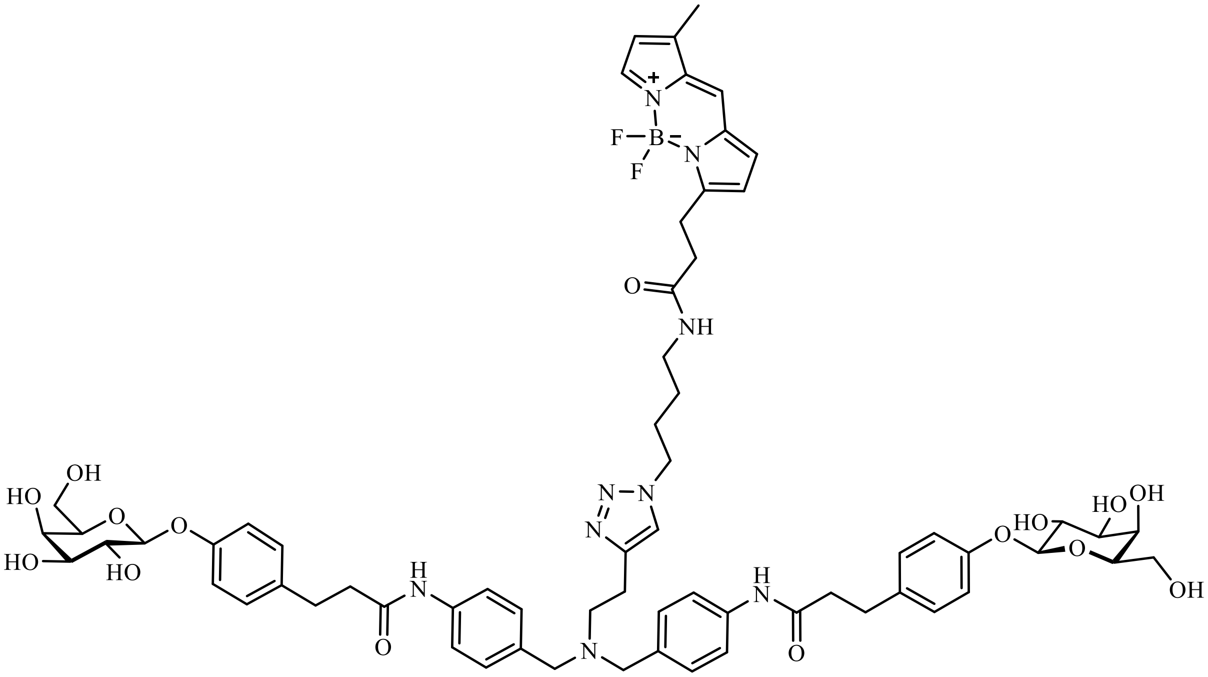

Divalent alkyne precursor **16** (6.5 mg, 7.2 µmol) was dissolved in DMF (1.5 mL). BDP FL azide solution (**18**, 80 µL, 100 mM in DMF, 8.0 µmol), $CuSO_4$ solution (120 µL, 100 mM in H_2O, 12 µmol), sodium ascorbate solution (120 µL, 100 mM in H_2O, 12 µmol) and DIPEA (5 µL, 28.7 µmol) were added. The reaction was stirred at r.t. for overnight. After lyophilization, the product was purified by preparative HPLC (water/acetonitrile with 1% formic acid, 20-45% acetonitrile). The product **19** was obtained as brick coloured solid (5.6 mg, 4.4 µmol, 61%).

^{1}H NMR (500 MHz, DMSO-d_6) δ 9.86 (s, 2H, NHCO), 8.01 (t, *J* = 5.6 Hz, 1H, NHCO), 7.72 (s, 1H, CH-triazole), 7.67 (s, 1H, ArH-BODIPY), 7.51 (d, *J* = 8.2 Hz, 4H, ArH), 7.21 (d, *J* = 8.3 Hz, 4H, ArH), 7.14 (d, *J* = 8.4 Hz, 4H, ArH), 7.07 (d, *J* = 4.0 Hz, 1H, ArH-BODIPY), 6.97 – 6.89 (m, 4H, ArH), 6.35 (d, *J* = 4.0 Hz, 1H, ArH-BODIPY), 6.29 (s, 1H, ArH-BODIPY), 5.11 (s, 2H, OH-2), 4.83 (s, 2H, OH-3), 4.75 (d, *J* = 7.7 Hz, 2H, H-1), 4.63 (s, 2H, OH-6), 4.48 (s, 2H, OH-4), 4.27 (t, *J* = 7.0 Hz, 2H, CH_2), 3.68 (d, *J* = 3.3 Hz, 2H, H-4), 3.57 – 3.42 (m, 14H, H-2, H-3, H-5, H-6, Ar-$\underline{C}H_2N$), 3.11 – 3.02 (m, 4H, 2xCH_2), 2.87 – 2.74 (m, 6H, $C\underline{H}_2CH_2CONH$, CH_2), 2.65 – 2.53 (m, 6H, $CH_2C\underline{H}_2CONH$, CH_2), 2.53 – 2.51 (m, 2H CH_2), 2.46 (s, 3H, CH_3), 2.25 (s, 3H, CH_3), 1.91 (t, *J* = 6.9 Hz, 2H, CH_2).
^{13}C NMR (126 MHz, DMSO-d_6) δ 171.00 (1C, NHC=O), 170.33 (2C, NHC=O), 159.19 (1C, ArC-BODIPY), 157.77 (1C, ArC-BODIPY), 155.84 (2C, ArC), 145.04 (1C, $\underline{C}$=CH-triazole), 144.13 (1C, ArC-BODIPY), 137.96 (2C, ArC), 134.47 (1C, ArC-BODIPY), 134.36 (2C, ArC), 133.83 (2C, ArC), 132.98 (1C, ArC-BODIPY), 129.04 (4C, ArCH), 128.93 (1C, ArCH-BODIPY), 128.87 (4C, ArCH), 125.37 (1C, ArCH-BODIPY), 122.08 (1C, C=$\underline{C}$H-triazole), 120.30 (1C, ArCH-BODIPY), 118.90 (4C, ArCH), 116.59 (4C, ArCH), 116.20 (1C, ArCH-BODIPY), 101.14 (2C, C-1), 75.44 (2C, C-5), 73.32 (2C, C-3), 70.30 (2C, C-2), 68.16 (2C, C-4), 60.40 (2C, C-6), 56.75 (2C, CH_2), 52.39 (1C, CH_2), 46.99 (1C, CH_2), 38.25 (2C, $CH_2\underline{C}H_2CONH$), 35.86 (1C, CH_2), 33.77 (1C, CH_2), 30.08 (2C, $\underline{C}H_2CH_2CONH$), 29.96 (1C, CH_2), 23.99 (1C, CH_2), 22.90 (1C, CH_2), 14.53 (1C, CH_3), 11.01 (1C, CH_3).
HPLC-MS: $[C_{65}H_{78}NF_2N_9O_{15} + H]^+$ calcd. 1274.58, found 1274.73.
HRMS: $[C_{65}H_{78}NF_2N_9O_{15} + H]^+$ calcd. 1274.5752, found 1274.5719.

Divalent fluorescent ligand 21

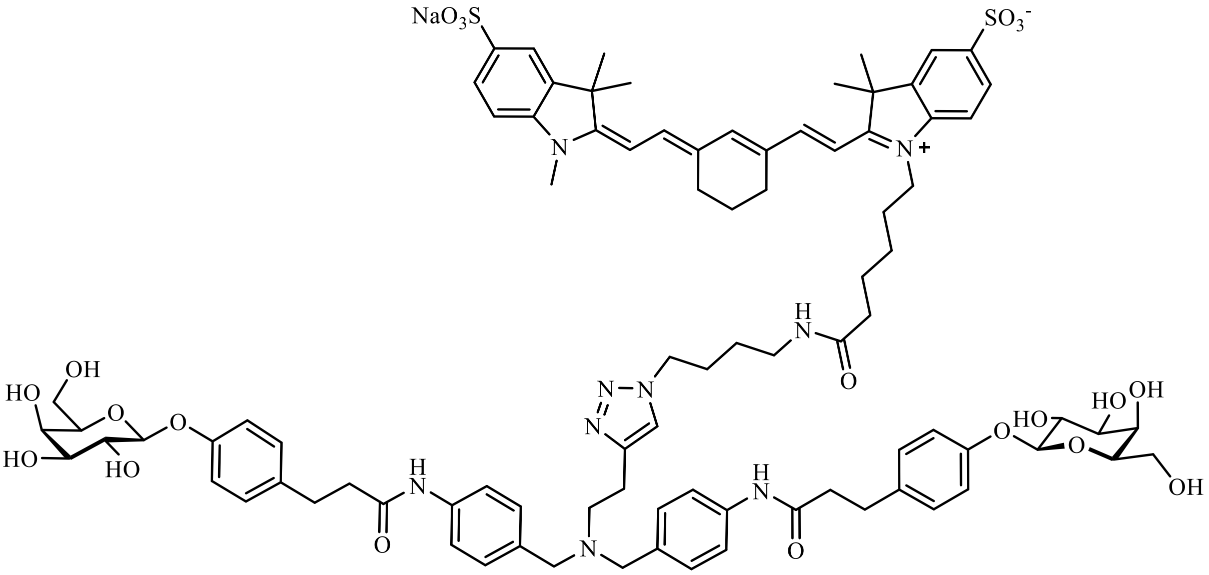

Divalent alkyne precursor **16** (6.75 mg, 7.5 µmol) and sulfo-Cyanine7 azide (**20**, 4.33 mg, 5.2 µmol) were dissolved in DMF (300 µL). $CuSO_4$ solution (241 µL, 100 mM in H_2O, 24.1 µmol), sodium ascorbate solution (181 µL, 100 mM in H_2O, 18.1 µmol) and DIPEA (5 µL, 28.7 µmol) were added. The reaction was stirred at r.t. for 3 h. After lyophilization, the product was purified by preparative HPLC (water/acetonitrile with 1% formic acid, 20-45% acetonitrile). The product **21** was obtained as a green solid (6.8 mg, 4.0 µmol, 77%).
^{1}H NMR (700 MHz, DMSO-d_6) δ 10.09 (s, 2H, CONH), 9.65 (s, 1H, SO_3H), 7.93 (s, 1H, CH-triazole), 7.84 (t, *J* = 5.7 Hz, 1H, CONH), 7.75 (dd, *J* = 8.9, 1.6 Hz, 2H, ArH), 7.73 – 7.58 (m,

9H, ArH, 2xCH=CH), 7.44 (s-broad, 4H, ArH), 7.31 (d, *J* = 8.3 Hz, 1H, ArH), 7.24 (d, *J* = 8.3 Hz, 1H, ArH), 7.18 – 7.10 (m, 4H, ArH), 6.98 – 6.90 (m, 4H, ArH), 6.15 (dd, *J* = 19.2, 14.0 Hz, 2H, 2xCH=CH), 5.10 (s, 2H, OH), 4.83 (s, 2H, OH), 4.75 (d, *J* = 7.7 Hz, 2H, H-1), 4.62 (s, 2H, OH), 4.47 (s, 2H, OH), 4.34 (s-broad, 4H, Ar-CH_2N), 4.26 (t, *J* = 7.0 Hz, 2H, CH_2), 4.11 (t, *J* = 7.1 Hz, 2H, CH_2), 3.68 (d, *J* = 3.3 Hz, 2H, H-4), 3.61 (s, 3H, NCH_3), 3.56 – 3.44 (m, 8H, H-2, H-5, H-6), 3.41 – 3.37 (m, 2H, H-3), 3.20 (s-broad, 2H, CH_2), 3.12 (s-broad, 2H, CH_2), 2.96 (q, *J* = 6.6 Hz, 2H, CH_2), 2.84 (t, *J* = 7.7 Hz, 4H, $C\underline{H_2}CH_2CONH$), 2.60 (t, *J* = 7.5 Hz, 4H, $CH_2C\underline{H_2}CONH$), 2.49 – 2.46 (m, 2H, CH_2), 2.04 (t, *J* = 7.1 Hz, 2H, CH_2), 1.84 – 1.77 (m, 4H, $2xCH_2$), 1.71 (q, *J* = 7.8, 7.2 Hz, 2H, CH_2), 1.64 (s, 6H, CH_3), 1.62 (s, 6H, CH_3), 1.54 (p, *J* = 7.3 Hz, 2H, CH_2), 1.30 (p, *J* = 7.9 Hz, 2H, CH_2).
^{13}C NMR (176 MHz, DMSO-d_6) δ 172.03 (1C, NHC=O), 170.80 (2C, NHC=O), 170.39 (1C, $C=N^+$), 155.86 (2C, ArC), 148.27 (1C, CH=CH), 147.17 (1C, CH=CH), 145.21, 144.57, 142.86, 142.48, 141.86, 140.42, 140.26, 140.12, 134.21, 132.29, 132.03, 129.06, 126.08, 123.82, 122.86, 119.78, 119.07, 116.19, 110.01, 109.81, 109.54, 101.12, 100.66, 99.78, 75.45, 73.32, 70.30, 68.15, 60.41, 55.79, 49.87, 48.64, 48.43, 47.17, 43.24, 38.22, 35.63, 35.00, 31.29, 29.96, 27.14, 26.92, 26.50, 25.65, 24.84, 23.33, 21.06.
HPLC-MS: $[C_{88}H_{107}N_9O_{21}S_2 + 2H]^{2+}$ calcd. 845.86, found 846.25.
HRMS: $[C_{88}H_{107}N_9O_{21}S_2 + 2H]^{2+}$ calcd. 845.8584, found 845.8547.

Imaging probe 22

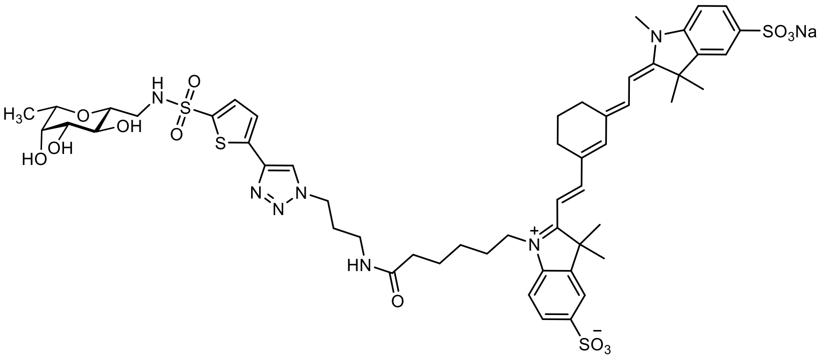

Sulfo-Cyanine7 azide (**20**, 4.7 mg, 5.7 µmol) and propargylated 1-deoxy fucoside **23** (4.2 mg, 12.1 µmol) were dissolved in DMF/H_2O (3:1, 400 µL). $CuSO_4$ solution (30 µL, 100 mM in H_2O, 3.0 µmol), sodium ascorbate solution (30 µL, 100 mM in H_2O, 3.0 µmol) and DIPEA (3 µL, 17.2 µmol) were added. The reaction was stirred at r.t. for 4 h and then lyophilized. Product was purified by preparative HPLC (water/acetonitrile with 1% formic acid, 20-55% acetonitrile). Compound **22** was obtained as green solid (6.0 mg, 5.3 µmol, 93%).
^{1}H NMR (500 MHz, DMSO) δ 8.69 (s, 1H, C<u>H</u>-triazole), 7.87 (t, *J* = 5.6 Hz, 1H, NHCO), 7.82 (s, 1H, $NHSO_2$), 7.75 (dd, *J* = 5.1, 1.6 Hz, 2H, ArH), 7.73 – 7.66 (m, 2H, CH=CH), 7.66 – 7.60 (m, 2H, ArH), 7.55 (d, *J* = 3.9 Hz, 1H, ArH-thiophene), 7.45 (d, *J* = 3.8 Hz, 1H, ArH-thiophene), 7.28 (dd, *J* = 14.9, 8.3 Hz, 2H, ArH), 7.22 – 7.01 (m, 1H, CH=CH), 6.14 (dd, *J* = 14.2, 4.6 Hz, 2H, CH=CH), 4.83 (d, *J* = 5.3 Hz, 1H, OH-2), 4.60 (d, *J* = 5.7 Hz, 1H, OH-3), 4.38 (t, *J* = 6.9 Hz, 2H, triazole-$C\underline{H_2}CH_2CH_2NHCO$), 4.27 (d, *J* = 5.5 Hz, 1H, OH-4), 4.11 (t, *J* = 7.1 Hz, 2H, $NHCOCH_2CH_2CH_2CH_2CH_2C\underline{H_2}$), 3.61 (s, 3H, CH_3N), 3.43 – 3.37 (m, 2H, H-4, H-5), 3.31 – 3.29 (m, 1H, $C\underline{H_{2,a}}NHSO_2$), 3.25 – 3.19 (m, 1H, H-3), 3.19 – 3.11 (m, 1H, H-2), 3.09 – 2.96 (m, 3H, H-1, triazole-$CH_2CH_2C\underline{H_2}NHCO$), 2.81 (dd, *J* = 13.0, 8.4 Hz, 1H, $C\underline{H_{2,b}}NHSO_2$), 2.52 – 2.47 (m, 4H, $CH=CHC\underline{H_2}CH_2C\underline{H_2}CH=CH$), 2.05 (t, *J* = 7.3 Hz, 2H,

$NHCOC\underline{H}_2CH_2CH_2CH_2CH_2CH_2$), 1.90 (p, J = 6.9 Hz, 2H, triazole-$CH_2C\underline{H}_2CH_2NHCO$), 1.79 (p, J = 6.9, 6.4 Hz, 2H, $CH{=}CHCH_2C\underline{H}_2CH_2CH{=}CH$), 1.74 – 1.68 (m, 2H, $NHCOCH_2CH_2CH_2CH_2C\underline{H}_2CH_2$), 1.65 (d, J = 10.9 Hz, 12H, $C\underline{H}_3CC\underline{H}_3$), 1.54 (p, J = 7.4 Hz, 2H, $NHCOCH_2C\underline{H}_2CH_2CH_2CH_2CH_2$), 1.37 – 1.26 (m, 2H, $NHCOCH_2CH_2CH_2C\underline{H}_2CH_2CH_2$), 1.07 (d, J = 6.3 Hz, 3H, Fuc-CH_3).
^{13}C NMR (126 MHz, DMSO) δ 171.99 (1C, C=O), 171.90 (1C, ArC), 170.55 (1C, ArC), 148.09 (1C, CH=CH), 147.34 (1C, CH=CH), 145.16 (1C, ArC), 144.82 (1C, ArC), 142.87 (1C, ArC), 142.33 (1C, ArC), 140.34 (1C, ArC-triazole), 140.22 (1C, ArC), 140.12 (1C, ArC), 139.73 (1C, ArC-thiophene), 138.54 (1C, ArC-thiophene), 132.22 (1C, ArC), 132.08 (1C, ArCH-thiophene), 132.02 (1C, ArC), 126.09 (1C, ArCH), 126.02 (1C, ArCH), 123.82 (1C, ArCH-thiophene), 122.20 (1C, ArCH-triazole), 119.77 (1C, ArCH), 119.73 (1C, ArCH), 109.92 (1C, ArCH), 109.87 (1C, ArCH), 100.53 (1C, CH=CH), 99.85 (1C, CH=CH), 78.31 (1C, C-1), 74.64 (1C, C-32), 73.65 (1C, C-5), 71.58 (1C, C-4), 69.78, 68.29 (1C, C-2), 48.59 (1C, $CH_3\underline{C}CH_3$), 48.47 (1C, $CH_3\underline{C}CH_3$), 47.67 (1C, triazole-$\underline{C}H_2CH_2CH_2NHCO$), 44.80 (1C, CH_2NHSO_2, 43.21 (1C, $NHCOCH_2CH_2CH_2CH_2CH_2\underline{C}H_2$), 35.64 (1C, triazole-$CH_2CH_2\underline{C}H_2NHCO$), 35.07 (1C, $NHCO\underline{C}H_2CH_2CH_2CH_2CH_2CH_2$), 31.26 (1C, CH_3N, 29.74, 27.12 (2C, $\underline{C}H_3C\underline{C}H_3$), 26.94 (2C, $\underline{C}H_3C\underline{C}H_3$), 26.55 (1C, $NHCOCH_2CH_2CH_2CH_2\underline{C}H_2CH_2$), 25.70 (1C, $NHCOCH_2CH_2CH_2\underline{C}H_2CH_2CH_2$), 24.87 (1C, $NHCOCH_2CH_2\underline{C}H_2CH_2CH_2CH_2$), 23.31 (2C, $CH{=}CH\underline{C}H_2CH_2\underline{C}H_2CH{=}CH$), 21.05 (1C, $CH{=}CHCH_2\underline{C}H_2CH_2CH{=}CH$), 16.93 (1C, Fuc-$CH_3$).
HPLC-MS: $[C_{53}H_{67}N_7O_{13}S_4 + 2H]^{2+}$ calcd. 569.69, found 569.76.
HRMS: $[C_{53}H_{67}N_7O_{13}S_4 + H]^+$ calcd. 1138.3753, found 1138.3749.

Biophysical evaluation

Expression and purification of LecA[17,18] and LecB (PAO1)[19,20] was performed in analogy to the previously published protocols. LecA was purified on melibiose-coupled sepharose CL- 6B affinity column[21] and then dialysed against TBS/Ca^{2+} buffer (20 mM Tris, 137 mM NaCl, 2.6 mM KCl at pH 7.4 supplemented with 1 mM $CaCl_2$). Protein concentration was determined by UV spectroscopy at 280 nm using a molar extinction coefficient of 27 960 M^{-1} cm^{-1} for LecA and 6 990 M^{-1} cm^{-1} for LecB.

All synthesized alkyne precursors (**S5*m***, **S5*p***, **S6*m***, **S6*p***, **S11**, **25**, **S16**, **S22** and **S24**) were analysed in the previously established competitive binding assays based on fluorescence polarization (Figure S2).[18,19] Galactose-based compounds (**S5*m***, **S5*p***, **S6*m***, **S6*p***, **S11**) were evaluated against LecA and C-glycoside-based compounds **23** and **S16** against LecB. As intended, xylose-based compounds **S22** and **S24** did not bind to LecA neither LecB in the tested concentration range and served as negative controls in both assays.

LecA competitive binding assay was performed in presence of 2% DMSO, whereas LecB competitive binding assay had less than 0.5% DMSO present. In short, the compound dilution series were prepared in 96 well plates. 10 µL of each dilution in triplicates were mixed with 10 µL of master mix (40 µM LecA, 20 nM galactose-fluorescein reporter ligand **S38** or 300 nM LecB and 20 nM **S39** in TBS/Ca^{2+} buffer, Figure S3) in 384-well microtiter plates (Greiner Bio-One, Germany, cat no 781900). The plates were centrifuged (1 min, 500 g), sealed (EASYseal, Greiner Bio-One, cat no 676001) and incubated for 1 h in case of LecA and 6 h in case of LecB in a dark wet chamber under gentle shaking. Fluorescence was measured on a

PheraStar FS plate reader (BMG Labtech GmbH, Germany) with excitation filters at 485 nm and emission filters at 535 nm. The measured intensities were reduced by the blank values (protein in TBS/Ca^{2+} buffer) and analysed with MARS Data Analysis Software (BMG Labtech GmbH, Germany). IC_{50} values were determined by the four-parameter variable slope model with the minimum and maximum limits of the fit fixed according to the controls in each assay (Me-α-D-Gal or L-Fuc).

Direct binding affinity determination of LecA and LecB imaging probes was performed with direct titration of protein and imaging probes in TBS/Ca^{2+} buffer. Protein dilution series was prepared in 96 well plates and 10 µL of each dilution in triplicates were mixed with 10 µL of fluorescent compound (20 nM) in 384-well microtiter plates (Greiner Bio-One, Germany, cat no 781900). The plates were centrifuged (1 min, 500 g), sealed and incubated for 2 h in dark wet chamber under gentle shaking. Fluorescence was measured on a PheraStar FS plate reader (BMG Labtech GmbH, Germany) with excitation filters at 485 nm and emission filters at 535 nm. The data were analysed with MARS Data Analysis Software (BMG Labtech GmbH, Germany) to calculate K_d values.

Surface plasmon resonance experiments were performed on a BIACORE X100 instrument (GE Healthcare) at 25 °C as described by Zahorska *et. al.*.[2] Averages and standard deviations were calculated from three independent experiments.

Isothermal titration calorimetry (ITC) was performed on an iTC200 (Malvern Panalytical) and the data were analyzed using Microcal Origin software (Malvern Panalytical). LecA (50 µM) in the cell was titrated with ligand (100 µM or 200 µM) in TBS/Ca^{2+} buffer supplemented with 5% DMSO at 25 °C. Steep titration slopes achieved with the divalent ligands were the result of high amount of protein present and the high binding affinity of the ligand (high 'value of c').[22] However, LecA protein concentration lower than 50 µM resulted in insufficient signal as a consequence of the low heat released upon binding.

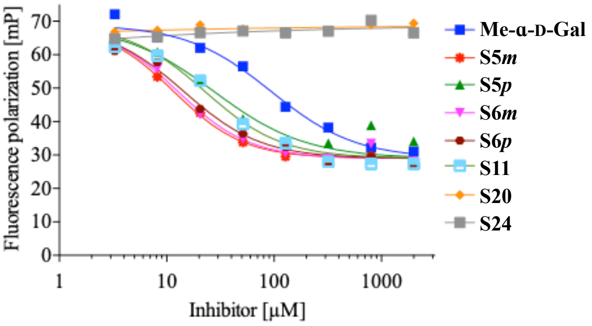

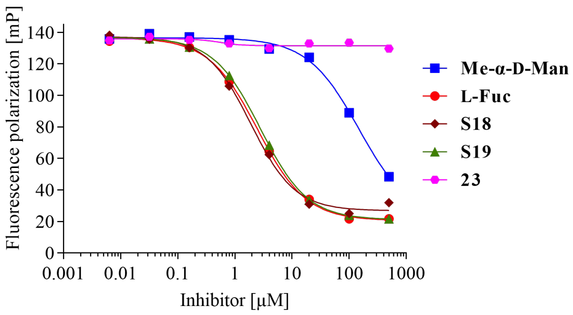

LecA			LecB		
Structure	Compound	IC50 [µM]	Structure	Compound	IC50 [µM]
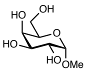	**Me-α-D-Gal**	101.0 ± 19.3	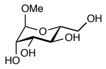	**Me-α-D-Man**	166 ± 22
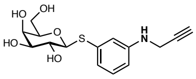	**S5*m***	17.8 ± 8.2	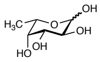	**L-Fuc**	2.6 ± 0.1
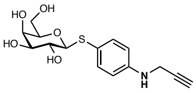	**S5*p***	31.5 ± 6.5	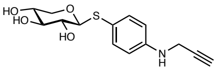	**S20**	n.b.
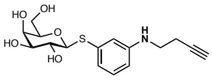	**S6*m***	17.8 ± 7.5	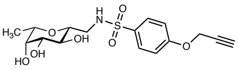	**S16**	3.8 ± 0.9
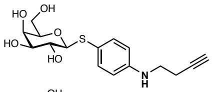	**S6*p***	22.3 ± 9.3	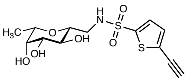	**23**	2.4 ± 0.5
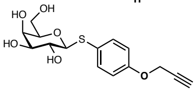	**S11**	24.3 ± 3.8			
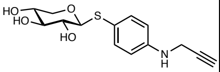	**S20**	n.b.			
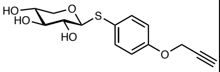	**S24**	n.b.			

Figure S2: Ligand evaluation against LecA (left) and LecB (right) in the competitive binding assay. One representative titration is shown for each. Averages and standard deviations from at least three independent titrations of triplicates each. n.b. = not binding.

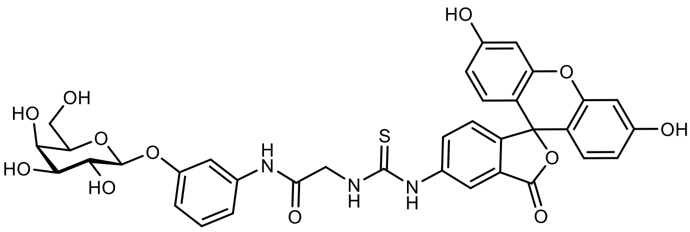

S38

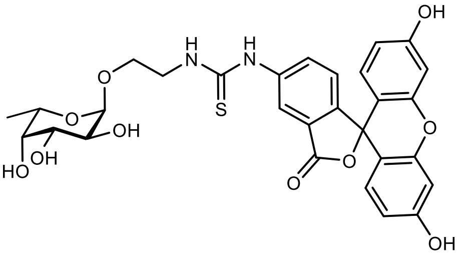

S39

Figure S3: Reporter ligands used in LecA (**S38**)[18] and LecB (**S39**)[19] competitive binding assays based on fluorescence polarization.

Bacterial cultivation

P. aeruginosa PAO1 expressing the red fluorescent protein mCherry (pMP7605)[23] was selected for this study. For this, *P. aeruginosa* PAO1 (DSM 19880, Braunschweig, Germany) was transformed with the mCherry plasmid (pMP7605).[23] Bacteria were stored at -80 °C. For experimental use, bacteria were streaked on lysogeny broth (LB) agar (1% tryptone, 1% NaCl, 0,5% yeast extract, 1.3% agar) containing 60 µg/mL gentamicin (Gm) and incubated at 37 °C overnight. For liquid cultures, bacterial colonies were transferred from LB agar to 10 mL LB (1% tryptone, 1% NaCl, 0,5% yeast extract) containing 60 µg/mL Gm and incubated at 37 °C and 180 rpm overnight. For exponential growth, 10 mL of LB containing 60 µg/mL Gm were inoculated with *P. aeruginosa* PAO1 mCherry (pMP7605)[23] using an overnight liquid culture and adjusted to OD_{600nm} 0.02.

In vitro biofilm staining under static conditions

Biofilm staining experiments were performed in analogy to Wagner *et al.* with *P. aeruginosa* PAO1 pMP7605 (Figure S4).[23,24] Bacterial overnight cultures were inoculated from single colonies in 5 mL LB supplemented with gentamicin (120 µg/mL) and grown at 37 °C and 180 rpm. The bacterial overnight cultures were then diluted to OD_{600} of 0.02 with LB and 500 µL of culture per well were transferred to 24-well plate (24CG, Art. No. 5231 or 24FC, Art. No. 3231). The biofilm was grown under shaking conditions (180 rpm) at 37 °C for 20–24 h. Biofilm aggregates were visualized using a confocal laser scanning microscope (Leica TCS Sp8 CLSM) using a 25× numerical-aperture water objective. Z-stacks (463 x 463 µm) were recorded every 2 µm for mCherry (ex.: 561 nm, red) and for fluorescein containing imaging compounds (ex.: 488 nm, green). 5 µL or 20 µL of 1 mM stock (TBS/Ca^{2+} buffer with 10% DMSO) of imaging compounds were carefully added to the wells containing biofilms (c = 10 µM or 40 µM, respectively) and incubated under static conditions for 30–190 min. Images were processed using the Imaris software (Bitplane AG An Oxford Instruments Company, Switzerland) by subtracting image backgrounds, applying a median filter and displayed as Maximum Intensity Projections (contrast range automatically adjusted by Imaris). Only one staining experiment was carried out for imaging probes **4** and **S26**, all other fluorescein conjugates were analyzed in at least three independent experiments.

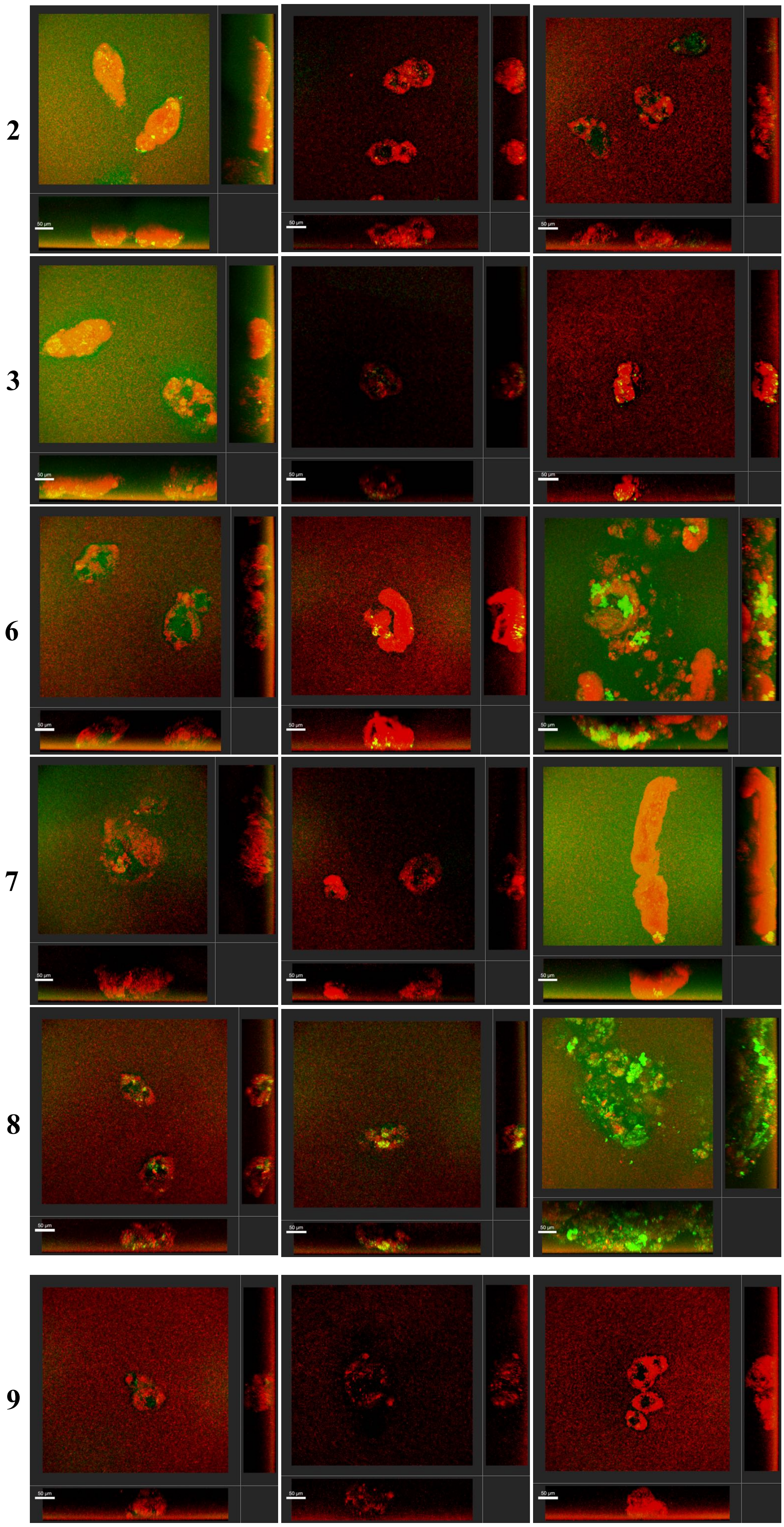

Figure S4: *P. aeruginosa* PAO1 pMP7605[23] biofilm aggregates (in red, from mCherry ex.: 561 nm) stained with 10 µM imaging compounds (in green, from fluorescein conjugate ex.: 488 nm) displayed as three-dimensional maximum intensity projections using Imaris. Images from three independent experiments are shown. Scale bars = 50 µm.

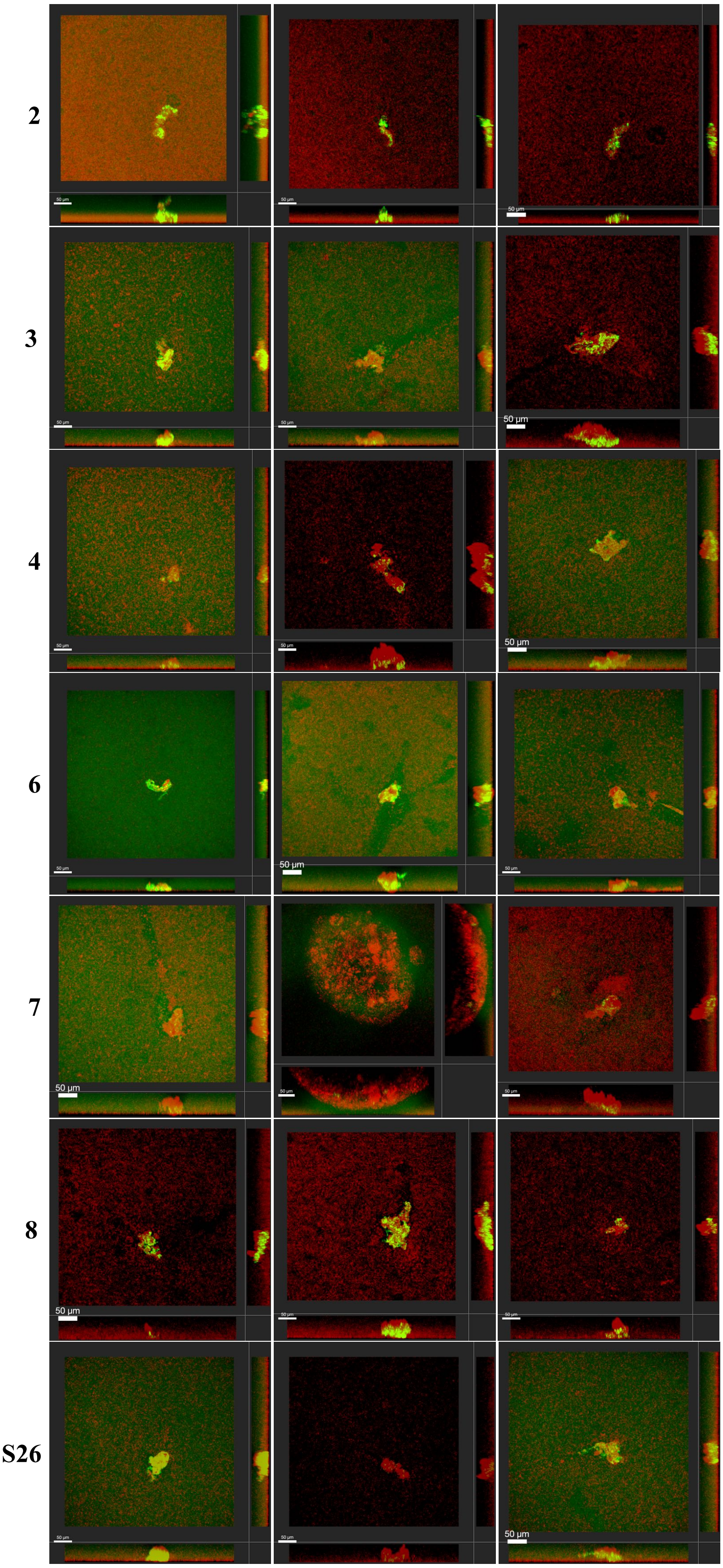

Figure S5: *P. aeruginosa* PAO1 pMP7605[23] biofilm aggregates (in red, from mCherry ex.: 561 nm) stained with 40 µM of probes (in green, from fluorescein conjugate ex.: 488 nm) displayed as three-dimensional maximum intensity projections using Imaris. Images from three independent experiments are shown. Scale bars = 50 µm.

9

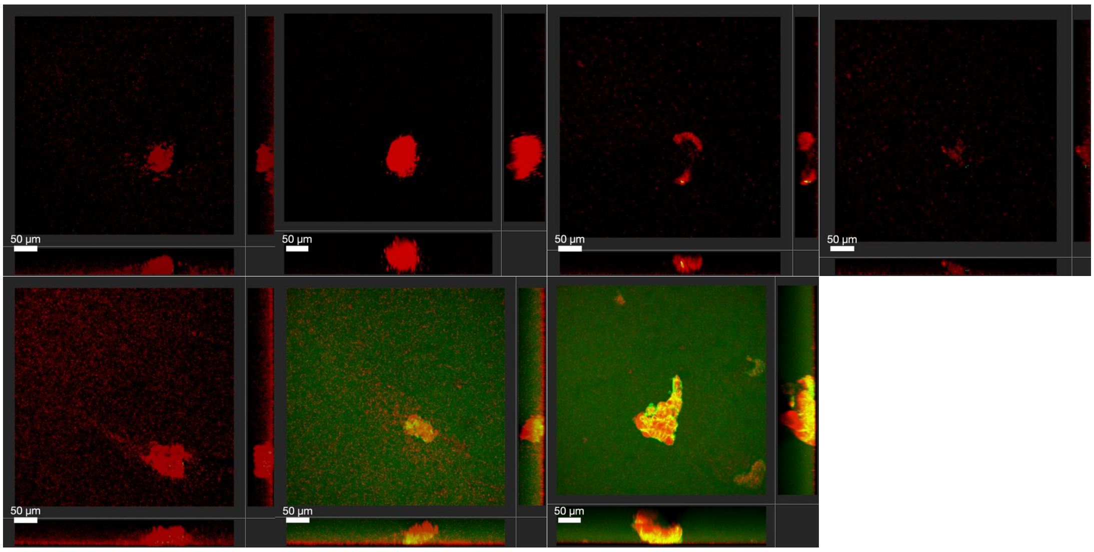

Figure S6: *P. aeruginosa* PAO1 biofilm aggregates stained with 40 µM of the azide modified fluorescein **9** and displayed as three-dimensional maximum intensity projection using Imaris. Inconsistent staining of **9** was observed. *P. aeruginosa* was expressing mCherry (ex.: 561 nm) from pMP7605[23] and is displayed in red and fluorescein conjugates (ex.: 488 nm) in green. Scale bars = 50 µm.

Stability and LecA binding and inhibition of divalent LecA ligands

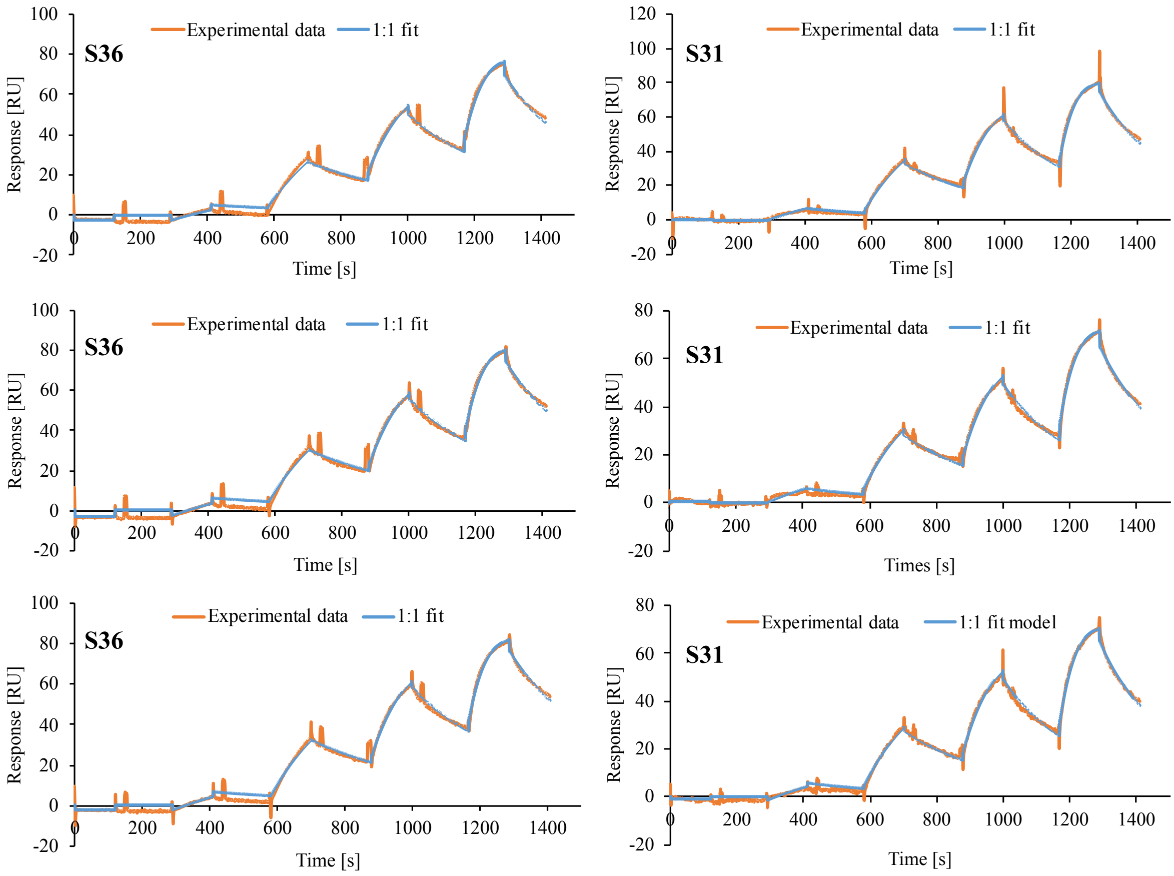

Figure S7: SPR of divalent LecA acylhydrazone ligand **S36** and divalent fluorescent ligand **S31**. Sensorgrams obtained from SPR single-cycle kinetics experiments. Five different concentrations of each compound (0, 10, 50, 100, 200 nM) were sequentially injected to obtain the experimental sensorgrams (orange lines), which were then fitted by a 1:1 model (blue lines) on BIACORE evaluation software.

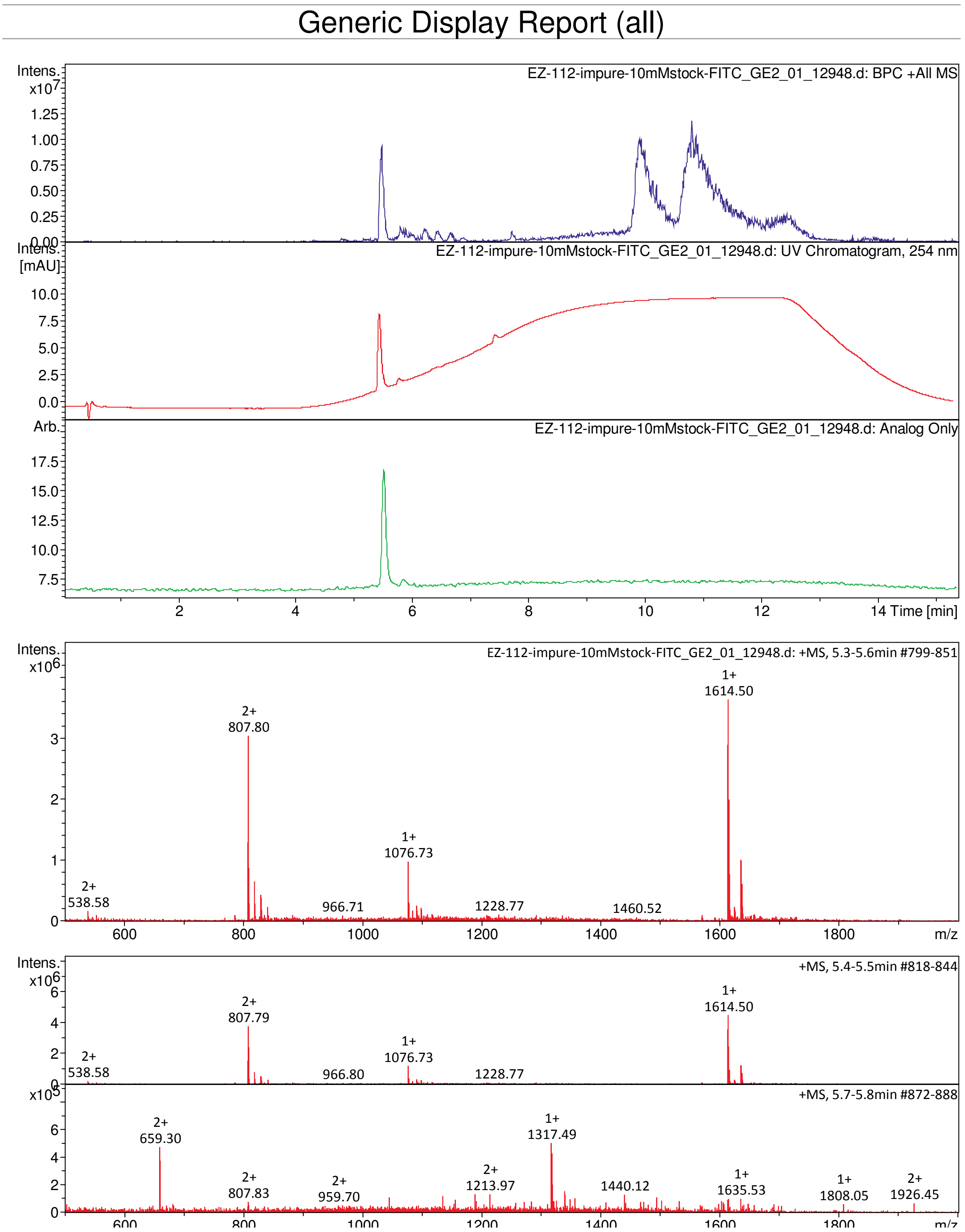

Figure S8: HPLC-MS chromatogram of the divalent fluorescent ligand **S31** from its DMSO stock. The analogue window (in green) displayed fluorescent detector signal set for fluorescein. The m/z of the major peak with retention time 5.5 min corresponds to compound **S31** (m/z 807.8^{2+}) while the minor peak with retention time 5.8 min corresponds to monohydrolyzed side product (m/z 659.3^{2+}).

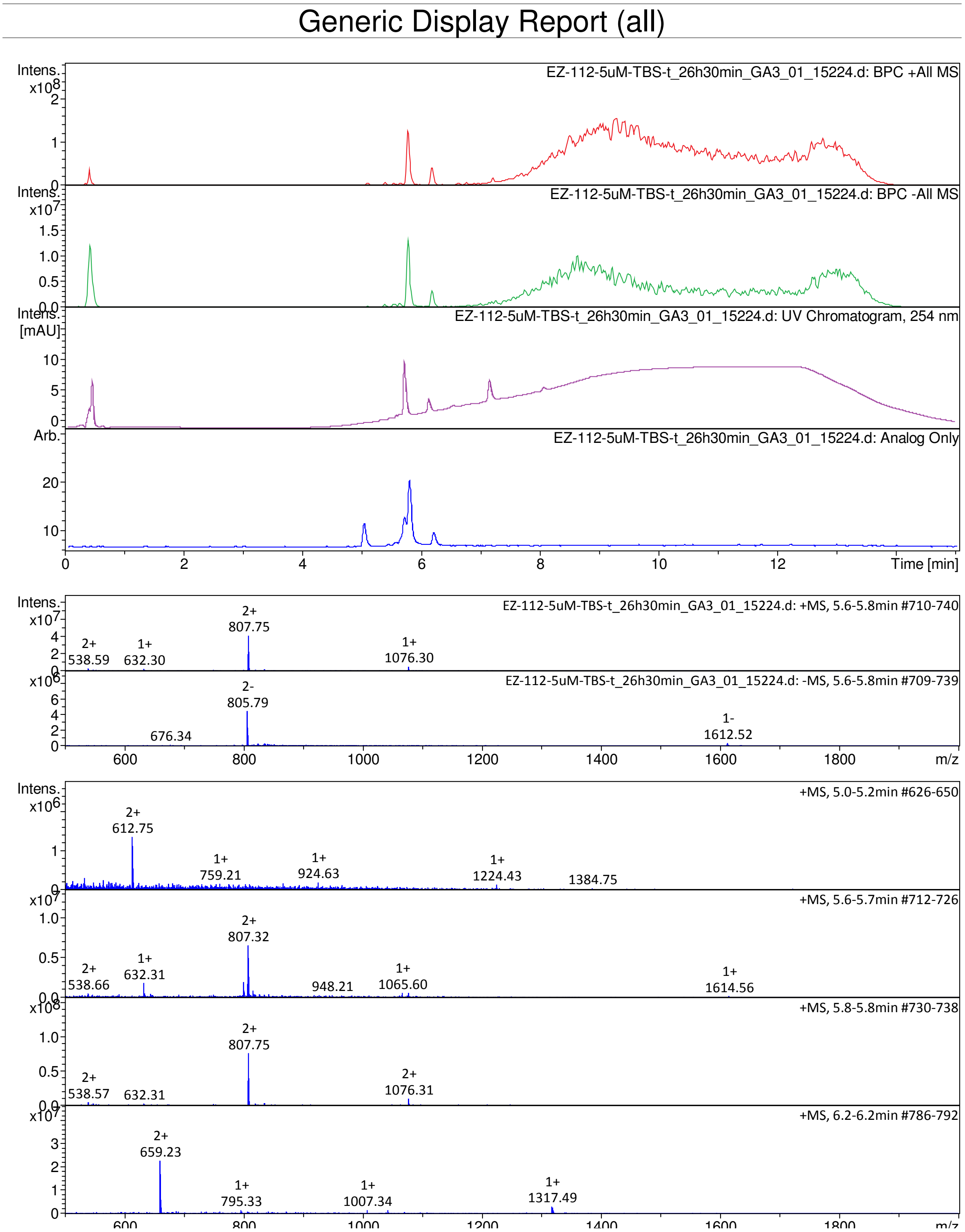

Figure S9: HPLC-MS chromatogram of the divalent fluorescent ligand **S31** in TBS/Ca^{2+} buffer after 26 h. The analogue window (in blue) displayed fluorescent detector signal set for fluorescein. New fluorescent peak of unknown identity was observed with retention time 5.1 min. The major peak, split by a shoulder, with retention time 5.5 min corresponds to compound **S31** (m/z 807.8^{2+}).

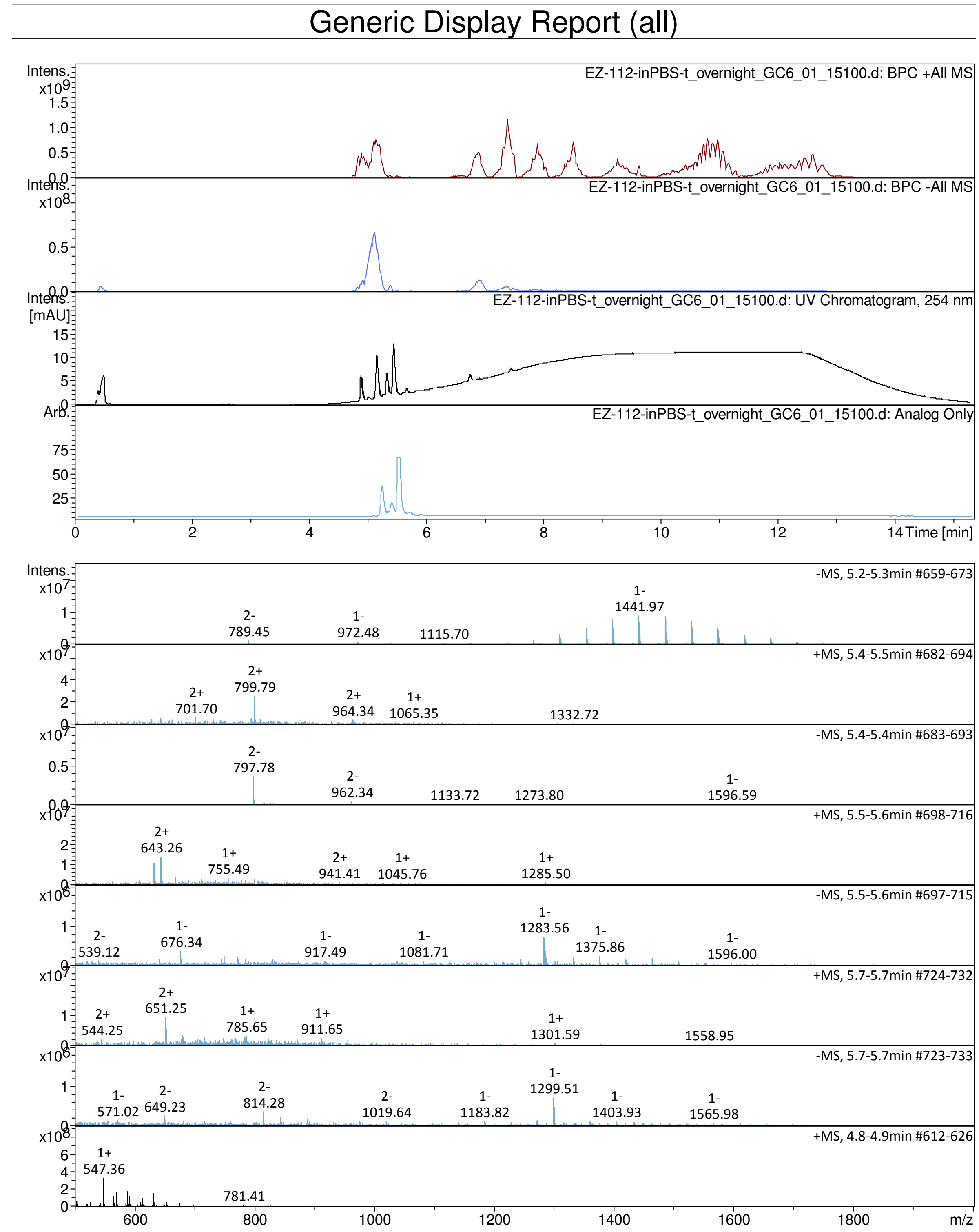

Figure S10: HPLC-MS chromatogram of divalent fluorescent ligand **S31** in PBS buffer (10 mM phosphate buffer pH 7.4, 2.7 mM KCl, 137 mM NaCl, 100 µM $CaCl_2$, 0.05% Tween 20) after overnight incubation (≈16 h). The analogue window (in light blue) displayed fluorescent detector signal set for fluorescein. The fluorescent peaks no longer corresponded to ligand **S31** (m/z 807.8^{2+}).

Generic Display Report

Analysis Info

Analysis Name	C:\Users\eza17\Documents\LCMS\EZ-SMY10-inTBS-24h_GA6_01_18147.d	Acquisition Date	06.05.2021 07:58:59
Method	18147.m	Operator	cbch
Sample Name	EZ-SMY10-inTBS-24h	Instrument	amaZon SL
Comment			

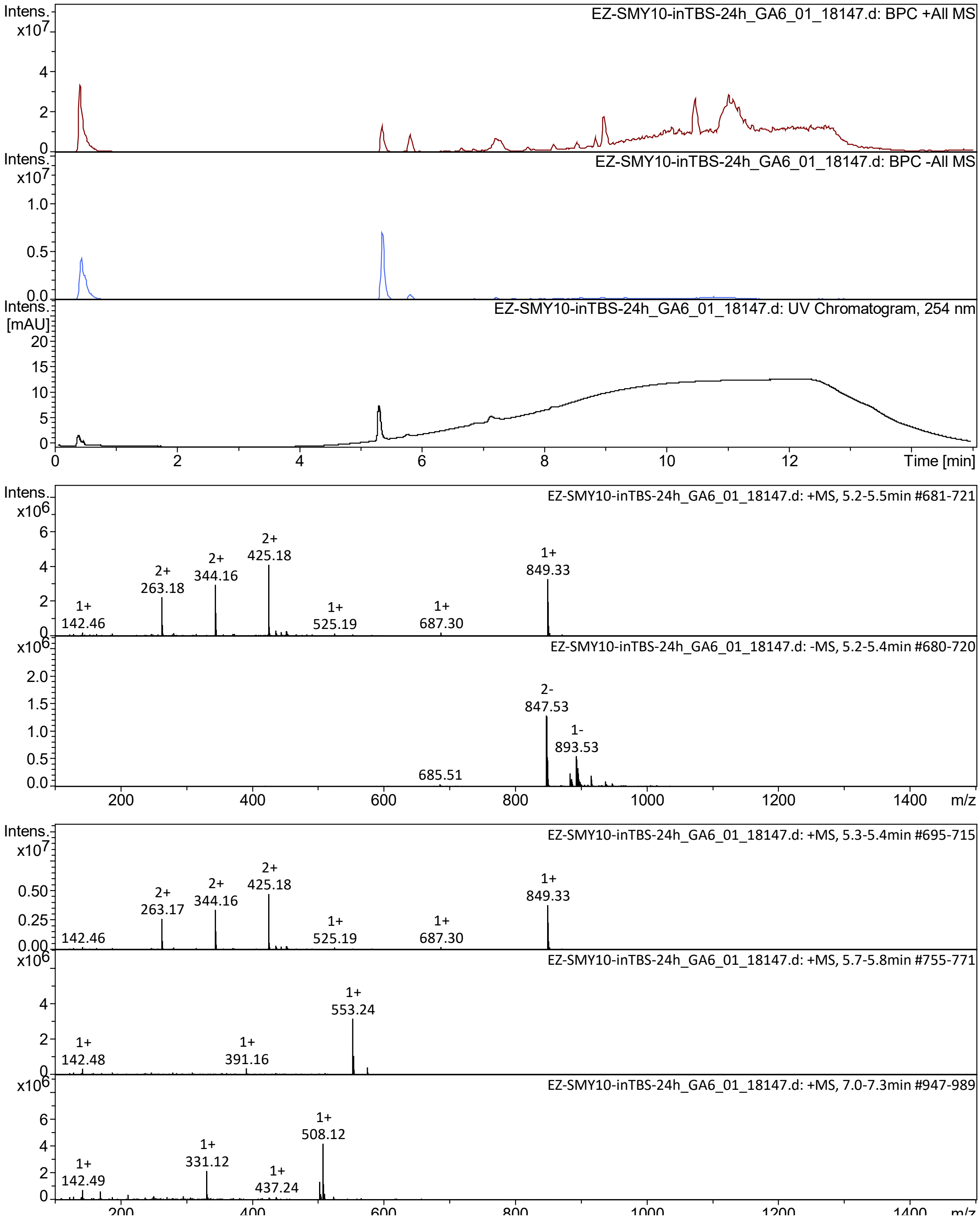

Bruker Compass DataAnalysis 4.4 printed: 06.05.2021 18:13:25 by: eza17 Page 1 of 1

Figure S11: HPLC-MS chromatogram of the parent acylhydrazone ligand **S30** (m/z 849.28[1+]) in TBS/Ca^{2+} buffer after 24 h.

Generic Display Report

Analysis Info

Analysis Name	C:\Users\eza17\Documents\LCMS\EZ-116-inTBS-36h_GA5_01_18153.d	Acquisition Date	06.05.2021 22:55:45
Method	18153.m	Operator	cbch
Sample Name	EZ-116-inTBS-36h	Instrument	amaZon SL
Comment			

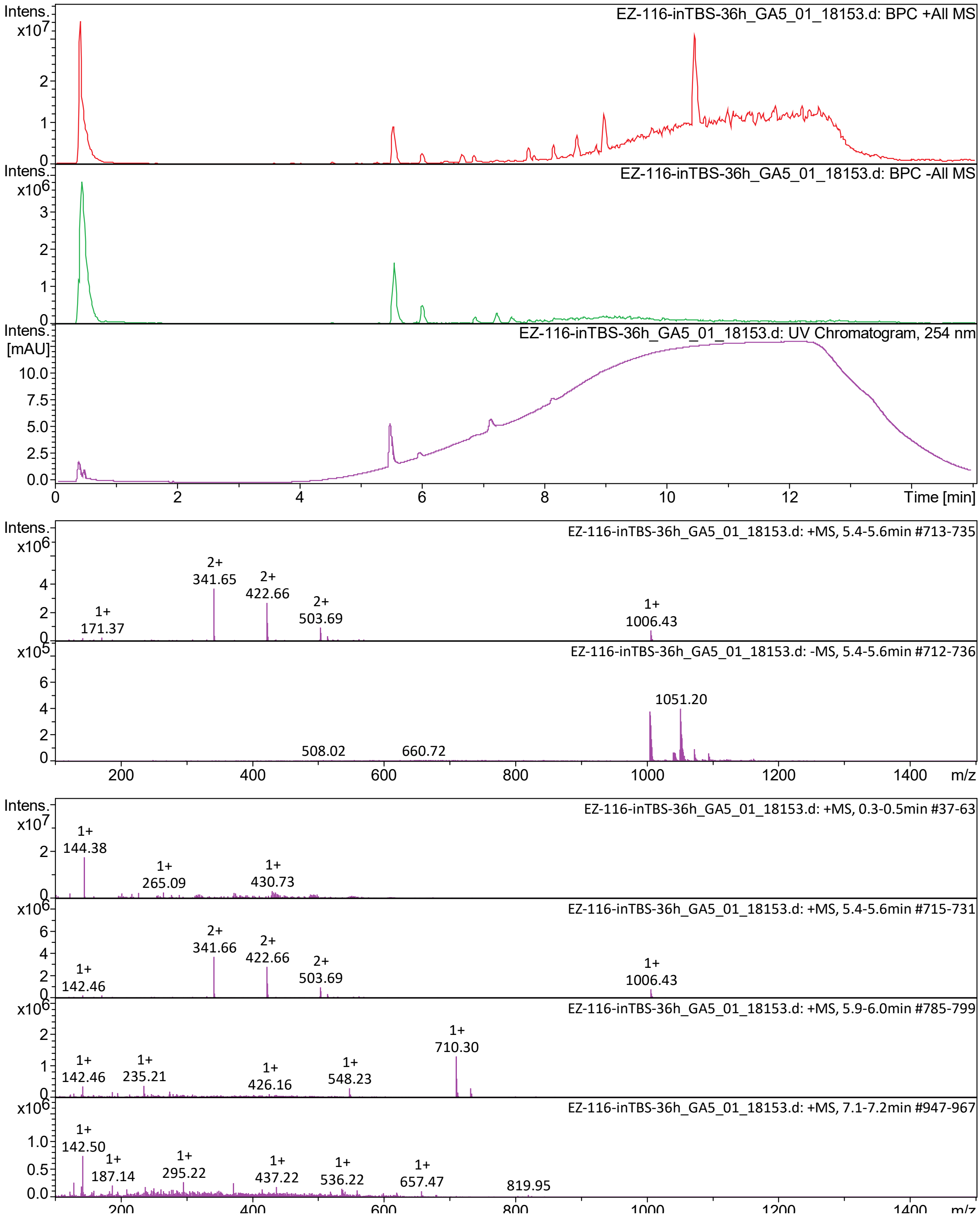

Bruker Compass DataAnalysis 4.4 printed: 07.05.2021 13:01:36 by: eza17 Page 1 of 1

Figure S12: HPLC-MS chromatogram of the branched divalent acylhydrazone ligand **S36** (m/z 1006.34^{1+}) in TBS/Ca^{2+} buffer after 36 h.

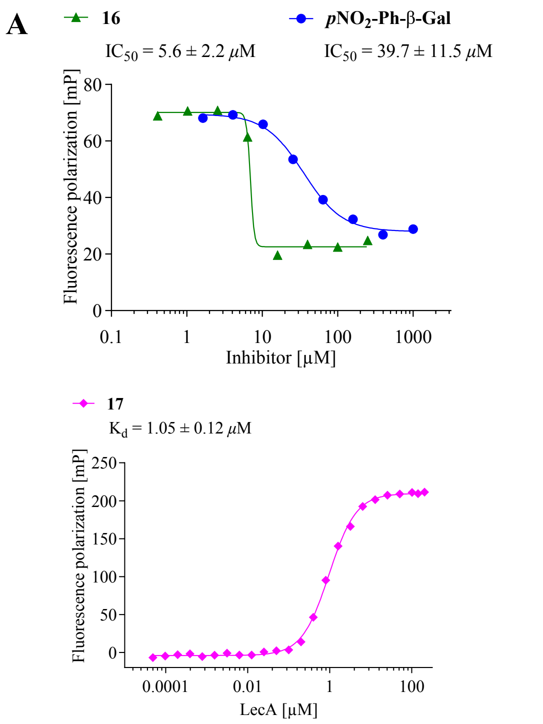

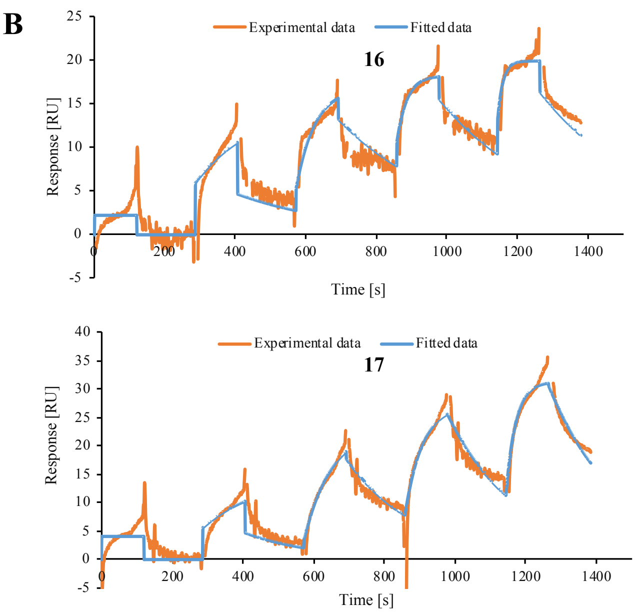

	k_{on} [x 10^3 $M^{-1}s^{-1}$]	k_{off} [x 10^{-3} s^{-1}]	kinetic K_d [nM]	equilibrium# K_d [nM]
**16**	359 ± 110	3.59 ± 1.30	9.9 ± 0.5	47.8 ± 44.1
**17**	255 ± 150	4.21 ± 1.35	19.3 ± 10.5	79.3 ± 24.9

Figure S13: Evaluation of the optimized fluorescent ligand 17 and its synthetic precursor 16 in LecA binding assays. (A) Competitive binding (top) and direct binding (bottom) by fluorescence polarization. (B) Kinetic and equilibrium analysis by SPR (#values with limited reliability, equilibrium not fully reached). Data for one representative experiment are depicted in A and B. Averages and std. dev. from at least three independent experiments.

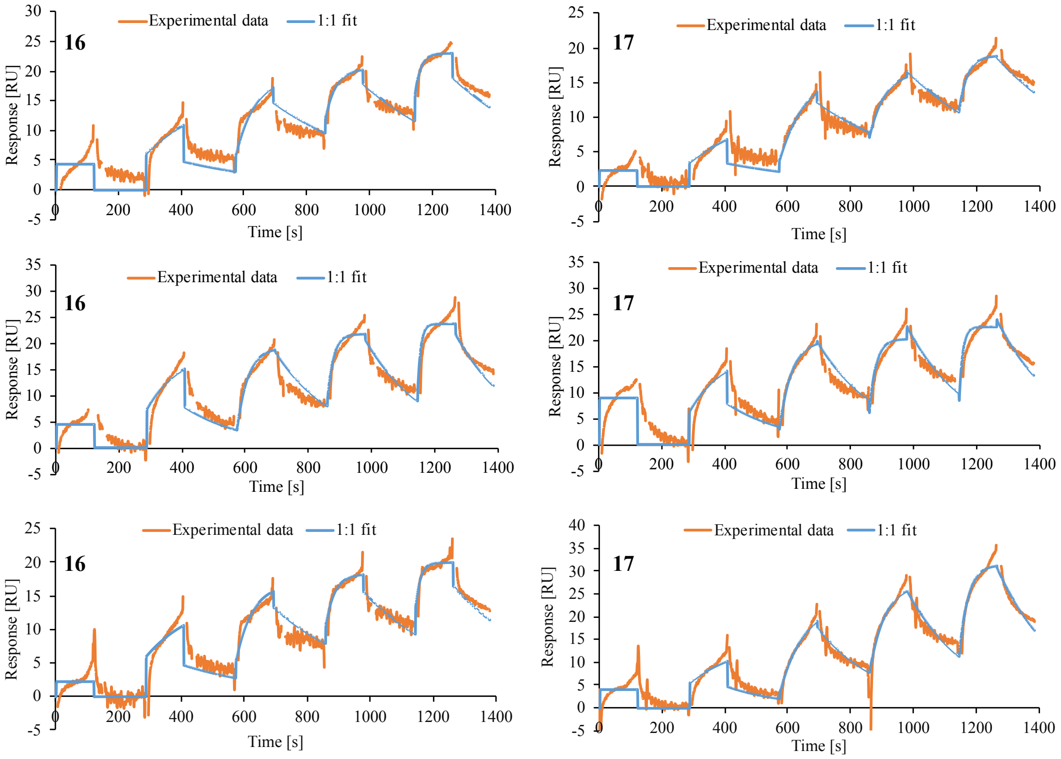

Figure S14: SPR of divalent LecA ligand 16 and divalent fluorescent ligand 17. Sensorgrams obtained from SPR single-cycle kinetics experiments. Five different concentrations of each compound (0, 10, 50, 100, 200 nM) were sequentially injected to obtain the experimental sensorgrams (orange lines), which were then fitted by a 1:1 model (blue lines) on BIACORE evaluation software.

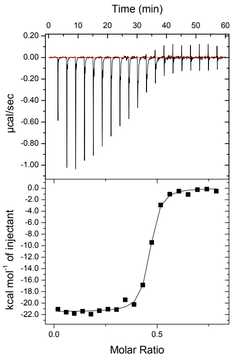

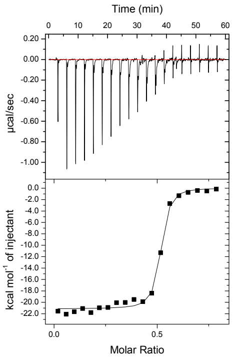

	50 µM LecA	50 µM LecA	
	200 µM **16**	200 µM **16**	Average ± s.d.
ΔG [kJ/mol]	-41,3	-42,5	-41.9 ± 0.6
ΔH [kJ/mol]	-90,1	-88,5	-89.3 ± 0.8
-TΔS [kJ/mol]	48,8	45,9	47.4 ± 1.4
N	0,45	0,50	0.47 ± 0.03
K_d [nM]	58,5	36,0	47.2 ± 11.3

Figure S15: ITC measurements of divalent alkyne ligand **16** with LecA performed in TBS/Ca2+ buffer with 5% DMSO at 25 °C.

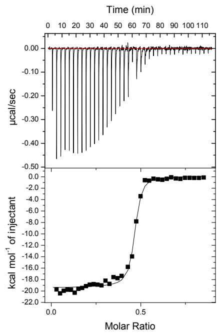

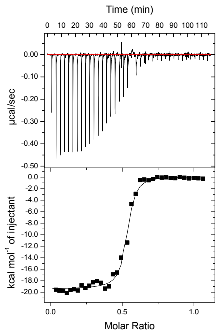

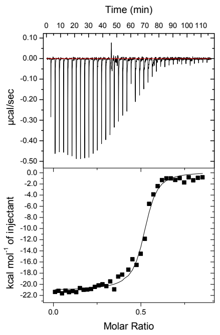

	50 µM LecA	50 µM LecA	50 µM LecA	
	100 µM **17**	100 µM **17**	100 µM **17**	Average ± s.d.
ΔG [kJ/mol]	-42,9	-41,5	-39,8	-41.4 ± 1.2
ΔH [kJ/mol]	-81,2	-81,3	-88,1	-83.5 ± 3.2
-TΔS [kJ/mol]	38,3	39,8	48,2	42.1 ± 4.3
N	0,46	0,52	0,51	0.50 ± 0.03
K_d [nM]	31,3	55,3	105,8	64.1 ± 31.1

Figure S16: ITC measurements of divalent fluorescent ligand **17** with LecA performed in TBS/Ca2+ buffer with 5% DMSO at 25 °C.

Generic Display Report (all)

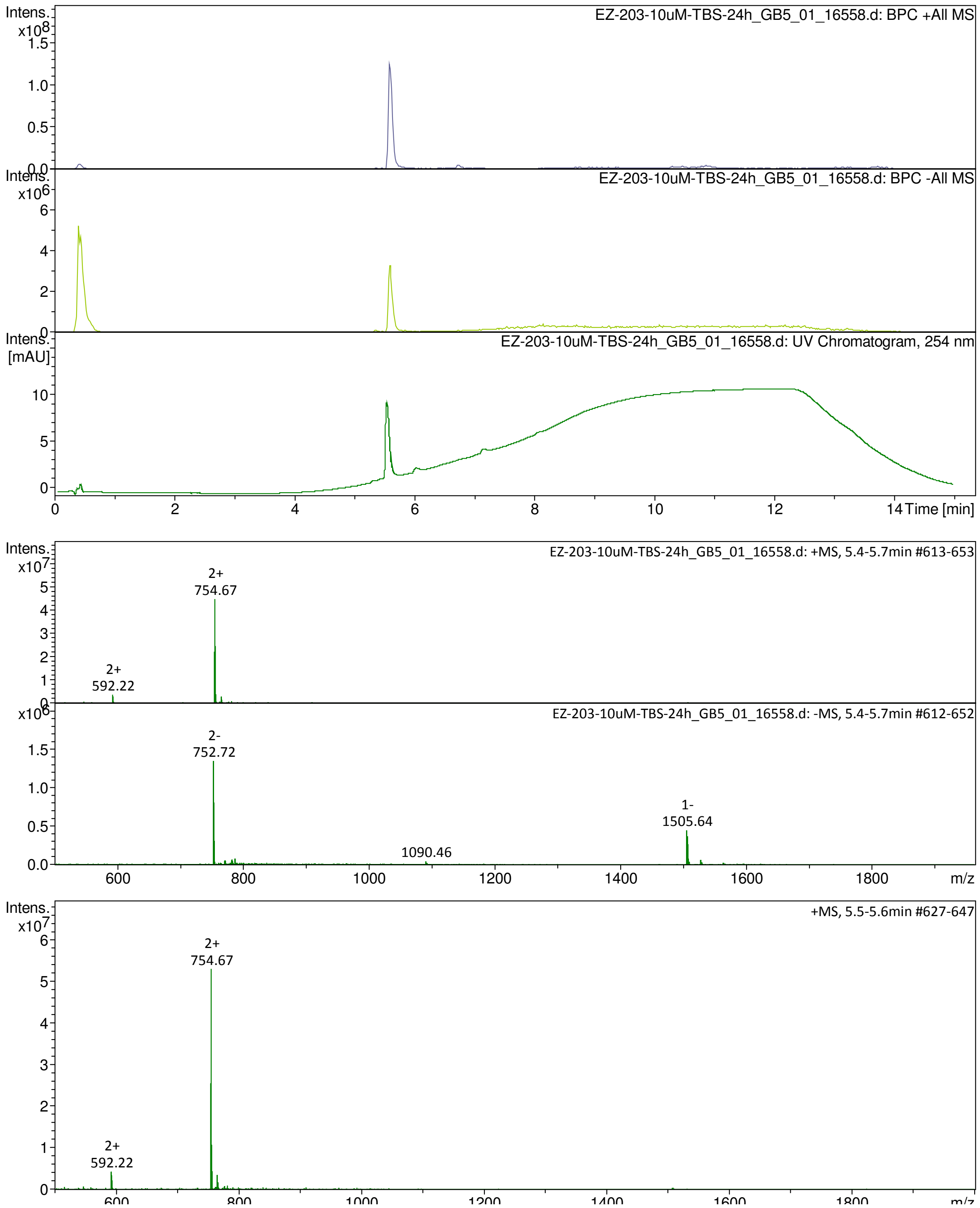

Figure S17: HPLC-MS chromatogram of divalent fluorescent ligand **17** (m/z 754.67^{2+}) in TBS/Ca^{2+} buffer after 24 h.

In vitro **biofilm staining under flow conditions**

The flow cell experiment was set up in analogy to Ghanbari *et al.* and in this study, optimized and established for biofilm staining.[25] In principle, the experiment is based on an inlet connected to a medium source, a peristaltic pump providing constant medium flow, a flow chamber enabling analysis of bacterial growth and an outlet (waste container/bottle).

The medium used in the flow system was LB diluted by factor 5 containing 60 µg/mL Gm. Silicone tubings 1.6 mm (ibidi, Germany) were used transporting medium from inlet to the peristaltic pump (Watson-Marlow Fluid Technology Solutions). Stopper hoses (CarlRoth) placed in peristaltic pump were coupled to silicone tubes via connectors (ibidi, Germany). Stopper hoses are connected to bubble traps (obtained by Claus Sternberg, DTU Copenhagen) via 0.8 mm silicone tubing (ibidi, Germany). Bubble traps were used to remove air from the medium flow and to avoid biofilm disruption. Downstream from the bubble traps, the flow cell (µ-Slide VI 0.4 ibiTreat, ibidi, Germany) was placed into the system and connected to 1.6 mm silicone tubes (ibidi, Germany). The outlet is leading the medium from flow chamber to a waste container.

In order to inoculate the flow cell with *P. aeruginosa* PAO1 mCherry (pMP7605)[23] the silicone tubes upstream and downstream of the flow chamber were pinched off by using commercial plastic clamps. The medium flow was stopped and the flow cell was inoculated by injecting 300 µL of the bacterial suspension (OD_{600nm} of 0.1) into the silicone tubing upstream of the flow chamber. For this, a cannula (0.30 x 12 mm, Sterican) was used. The injection point was sealed with silicone glue. After bacterial settling time of 30 min, the flow was started again and clamps were removed. Growth temperature was regulated by using a heat system (ibidi, Germany). Biofilm growth occurred at 30 °C for 48 h.

As described above, two different staining methods have been evaluated for *P. aeruginosa* PAO1 biofilm imaging, (I) compound injection into the system and (II) compound supplementation into the medium (Figure S17). Both biofilm staining procedures were performed and analyzed in the darkness.

With the injection method, silicone tubes were pinched off 3 cm upstream of the flow chamber. The flow system was stopped and compounds were injected into the tubing upstream of the slide containing biofilms (Figure S17A). Final concentrations were calculated based on the volume of the flow cell channel, tubings and connectors (262 µL), the injected volume (9 µL) and the concentration of the injected compound (500 µM, 250 µM, 100 µM) resulting in concentrations of 17 µM, 8.5 µM or 3.4 µM. The system flow was started again and the clamp was removed. The system flow transported the compound from the injection site (tubing) into the flow cell channel. When the dyes reached the biofilm aggregates as monitored by the fluorescence microscope, the flow was stopped and tubing pinched off again for a brief incubation period (10 min). After this, the flow was resumed and wash out of unbound compounds was monitored for a time period of 20 min by using fluorescence microscopy.

With the method of compound supplementation into the medium, the medium source was exchanged by LB diluted by factor 5 containing 60 µg/mL Gm and 500 nM of the imaging compounds (Figure S17B). Compounds were constantly added to the biofilm for a time period of 4 h via the system flow (3 mL/h). After this accumulation period, stained biofilms were washed with medium flow (30 min, 3 mL/h, no compound) and analyzed on a fluorescence microscope after 4 h of accumulation and 30 min of wash. CLSM analysis was performed after 4 h of compound accumulation without wash period.

Analysis using fluorescence microscopy (Leica DMi8) was based on recording timelapses and snapshots. Green signals corresponding to divalent LecA targeting fluorescein ligand **17**, divalent LecA targeting BODIPY ligand **19,** fluorescein control **24** and BDP FL azide control **18**, were detected by an excitation wavelength of 460 to 500 nm and an emission wavelength of 512 to 542 nm. mCherry signals were detected by an excitation wavelength of 540 to 580 nm and an emission wavelength of 592 to 668 nm.
Three-dimensional images were recorded using CLSM (Leica TCS Sp8 CLSM). Green signals originating from divalent LecA targeting BODIPY ligand **19** and control BDP FL azide **18** were detected at an excitation wavelength of 503 nm and an emission wavelength of 509 nm. mCherry signals were detected at an excitation wavelength of 561 nm and an emission wavelength of 568 nm.

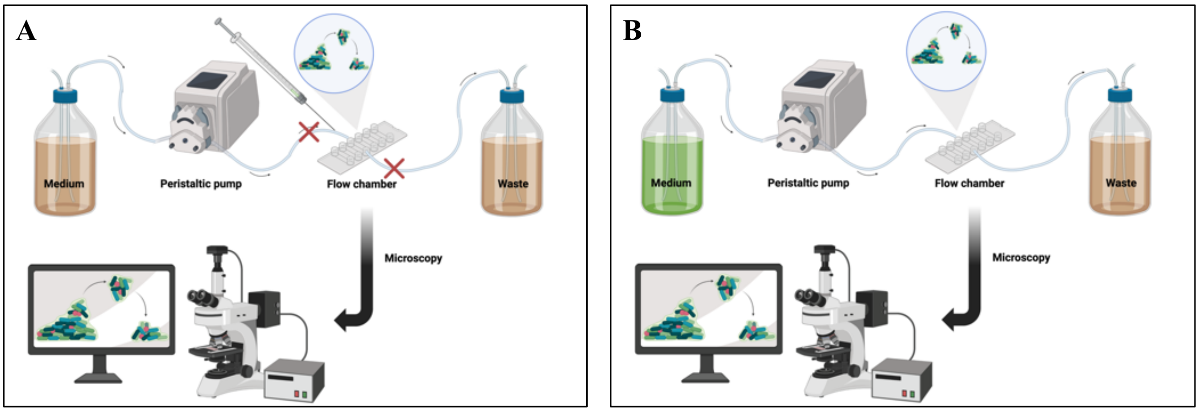

Figure S18: Experimental setup of two different staining procedures for *P. aeruginosa* PAO1 biofilm imaging using divalent LecA ligands. Two different staining procedures were optimized in order to analyse *P. aeruginosa* PAO1 biofilms grown under flow conditions for biofilm imaging using divalent LecA ligands: (A) compound injection into the system and (B) compound supplementation into the medium. Using the injection method, compounds were injected upstream of the flow chamber containing biofilms while the flow was stopped. The incubation time was 10 min following restorage of the flow and subsequent washing of the compounds by the constant flow rate. In contrast, using the supplementation method, compounds were added to the medium and constantly added to the biofilms in the flow chamber over a time period of 4 h. Created with Biorender.com.

Analysis of excitation and emission maxima of the divalent LecA targeting imaging probes

For analyzing the excitation and emission maxima of the imaging compounds, dilution series in TBS/Ca^{2+} (10 µM to 0.032 nM) were performed in 96-well imaging plates (Greiner). Measurements were done using a plate reader (Fluostar Omega, BMGLabtech). Detected excitation maxima of the fluorescein conjugated divalent imaging probe **17** and the fluorescein control **24** (CAS no.: 518-47-8, CarlRoth) were at 497 and 489 nm, respectively. Emission maxima were detected at 519 and 514 nm for compound **17** and **24** (Fig S18). For the BODIPY conjugated divalent LecA ligand **19** and the control BDP FL azide **18**, excitation maxima of 506 and 503 nm were observed. Emission maxima of **19** and **18** were detected at 513 and 511 nm (Fig S19).

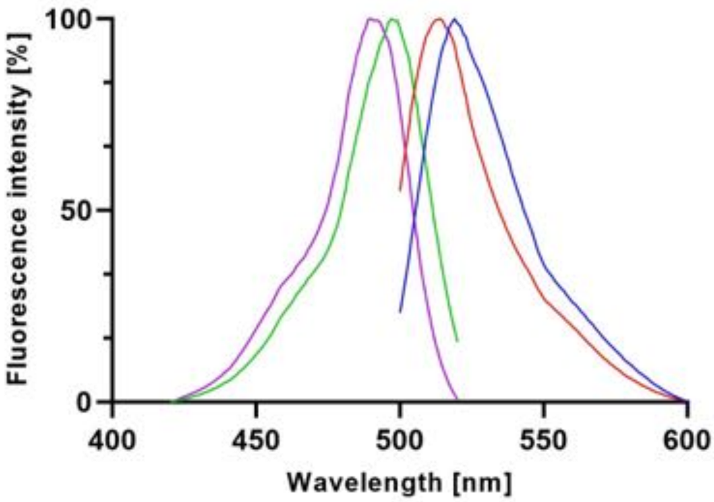

Figure S19: Excitation and emission maxima of the fluorescein conjugated divalent LecA ligand 17 and the fluorescein control 24. The divalent ligand **17** showed an excitation maximum at 497 nm and an emission maximum at 519 nm. For the control **24** an excitation maximum of 489 nm and an emission maximum of 514 nm were detected.

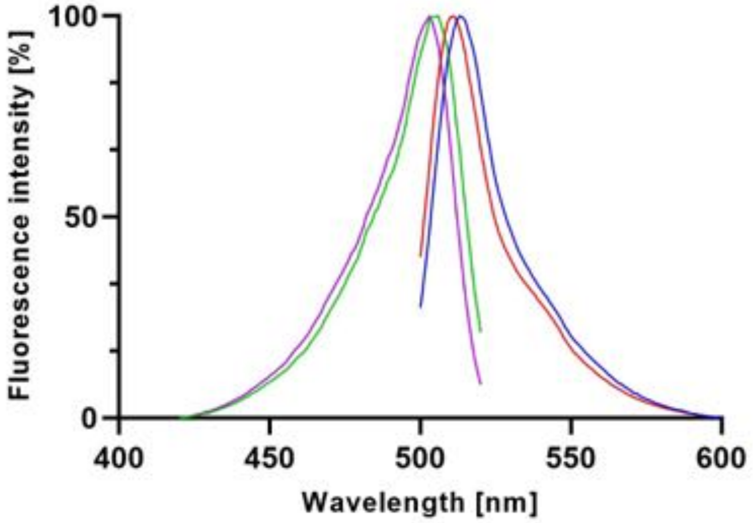

Figure S20: Excitation and emission maxima of the BODIPY conjugated divalent LecA ligand 19 and the control BDP FL azide 18. For the BODIPY conjugated divalent ligand **19** an excitation maximum of 506 nm and an emission maximum of 513 nm were detected. The control **18** showed an excitation maximum at 503 nm and an emission maximum at 511 nm.

Determination of fluorescence intensities

Fluorescein suffers from rapid bleaching and different compounds that were treated differently during synthesis may show different molar fluorescence. Therefore, fluorescence intensities of imaging compounds were analysed and compared to respective controls. For this, imaging compounds were serially diluted in TBS/Ca^{2+} in a 96-well imaging plate (Greiner). The analysis was performed using a plate reader (Fluostar Omega, BMGLabtech). Control fluorescein disodium salt **24** (CAS no.: 518-47-8, CarlRoth) showed a 4-fold higher fluorescence intensity compared to the fluorescein conjugated divalent imaging probe **17**. For

the control BDP FL azide (**18**) 10-fold higher intensities compared to the BODIPY conjugated divalent LecA ligand were achieved (**19**) (Table S1). Therefore, recorded image intensities were normalized using the Fiji software.

Table S1: Fluorescence intensities of fluorophore conjugated divalent LecA targeting imaging probes (17, 19) and the respective controls fluorescein and BDP FL azide (24, 18). Compounds were serially diluted in TBS/Ca^{2+} and fluorescence intensities were measured using a microplate reader.

Concentration [nM]	TBS/Ca^{2+}	17	24	19	18	TBS/Ca^{2+}
500	293	9906	45810	835	20234	299
100	296	1463	8827	345	3450	304
20	278	525	2391	287	1265	295
4	295	363	659	295	532	298
0.8	293	325	366	289	445	289
0.16	290	291	293	287	310	296
0.032	278	272	283	279	278	275
0.0064	301	285	293	304	295	298

Therefore, recorded image intensities were normalized using the ImageJ software. Fluorescence signals of recorded images were reduced by factor of 4 for fluorescein **24** and by factor of 10 for BDP FL azide **18**. Unprocessed images are presenting the raw data (Figure S20, S21).

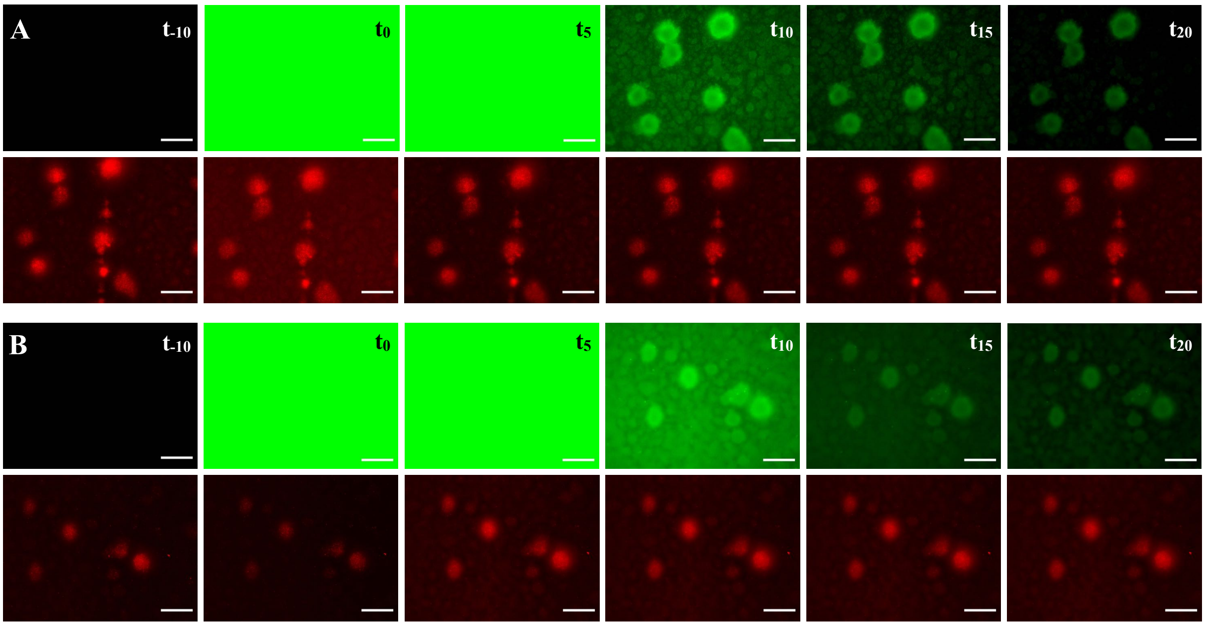

Figure S21: Staining of *P. aeruginosa* PAO1 biofilms in the flow system following the injection method (raw data without normalization to different fluorescence intensities). Fluorescent compound (stocks of 500 µM **17** or **24**) was injected into the tubings upstream of the flow cell channel resulting in a final concentration of 17 µM within the channel. Pictures of same aggregates were recorded before compound injection (t_{-10}), during 10 min incubation period with no flow (t_0) and during the wash period (constant flow, 3 mL/h) at different time points t_5, t_{10}, t_{15}, t_{20} (5, 10, 15 and 20 min) using a fluorescence microscope. The green signal corresponds to the fluorescently labelled divalent LecA ligand **17** (A) or to the fluorescein control **24** (B). *P. aeruginosa* expressing mCherry from pMP7605[23] is shown in red. Scale bars = 100 µm.

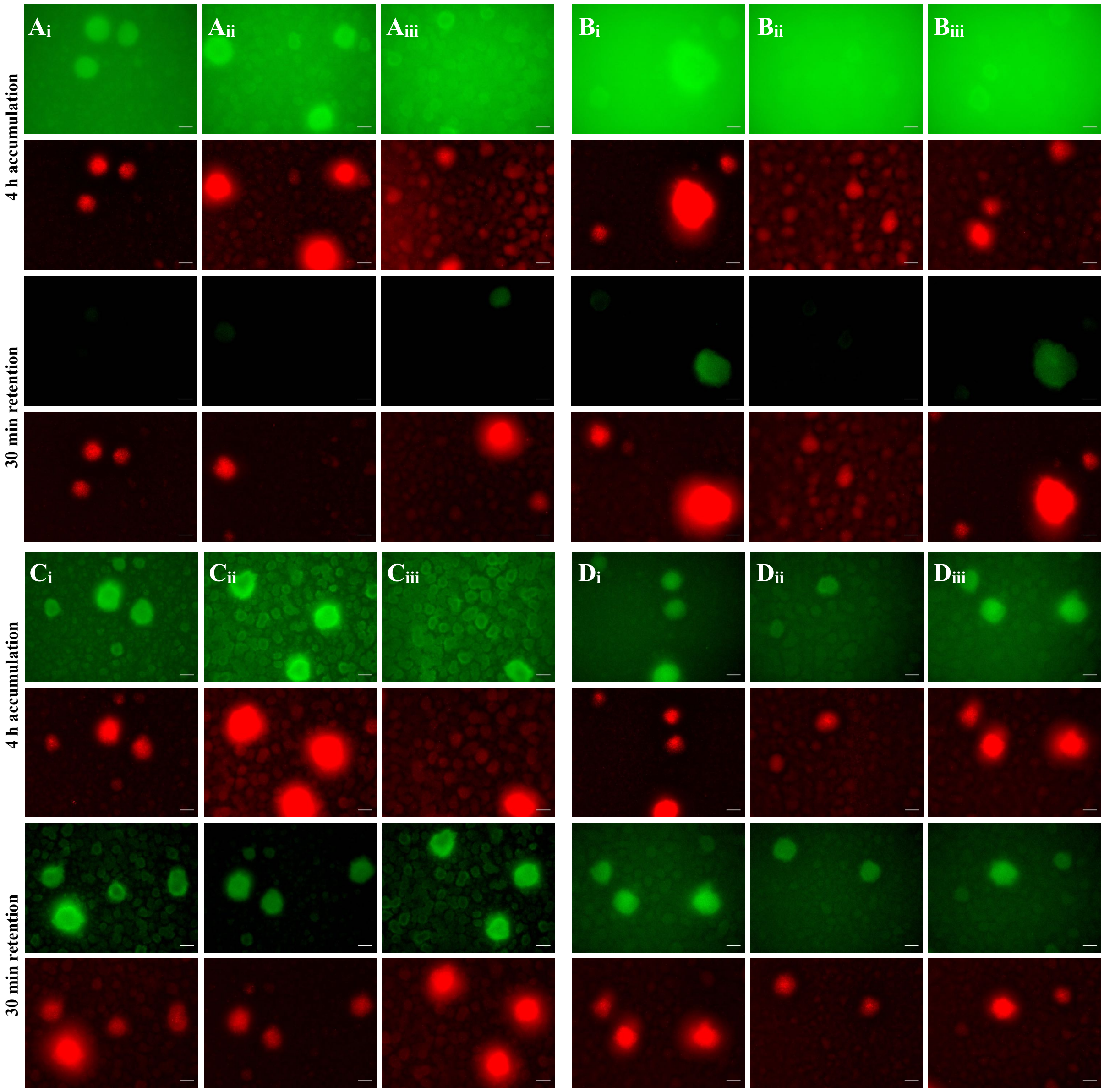

Figure S22: Staining of *P. aeruginosa* PAO1 biofilms in the flow system following compound supplementation into the medium (raw data without normalization to different fluorescence intensities). Biofilm staining with (A) fluorescein divalent imaging probe **17**, (B) fluorescein control **24**, (C) BODIPY divalent imaging probe **19** and (D) BDP FL azide control **18** was observed after 4 h of constant flow (3 mL/h) of LB media containing 500 nM of respective compound (accumulation period). Several snapshots of biofilm aggregates within one channel were recorded ($_{i\text{-}iii}$). Retention of imaging compounds is shown after 30 min wash period with medium (3 mL/h, no compound). Pictures were recorded using fluorescence microscopy and green signal correspond to fluorescently labelled divalent LecA imaging probes (**17** and **19**) and control compounds (**24** and **18**). *P. aeruginosa* expressing mCherry from pMP7605[23] is shown in red. Scale bars = 50 µm.

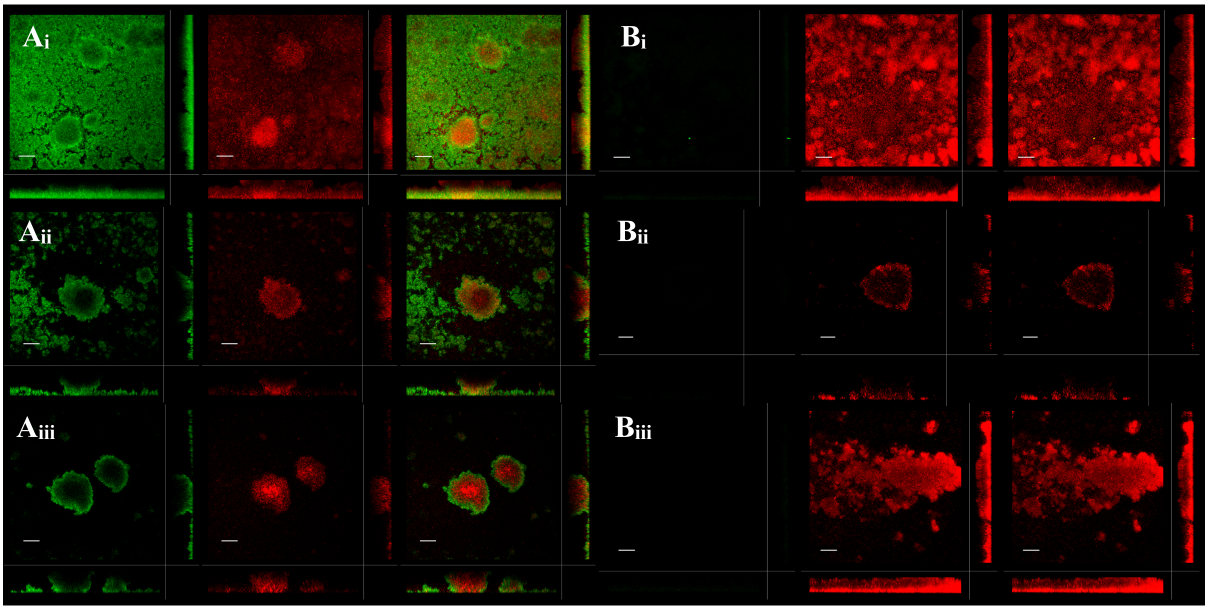

Figure S23: Staining of *P. aeruginosa* PAO1 biofilms in the flow system following compound supplementation into the medium and analysis by CLSM. *P. aeruginosa* PAO1 labelled with the red fluorescent protein mCherry (pMP7605)[23] was grown in a flow system. 500 nM of the (A) BODIPY conjugated LecA imaging probe **19** and the control (B) BDP FL azide **18** were constantly added to the biofilms via the flow system for 4 h. CLSM was performed for analysis. Z-Stacks were recorded for each channel ($_{i-iii}$). Green signals correspond to the BODIPY conjugated imaging probe (**19**) and the control BDP FL azide (**18**), respectively. Red signals correspond to mCherry protein (pMP7605).[23] Z-Stack size, 2 µm. Scale bars = 50 µm.

Lung infection model and *in vivo* imaging of *P. aeruginosa*

C57BL/6J and albino (Tyr^{c2J}) C57BL/6 mice were bred and housed at the Central Animal Facility, Hannover Medical School, Germany. Animal experiments were performed with permission number: 33.9-42502-04-16/2072 from the Lower Saxony State Office for Consumer Protection and Food Safety (LAVES), Germany. Mice were anesthetized with 3% isoflurane (CP Pharma) and infected intratracheally with $2x10^6$ cfu/50 µL of *P. aeruginosa* PAO (DSM1707, Braunschweig, Germany) as described previously.[26]

50 – 100 nmol sulfo-Cyanine7-compound (divalent LecA probe **21**; LecB probe **22**; control sulfoCy7 free acid, CAS no.: 2104632-30-4, Lumiprobe; control sulfoCy7 azide, **20**, Lumiprobe) per 20 g mouse was injected intravenously 2 h after infection. Optical imaging was performed 4 – 7 h after compound administration. Mice were anesthetized with 3% isoflurane and fluorescence (ex/em [nm]: 640/680, 700, 720, 740; 675/720, 740, 760) was recorded with an *in vivo* imaging system (IVIS SpectrumCT, PerkinElmer). Subsequently mice were euthanized and organs were imaged (675/760 nm) *ex situ* individually. To enhance Cyanine7 specific fluorescent signal spectral unmixing was performed on *in vivo* images using LivingImage 4.7 (PerkinElmer).

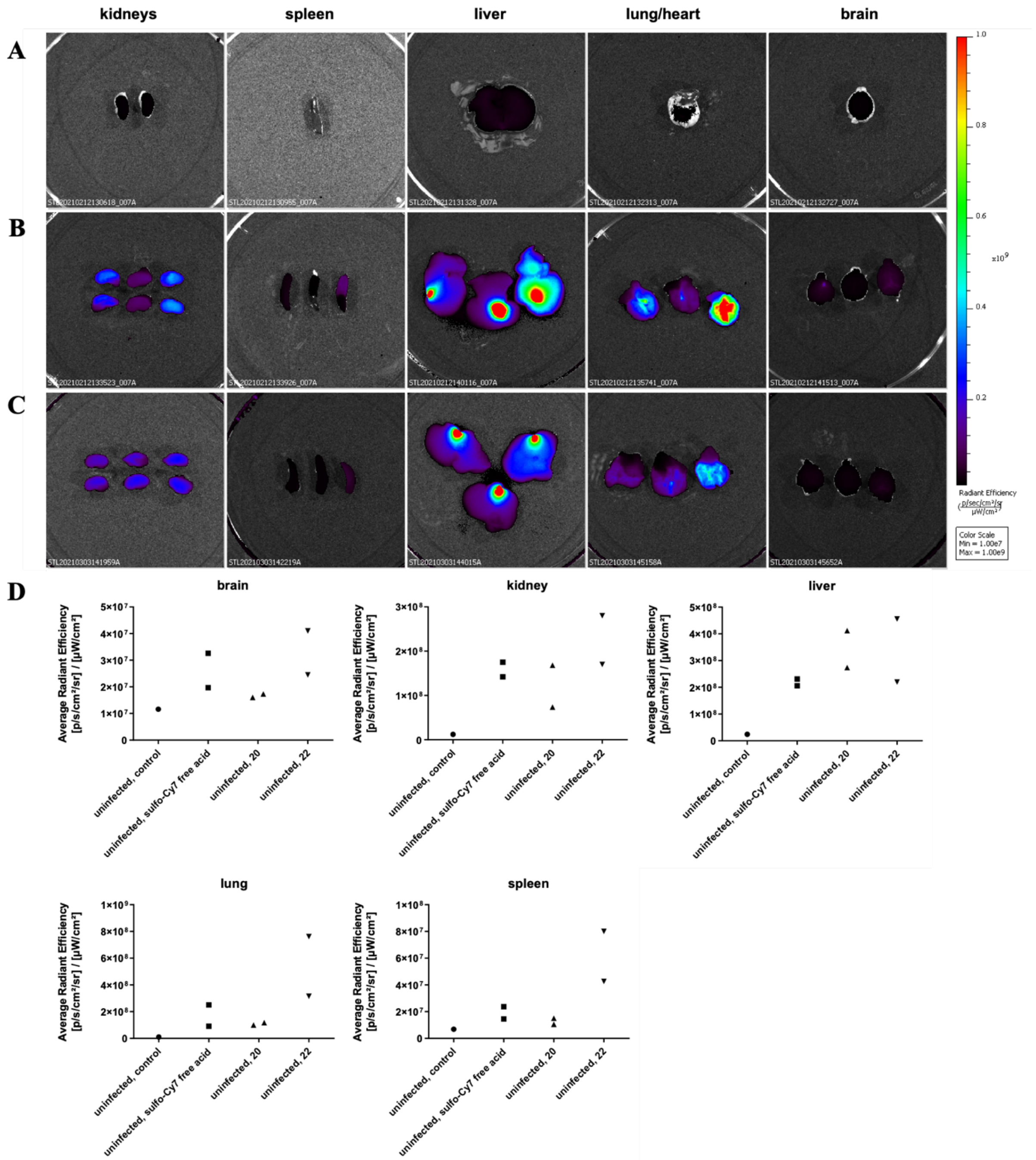

Figure S24: ***Ex situ* analysis of different organs of uninfected mice.** Intravenous injection of 50 nmol/20 g: (A) Control, no dye; (B) and (C) sulfo-Cy7 free acid (left), sulfo-Cy7 azide **20** (middle) and LecB probe **22** (right). Fluorescent intensities are shown as false color pictures. (D) Quantification analysis in average radiant efficiency.

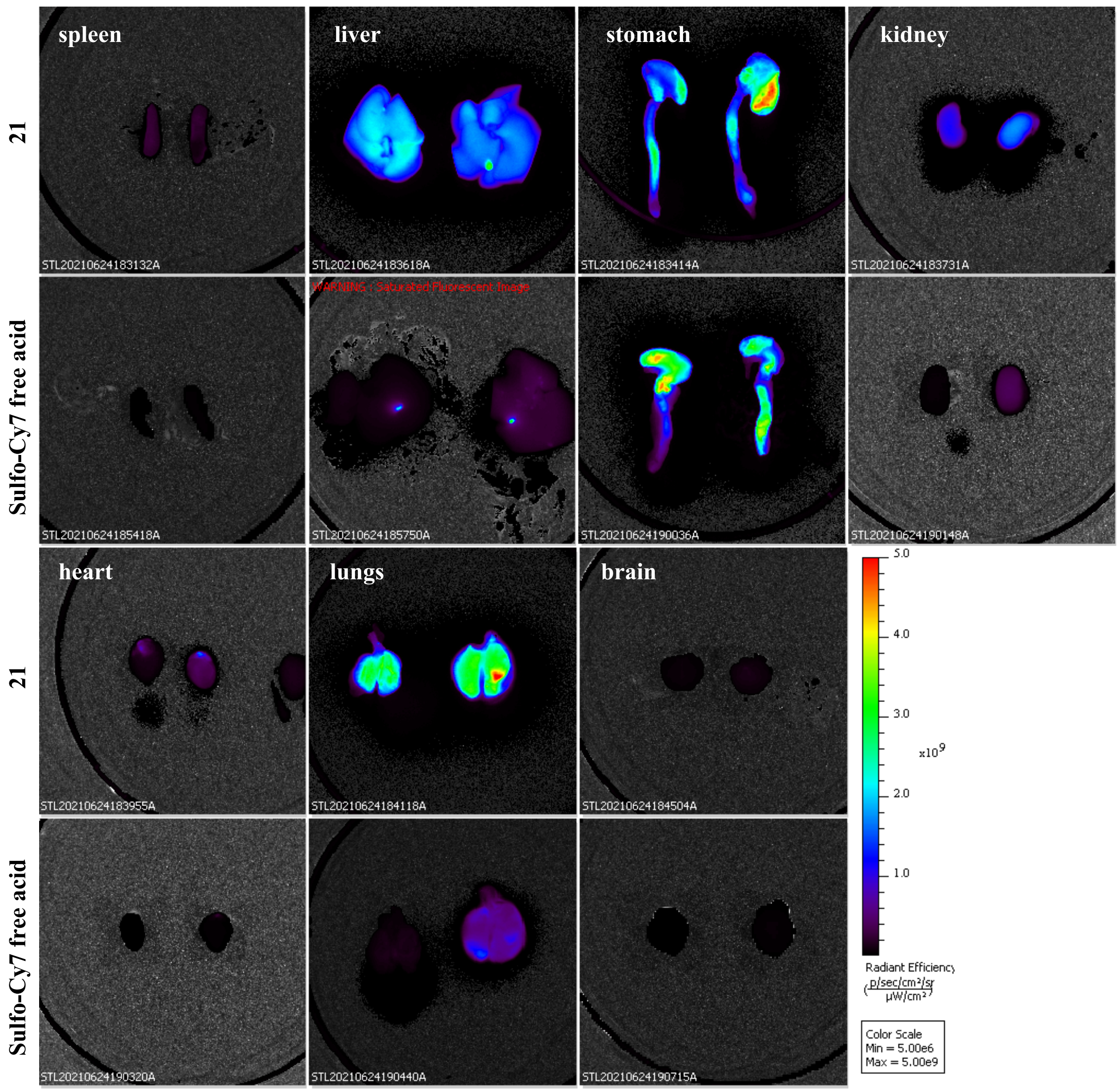

Figure S25: *Ex situ* analysis of different organs after acute lung infection with *P. aeruginosa* PAO. Imaging probe **21** and sulfo-Cy7 free acid as control (50 nmol/20 g) were injected i. v. 2 h after intratracheal infection with PAO (right) or into uninfected control mice (left). Fluorescent intensities are shown as false color pictures.

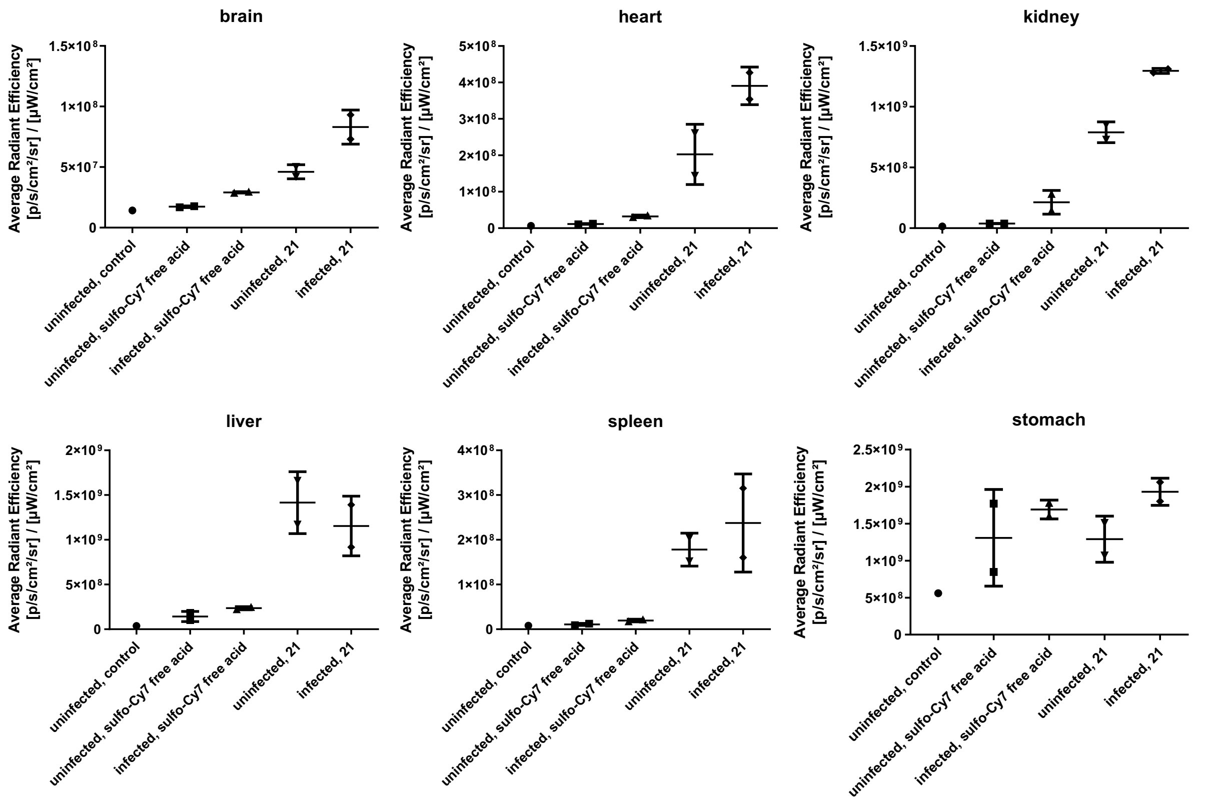

Figure S26: Quantification analysis in average radiant efficiency of different mice organs after lung infection with *P. aeruginosa* PAO. Divalent LecA targeting imaging probe **21** and control sulfo-Cy7 free acid (50 nmol/20 g) were injected intravenously 2 h after intratracheal infection of mice with *P. aeruginosa* or to uninfected control mice. ROI analysis in average efficiency was performed for different organs.

Lung sections and *ex vivo* imaging of *P. aeruginosa*

Sections of 8 µm were made from the frozen lung lobes using a cryostat (Leica, Germany). The sections were mounted on adhesive slides, dried overnight at room temperature and mounted with a DAPI containing gel. Confocal imaging was performed on a Leica SP8 STELLARIS system equipped with a super-continuum white light laser (WLL) and a multi-photon laser source (MP) through a water immersion objective (Leica HC FLUOTAR L 25x/1,0 with a motorized correction ring). Images (1024x1024 pixels) were acquired using the MP excitation of 720 nm and emission band (520 nm – 520 nm) for detecting DAPI and the WLL at 730 nm excitation with an emission band 748 nm – 848 nm for Cyanine7 employing non-descanned or descanned HyD detectors, respectively. Further image analysis and image overlay was performed in ImageJ2 software.[27]

NMR spectra

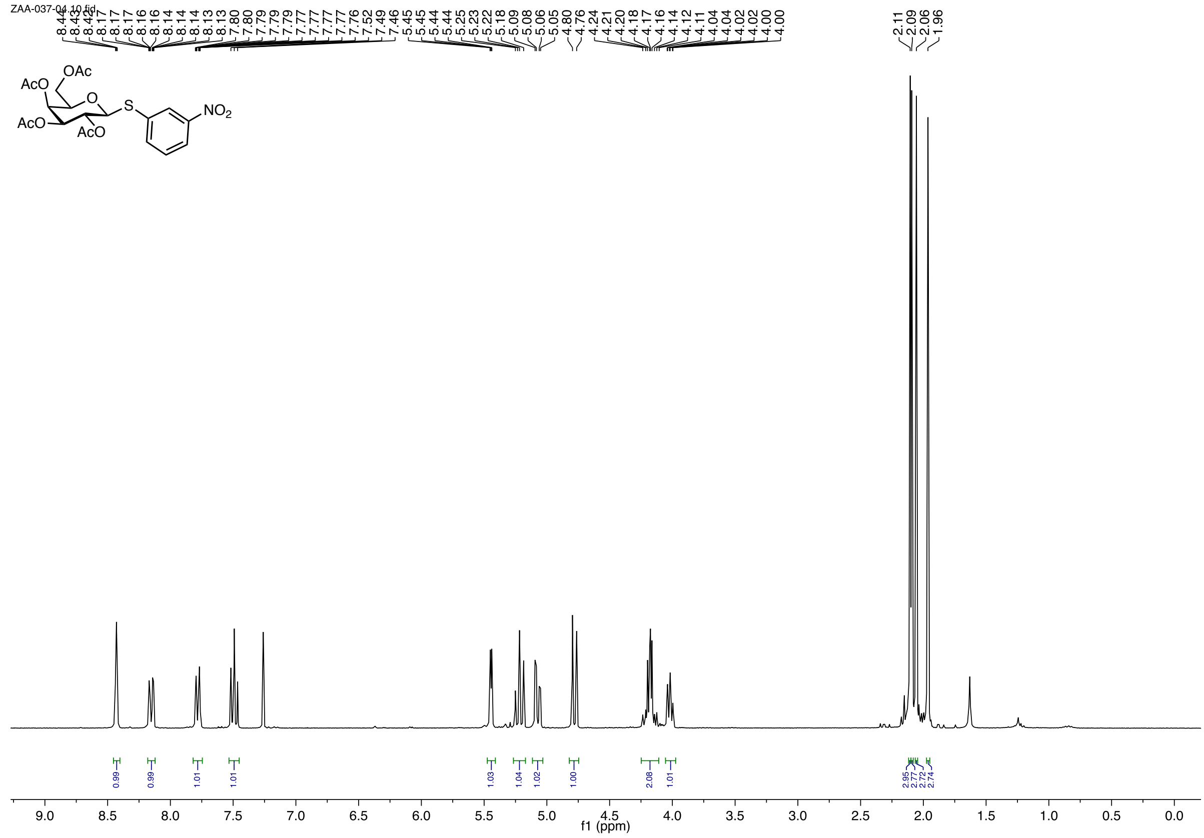

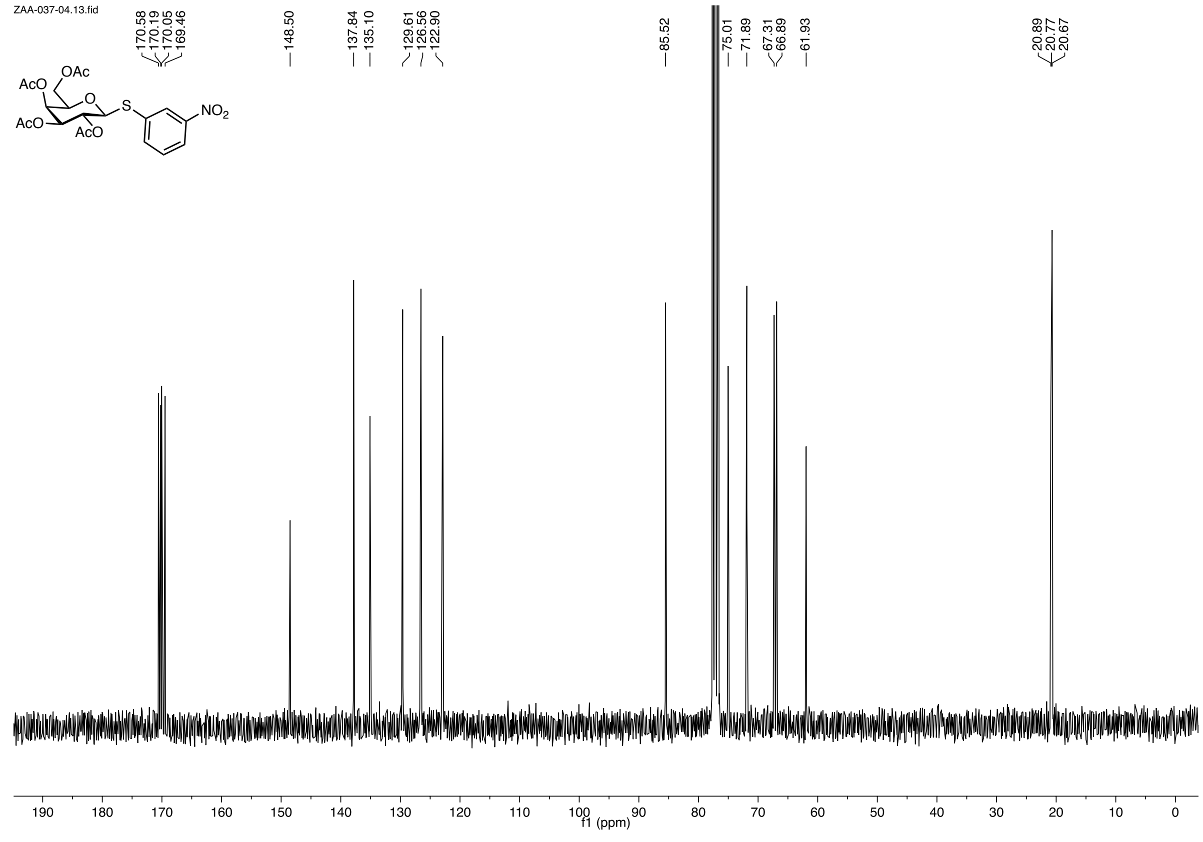

^{1}H and ^{13}C NMR of **S1*m***

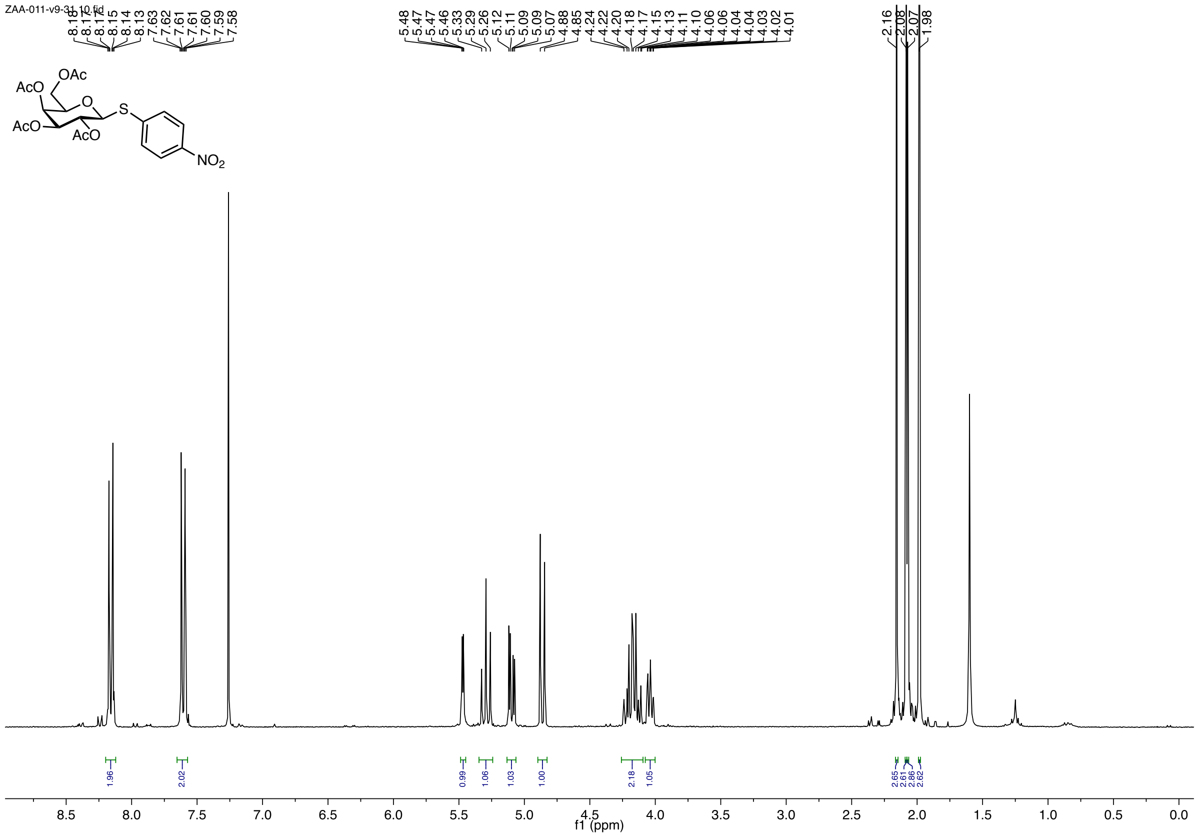

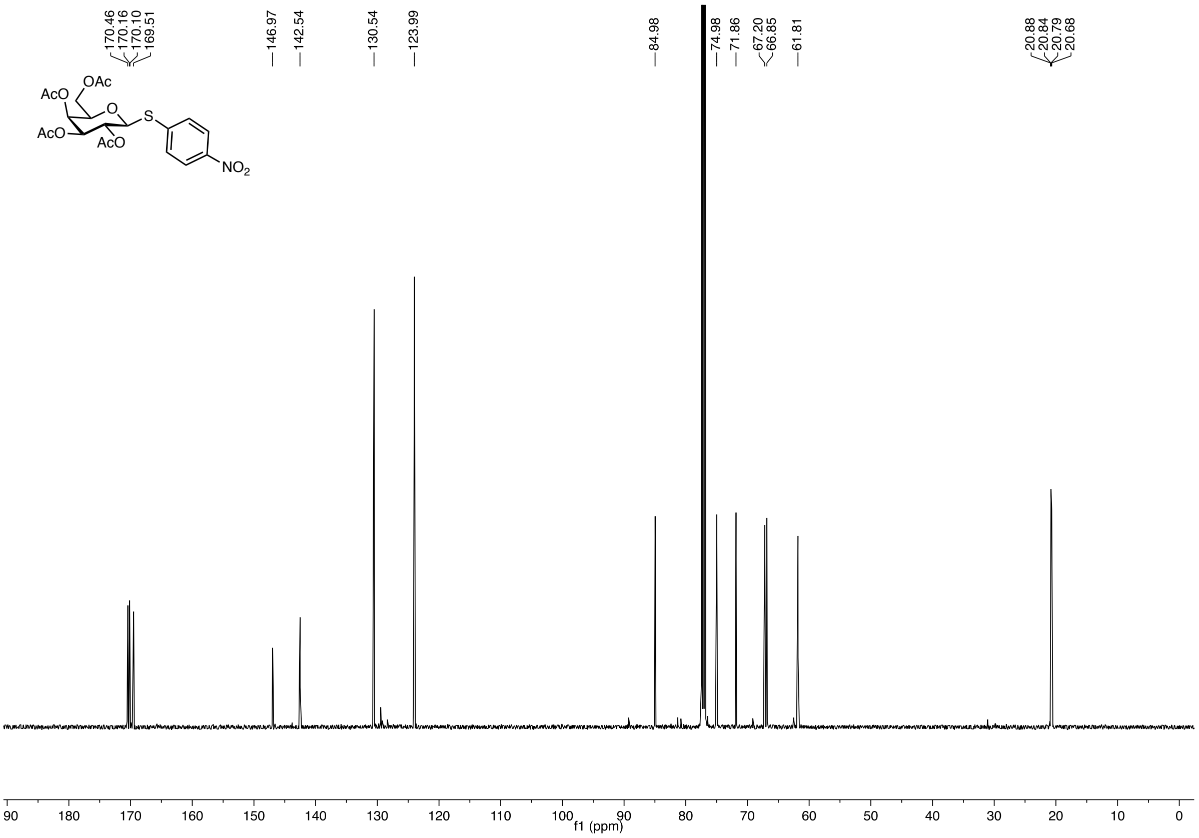

^{1}H and ^{13}C NMR of **S1*p***

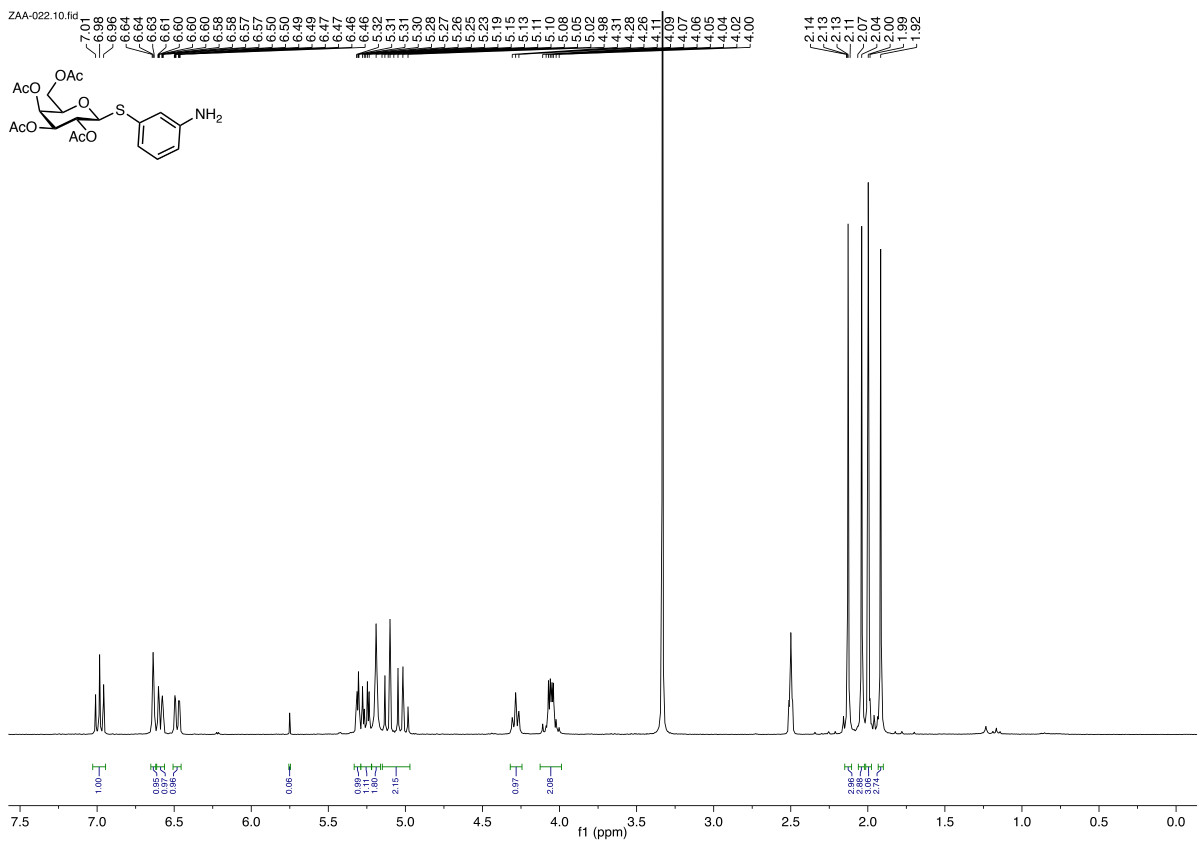

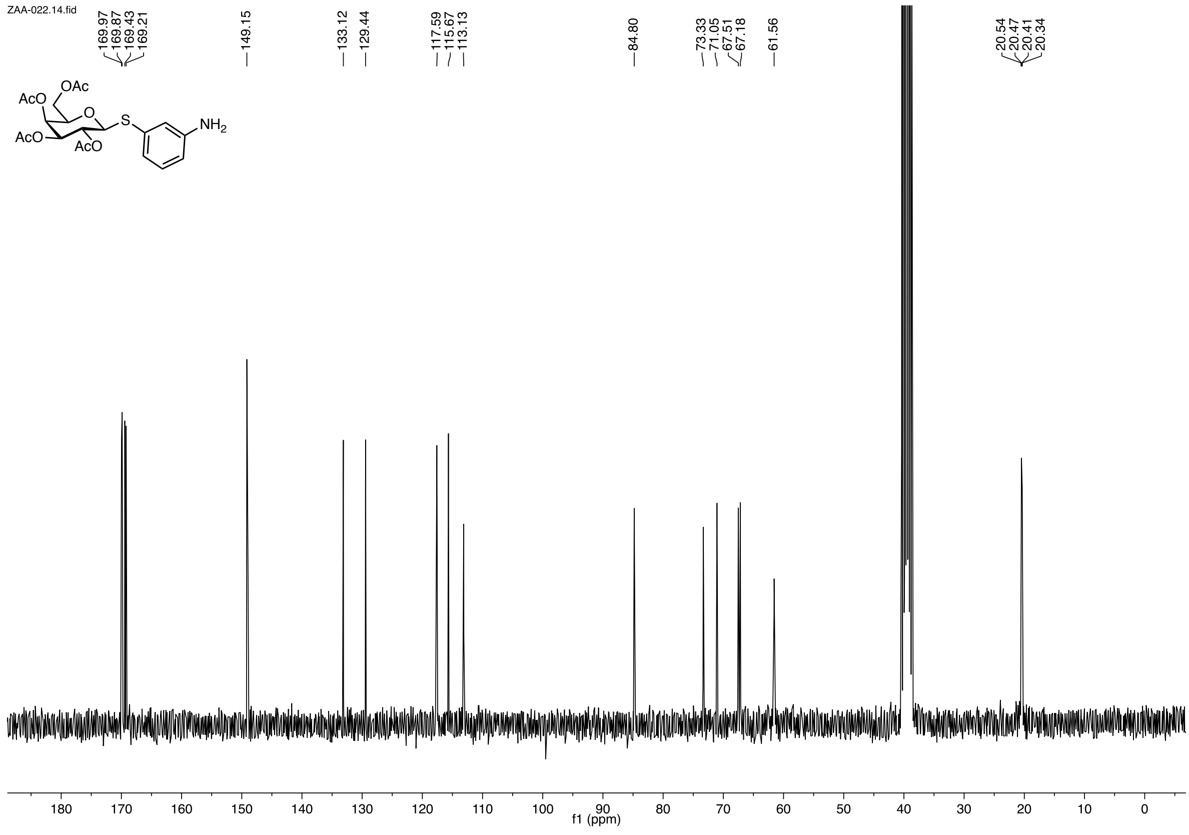

^{1}H and ^{13}C NMR of **S2*m***

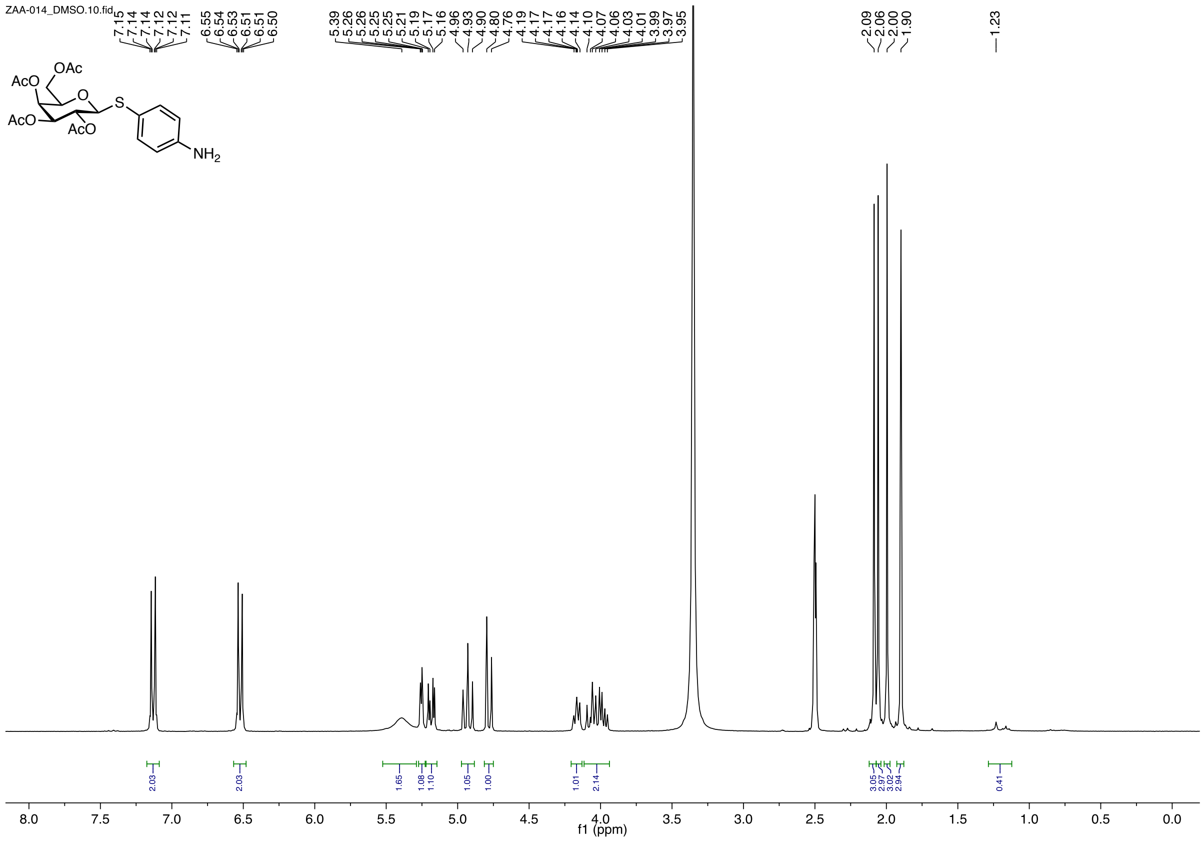

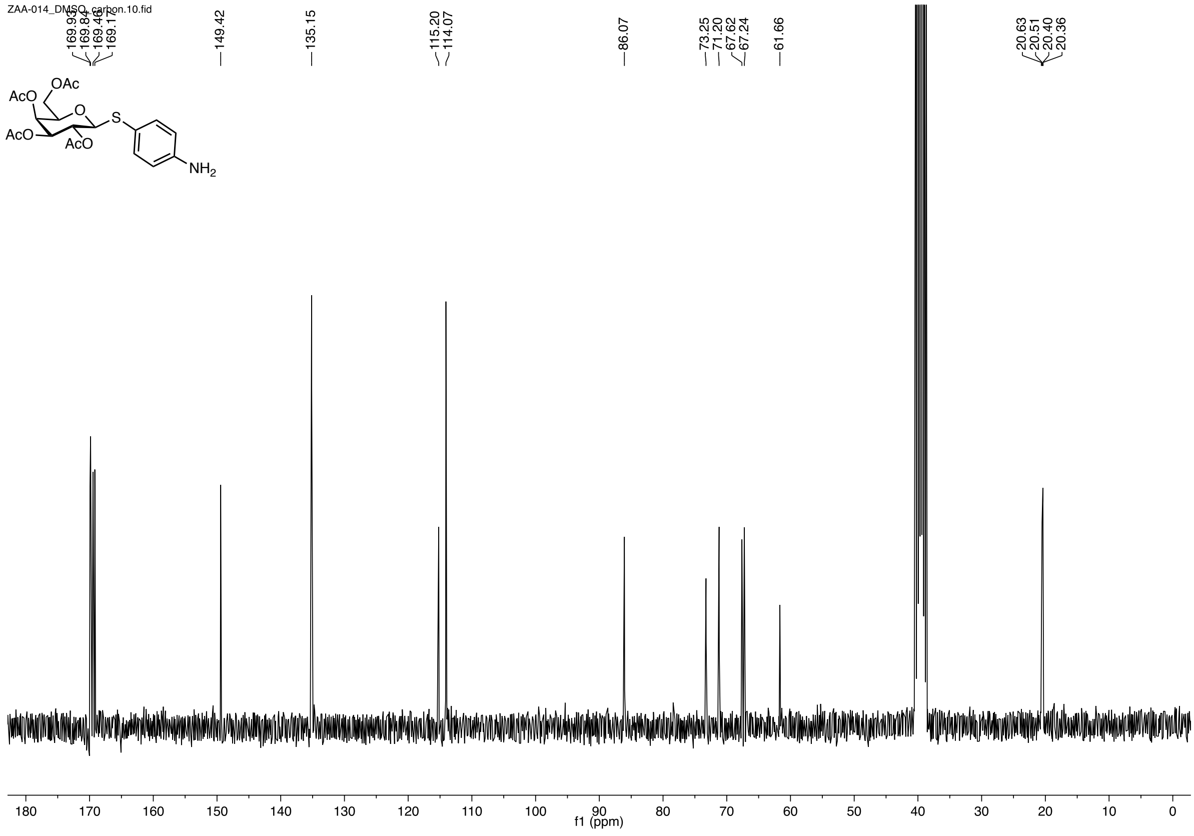

^{1}H and ^{13}C NMR of **S2*p***

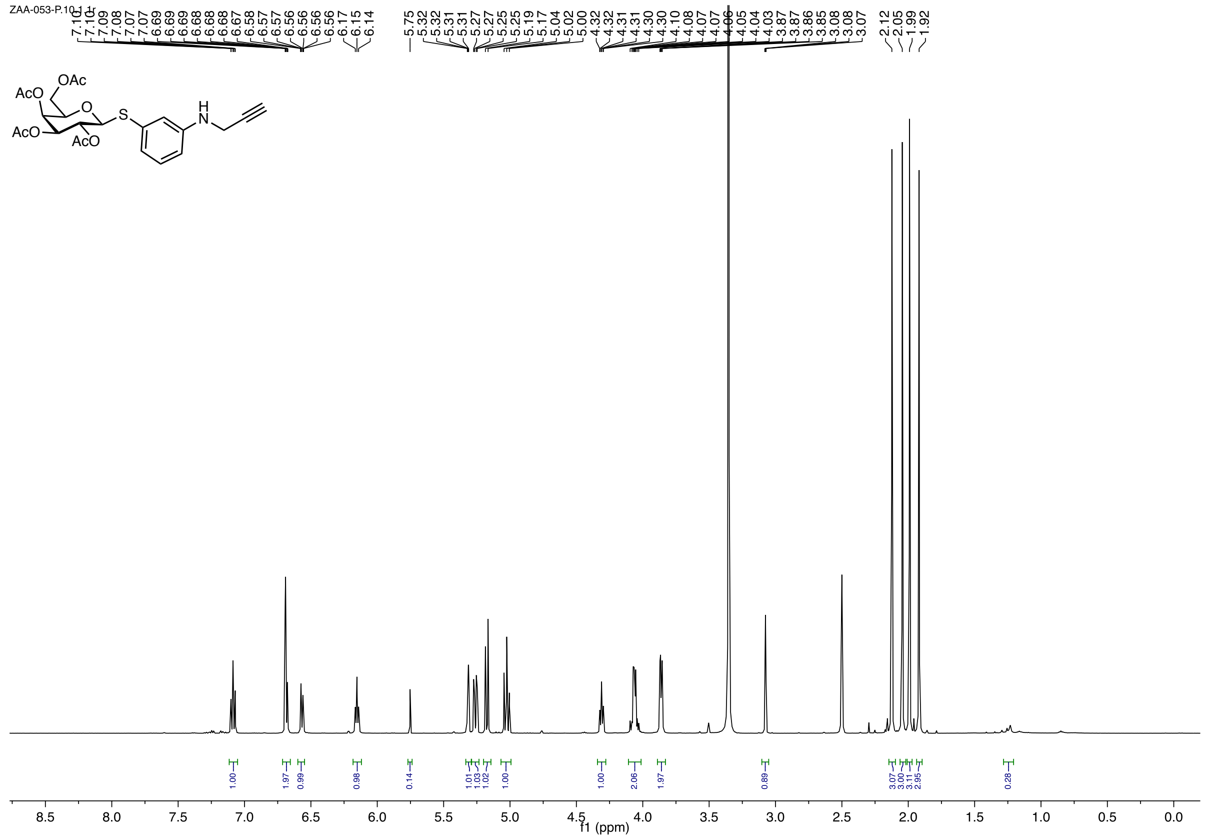

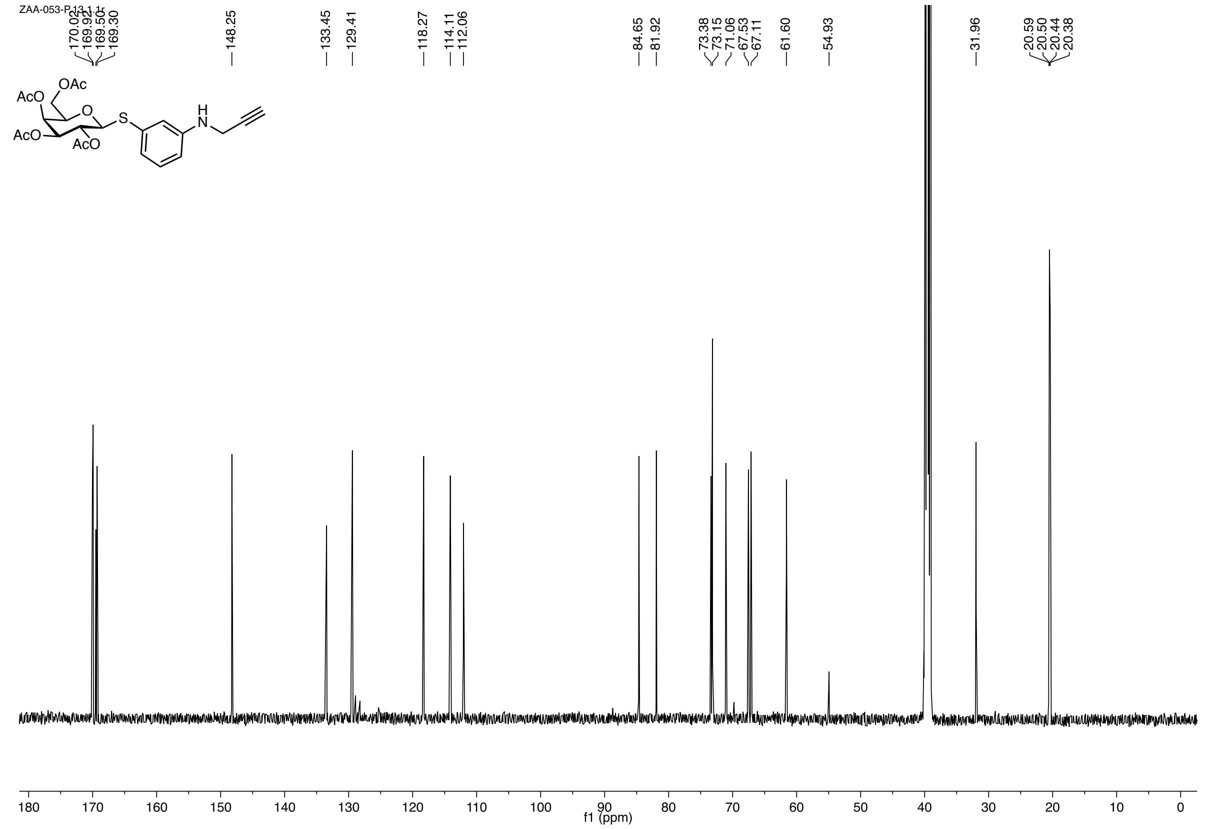

^{1}H and ^{13}C NMR of **S3*m***

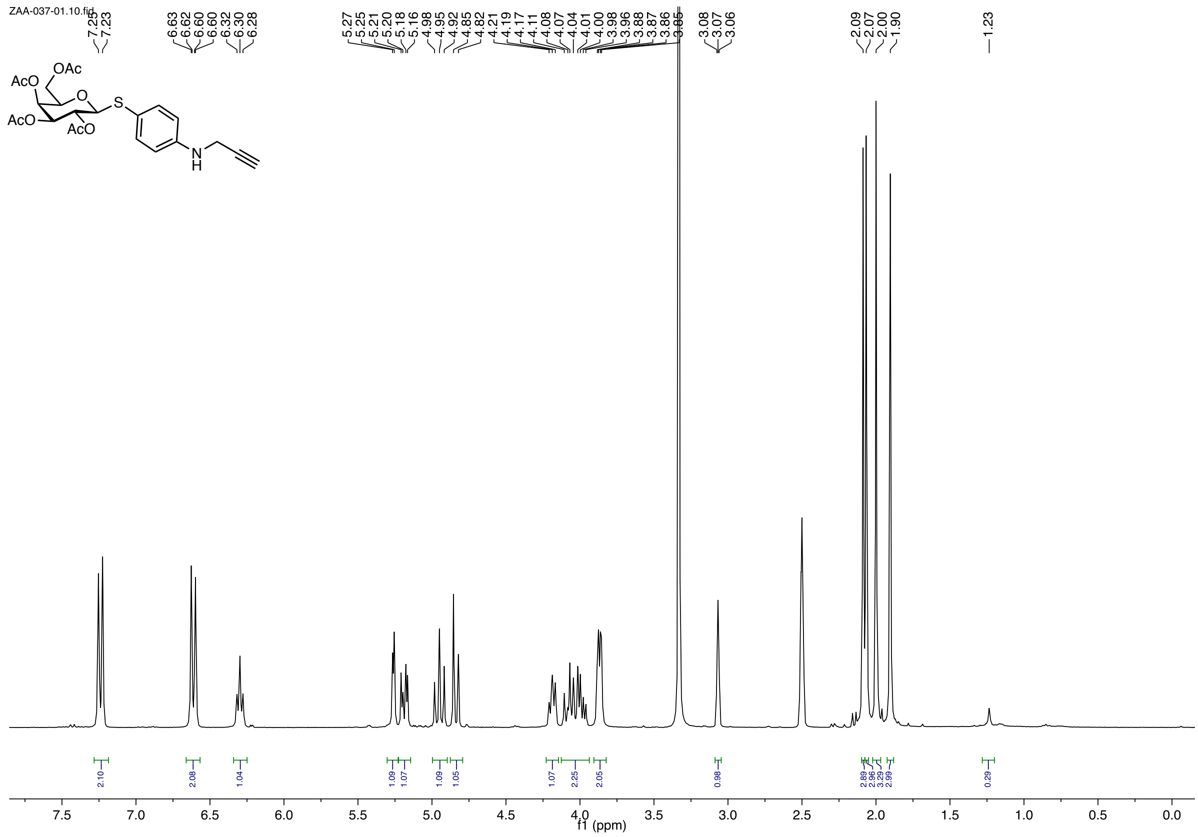

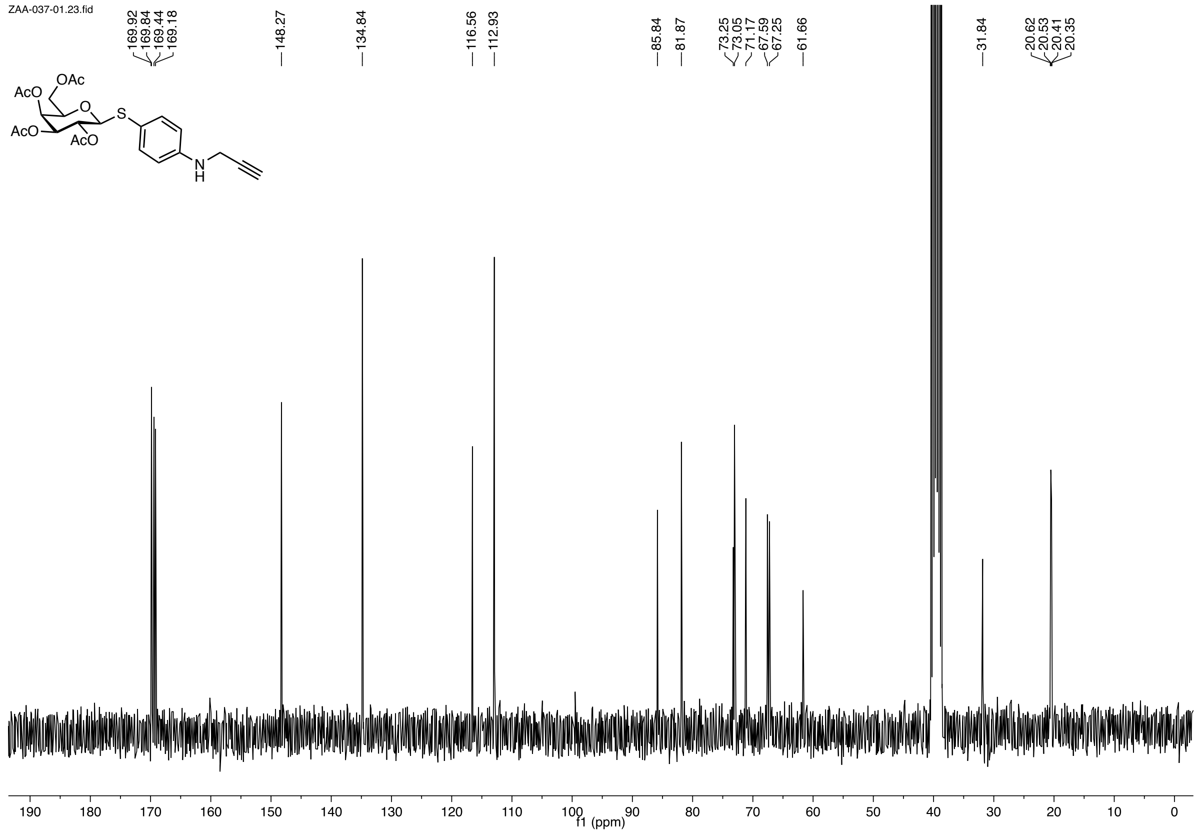

^{1}H and ^{13}C NMR of **S3*p***

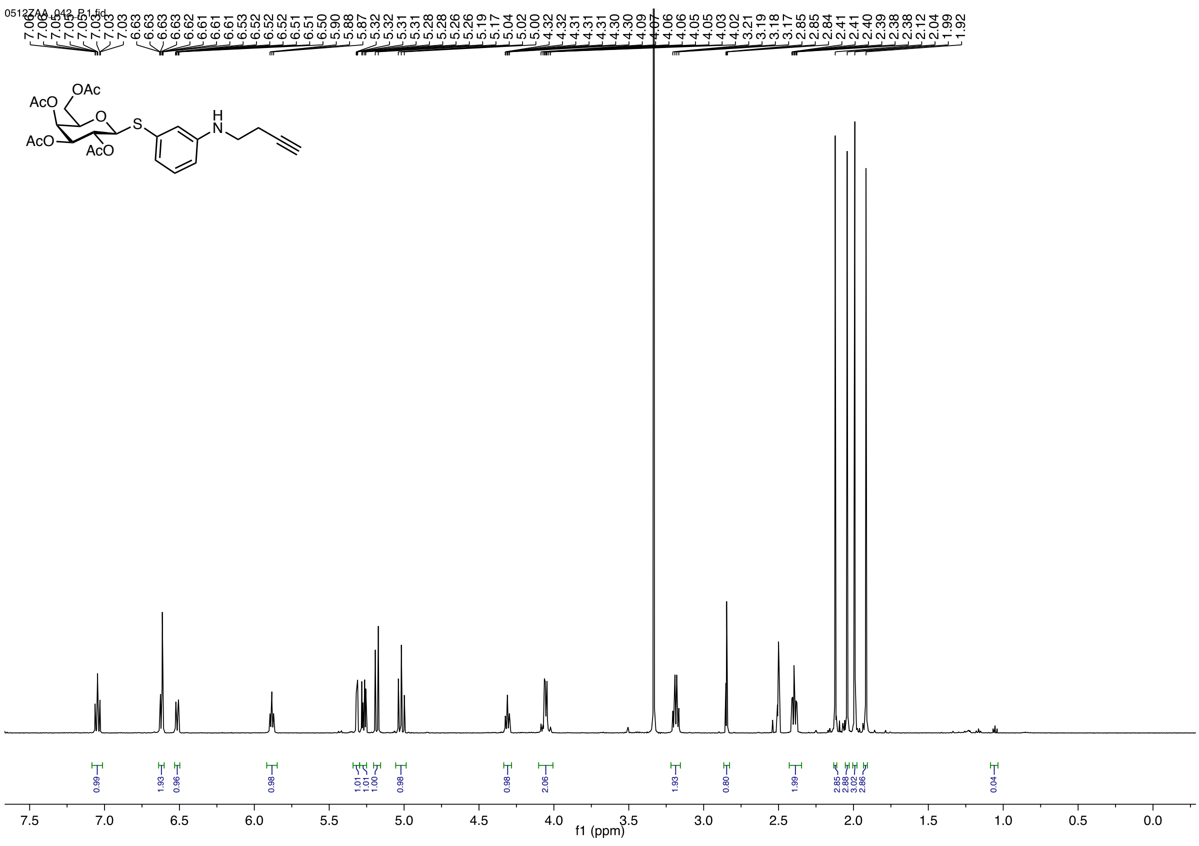

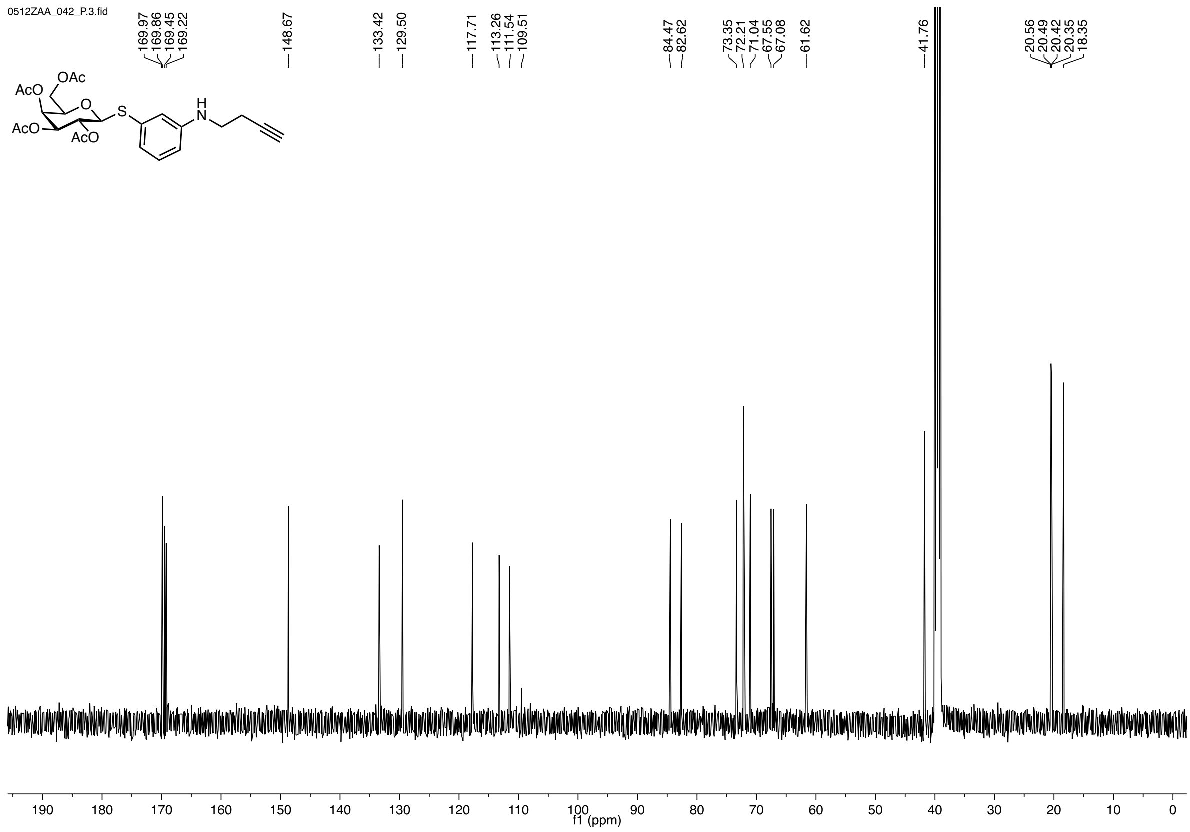

^{1}H and ^{13}C NMR of **S4*m***

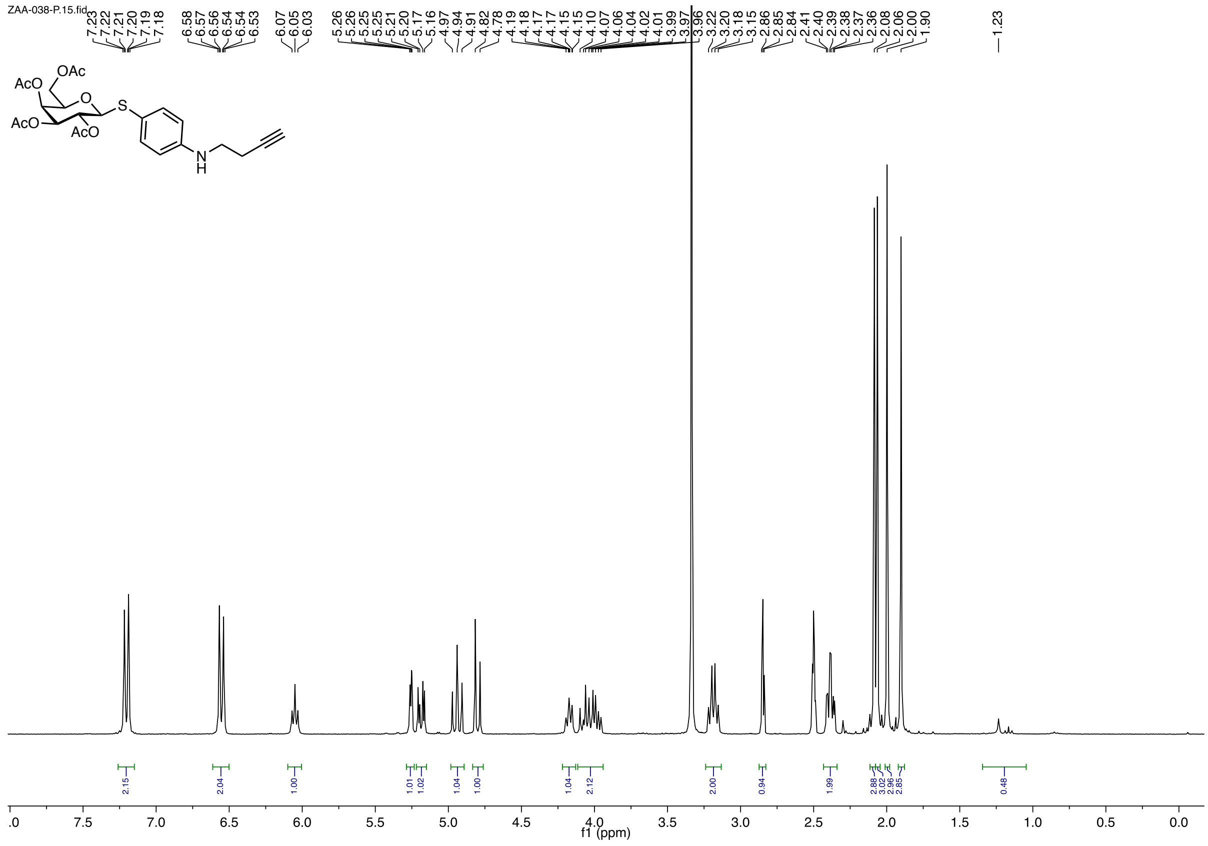

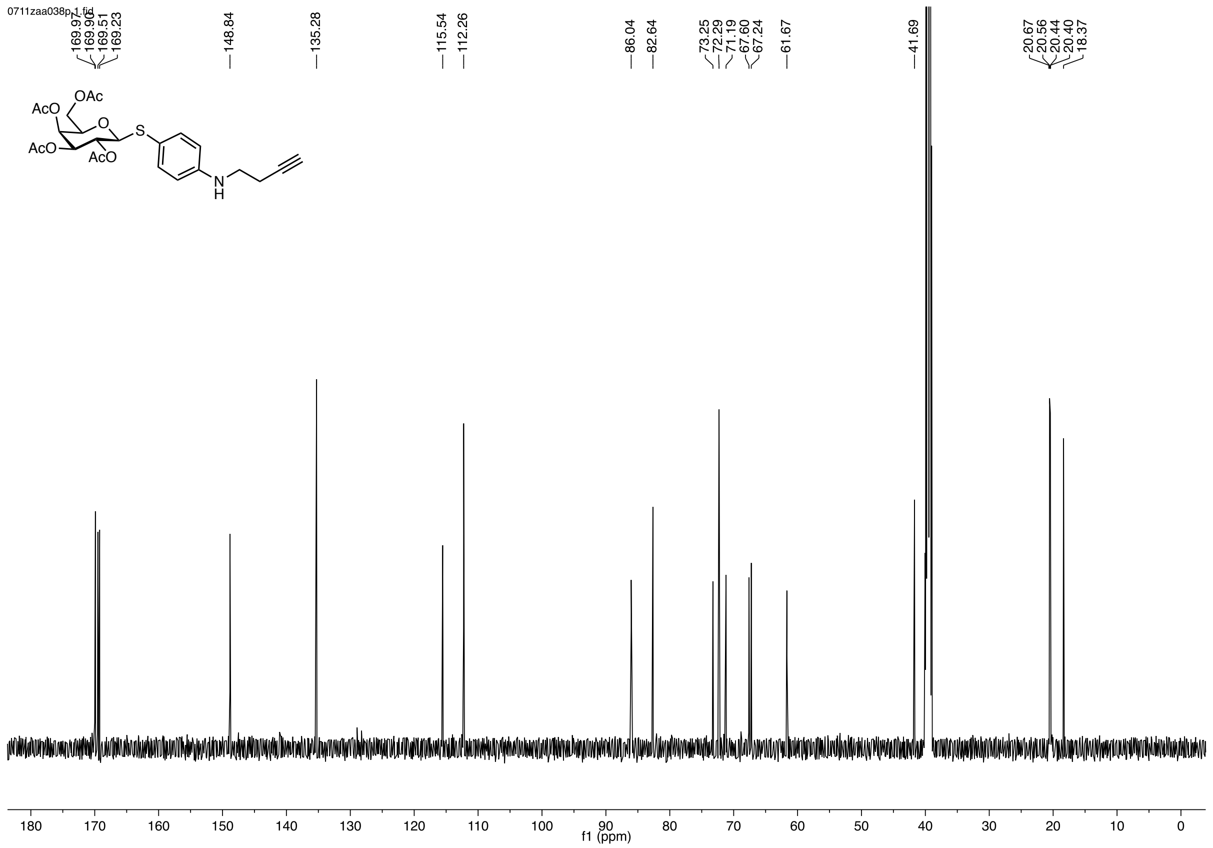

^{1}H and ^{13}C NMR of **S4*p***

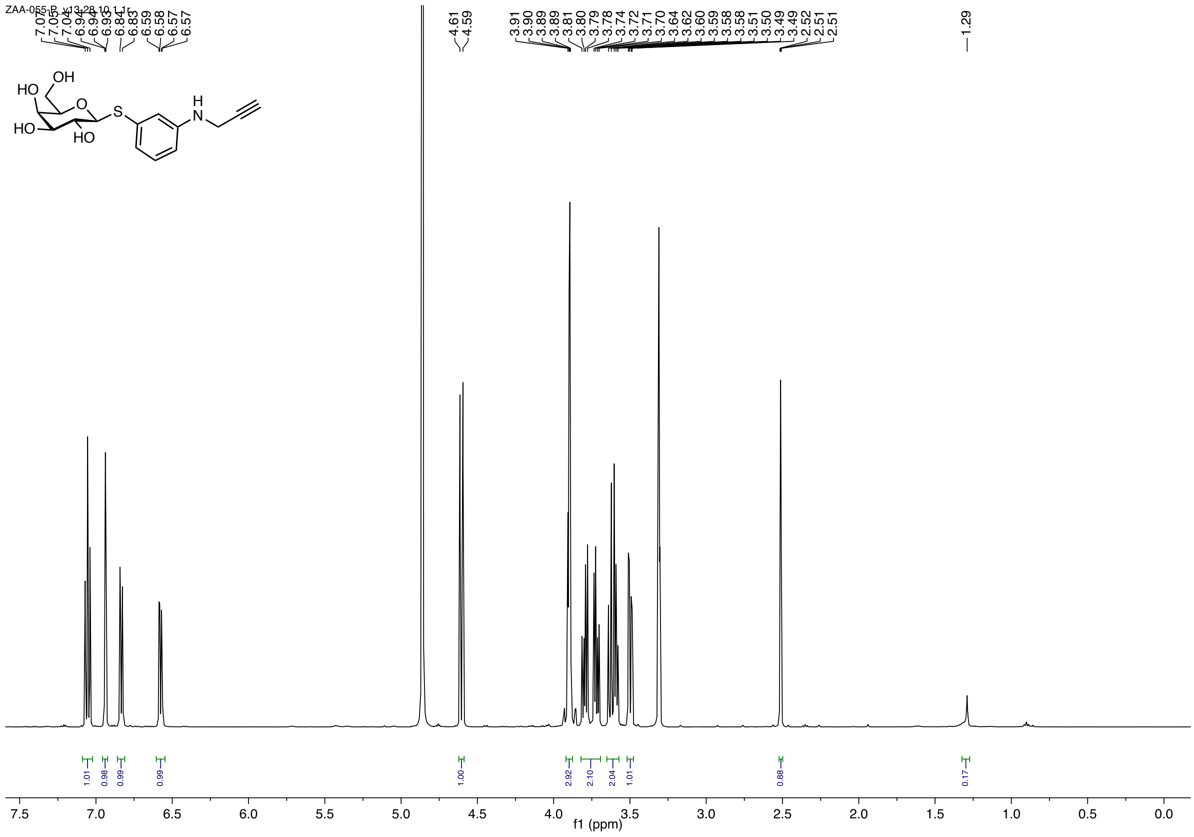

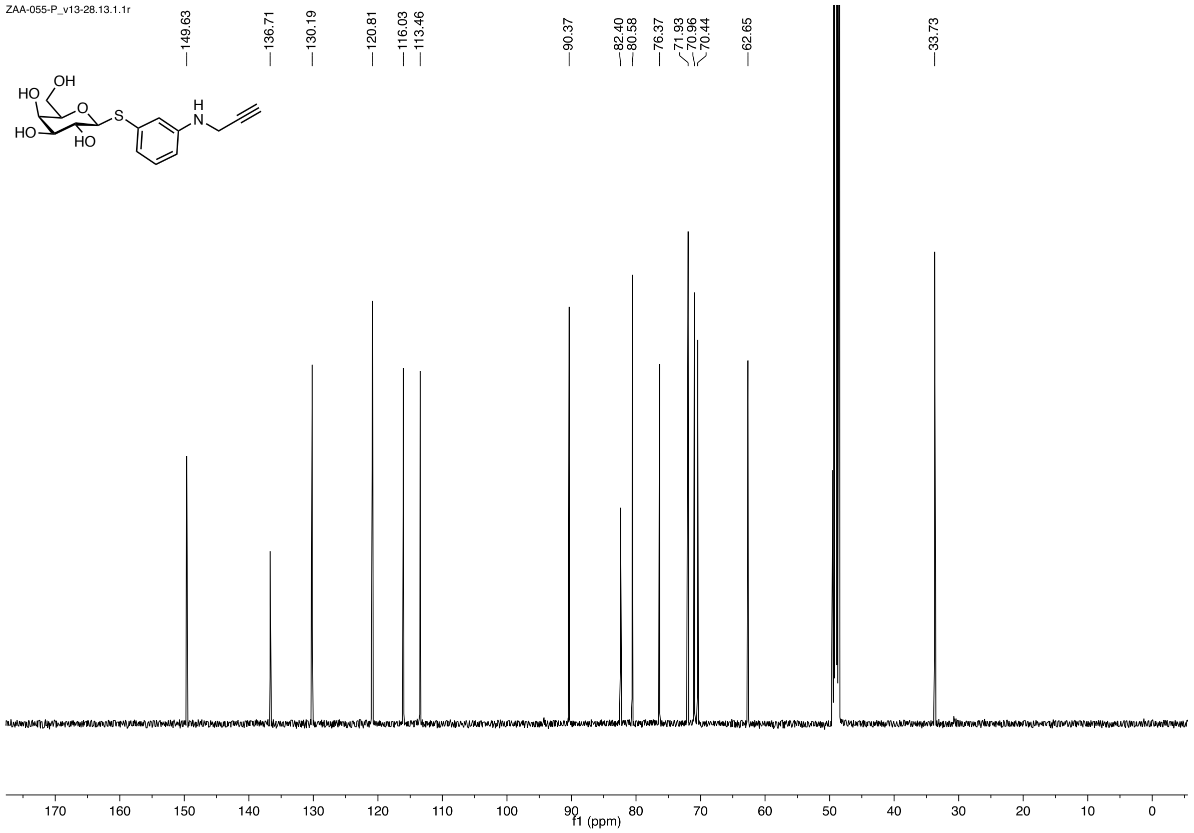

^{1}H and ^{13}C NMR of **S5*m***

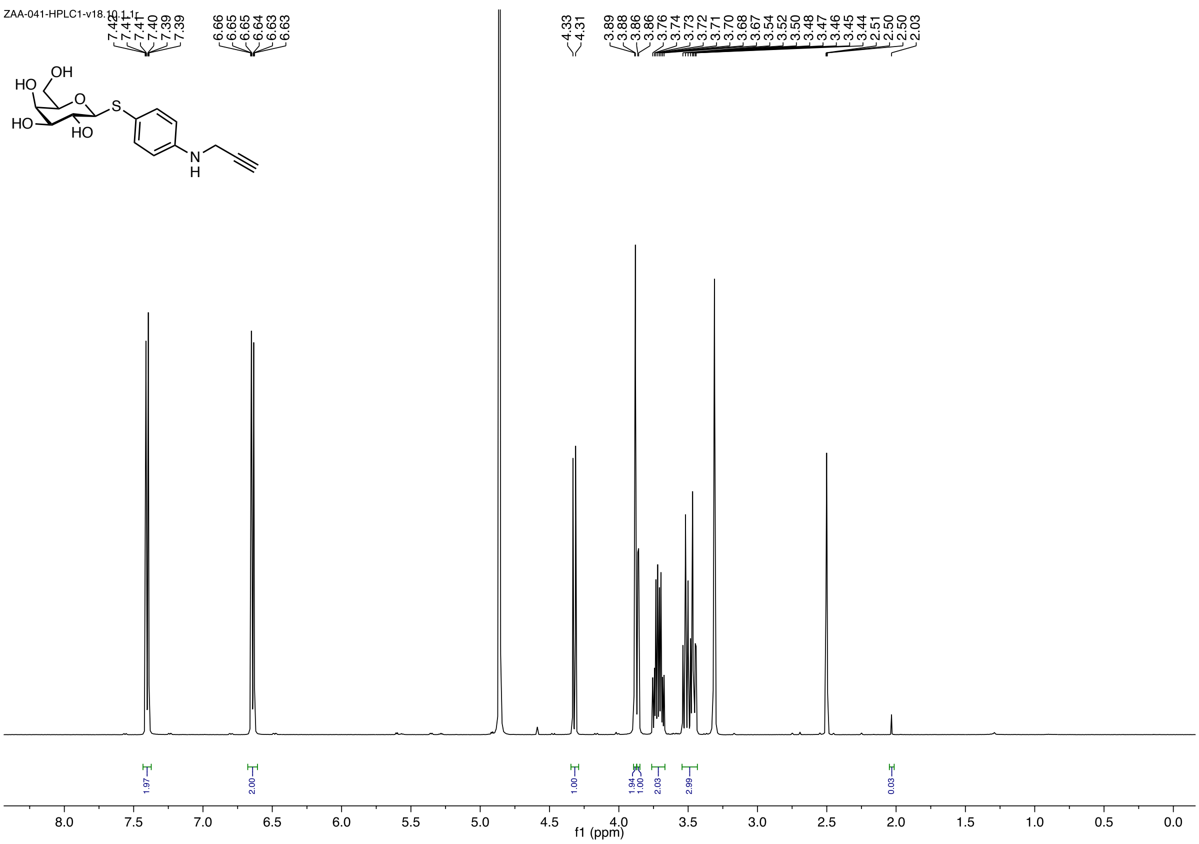

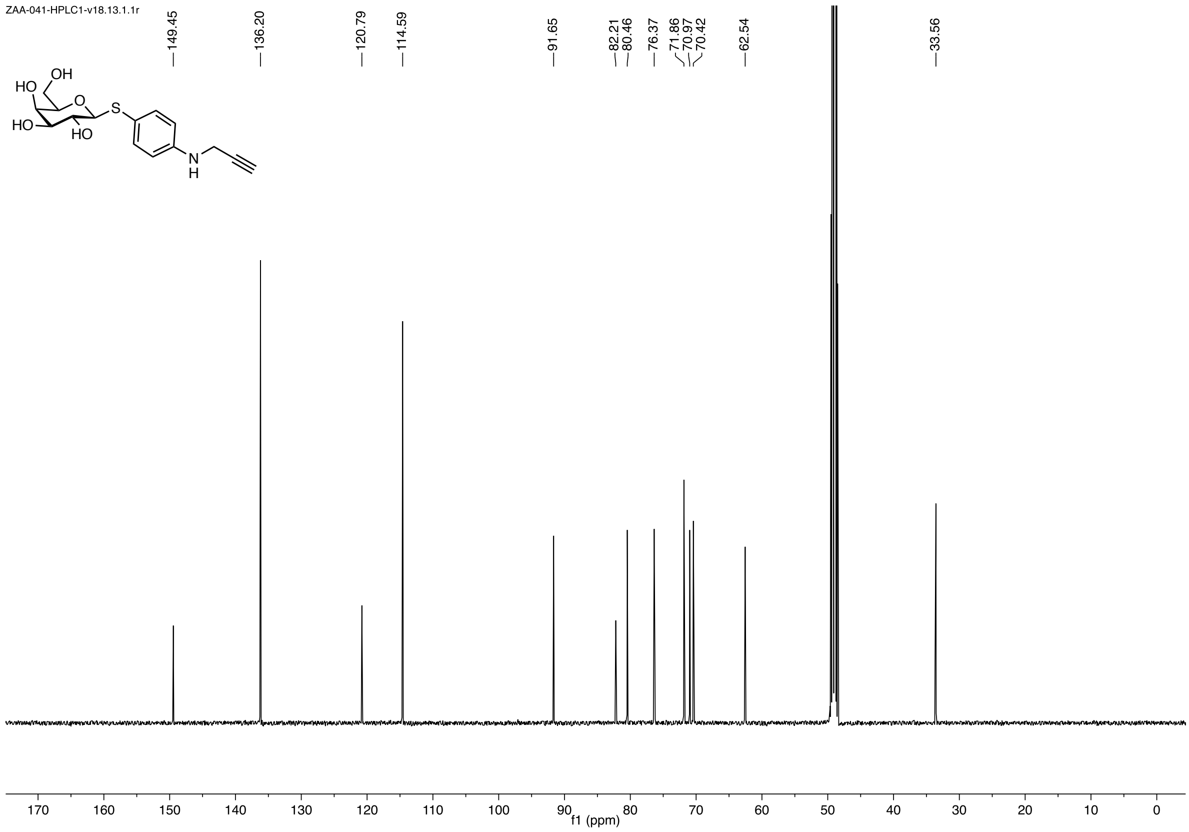

^{1}H and ^{13}C NMR of **S5*p***

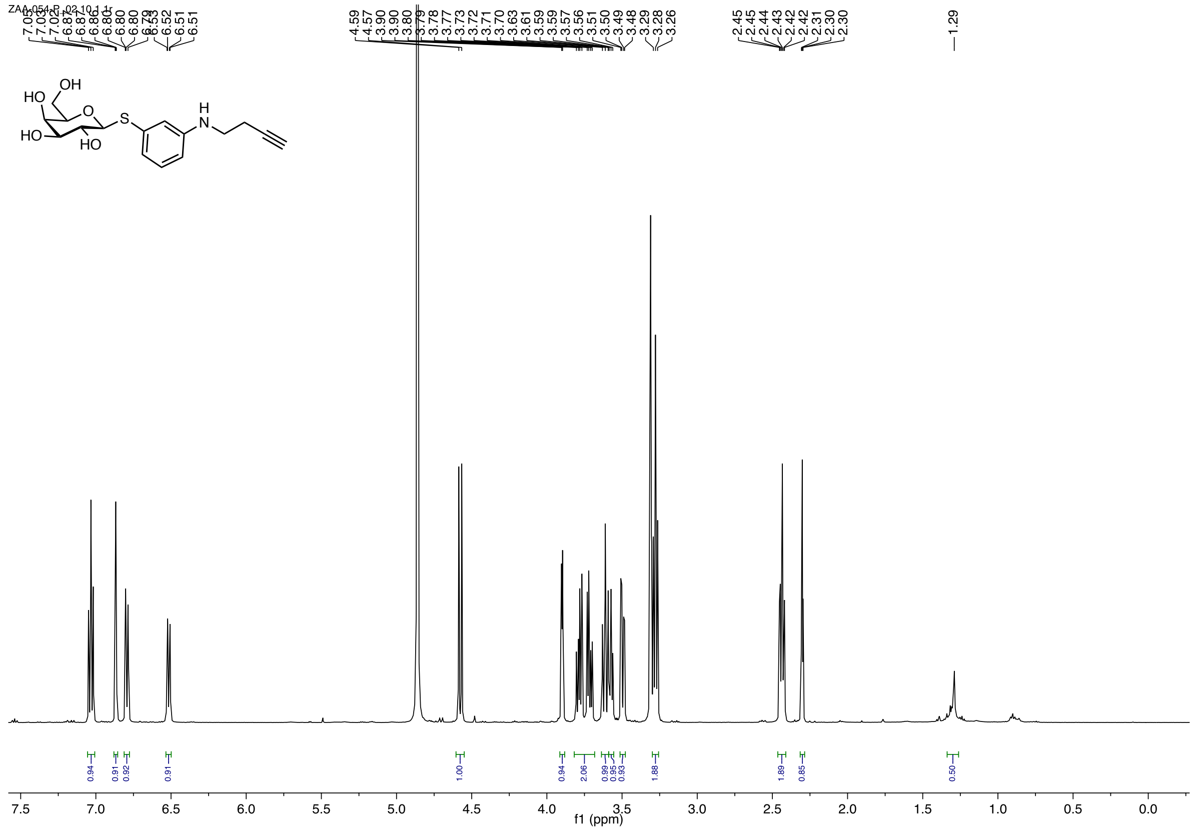

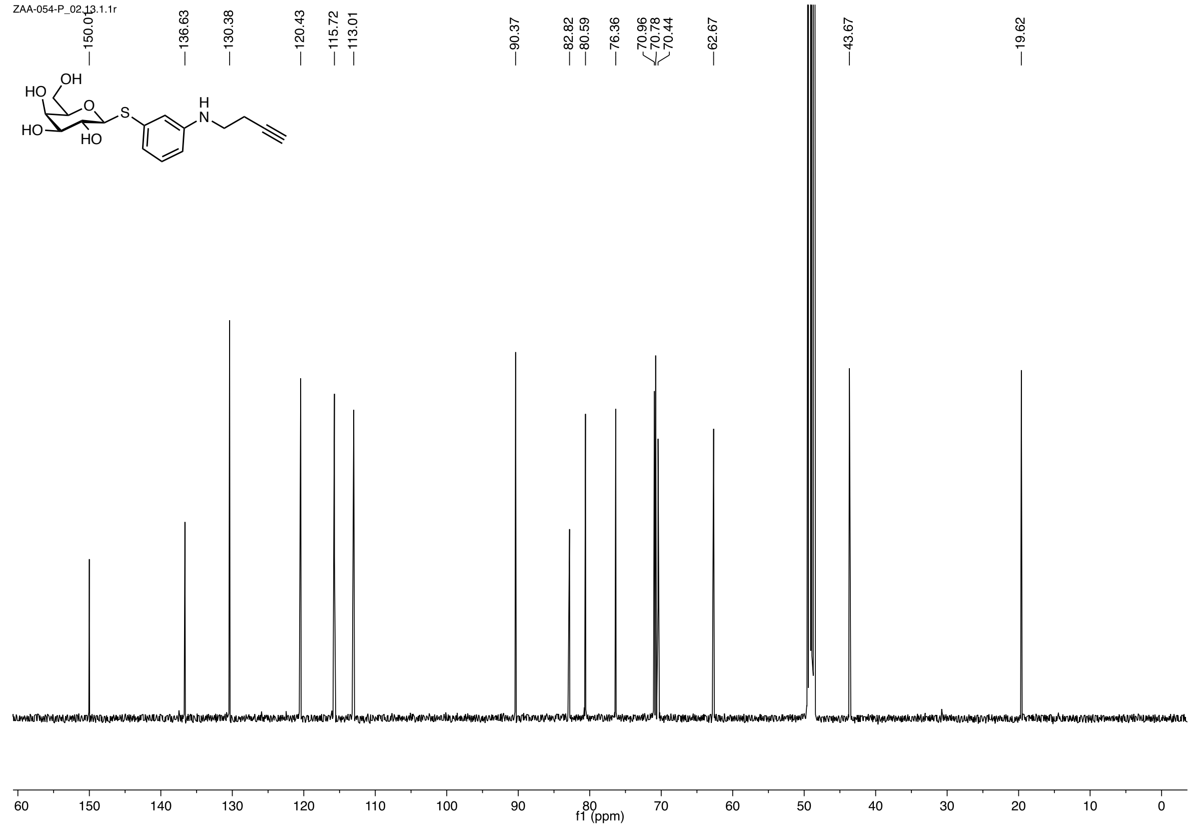

^{1}H and ^{13}C NMR of **S6*m***

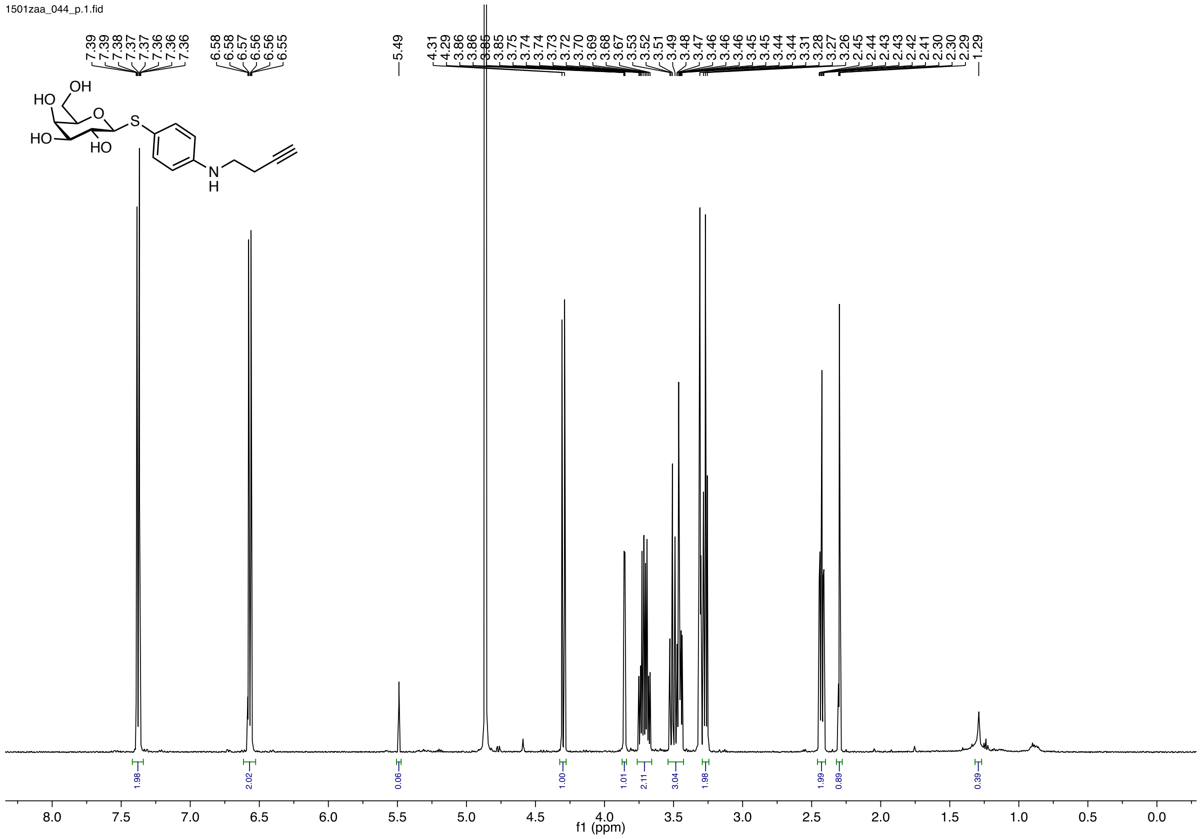

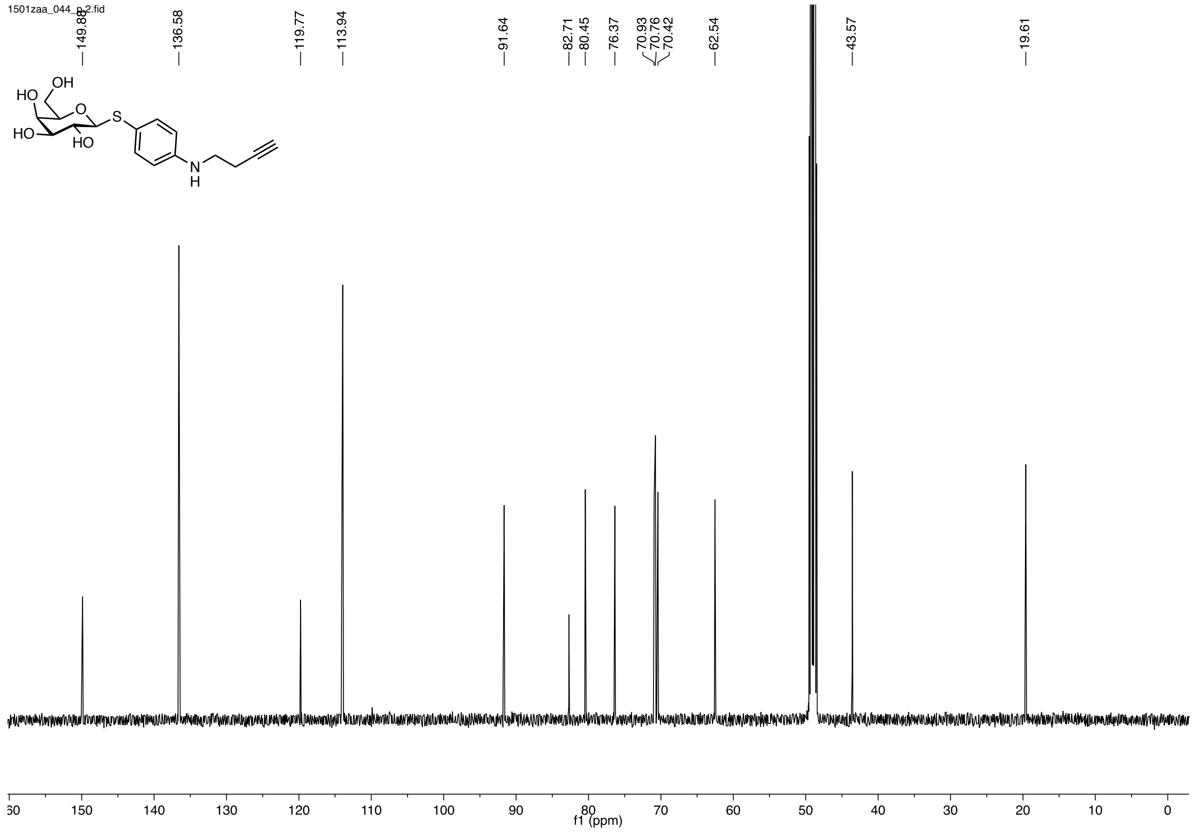

^{1}H and ^{13}C NMR of **S6*p***

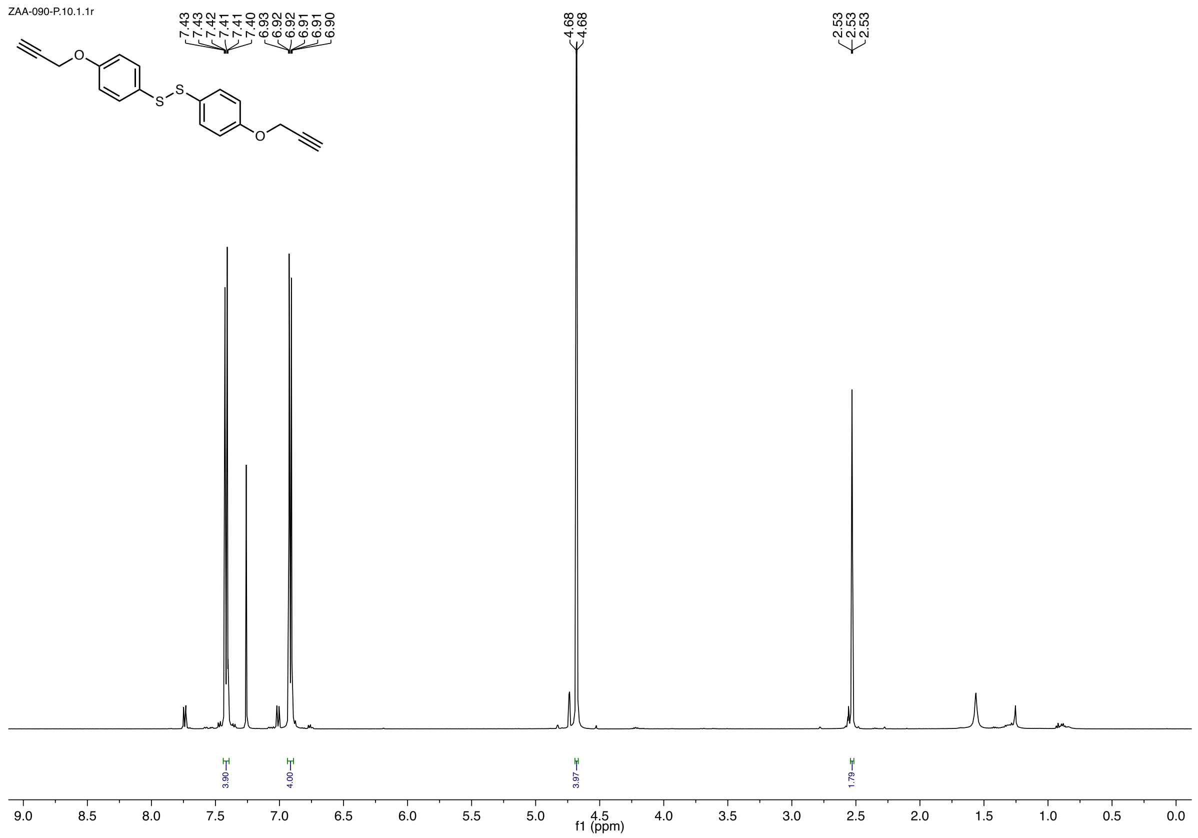

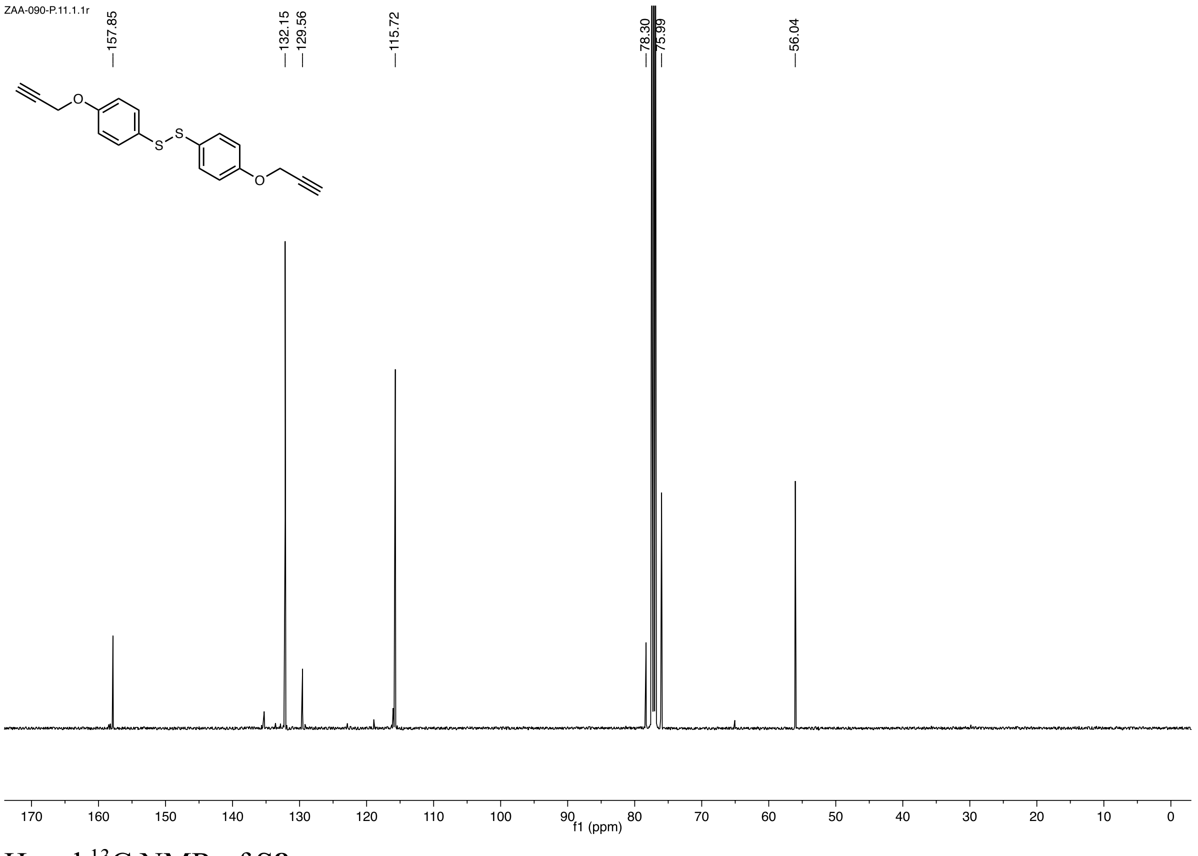

^{1}H and ^{13}C NMR of **S8**

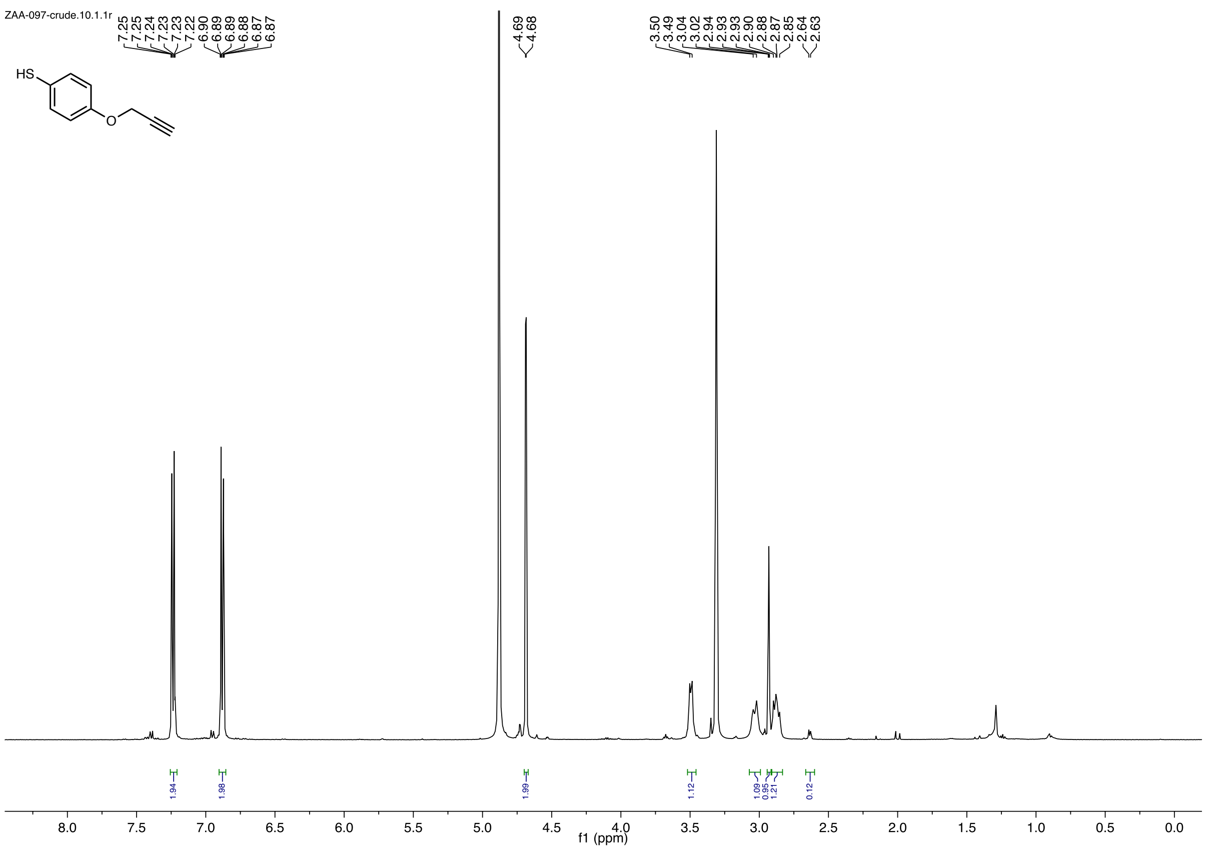

^{1}H of **S9** (crude, impure)

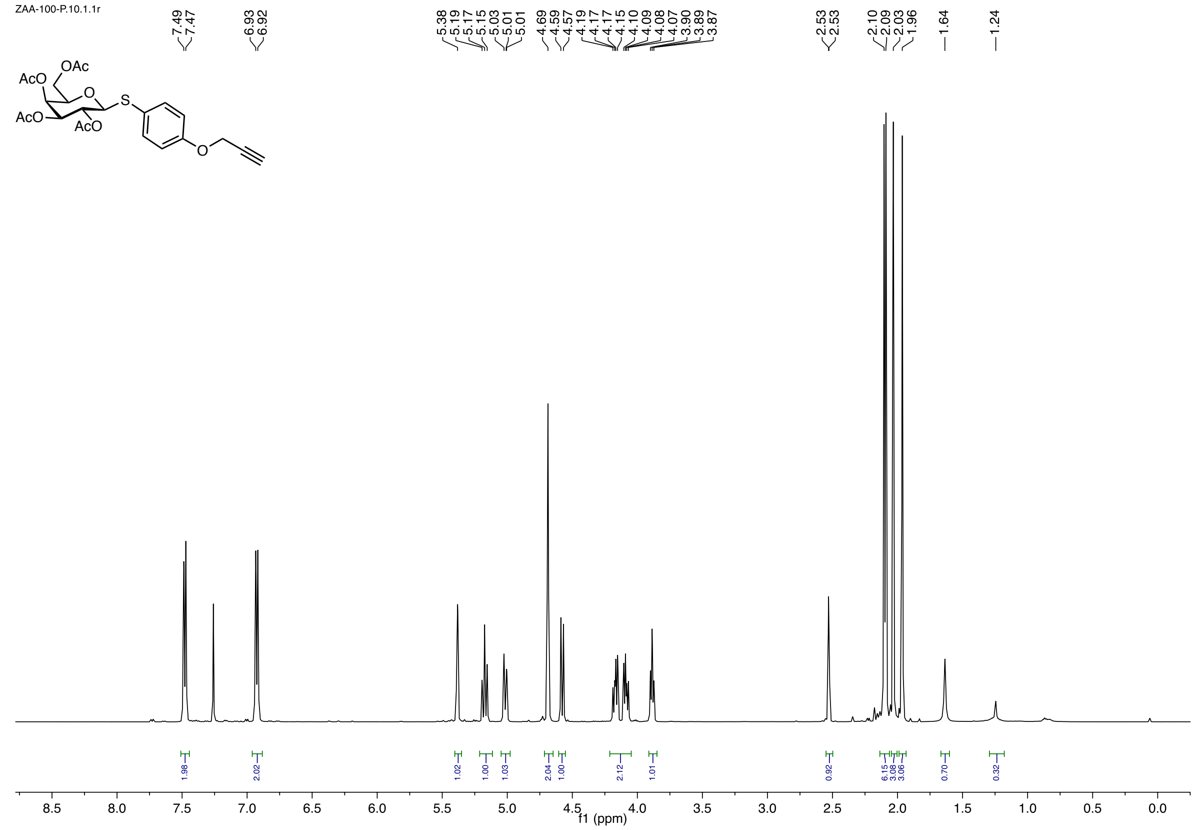

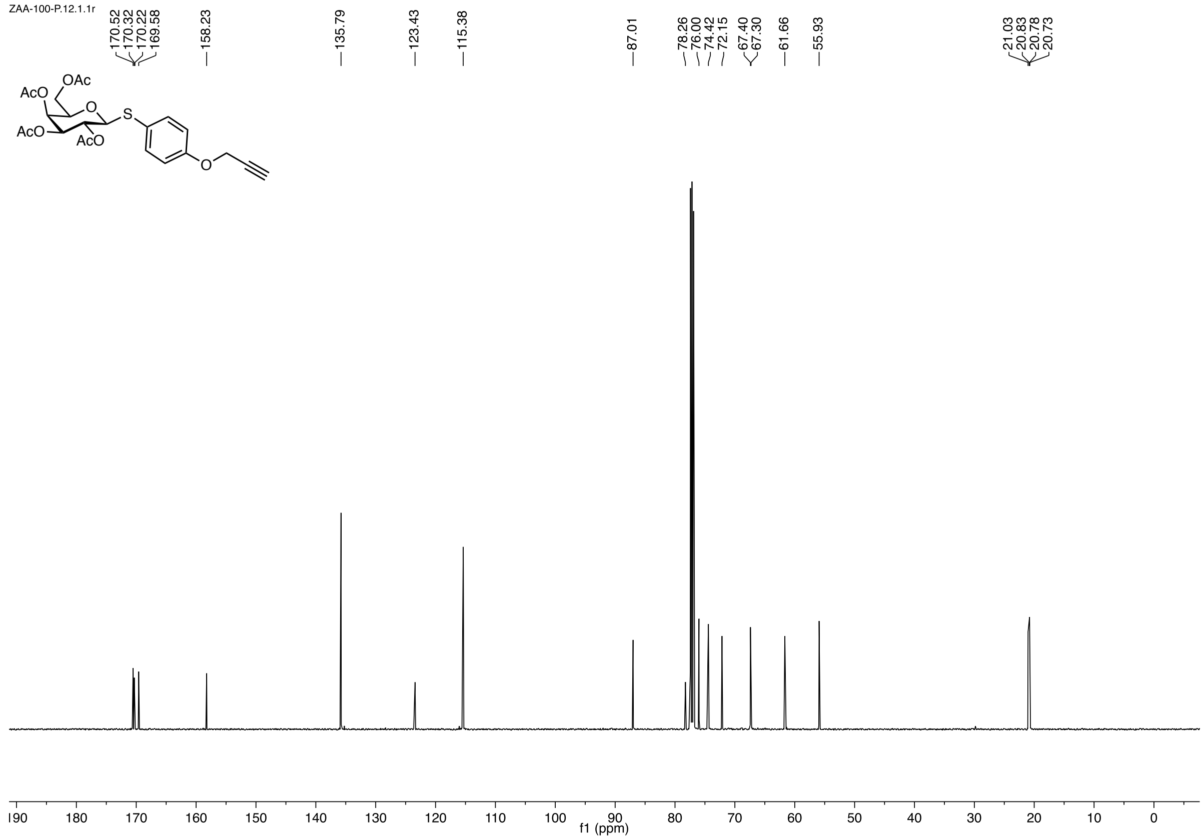

^{1}H and ^{13}C NMR of **S10**

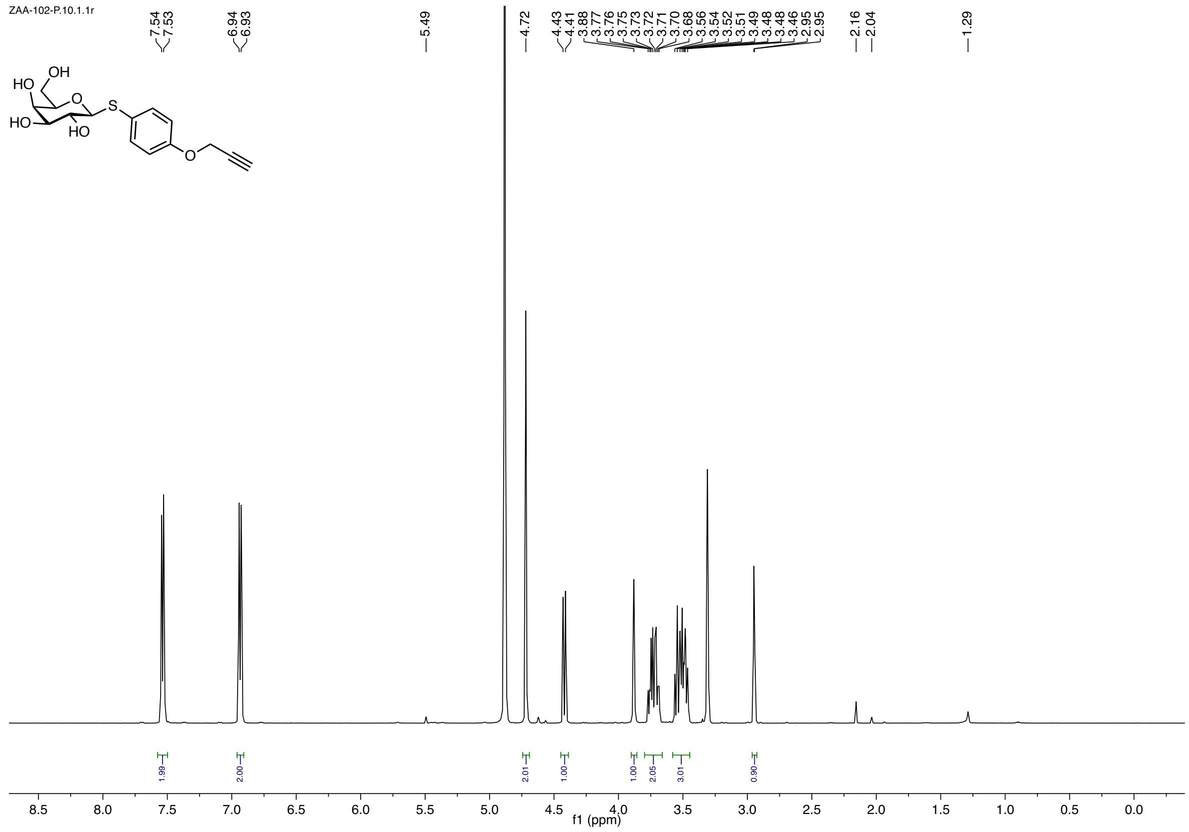

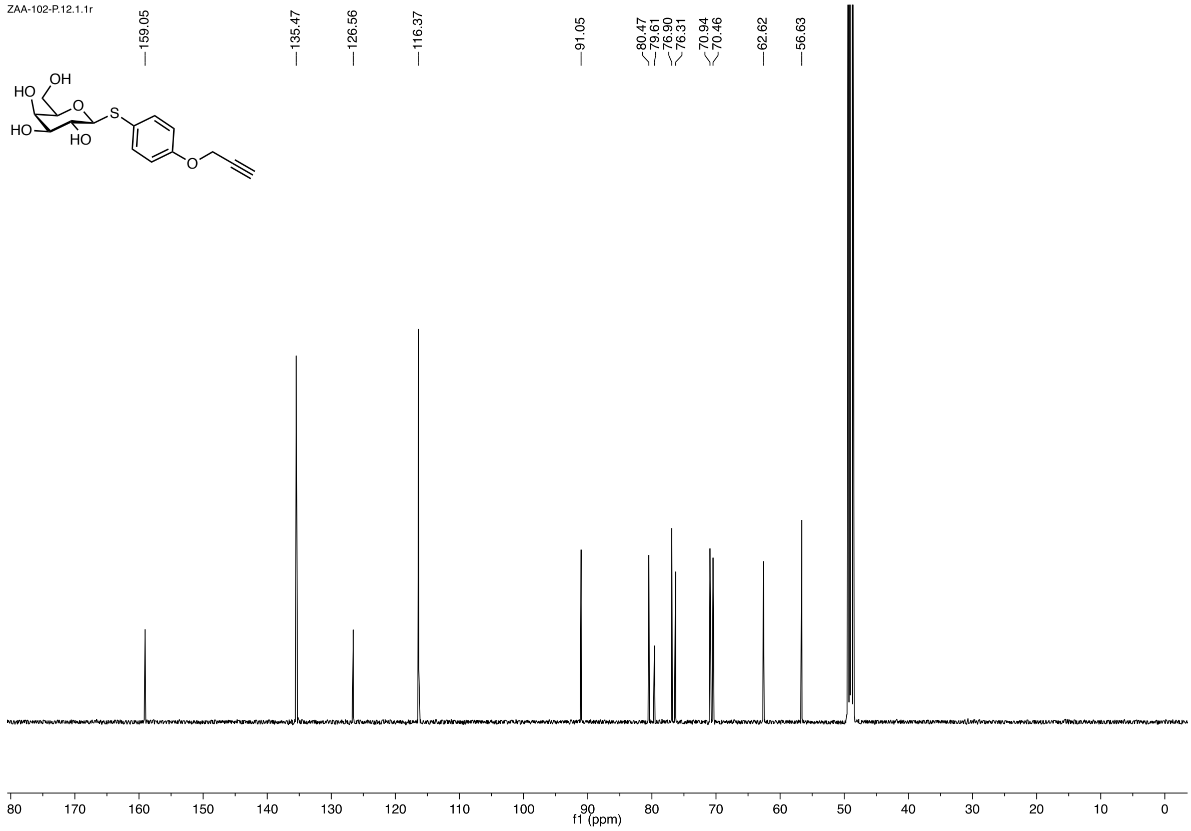

^{1}H and ^{13}C NMR of **S11**

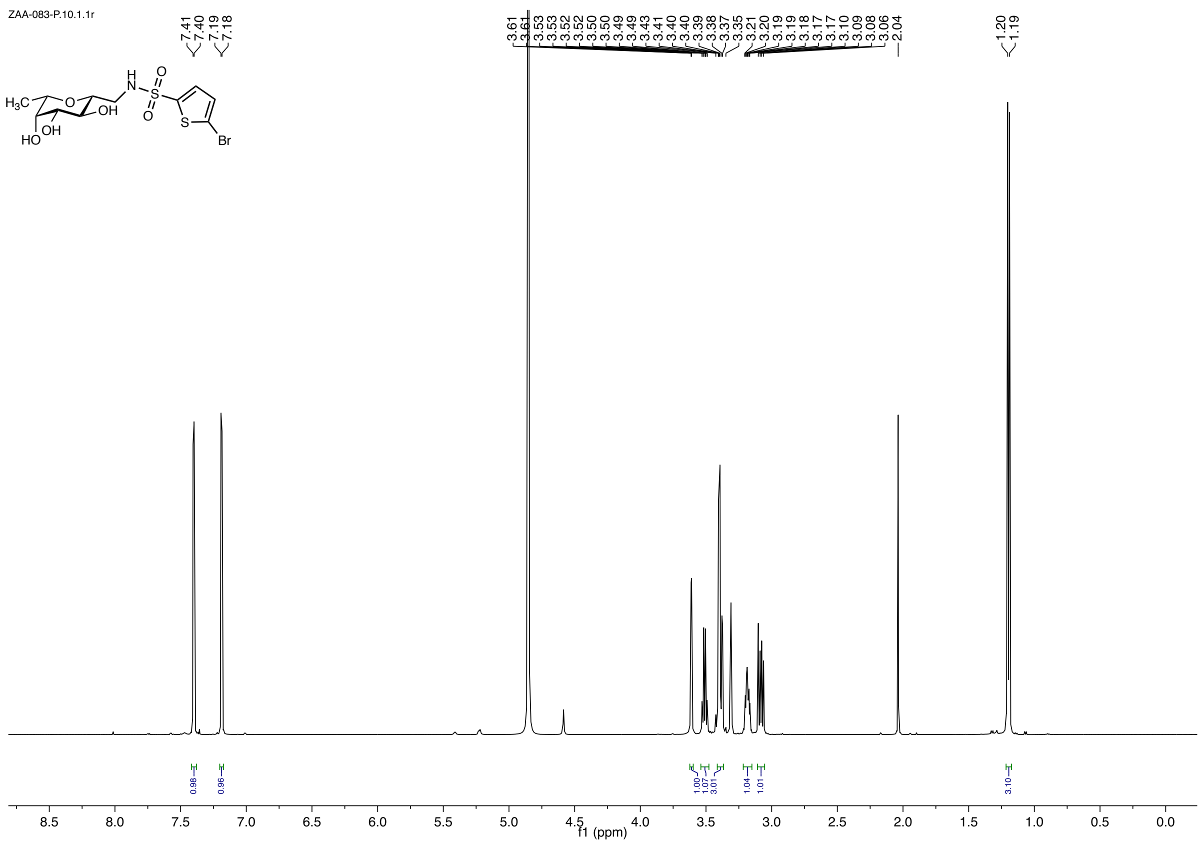

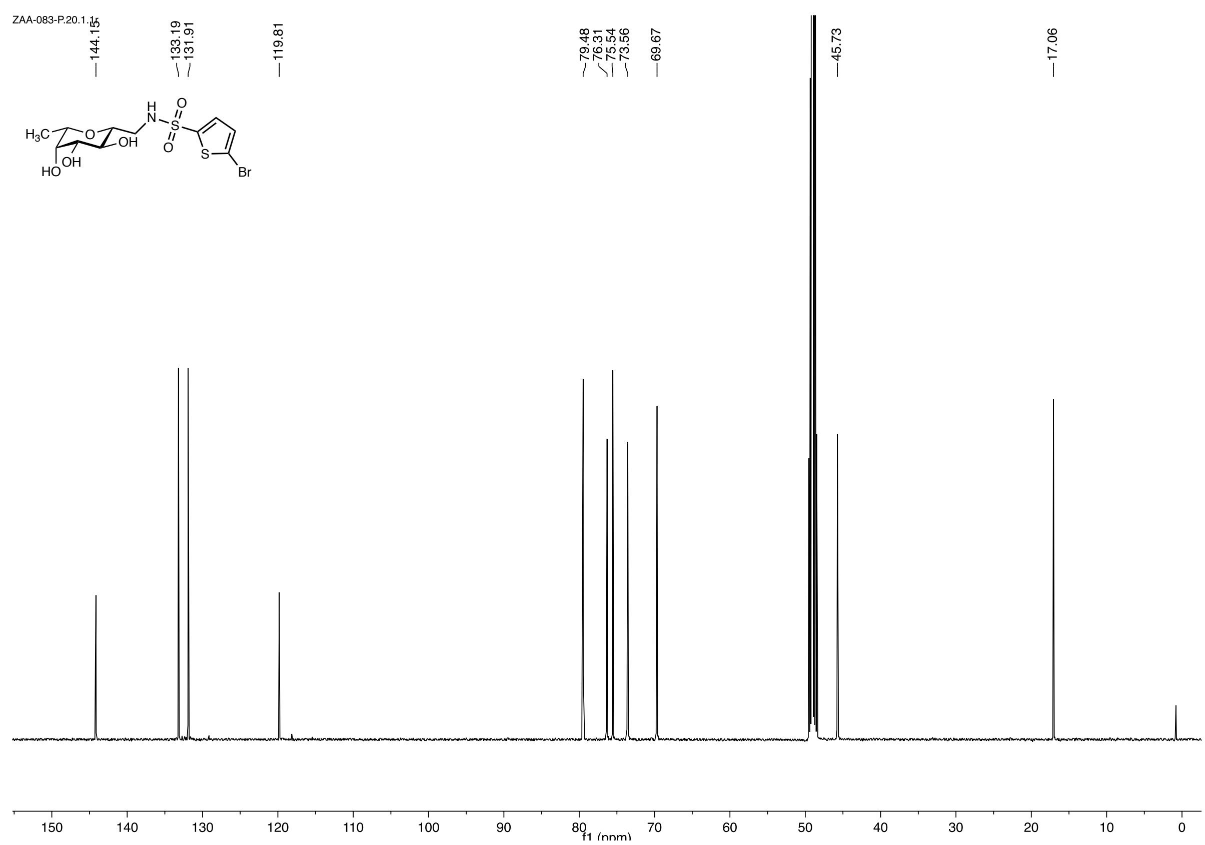

^{1}H and ^{13}C NMR of **S15**

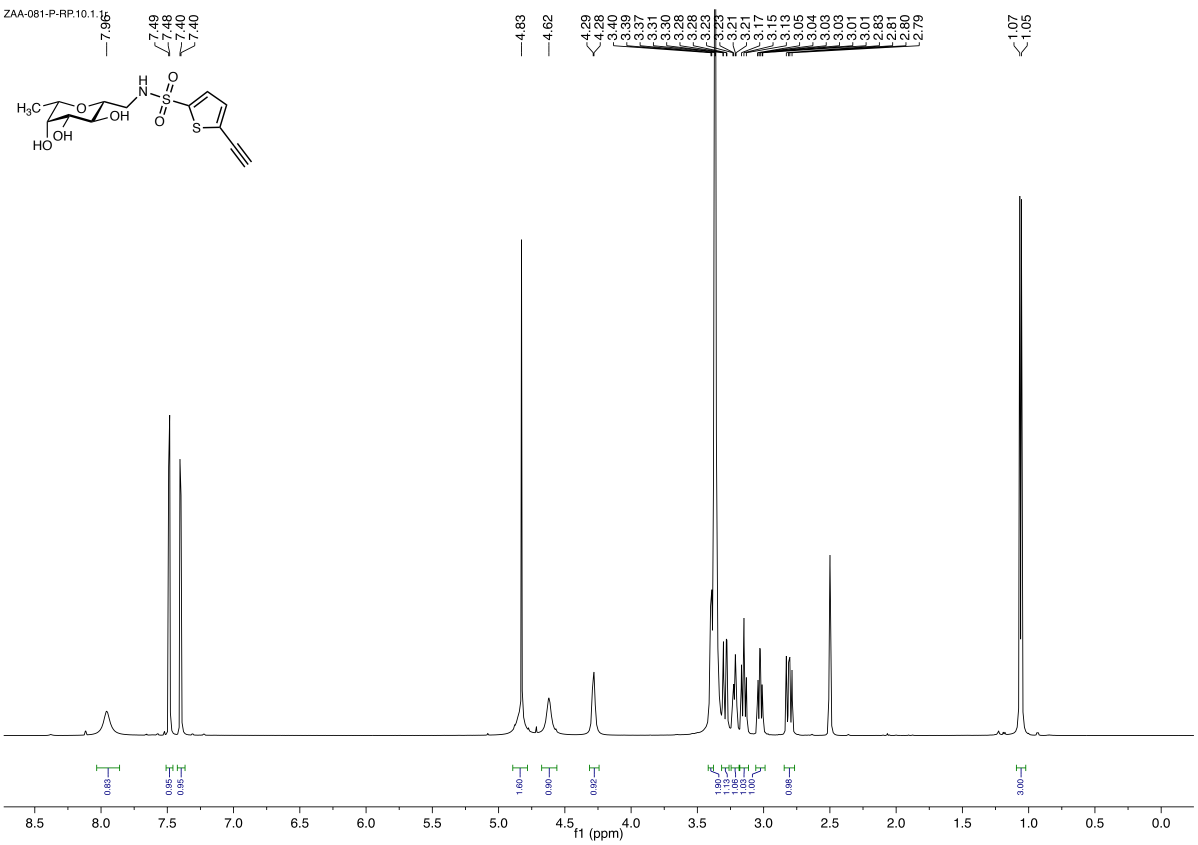

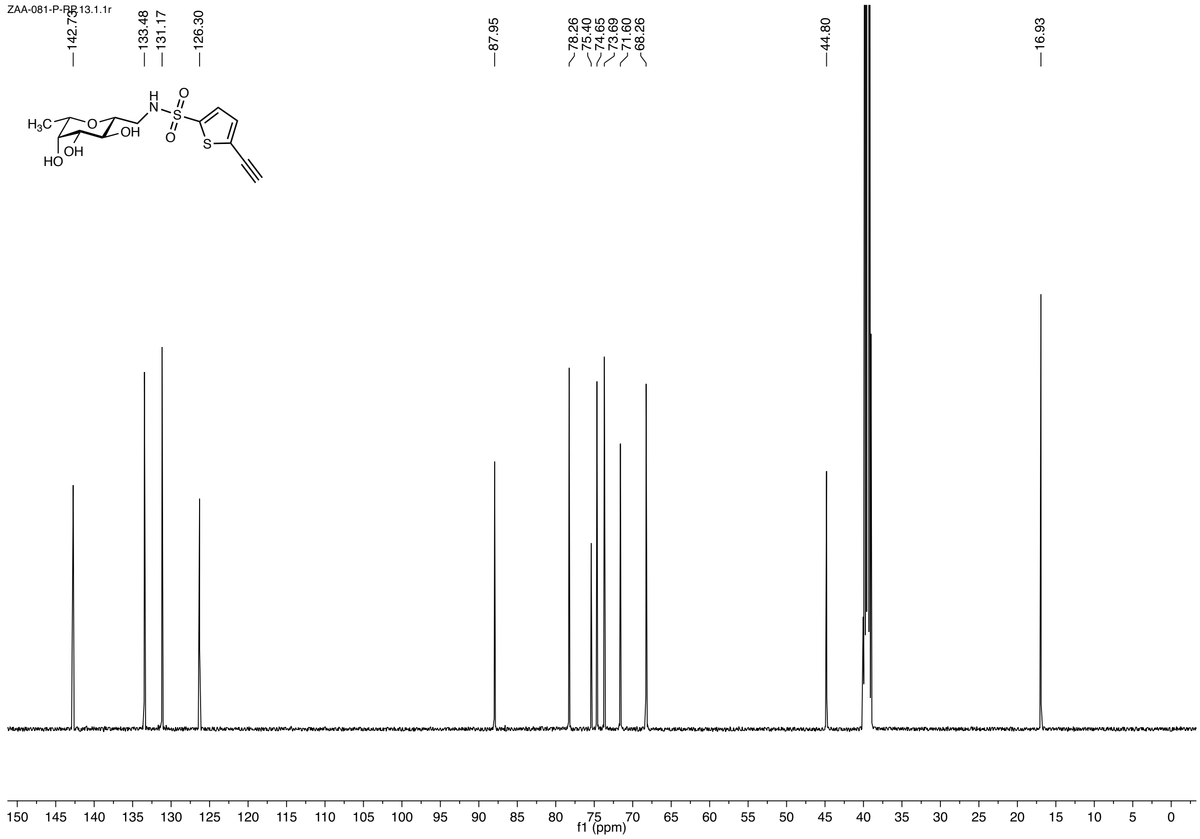

^{1}H and ^{13}C NMR of **23**

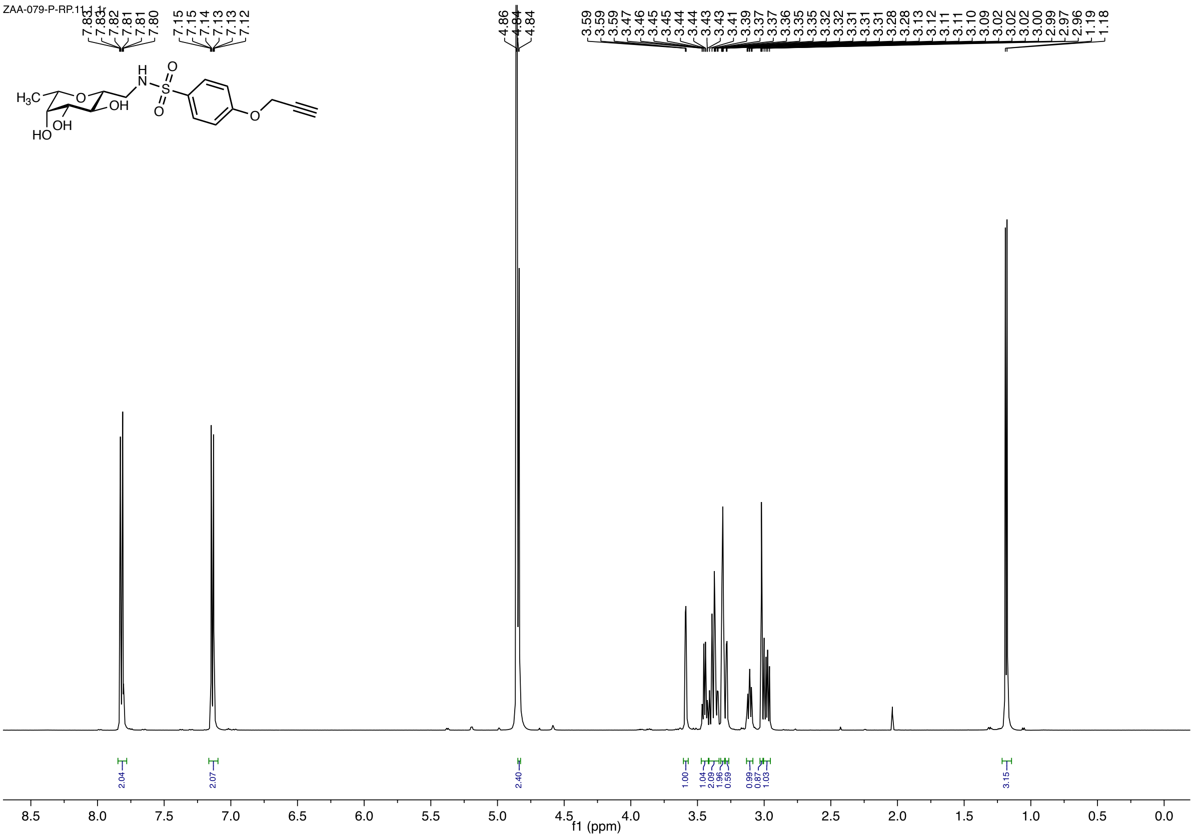

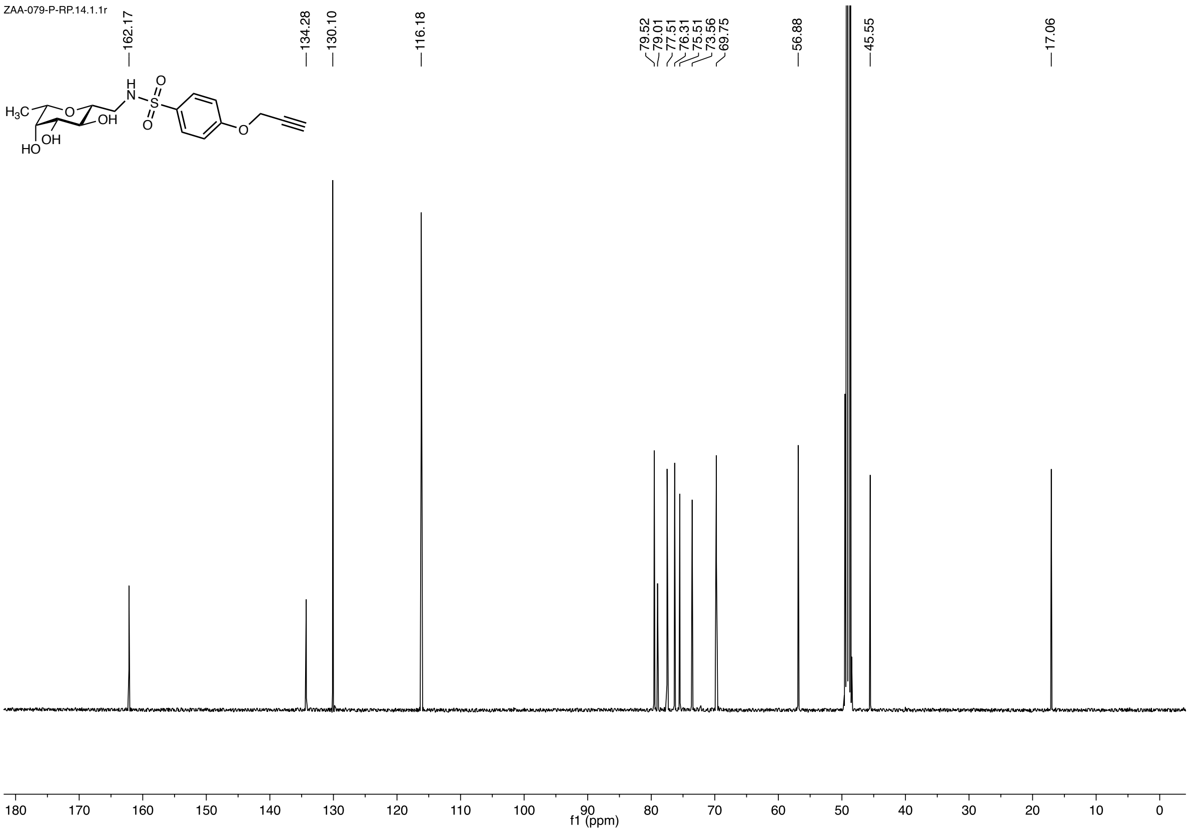

^{1}H and ^{13}C NMR of **S16**

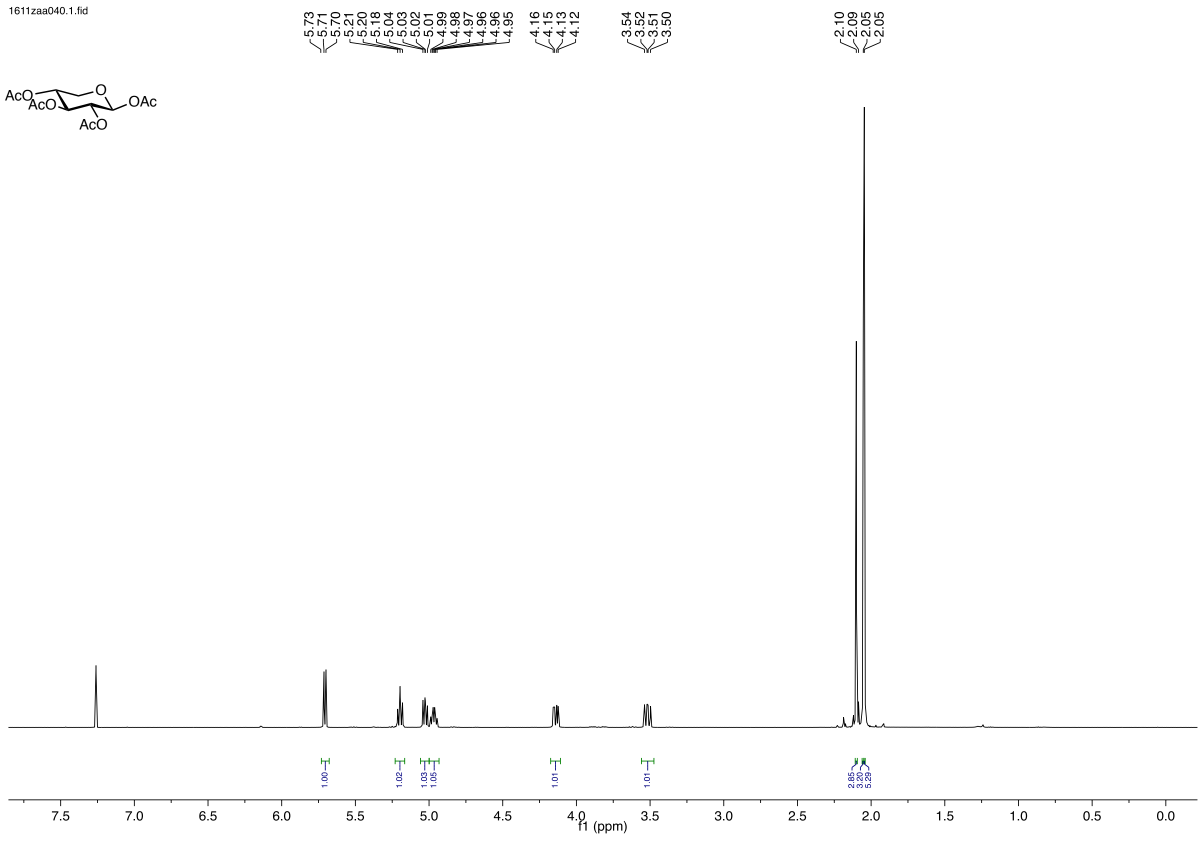

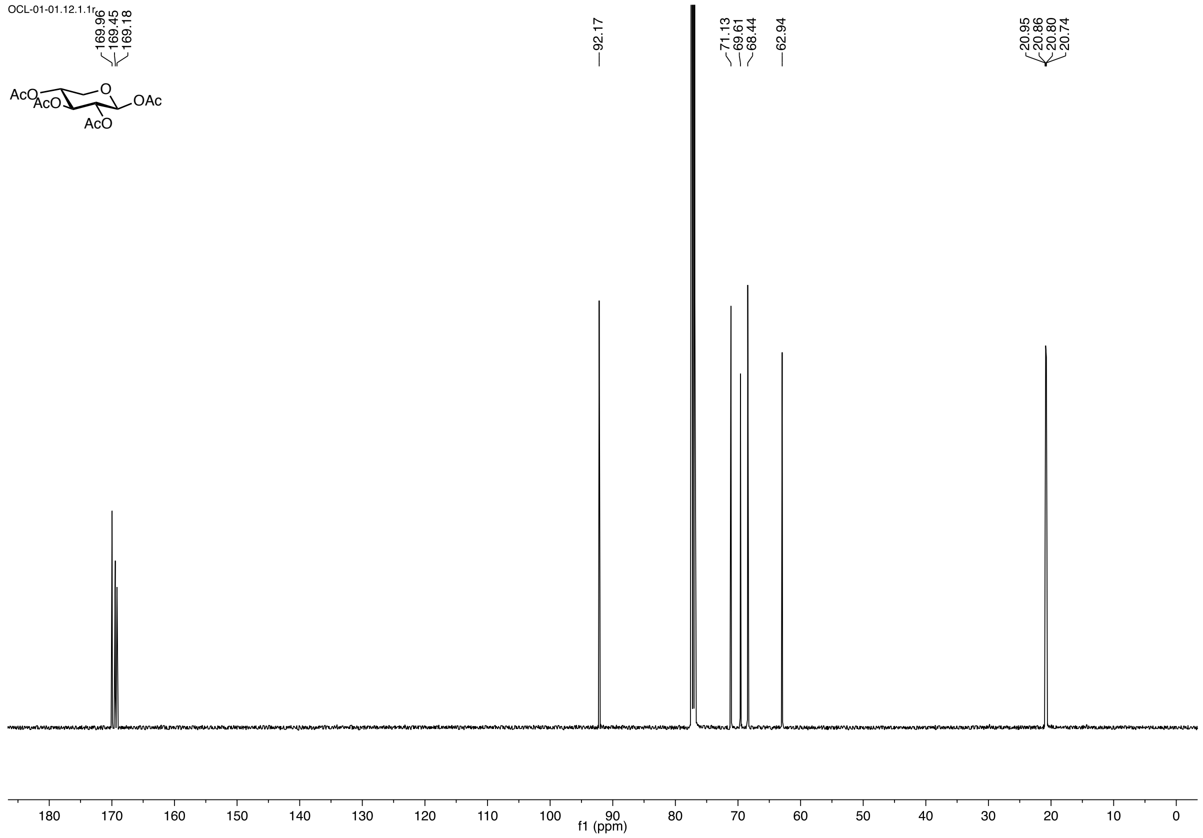

^{1}H and ^{13}C NMR of **S18**

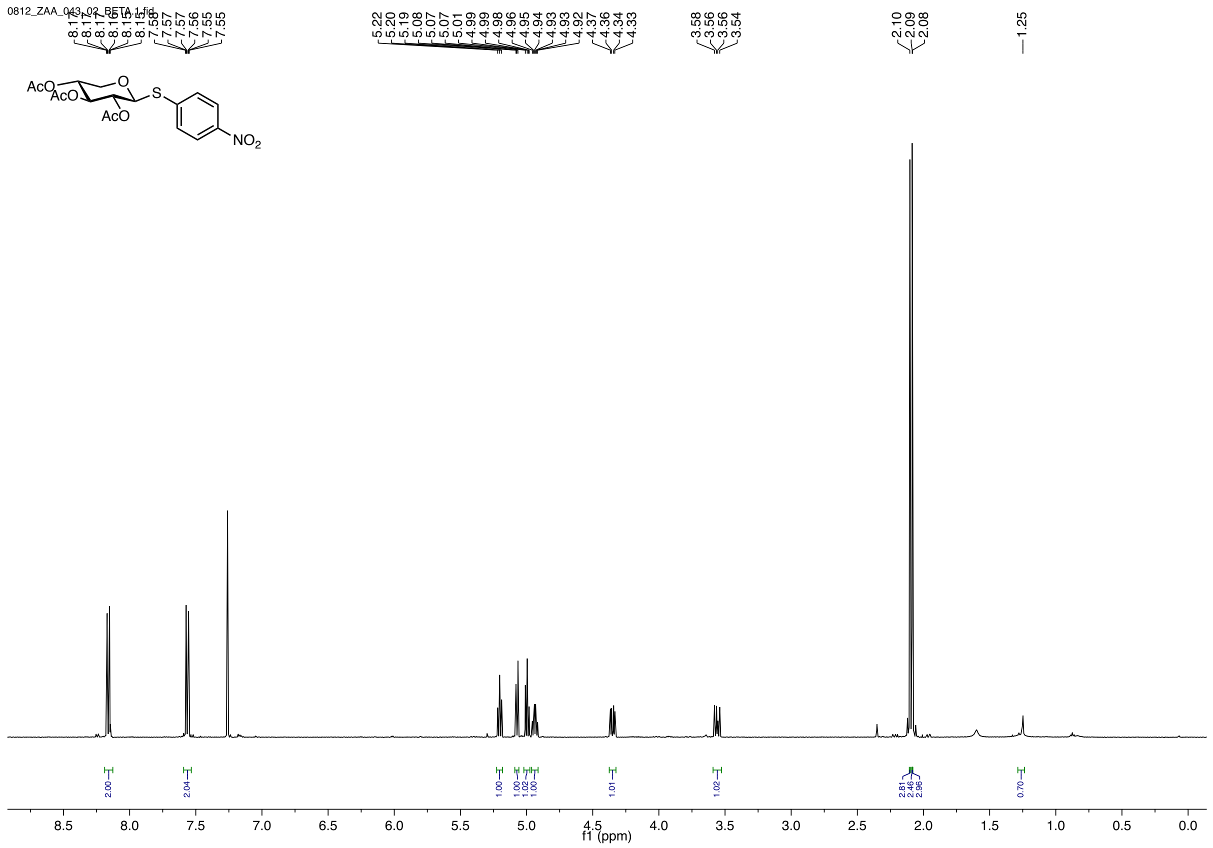

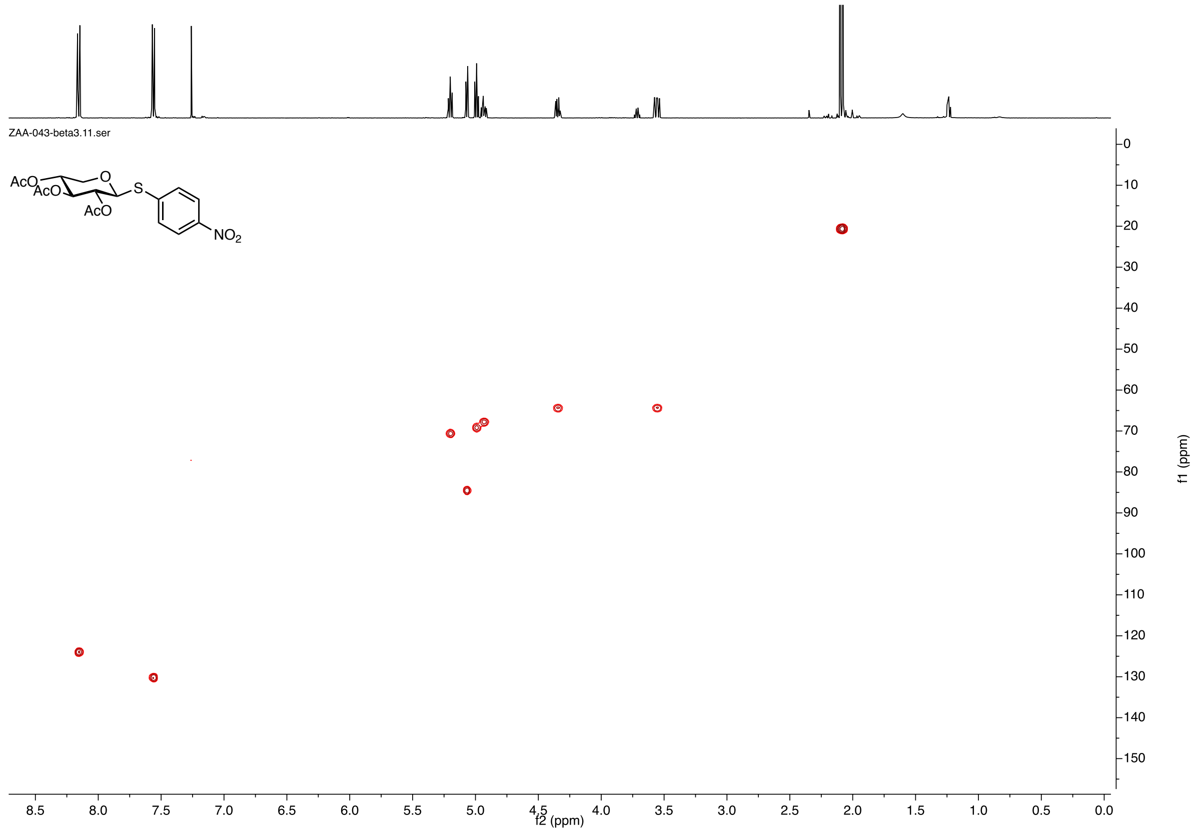

^{1}H and HSQC NMR of **S19**

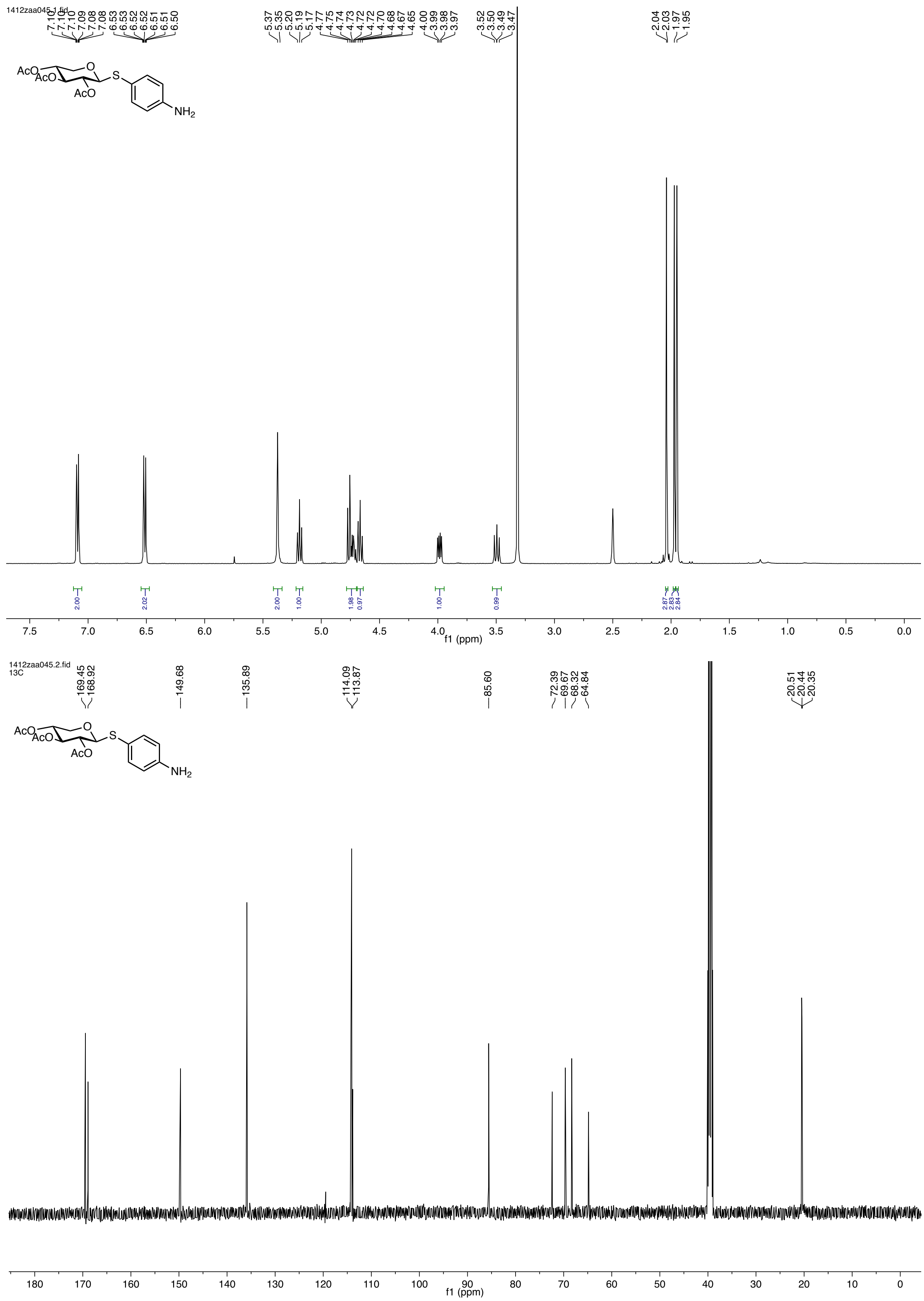

^{1}H and ^{13}C NMR of **S20**

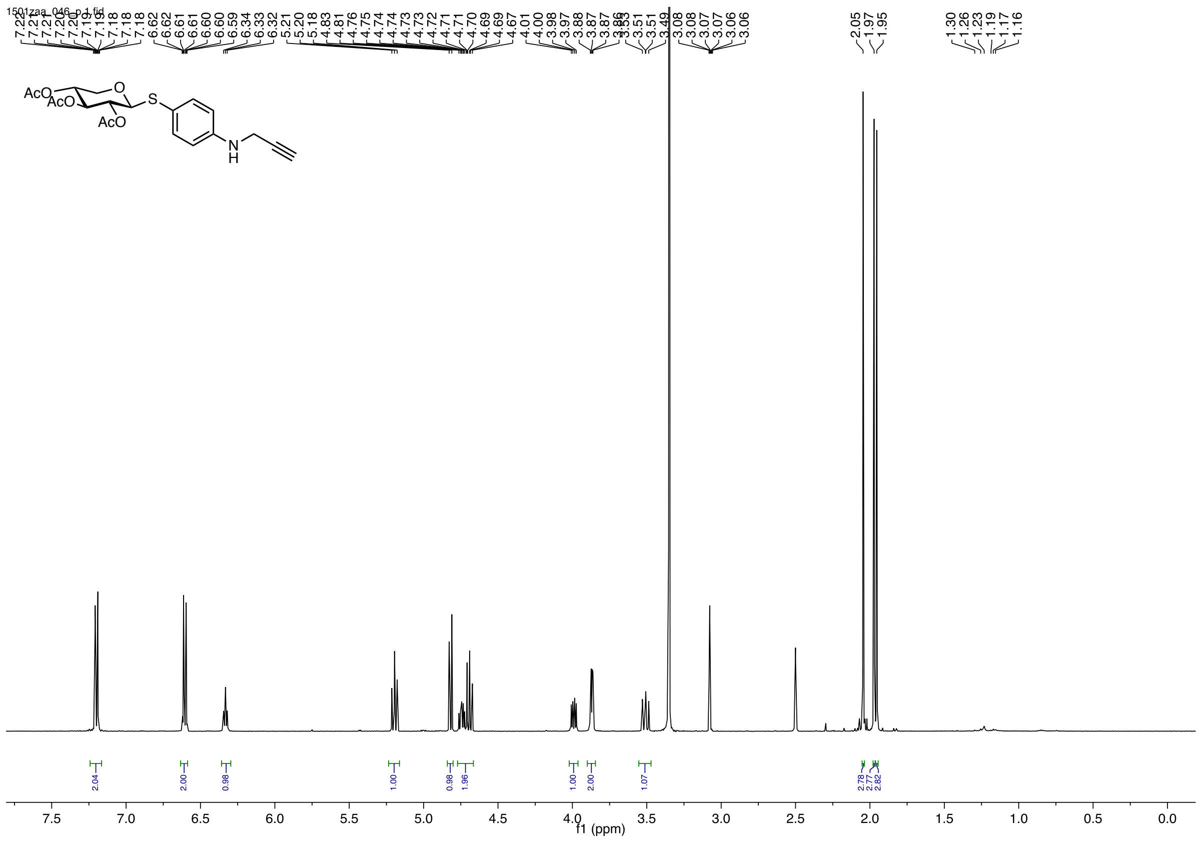

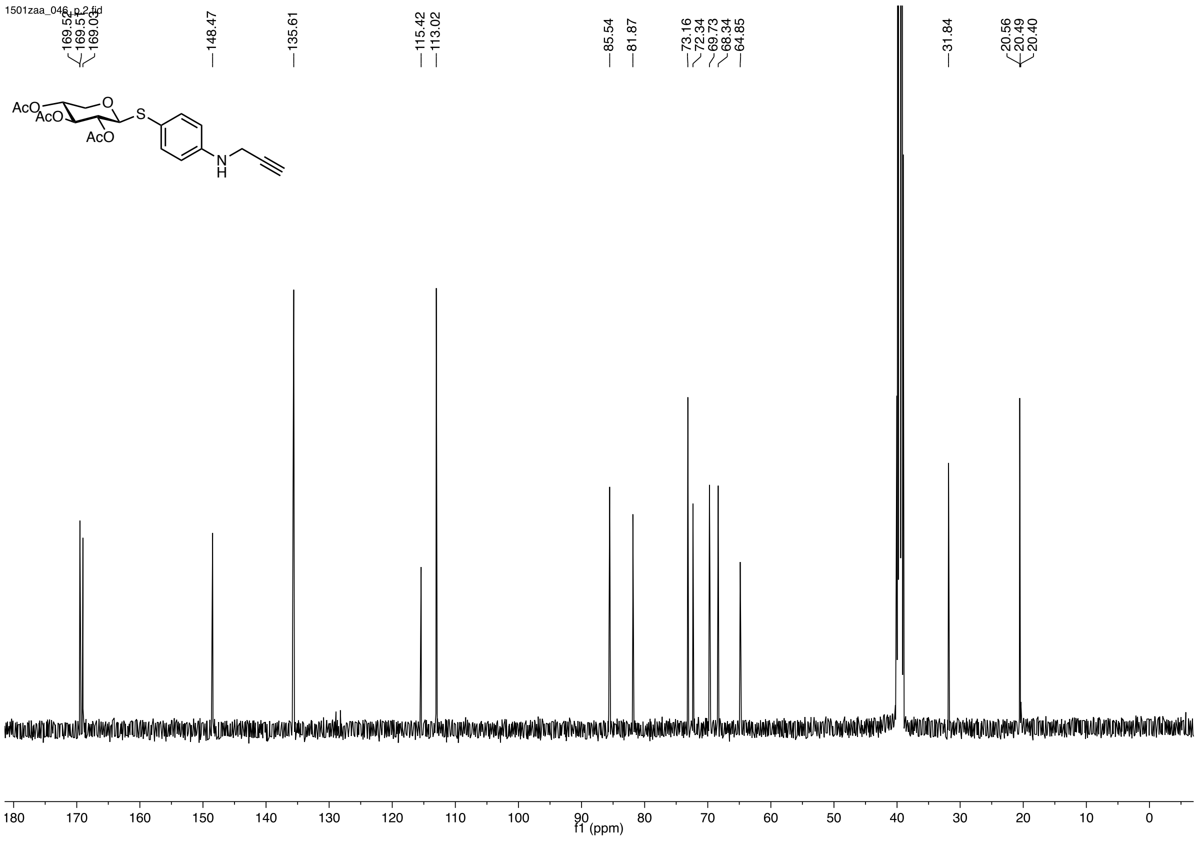

^{1}H and ^{13}C NMR of **S21**

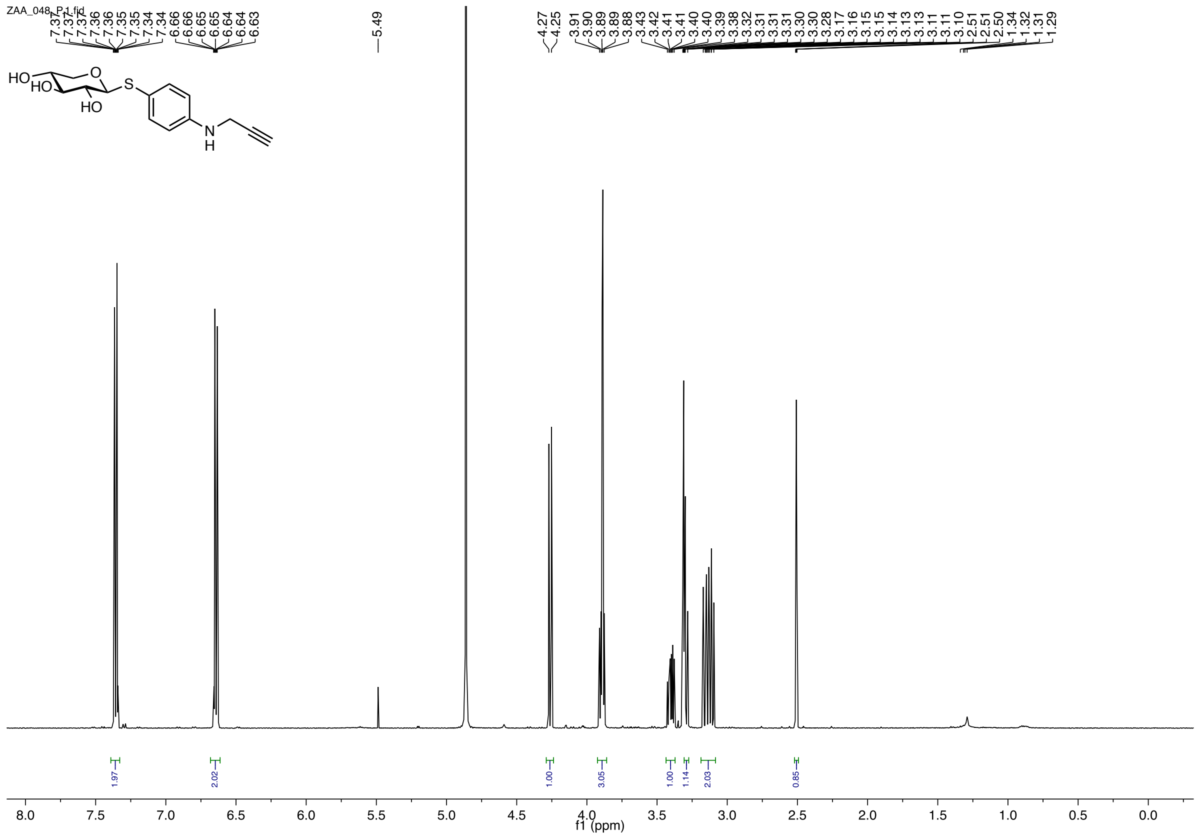

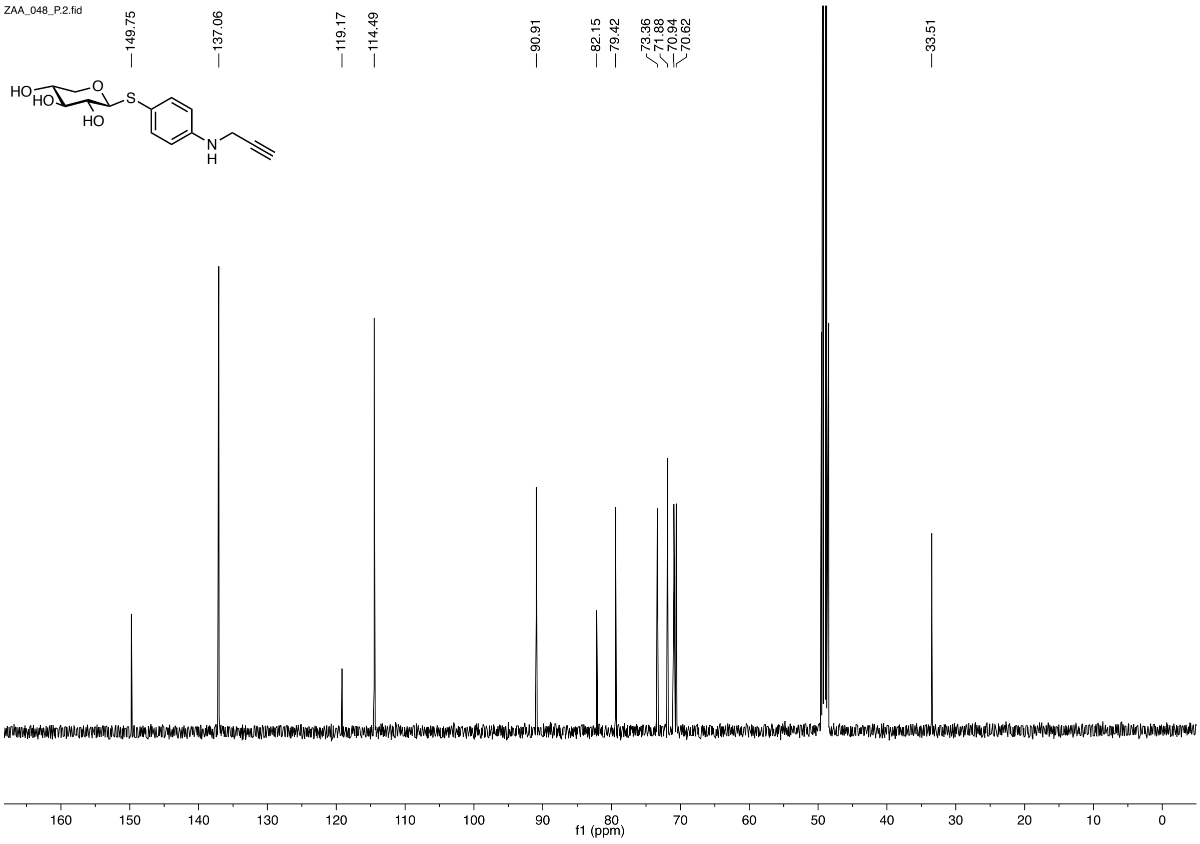

^{1}H and ^{13}C NMR of **S22**

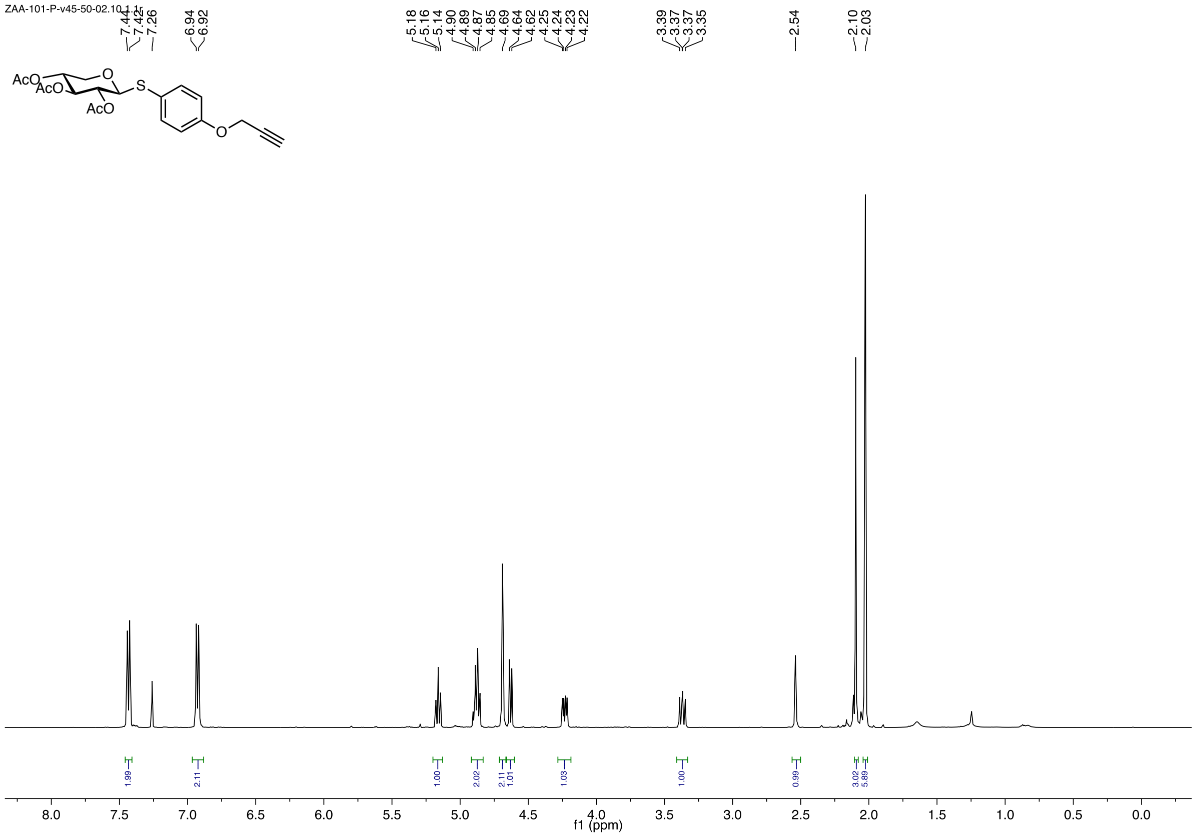

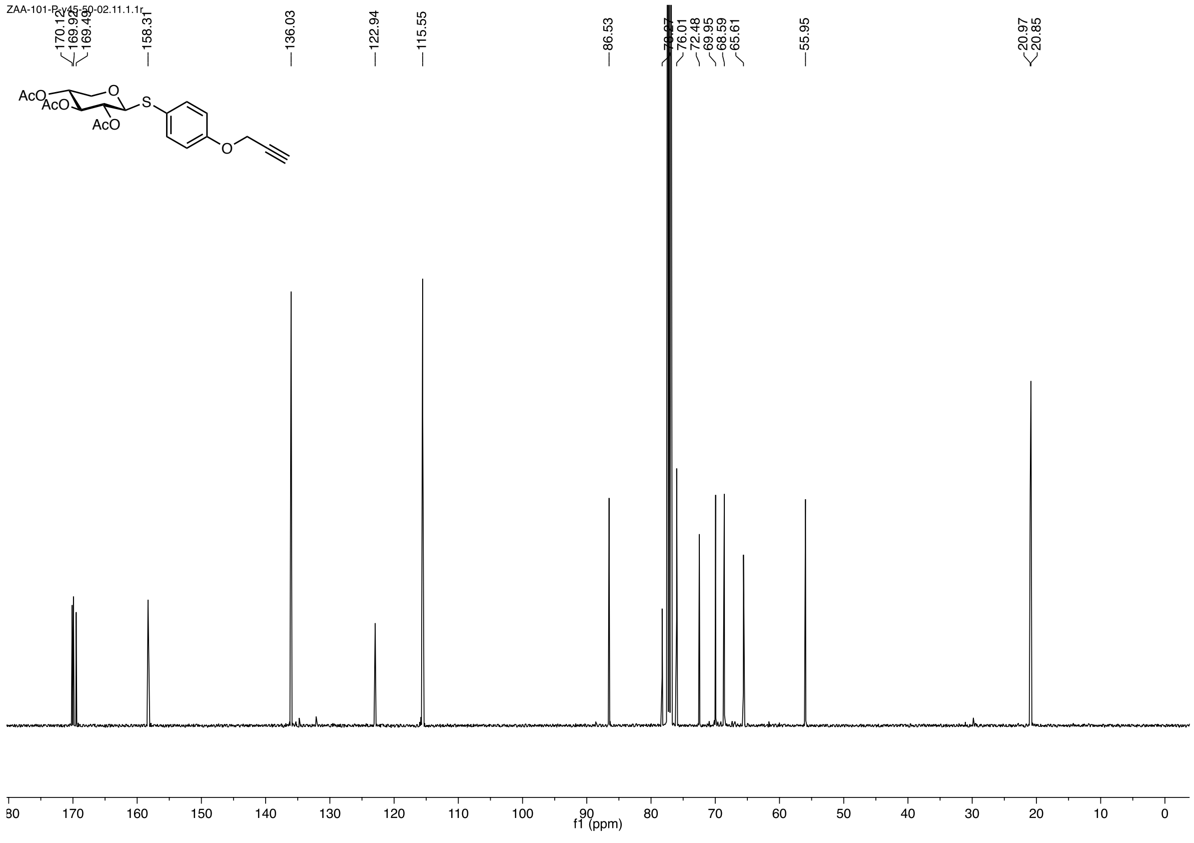

^{1}H and ^{13}C NMR of **S23**

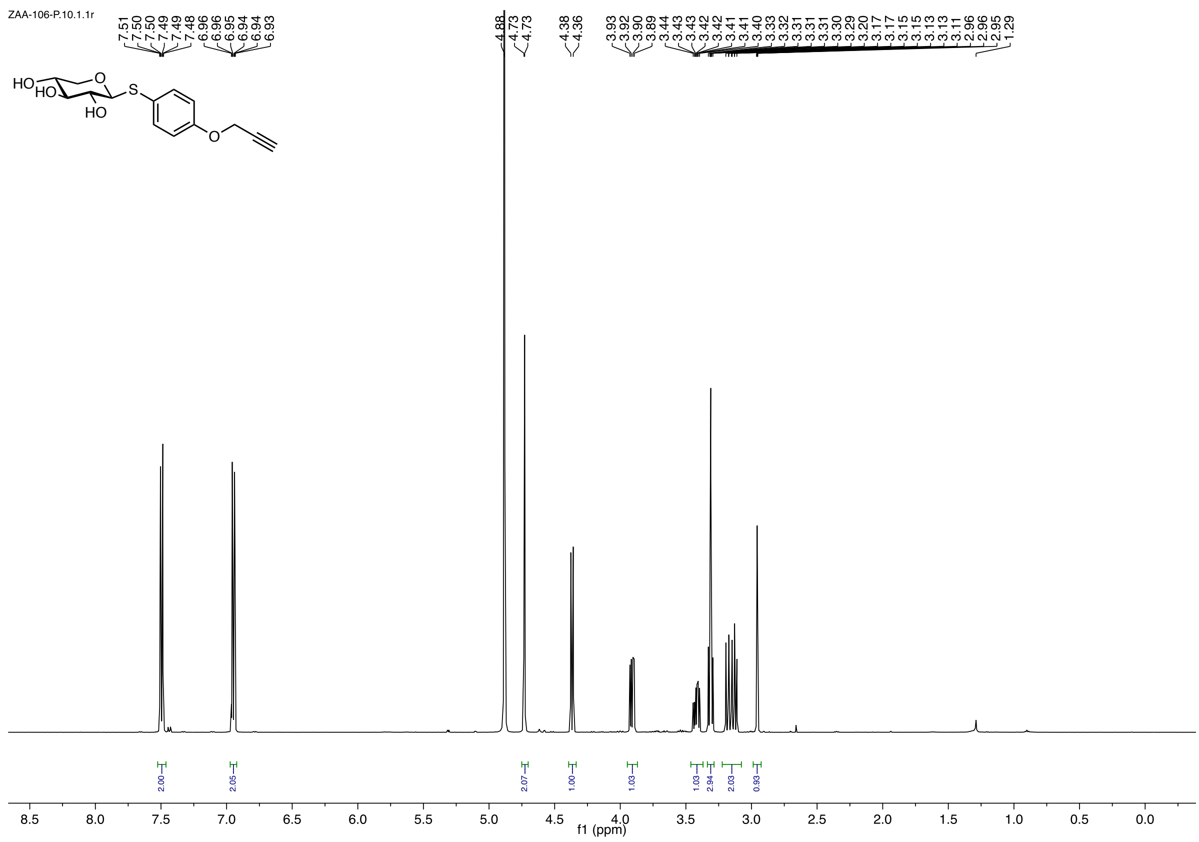

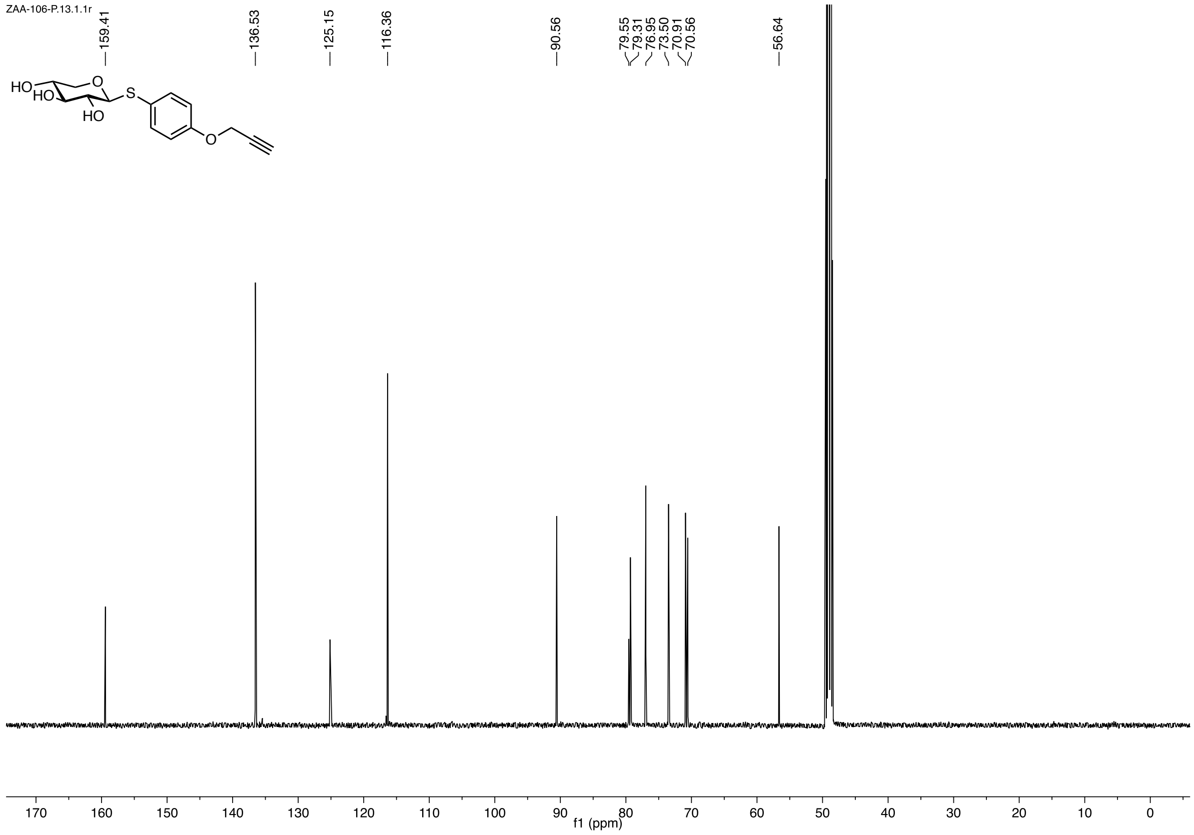

^{1}H and ^{13}C NMR of **S24**

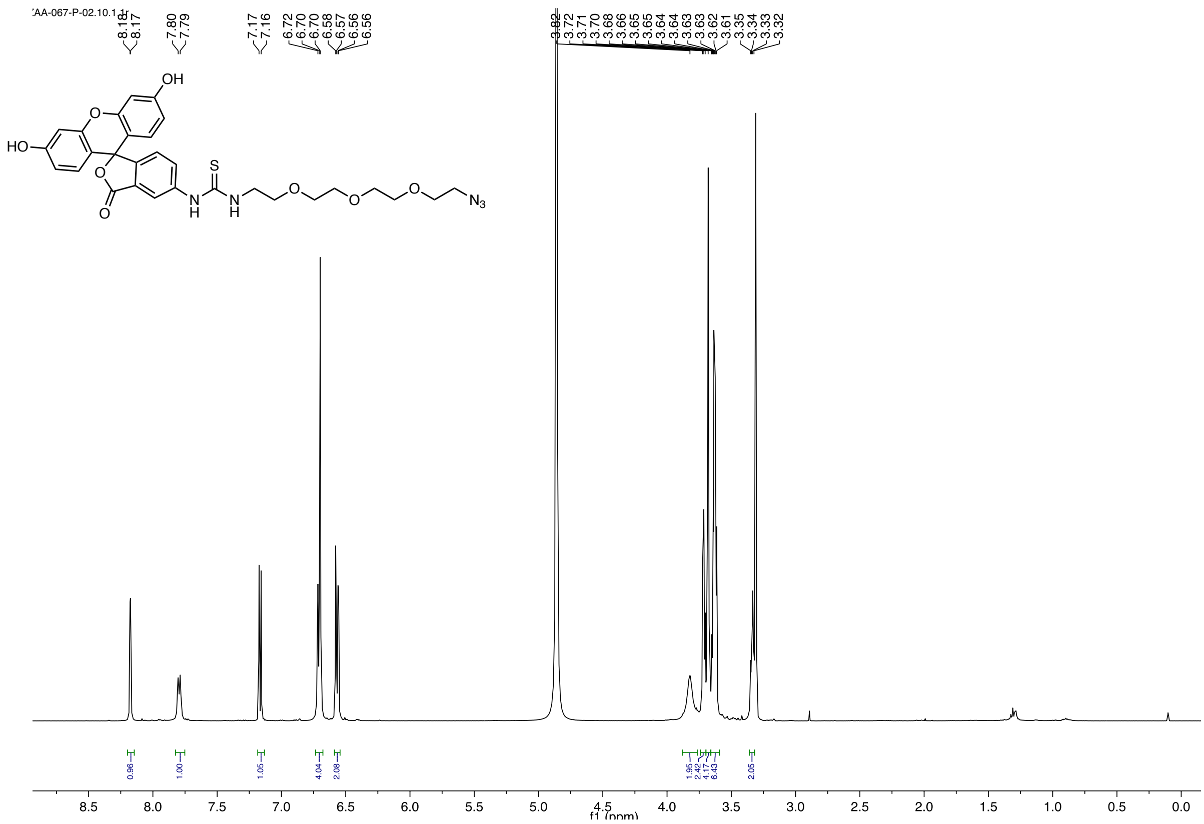

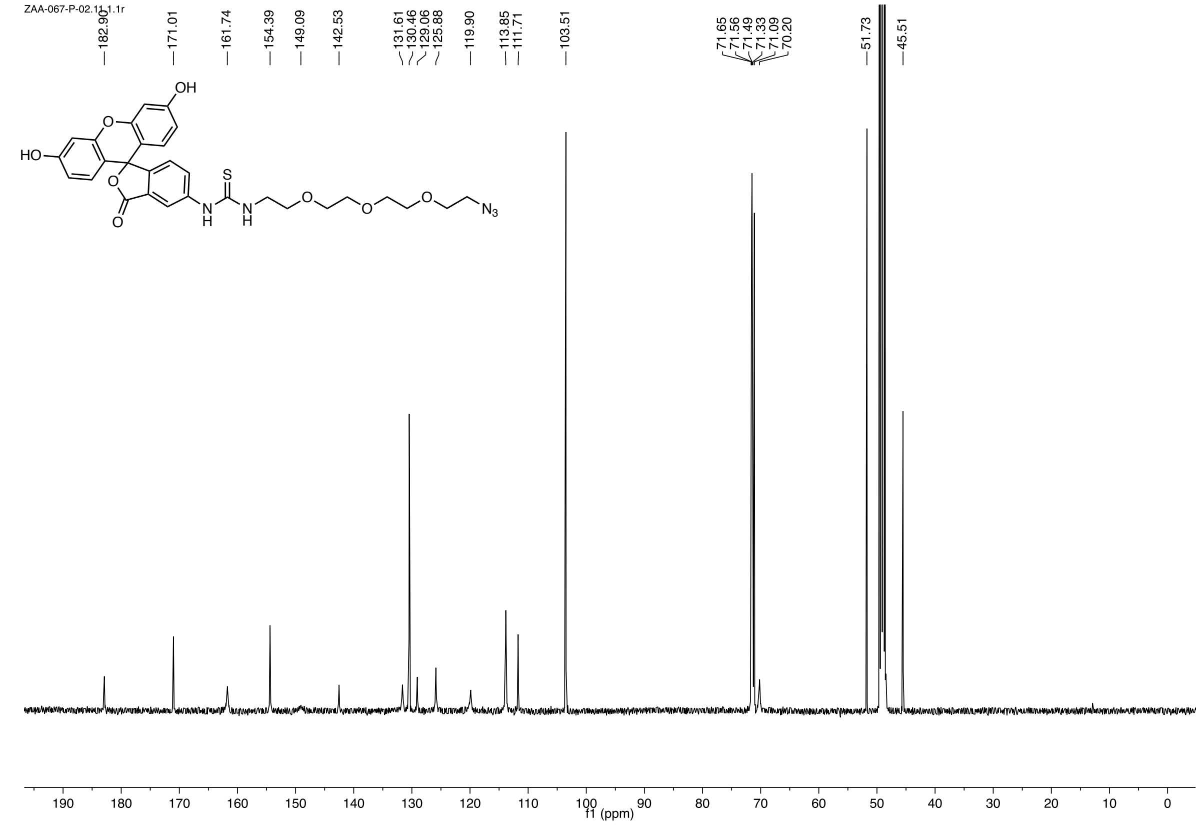

^{1}H and ^{13}C NMR of **9**

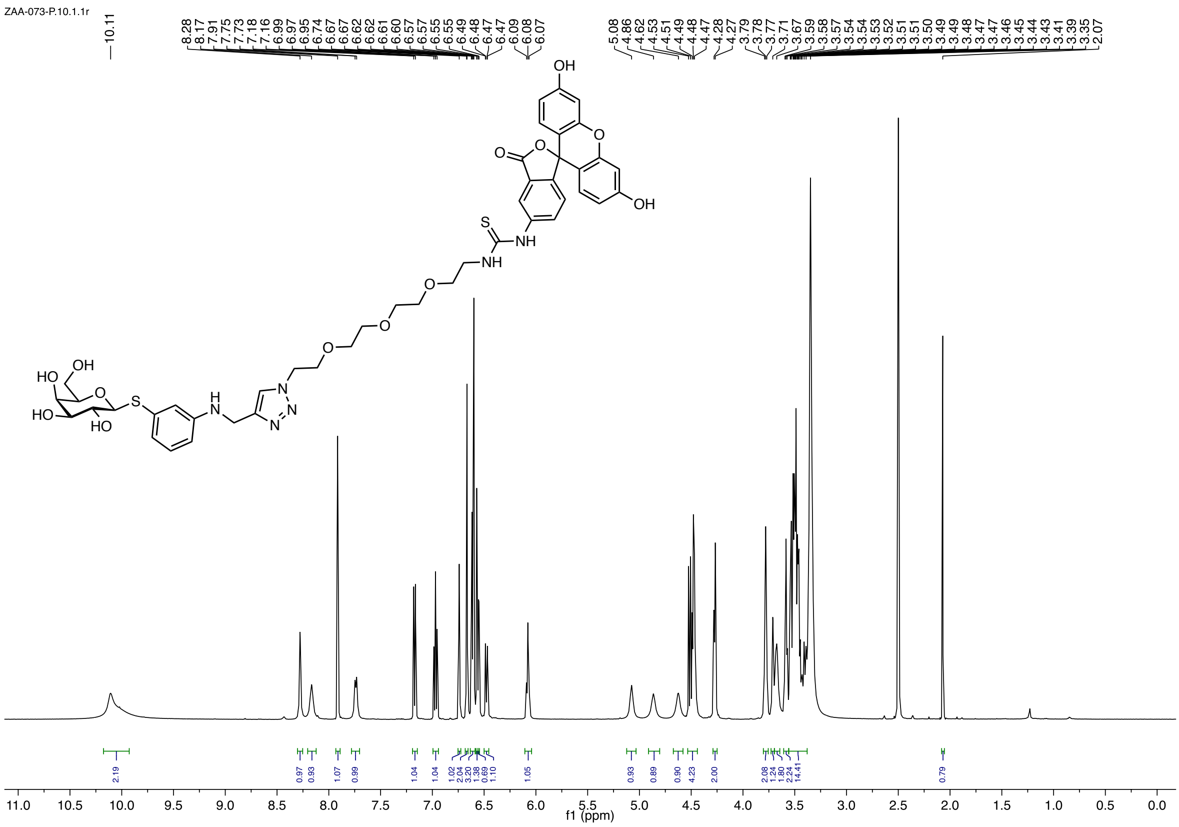

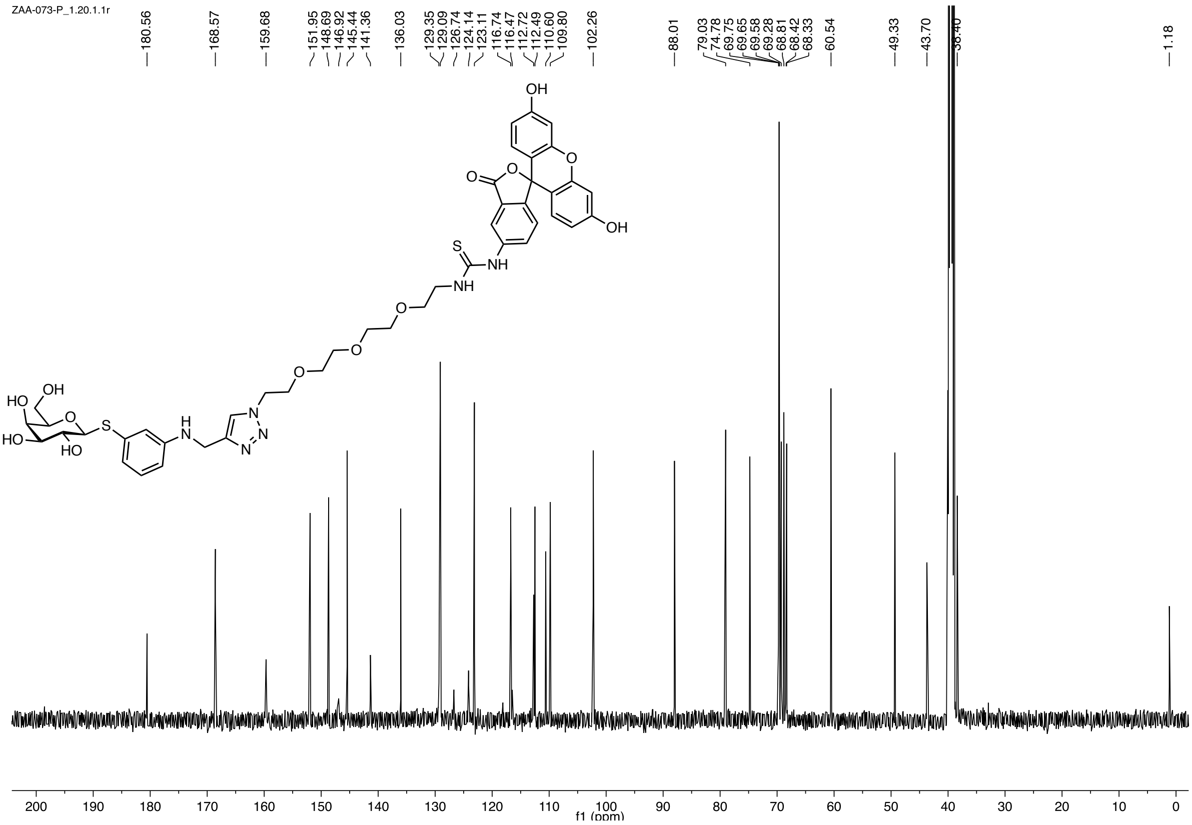

^{1}H and ^{13}C NMR of **2**

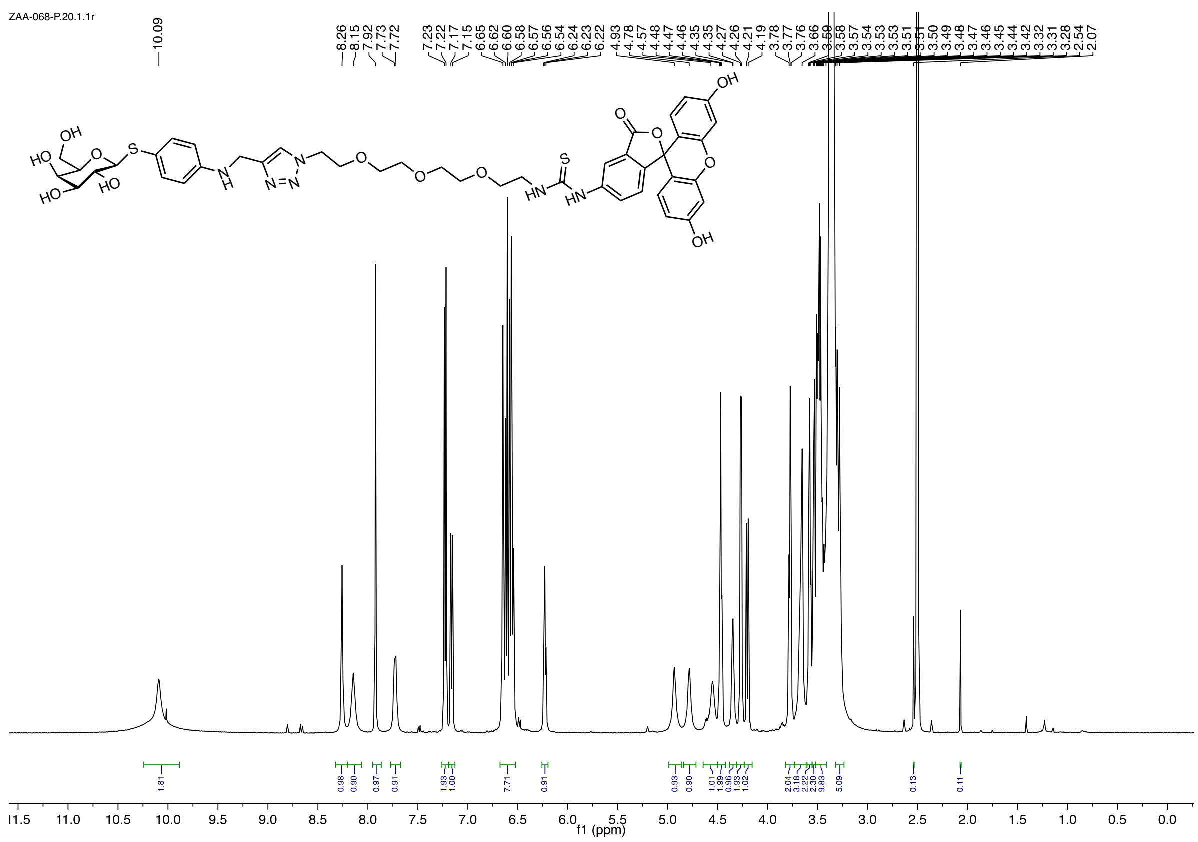

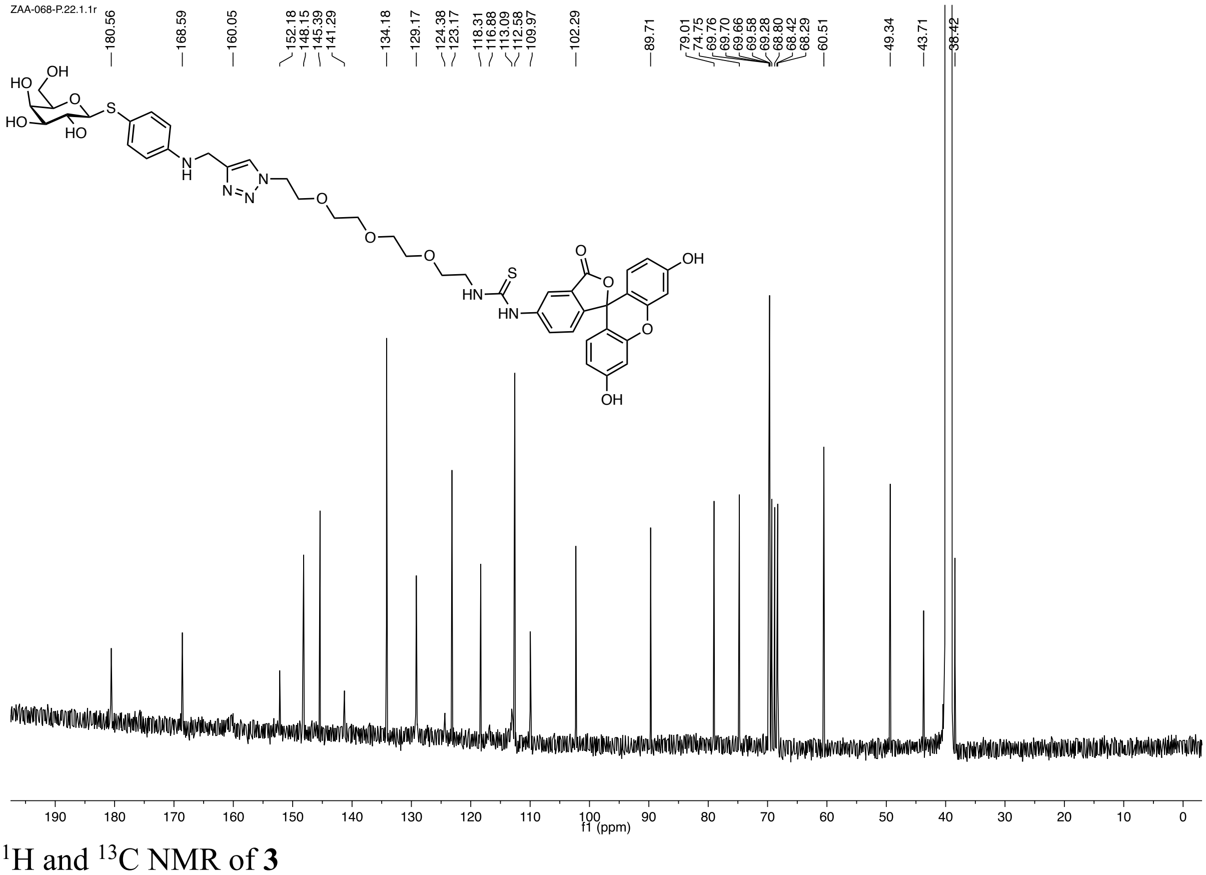

^{1}H and ^{13}C NMR of **3**

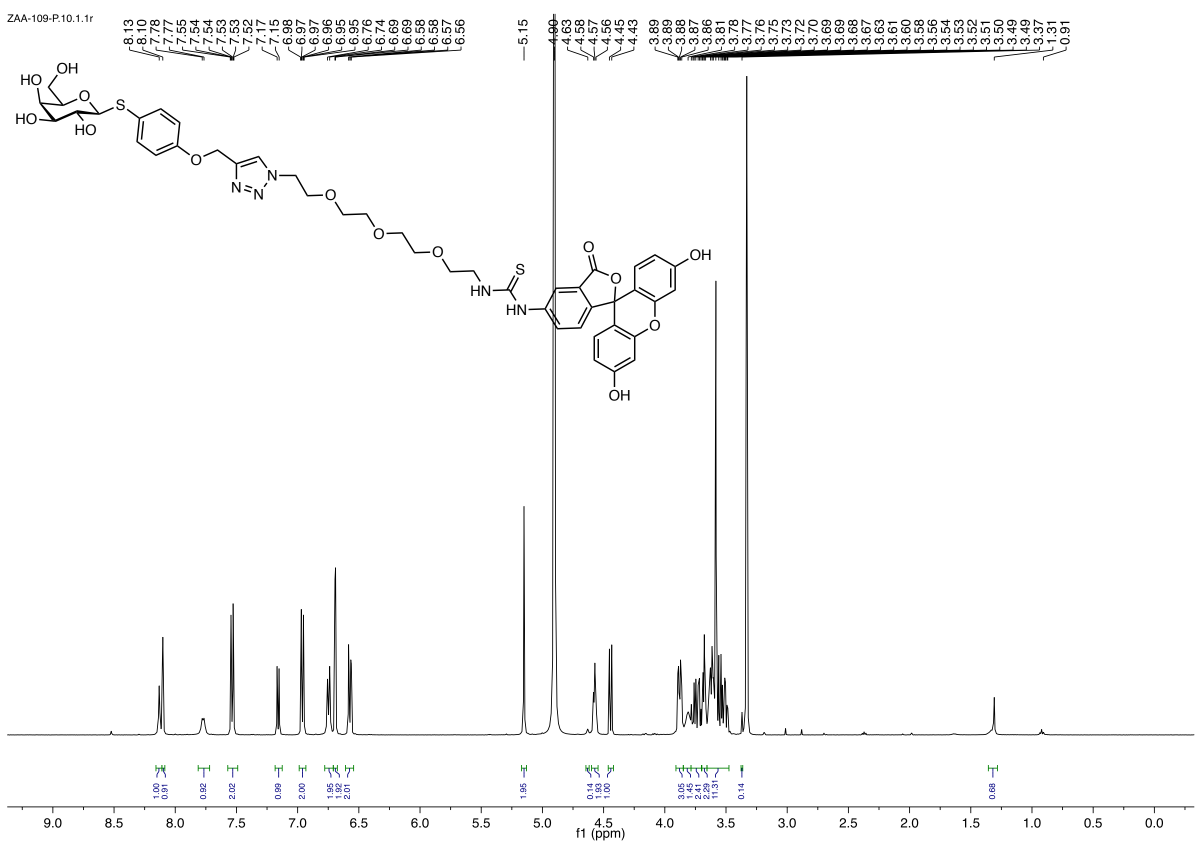

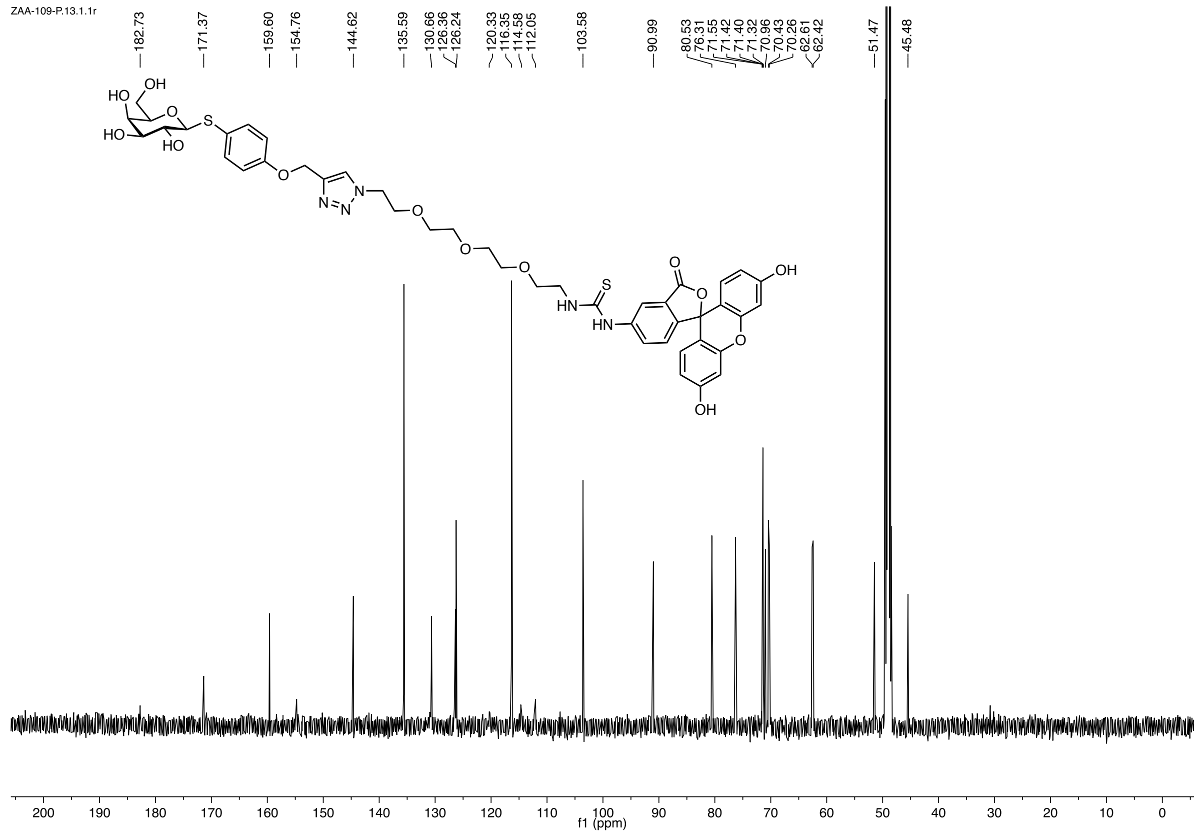

^{1}H and ^{13}C NMR of **4**

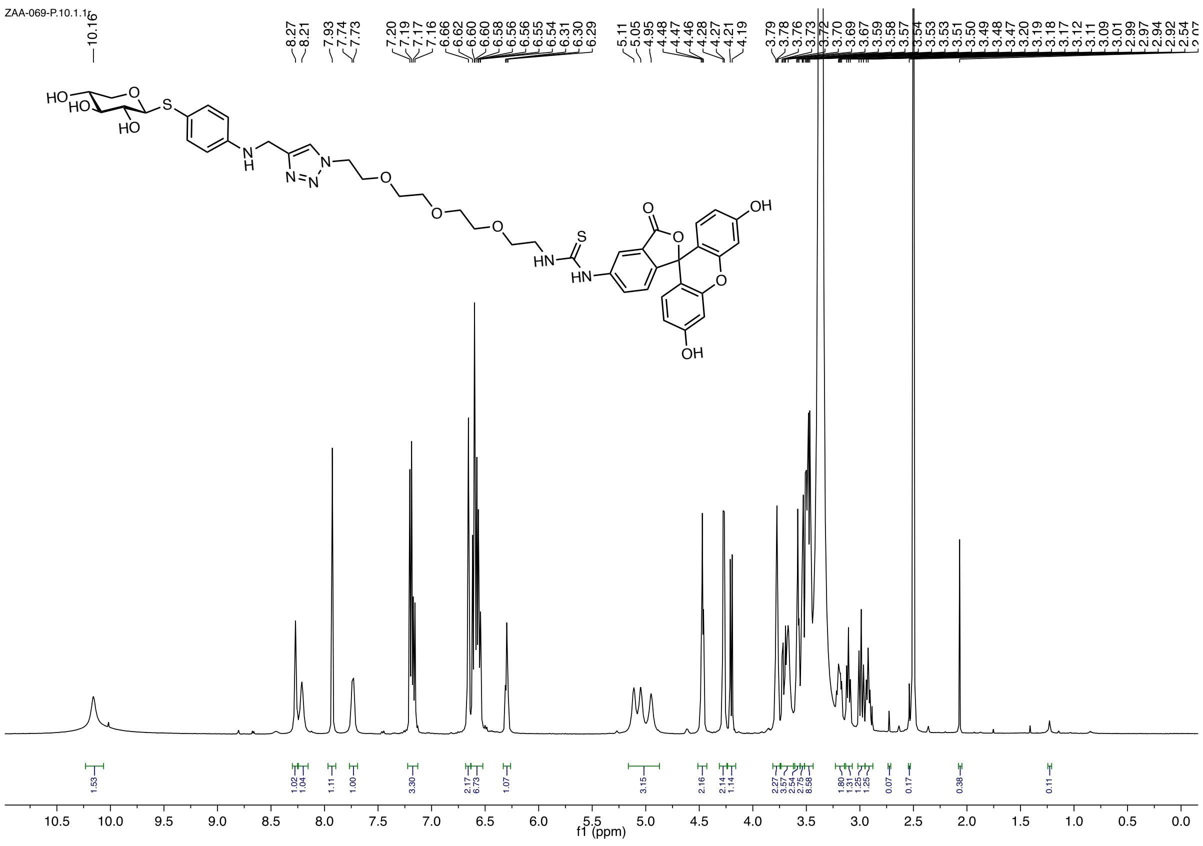

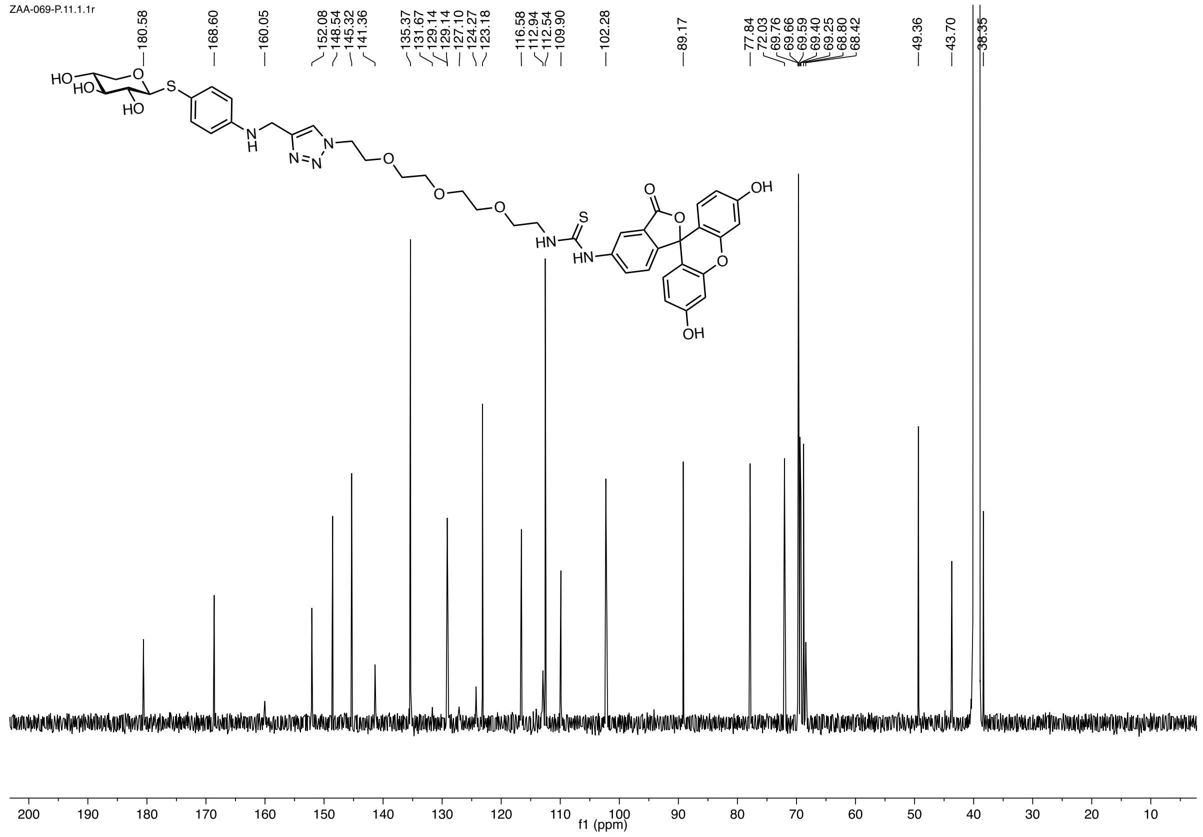

^{1}H and ^{13}C NMR of **8**

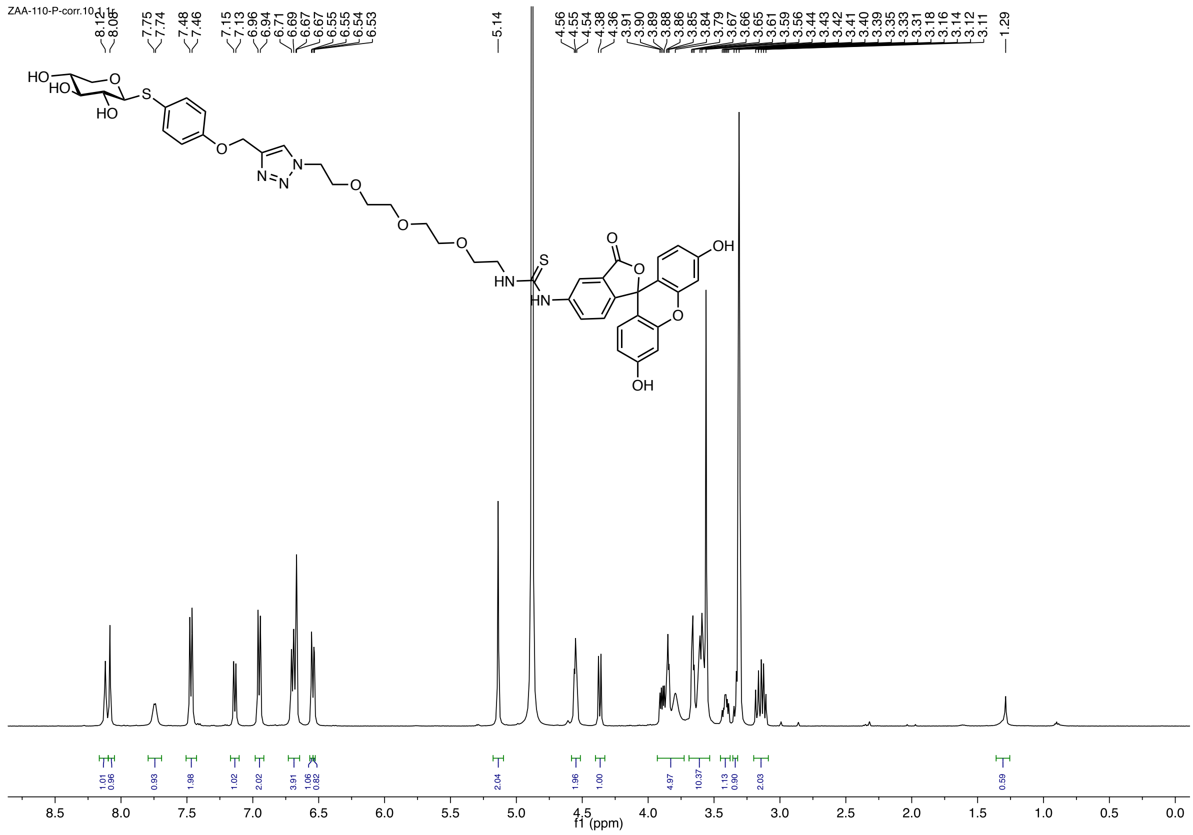

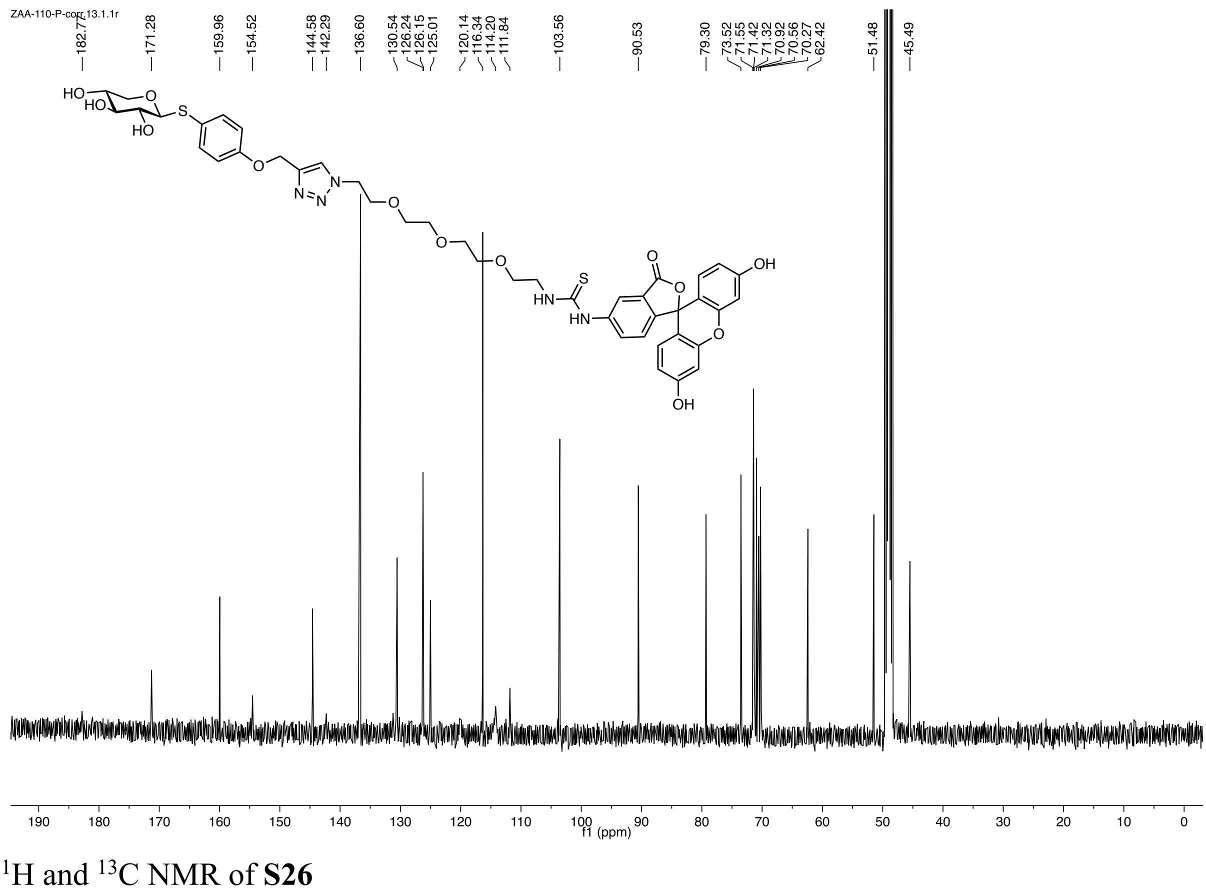

^{1}H and ^{13}C NMR of **S26**

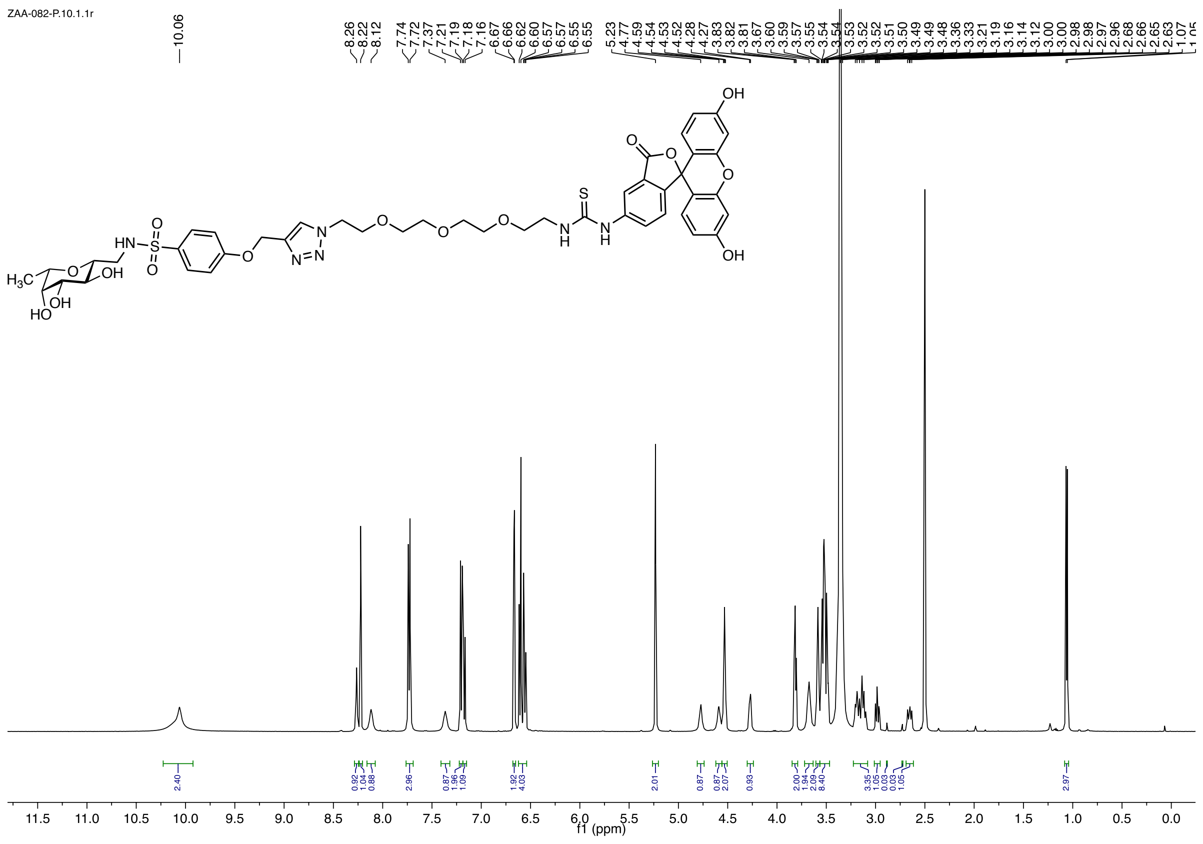

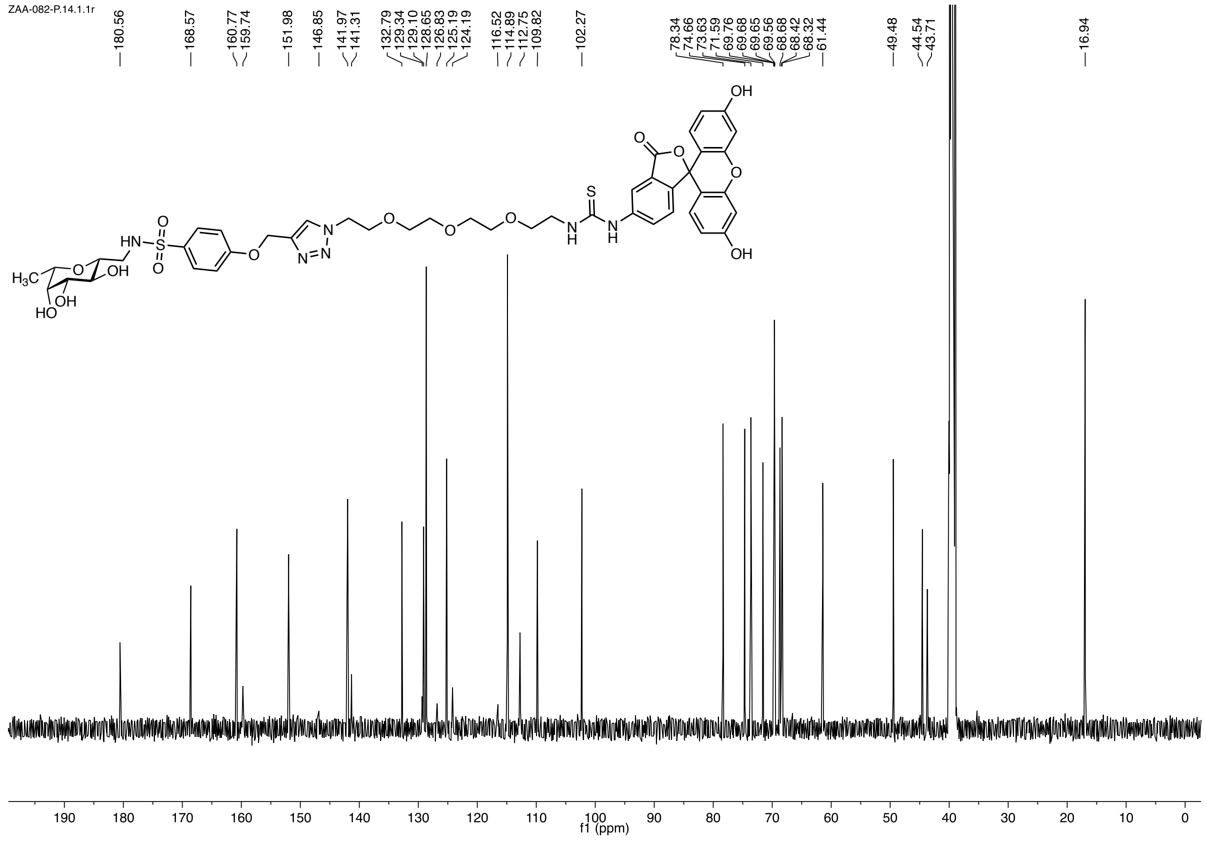

^{1}H and ^{13}C NMR of **7**

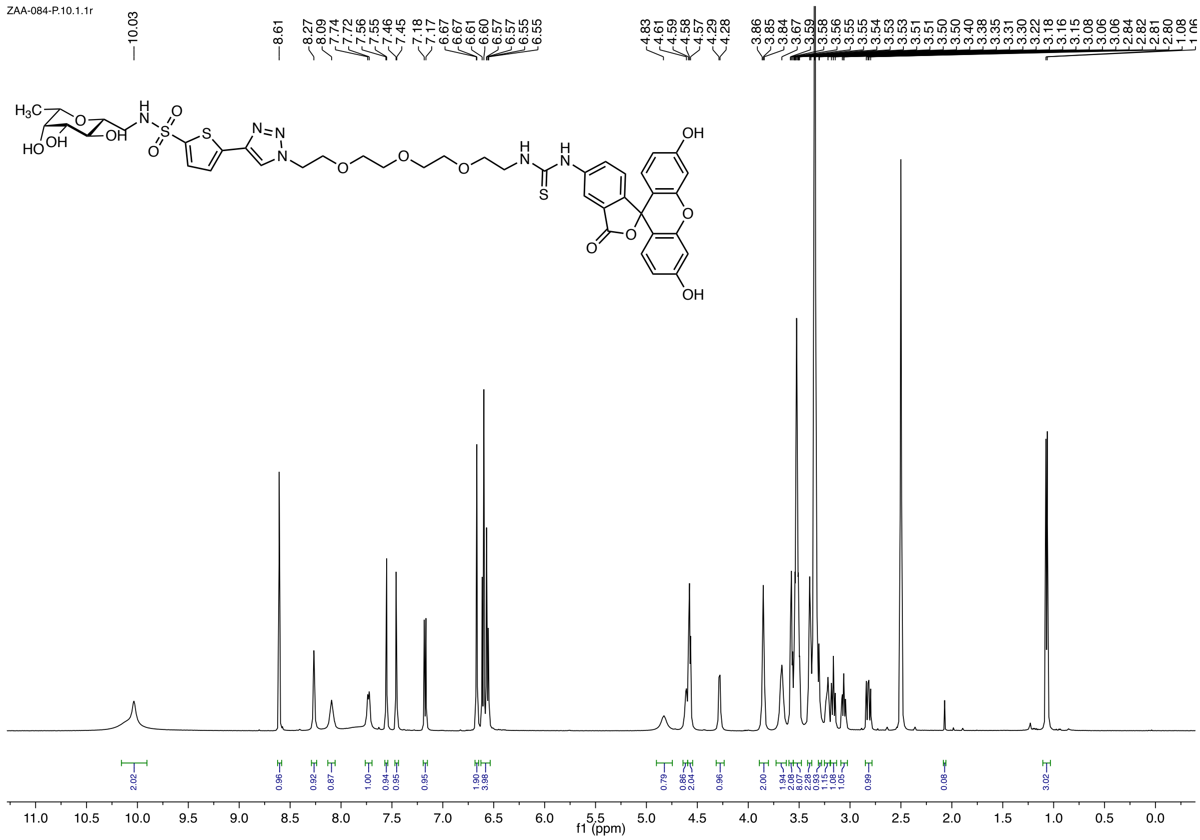

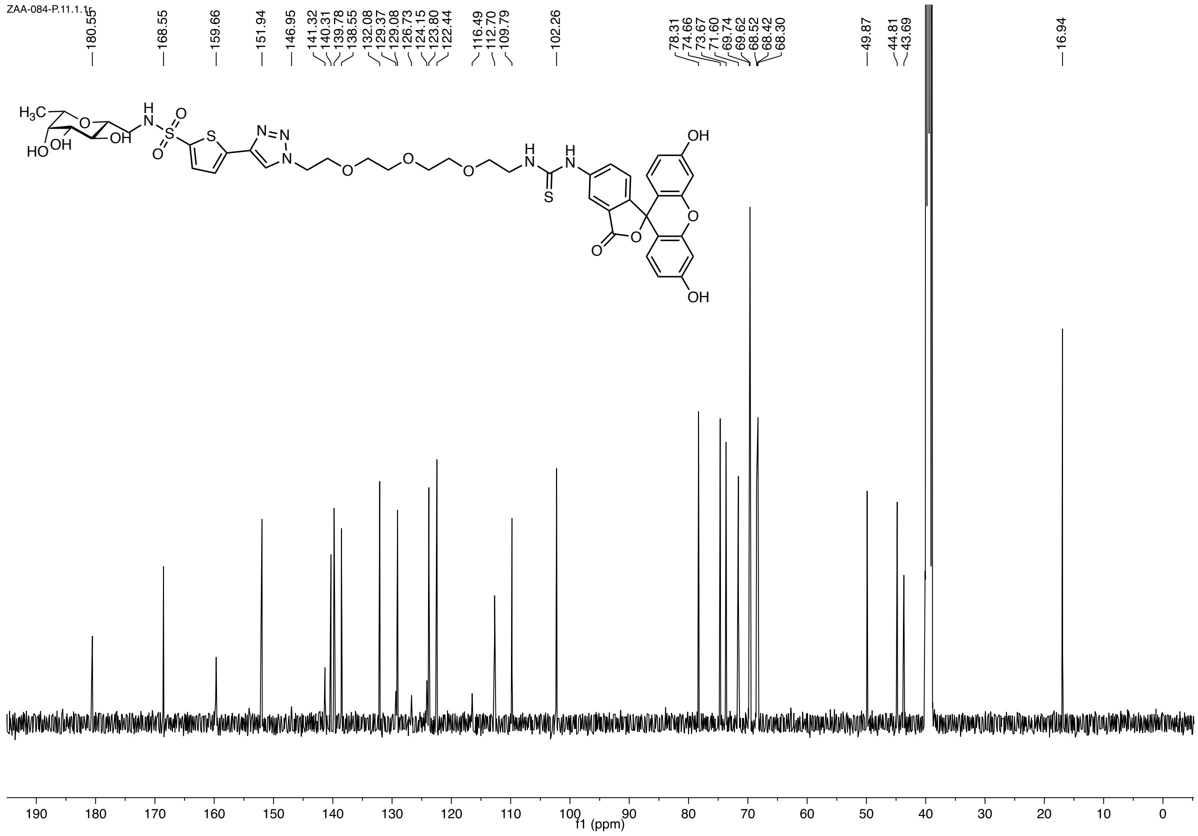

^{1}H and ^{13}C NMR of **6**

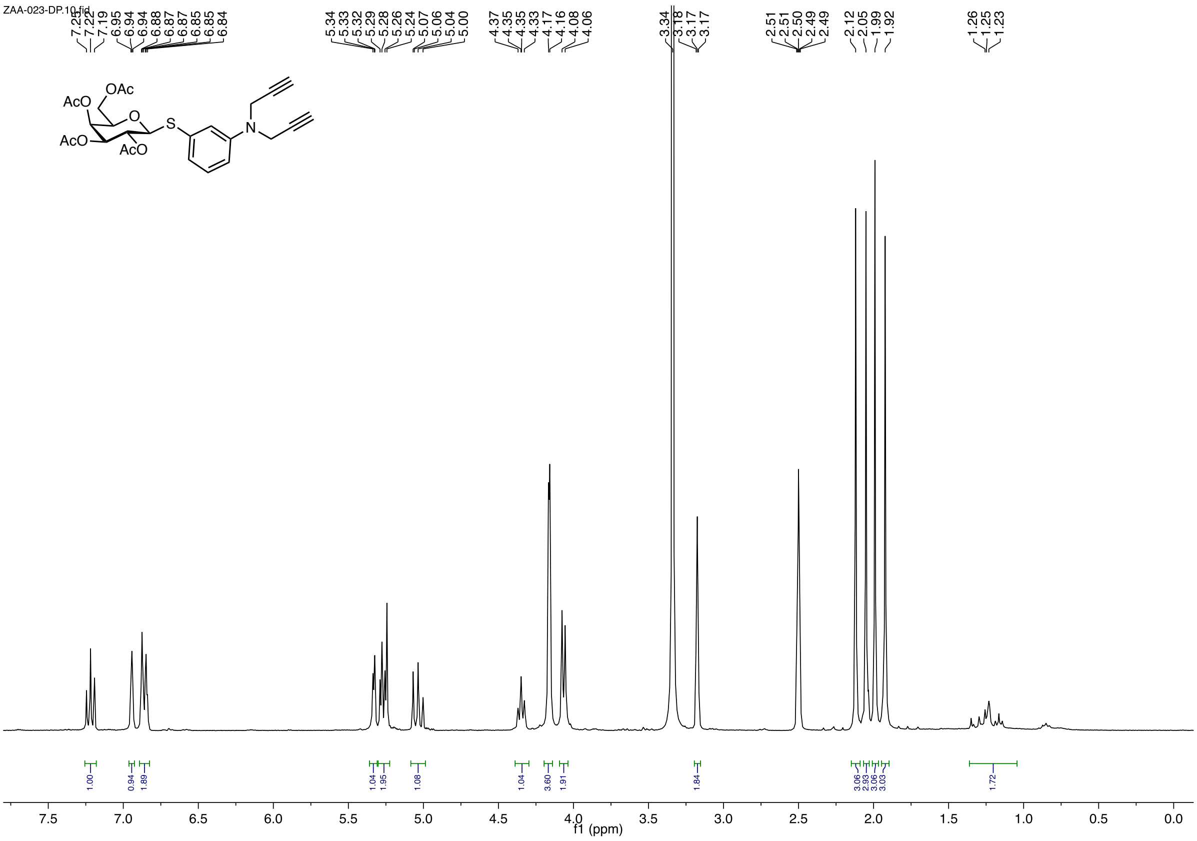

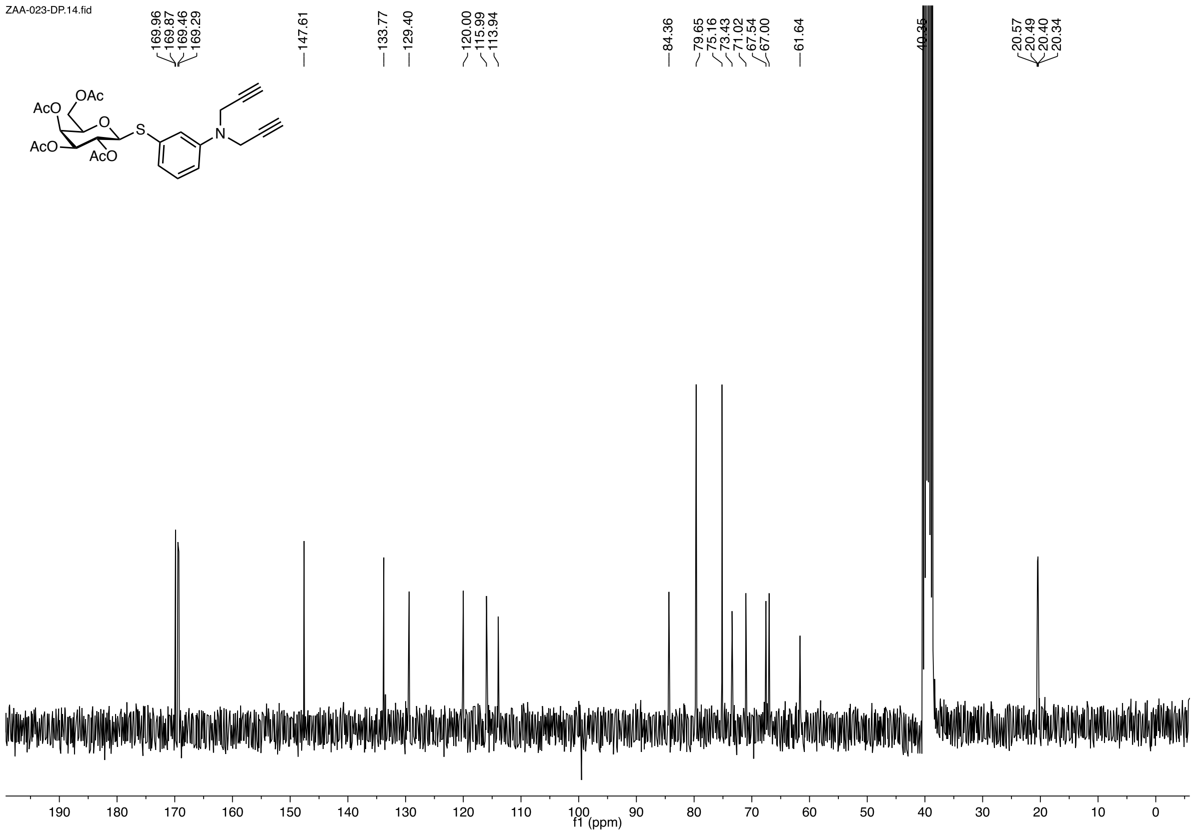

^{1}H and ^{13}C NMR of **S27*m***

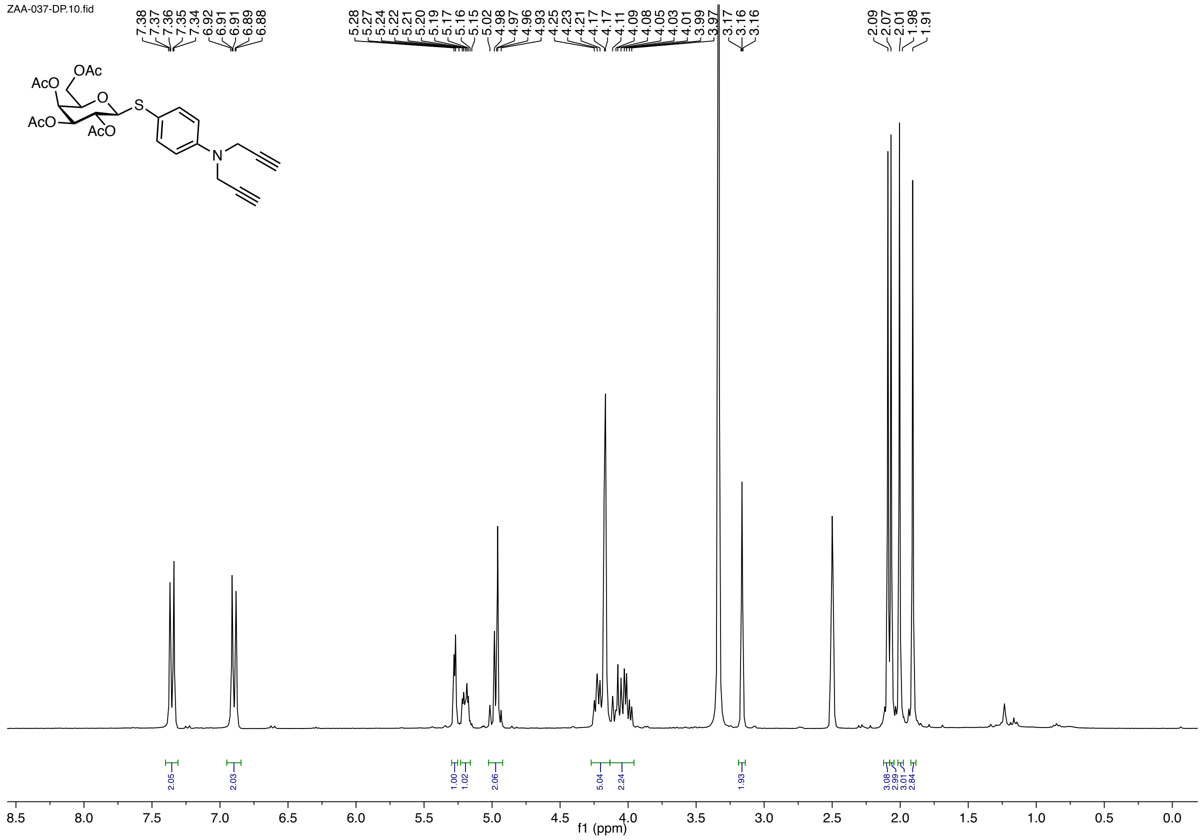

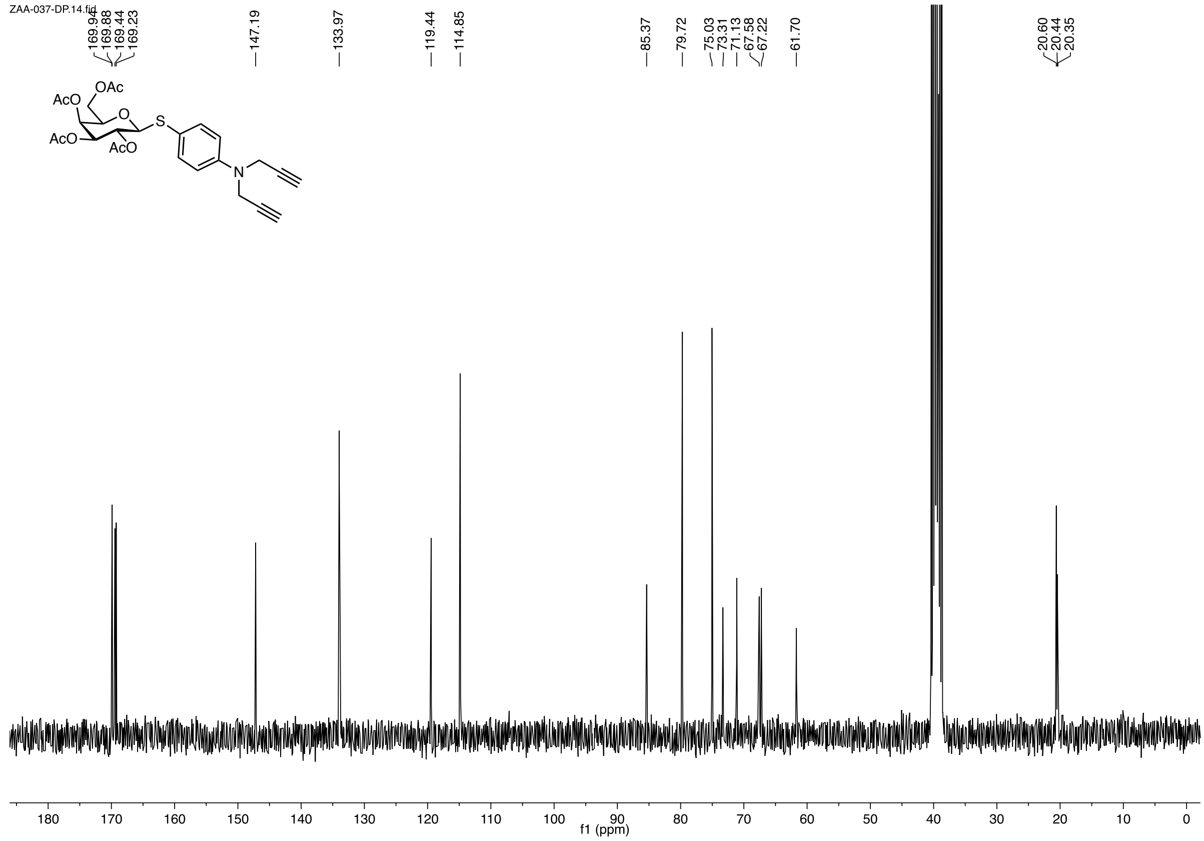

^{1}H and ^{13}C NMR of **S27*p***

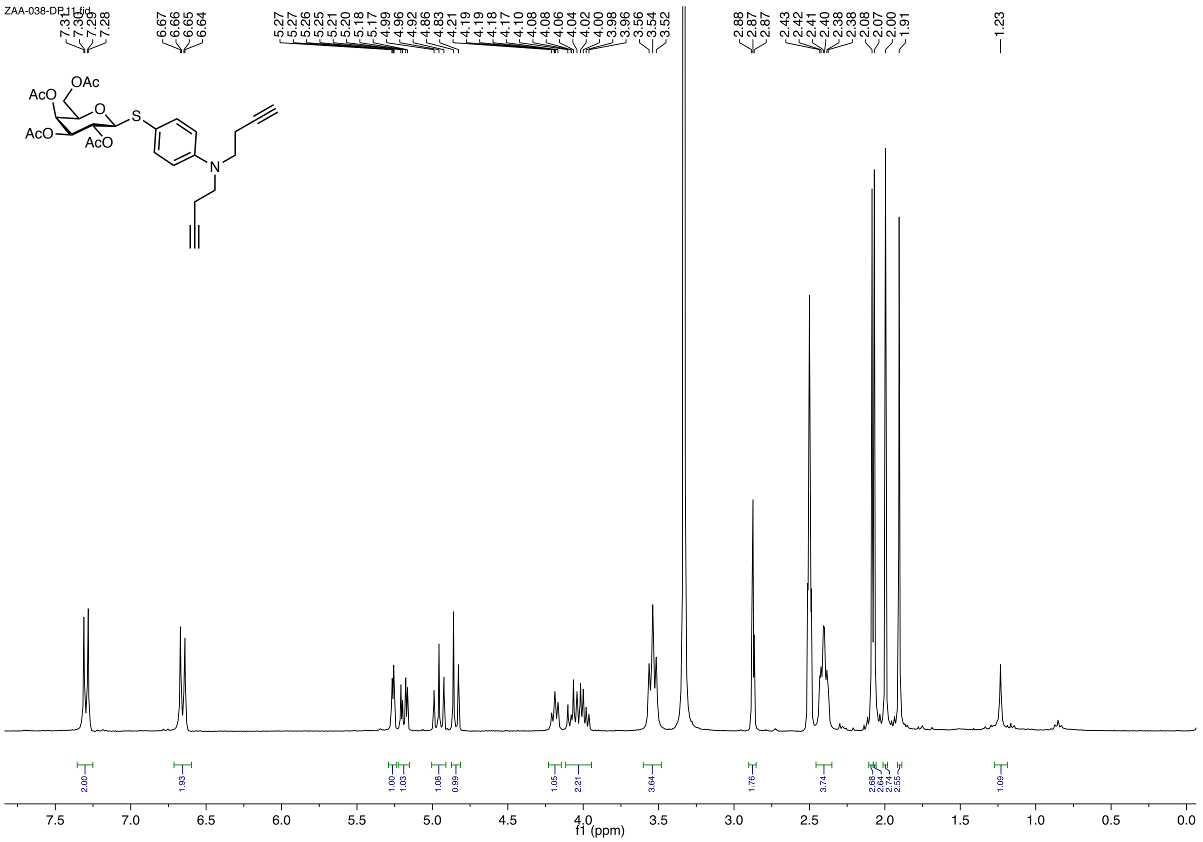

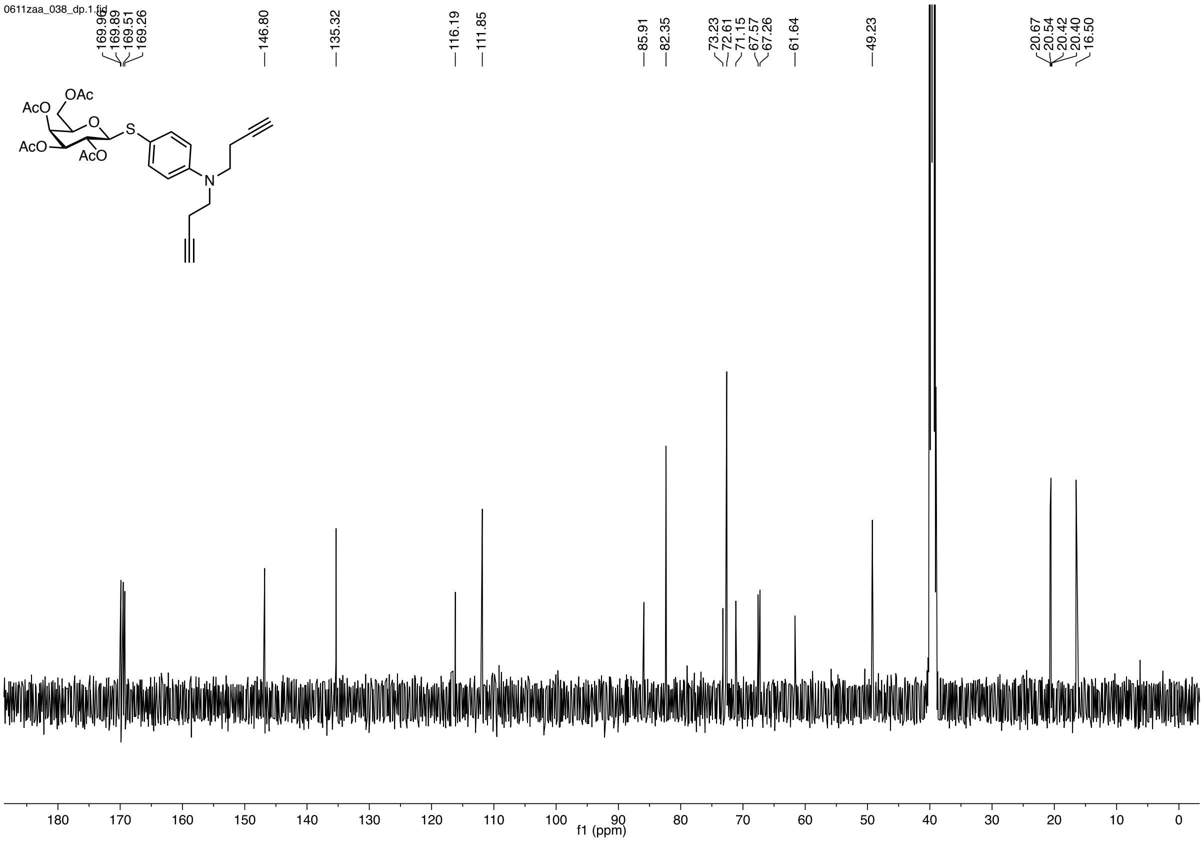

^{1}H and ^{13}C NMR of ***S28p***

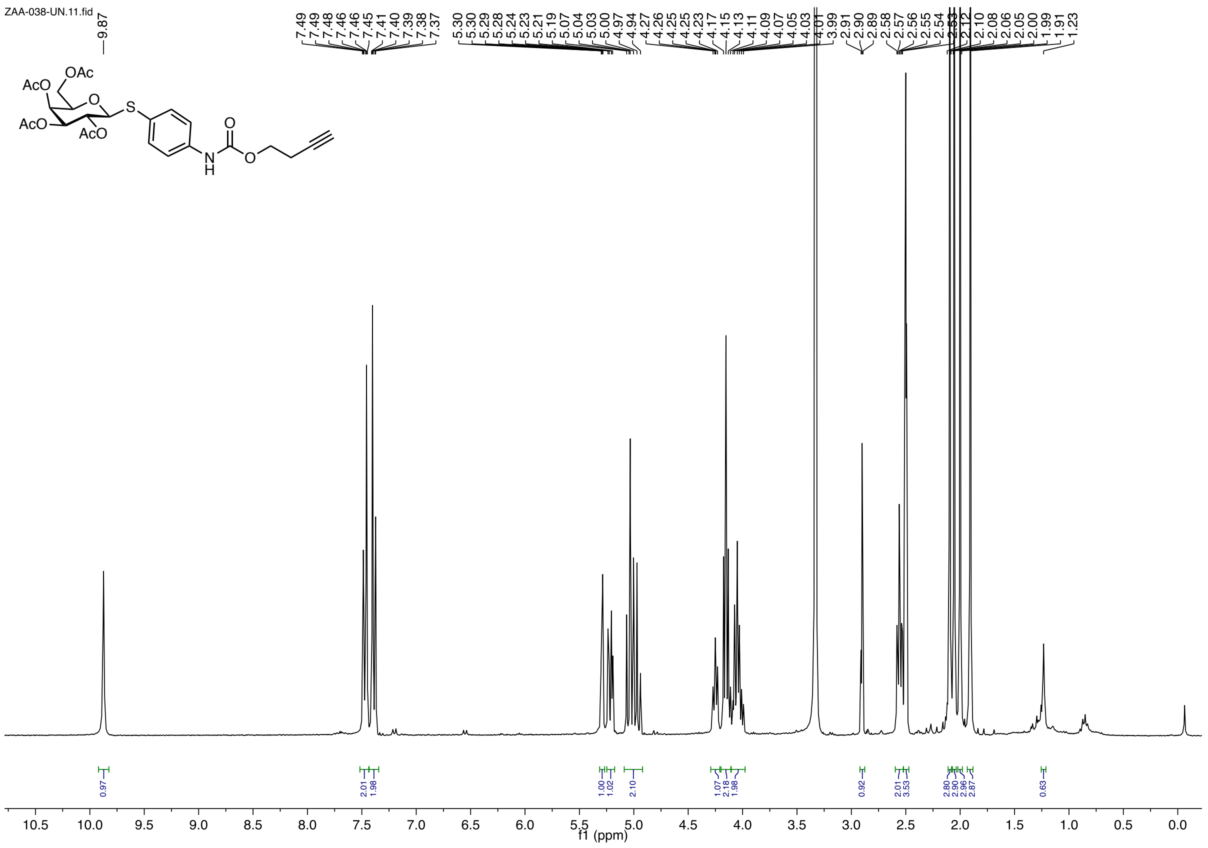

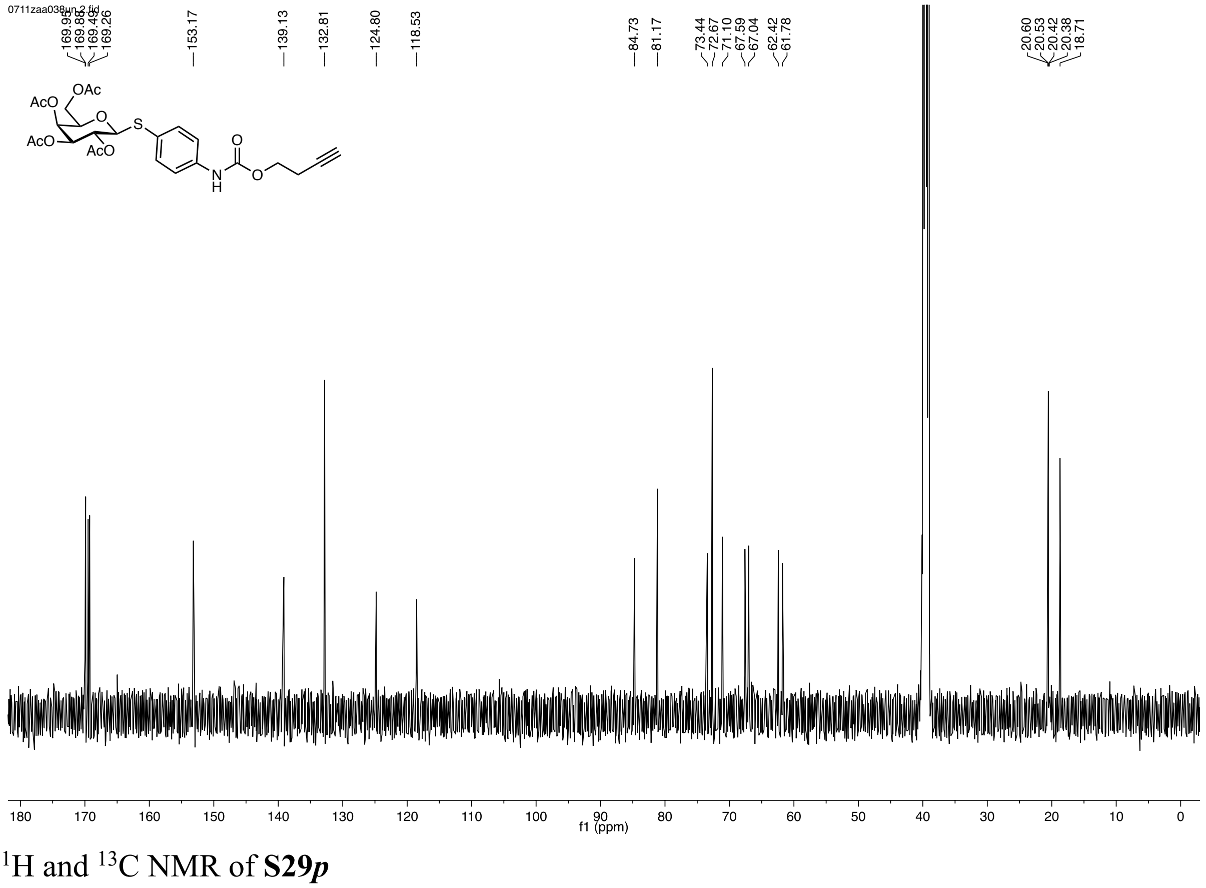

^{1}H and ^{13}C NMR of **S29*p***

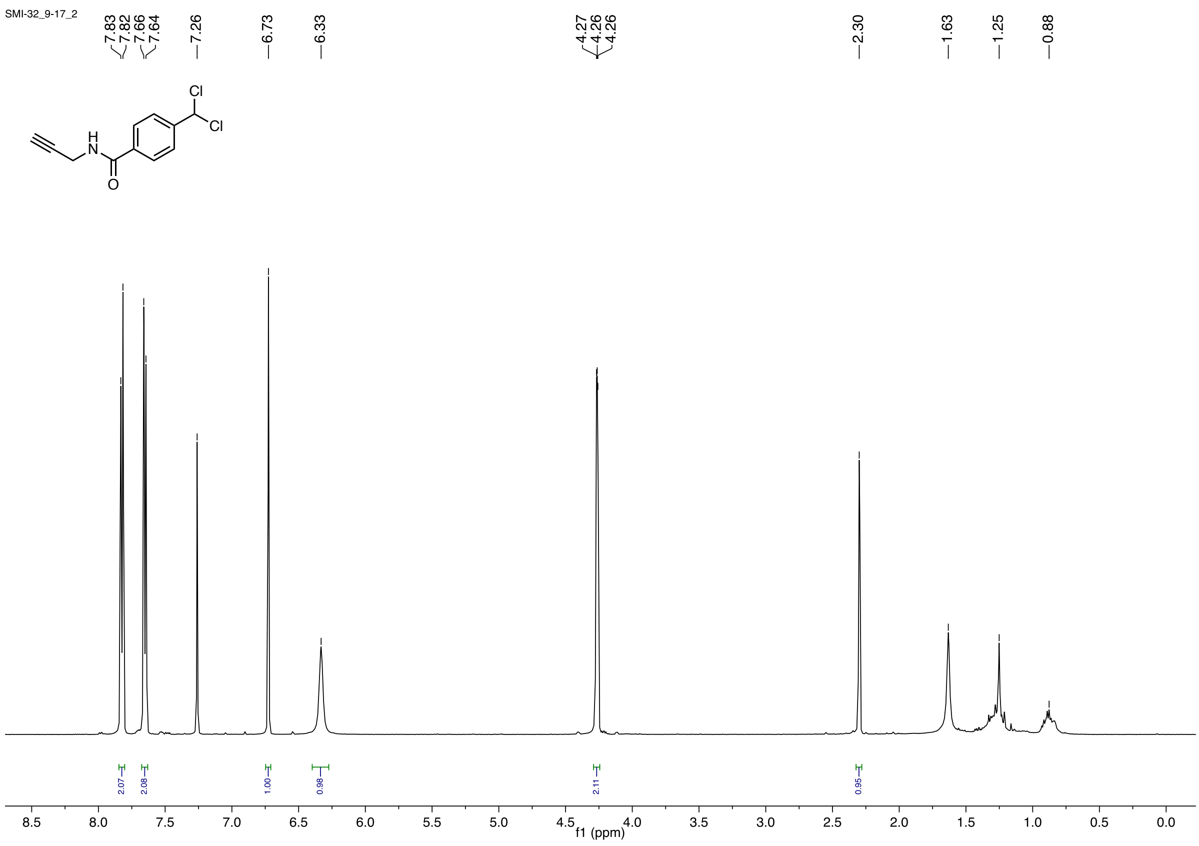

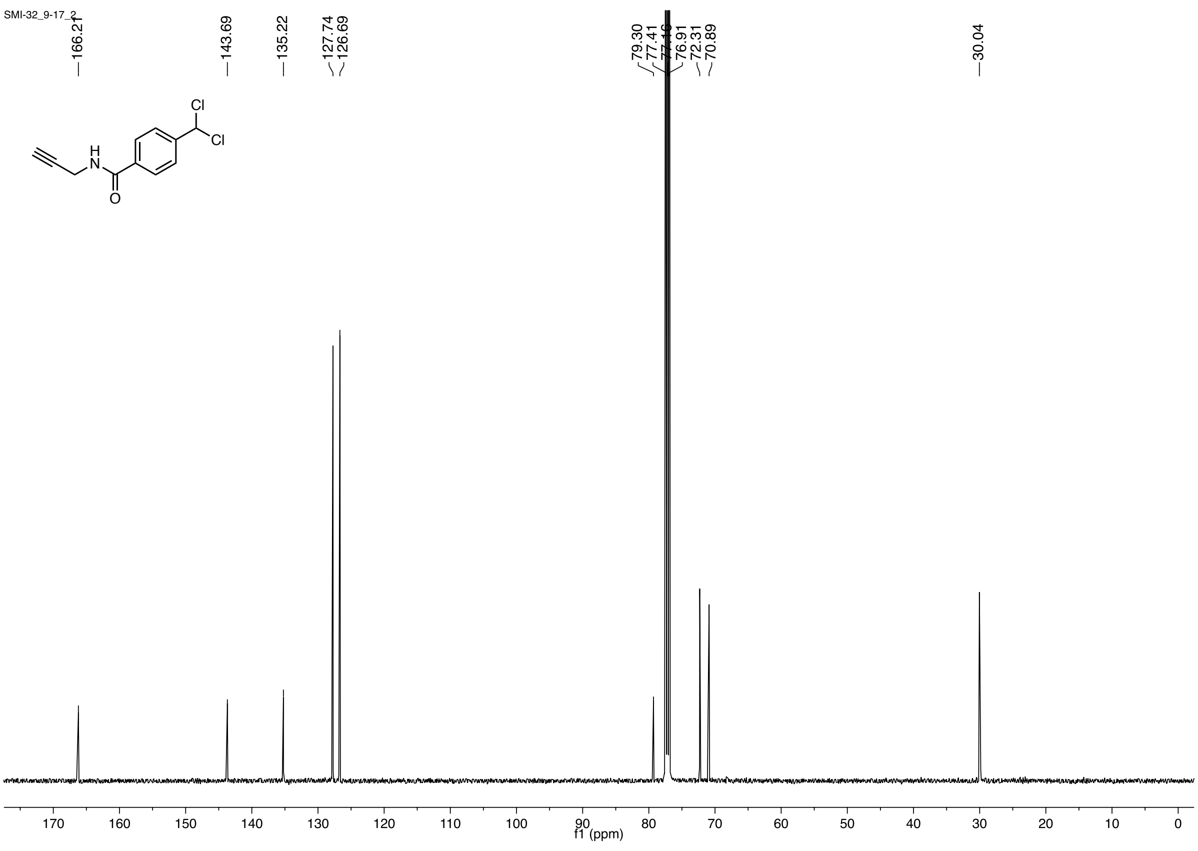

^{1}H and ^{13}C NMR of **S33**

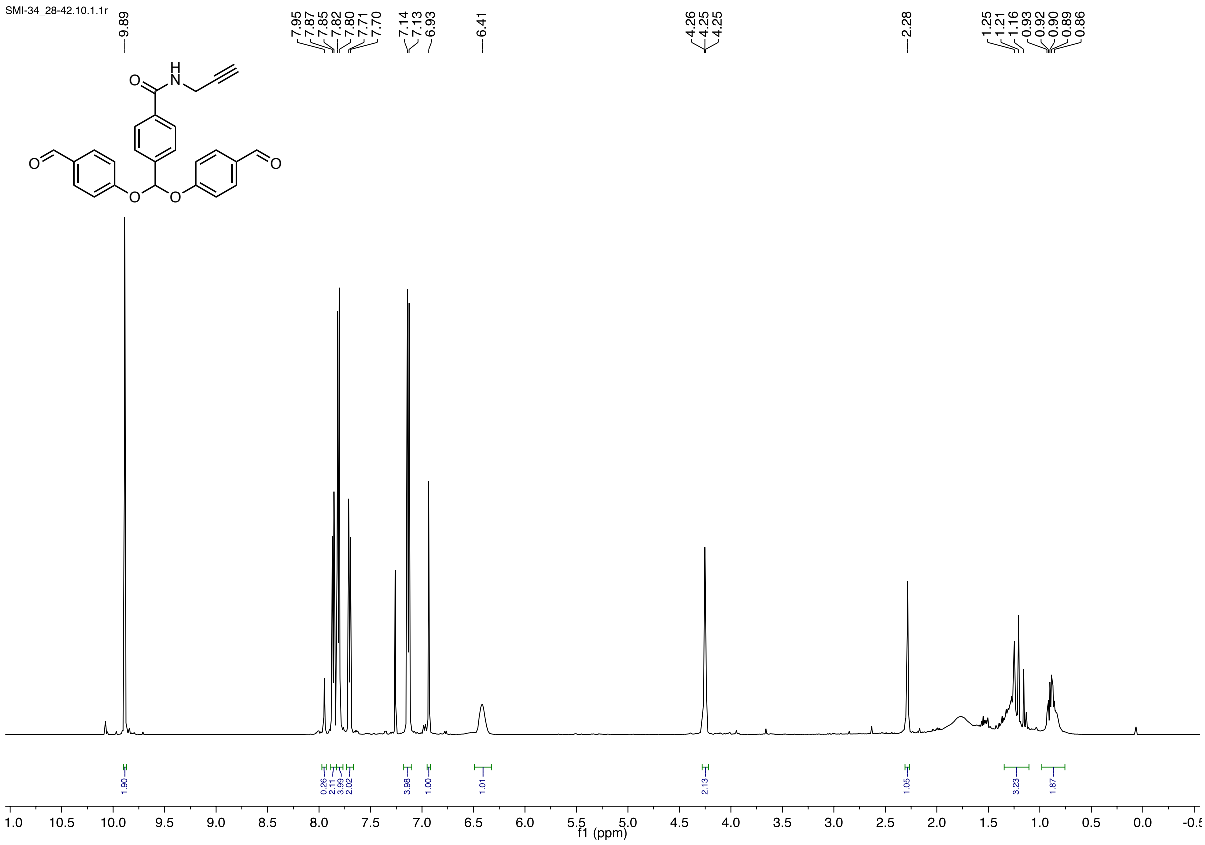

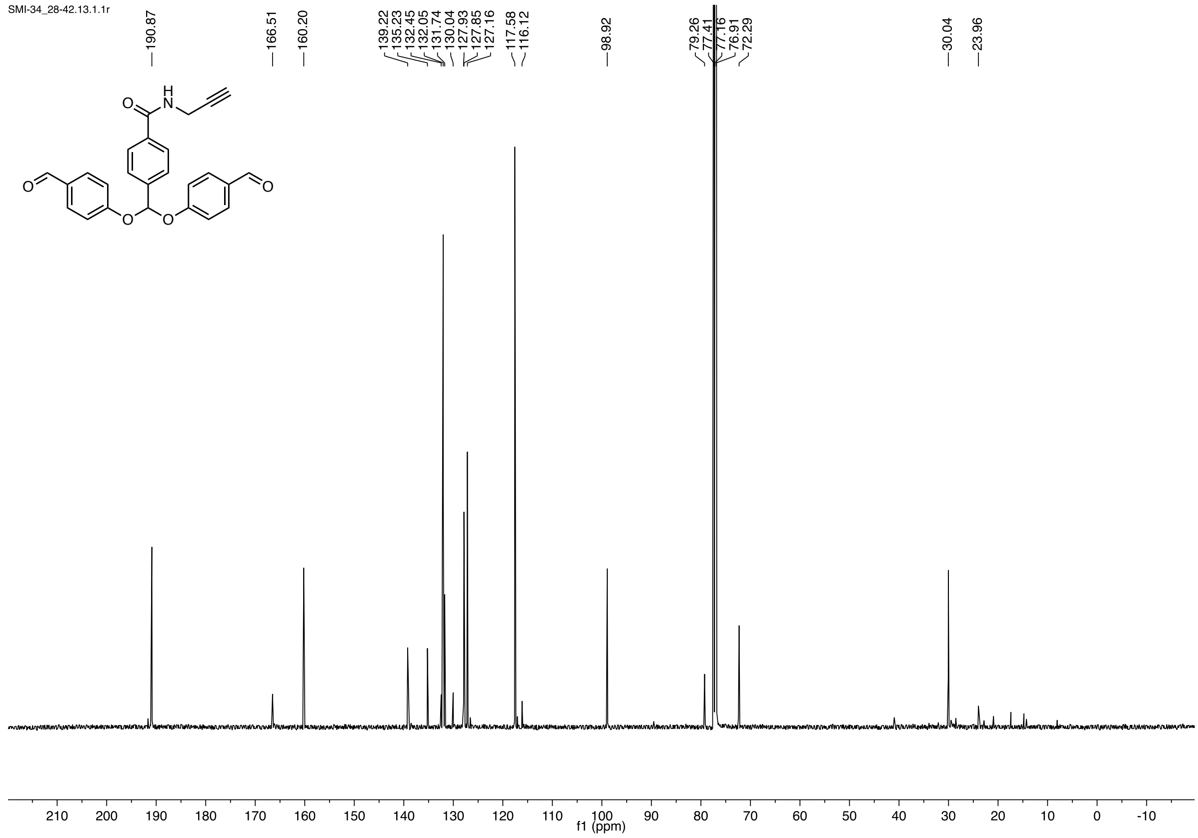

^{1}H and ^{13}C NMR of **S34**

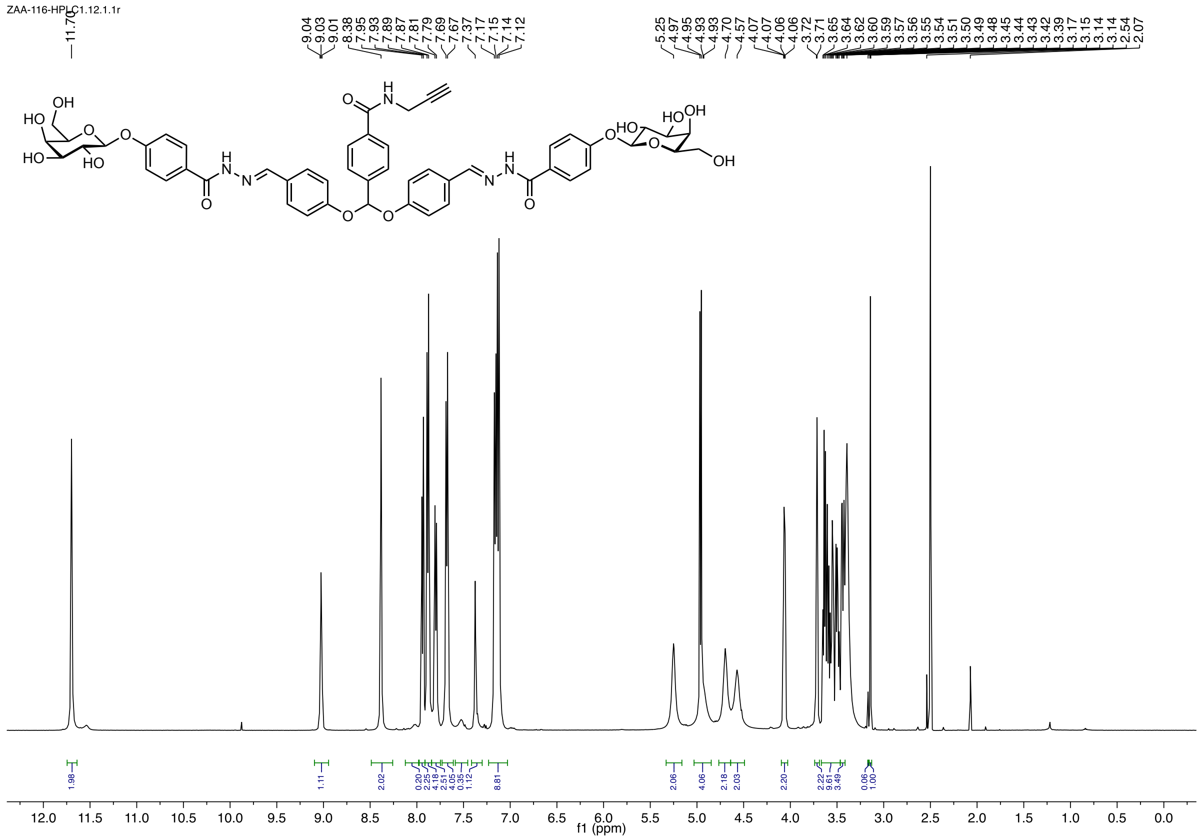

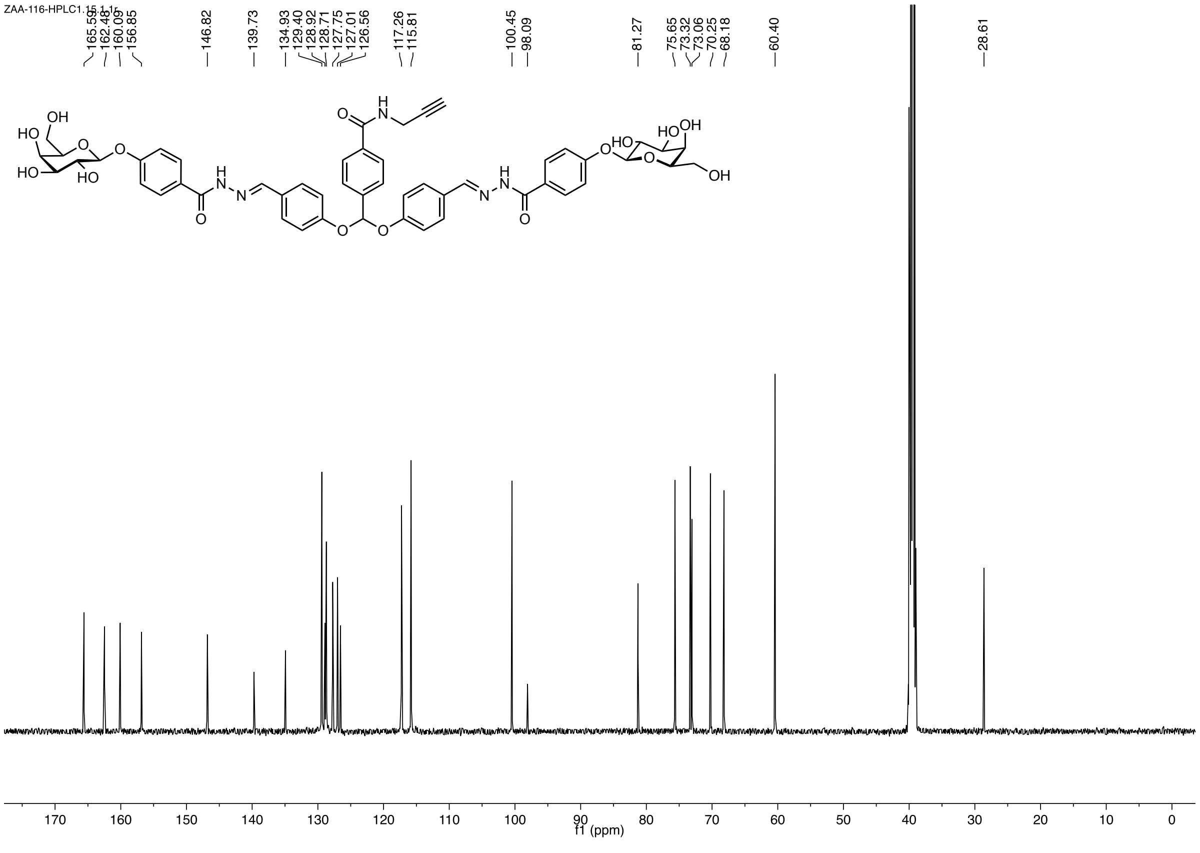

^{1}H and ^{13}C NMR of **S36**

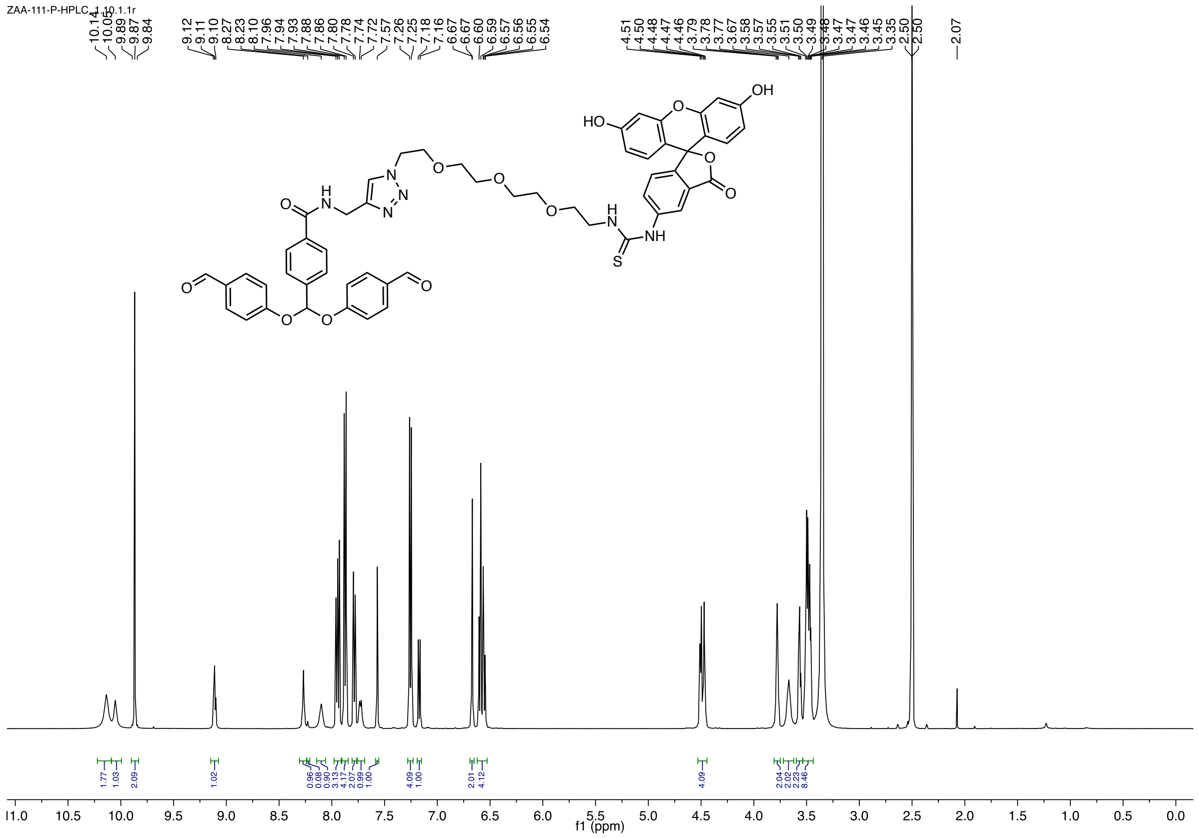

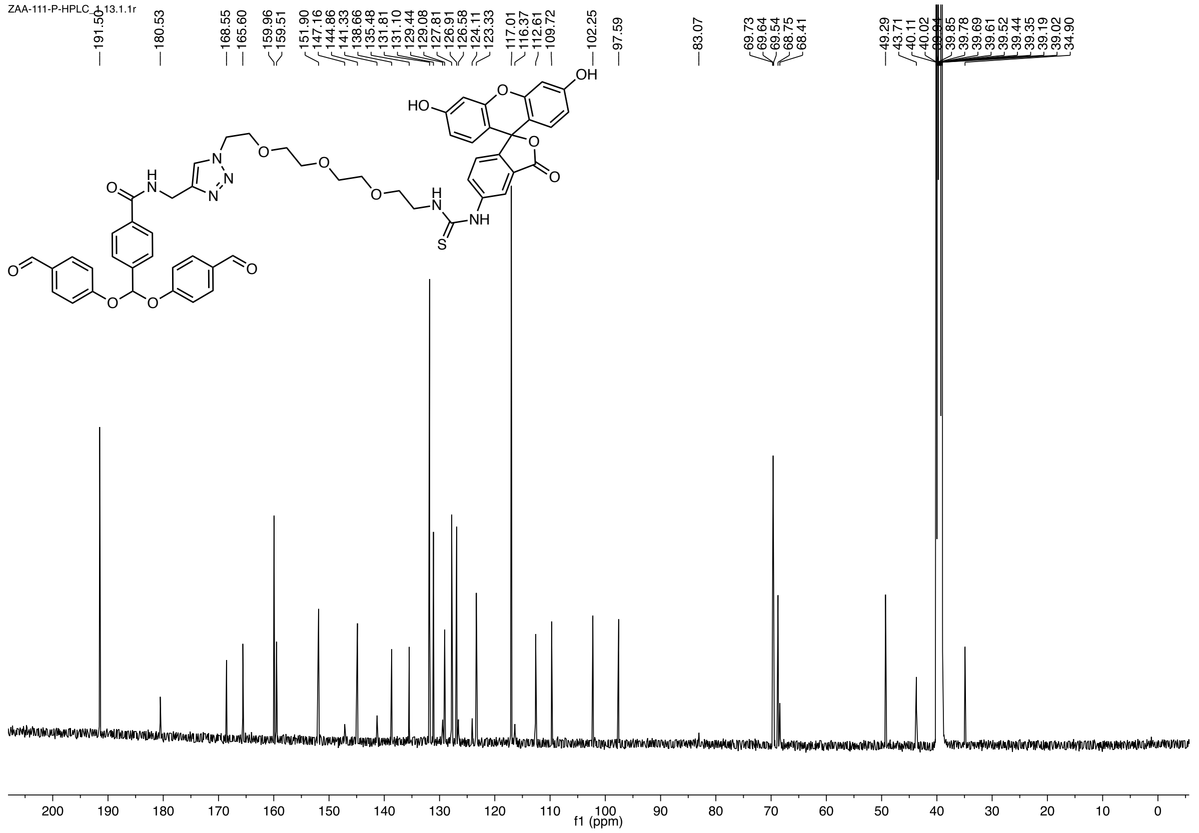

^{1}H and ^{13}C NMR of **S37**

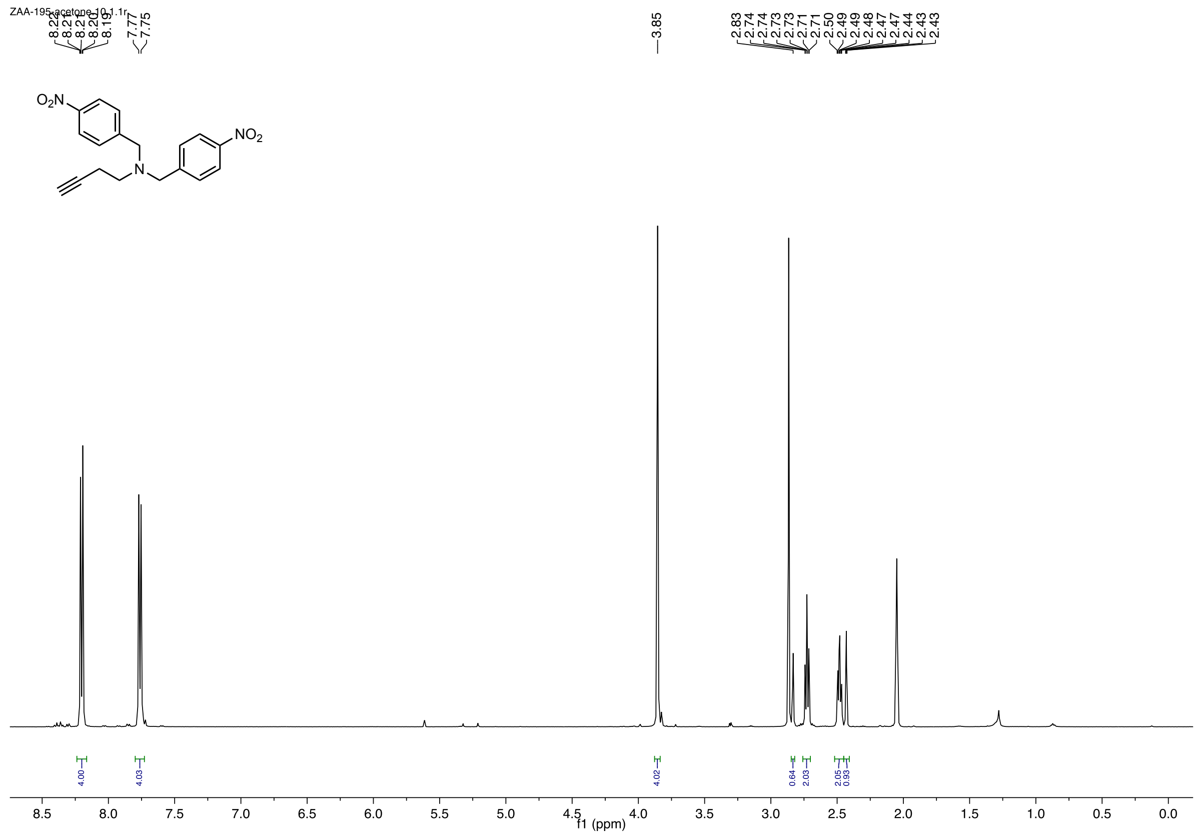

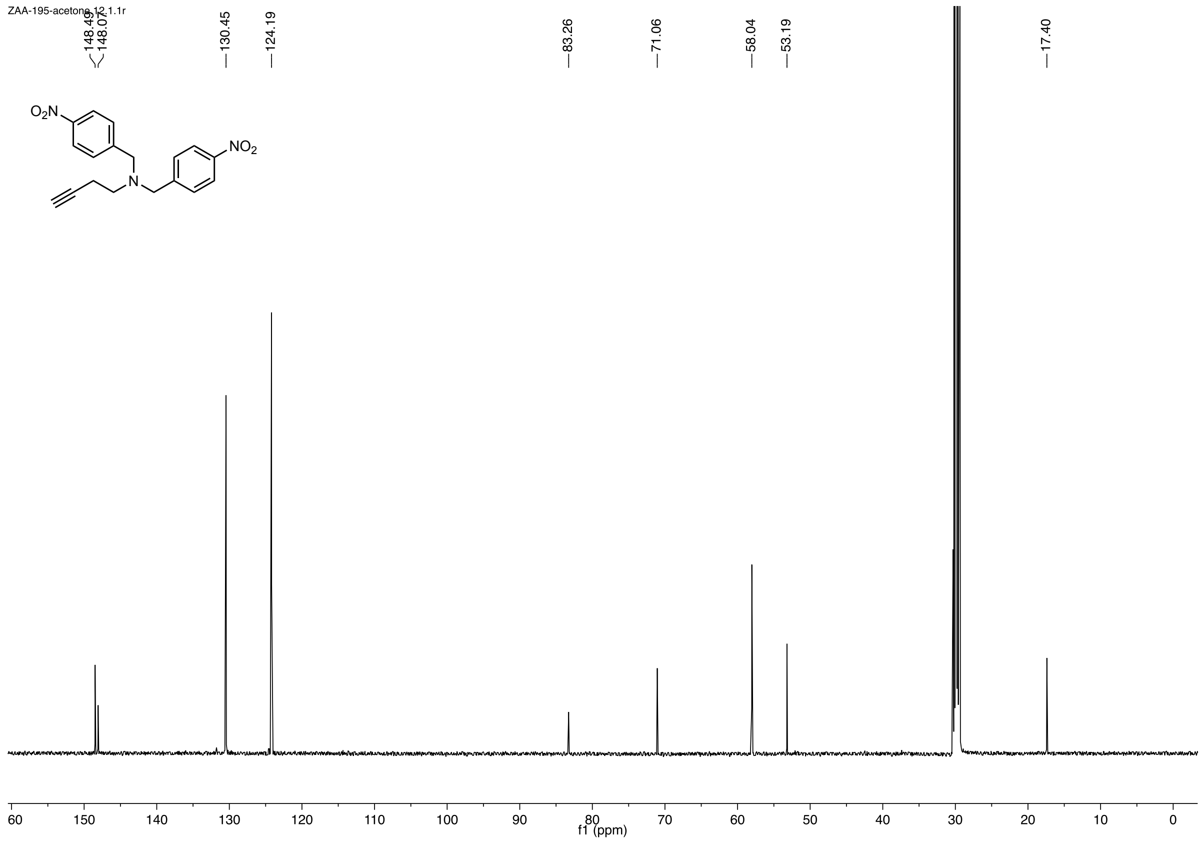

^{1}H and ^{13}C NMR of **11**

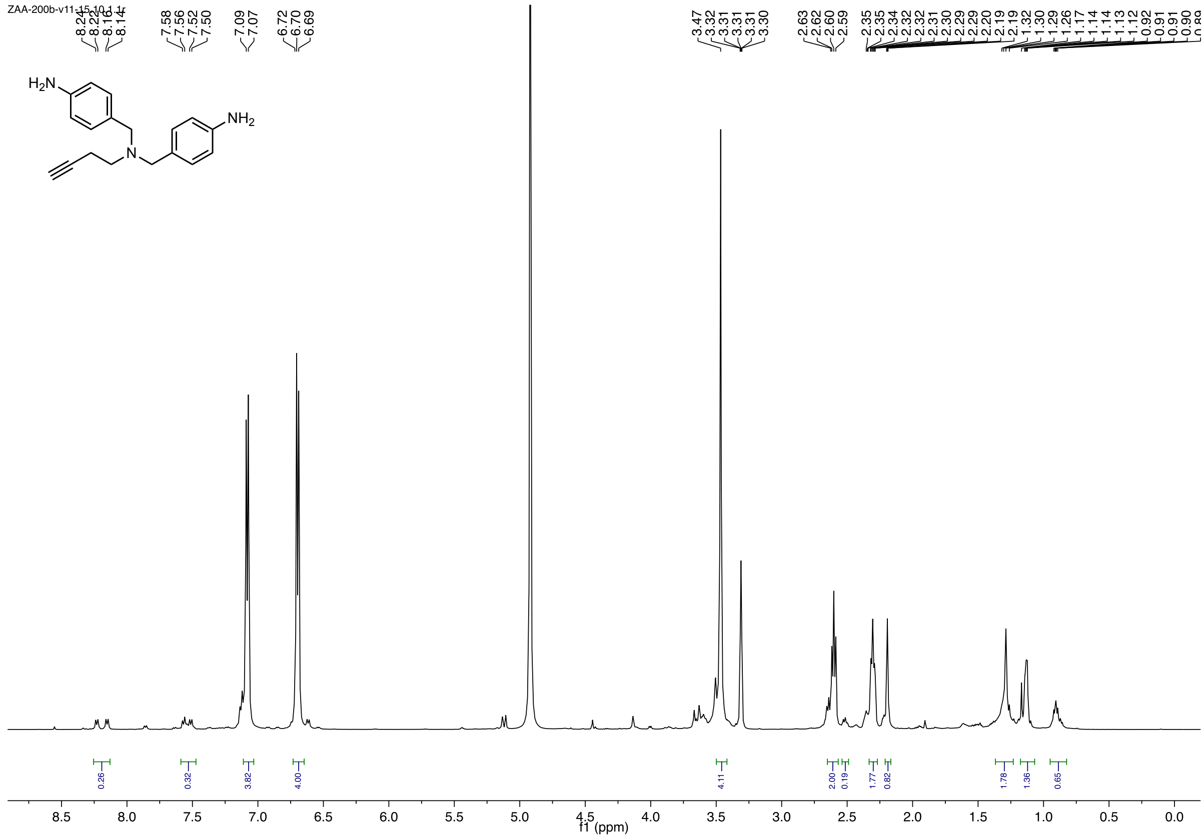

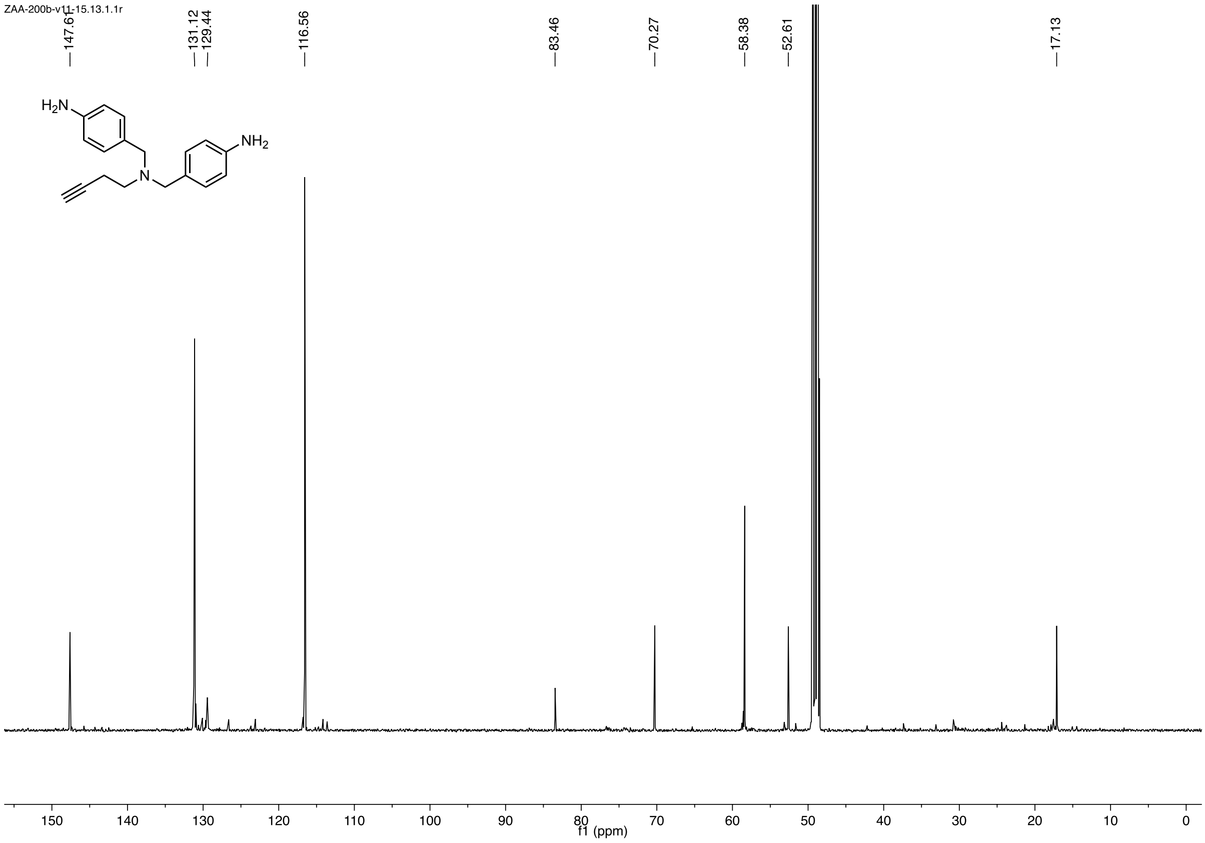

^{1}H and ^{13}C NMR of **12**

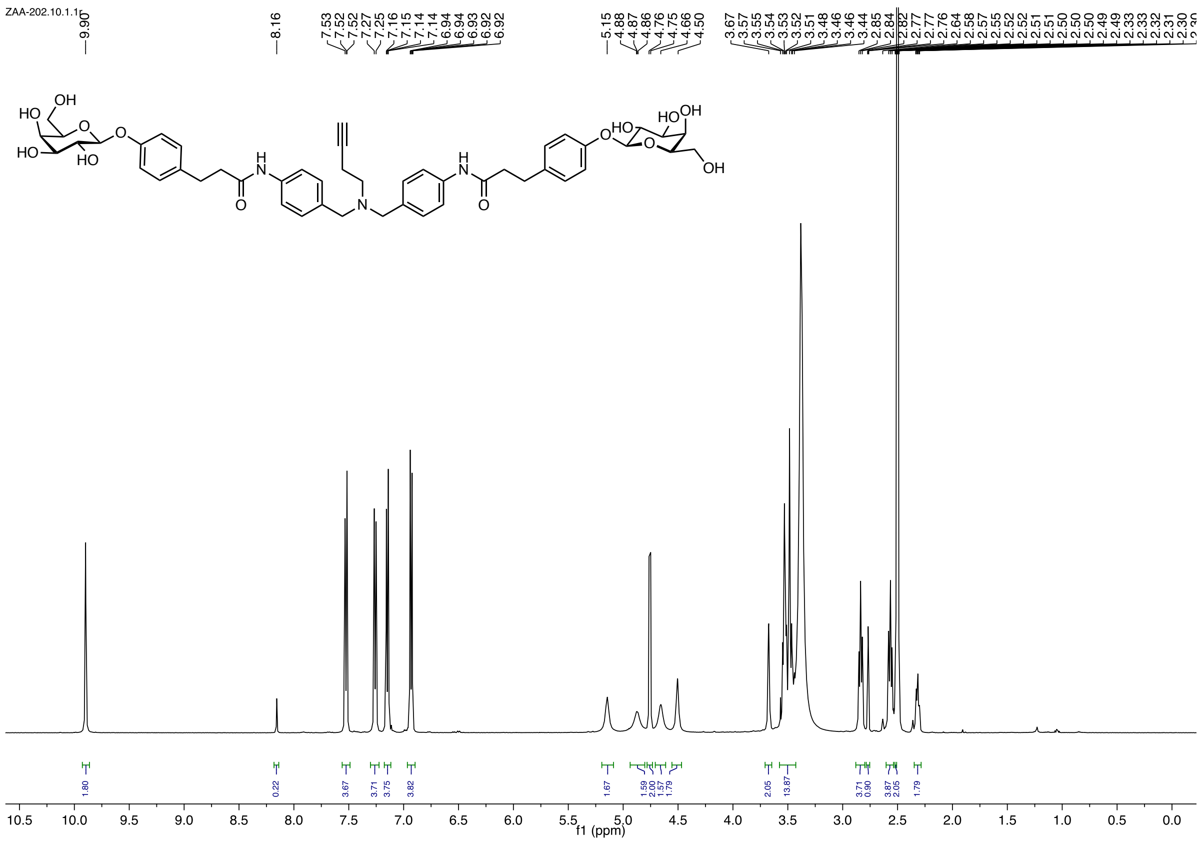

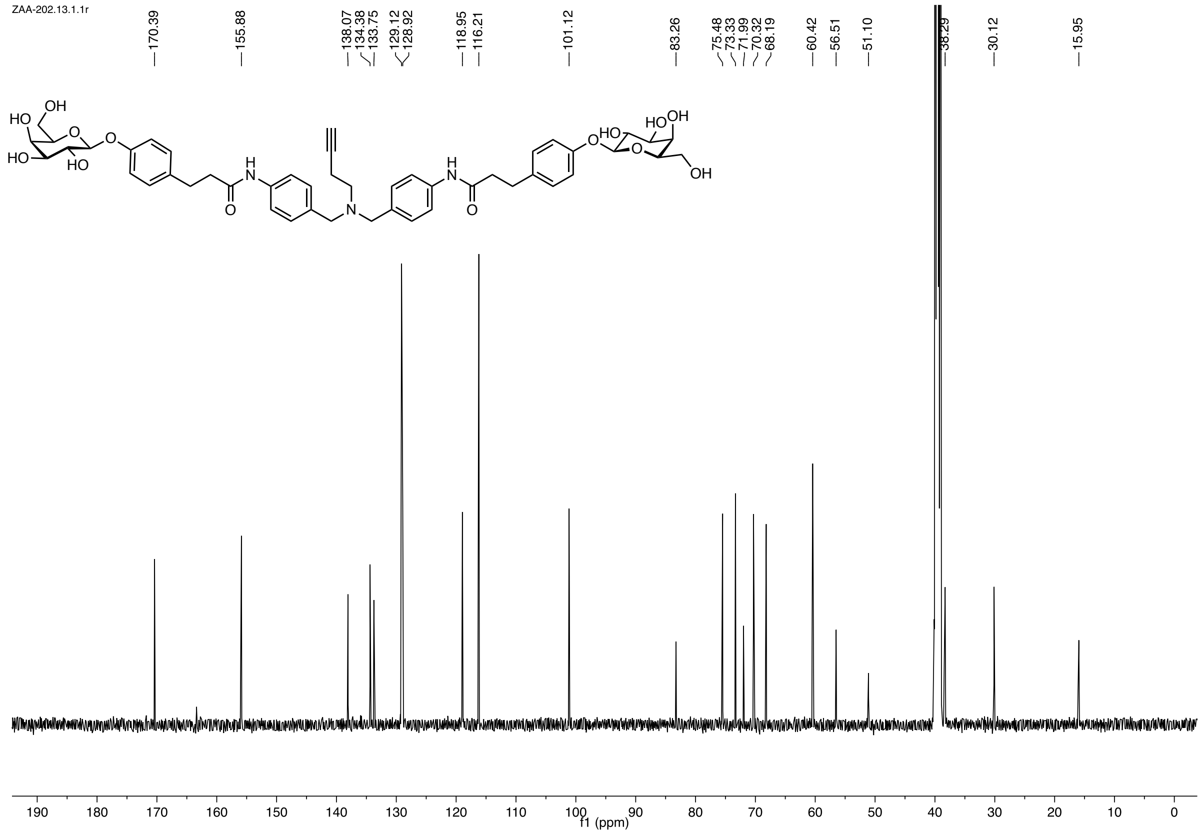

^{1}H and ^{13}C NMR of **16**

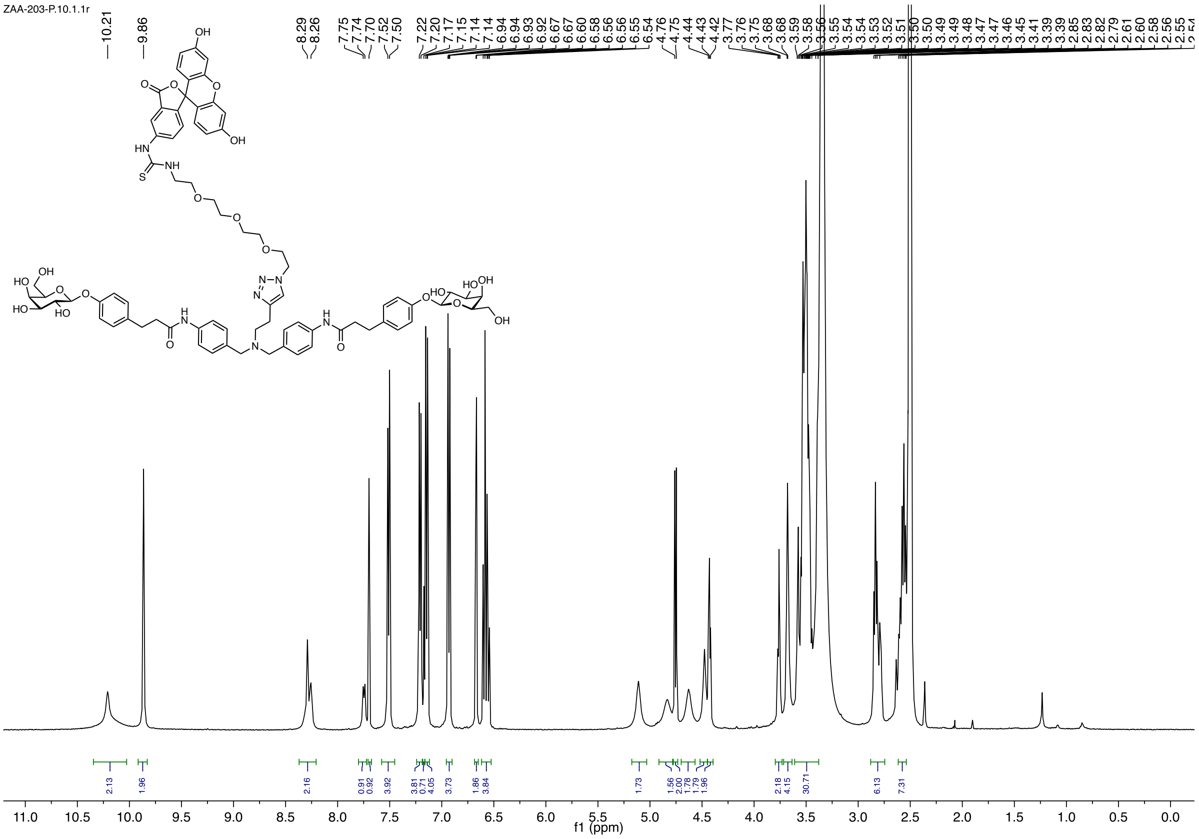

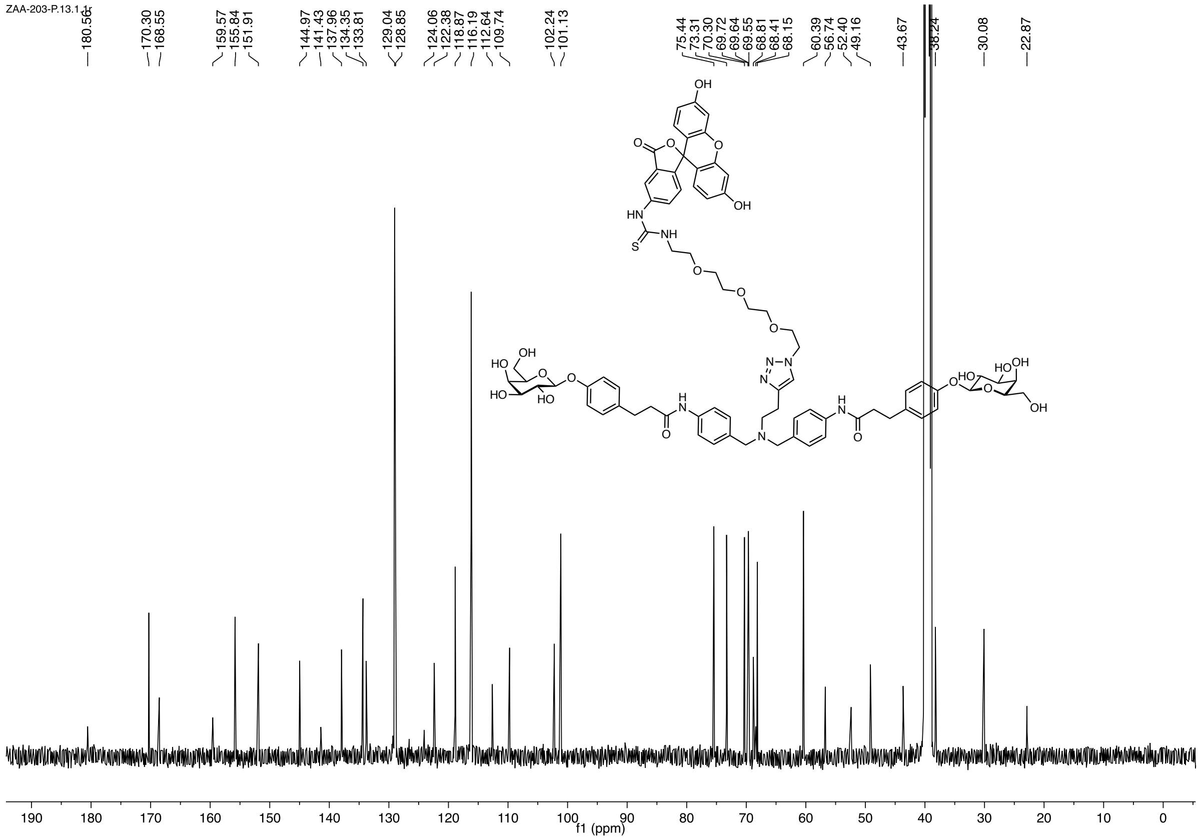

^{1}H and ^{13}C NMR of **17**

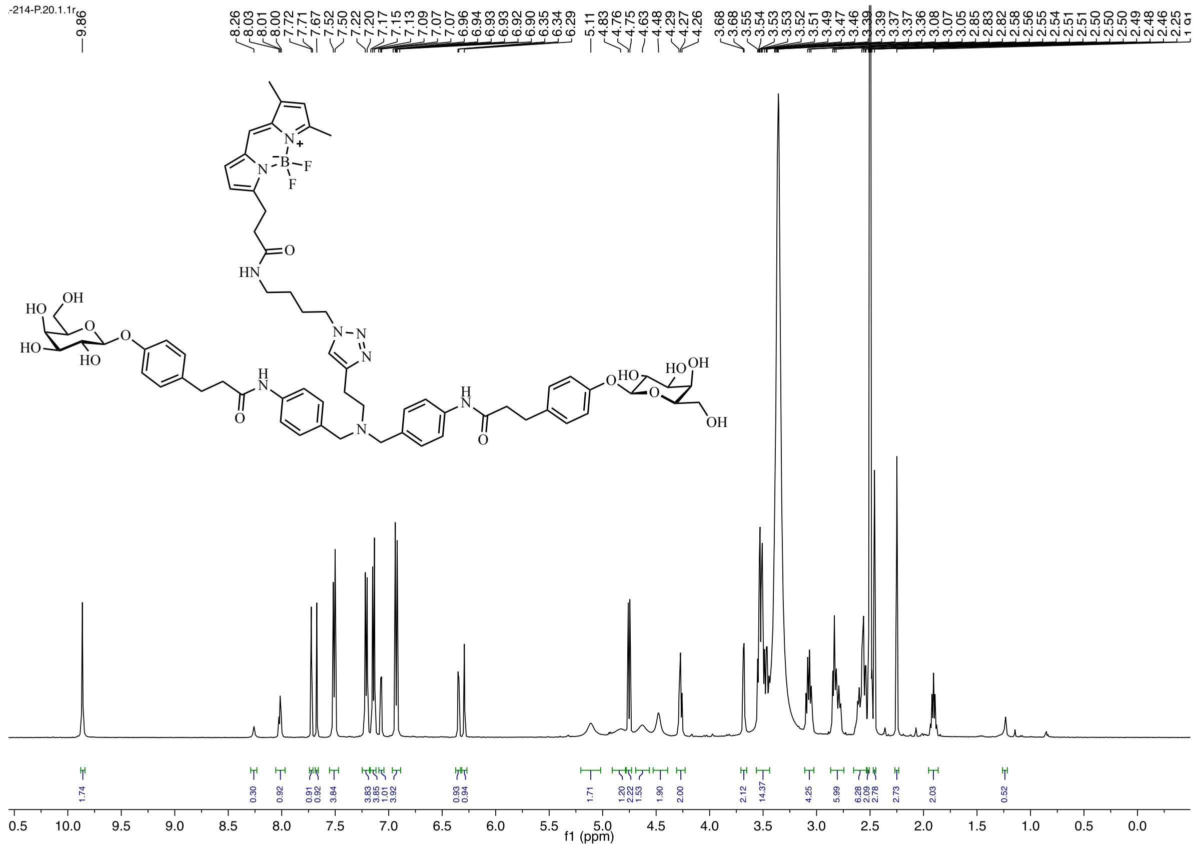

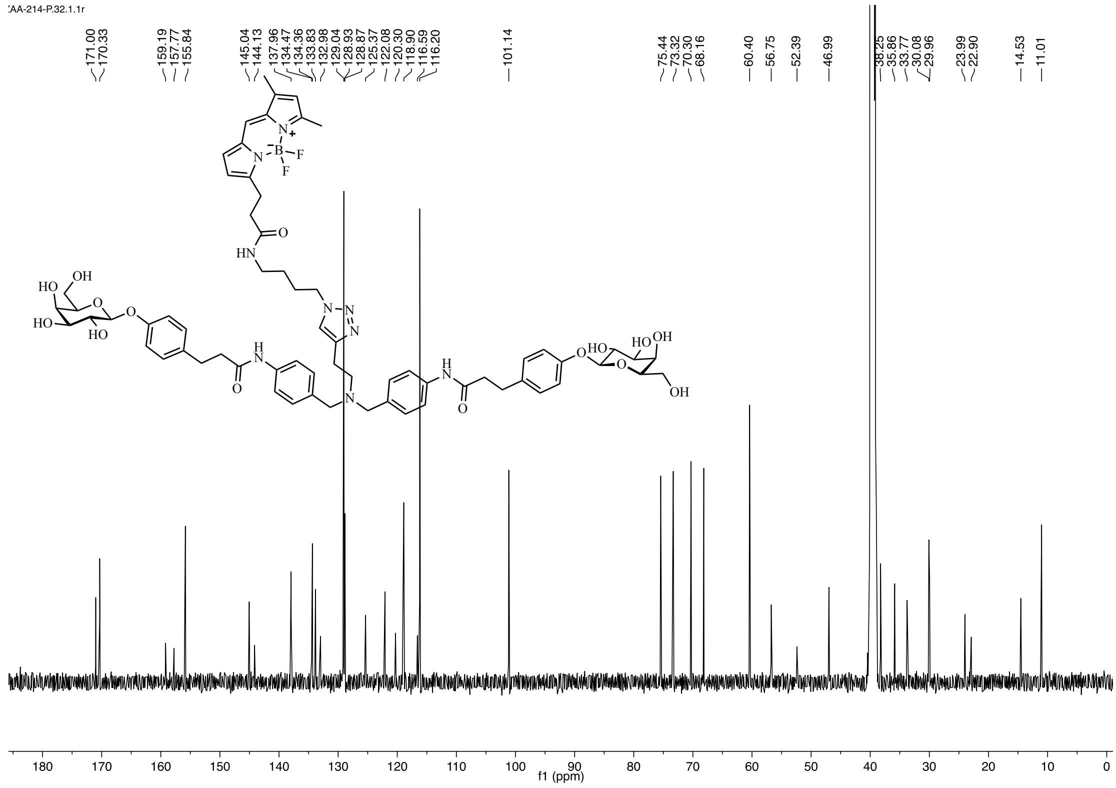

^{1}H and ^{13}C NMR of **19**

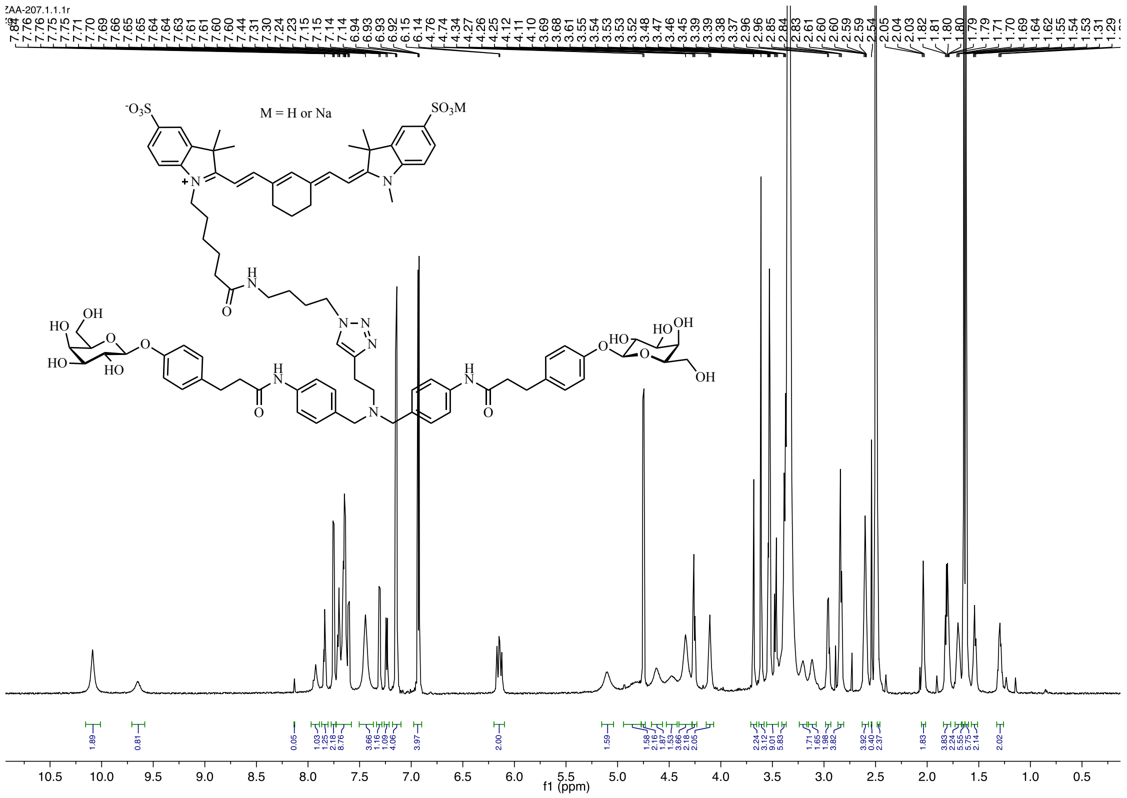

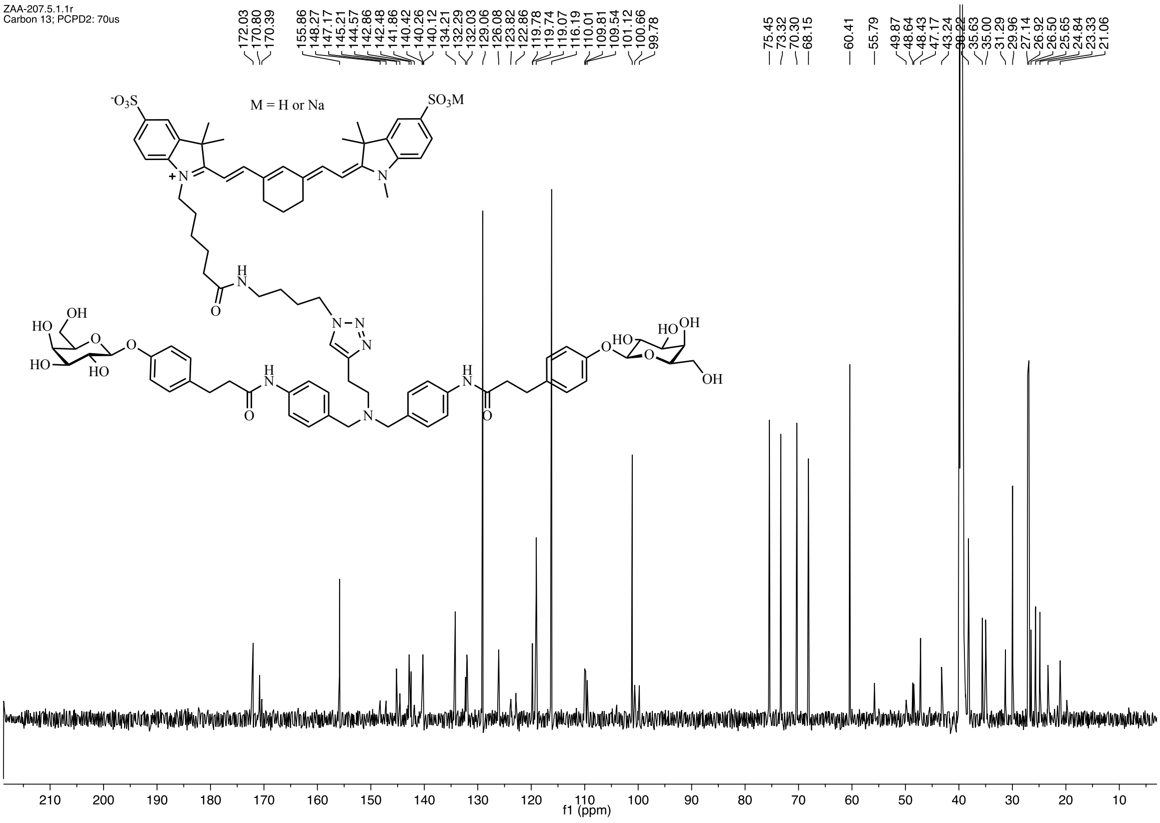

^{1}H and ^{13}C NMR of **21**

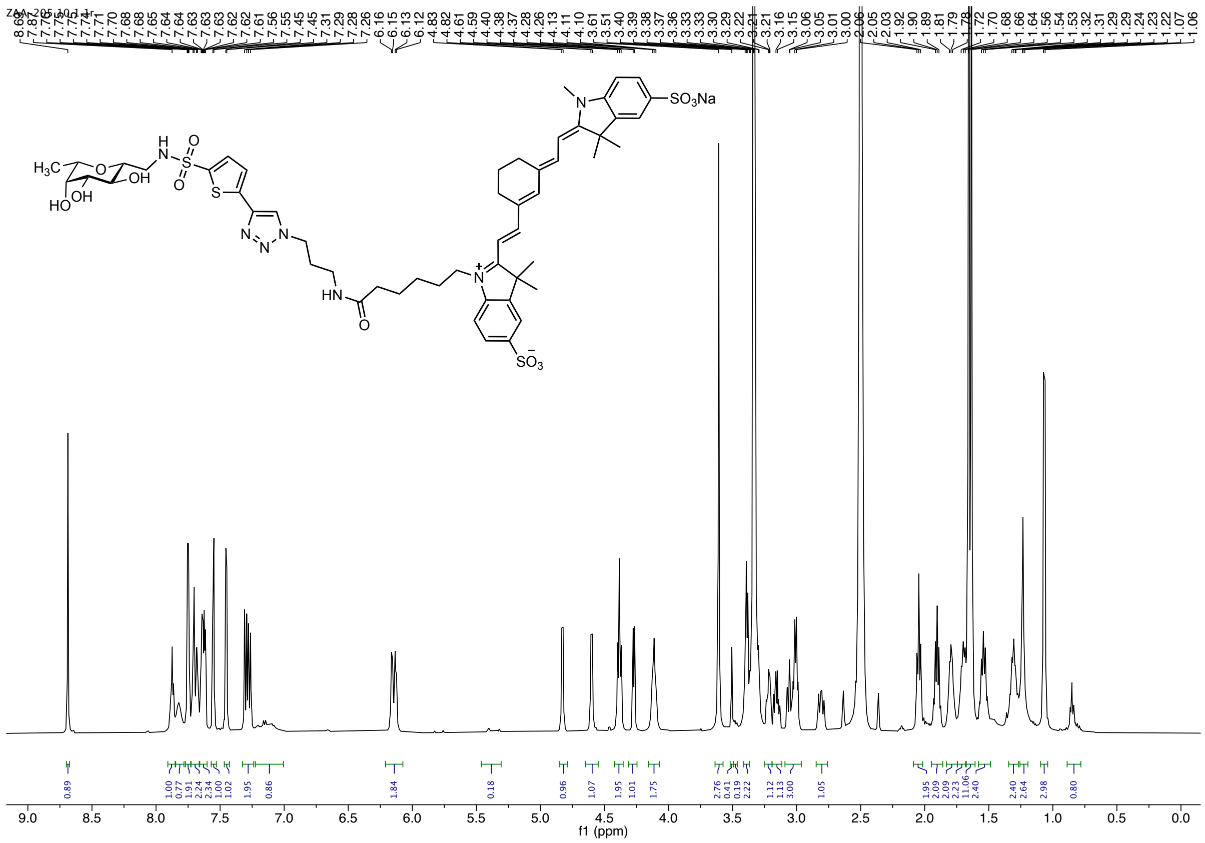

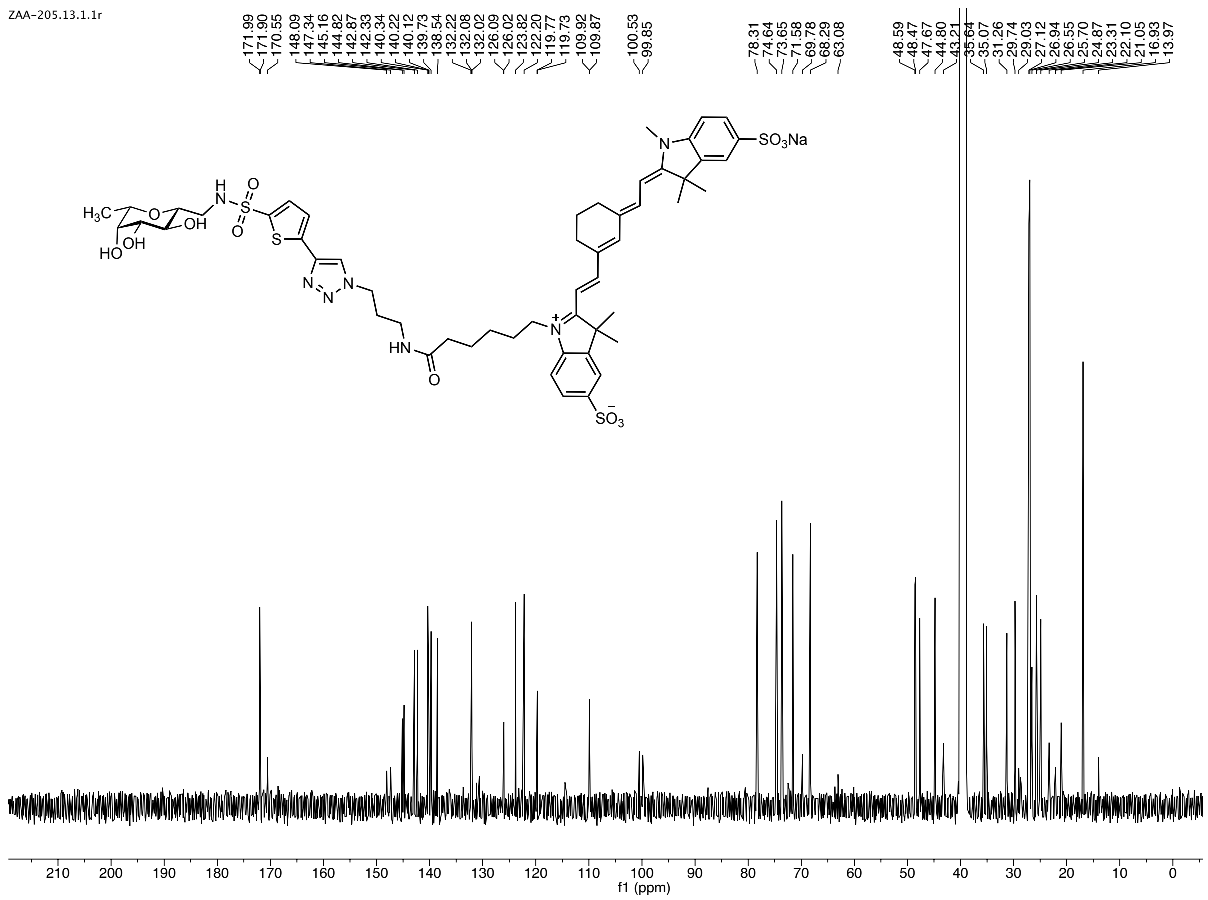

^{1}H and ^{13}C NMR of **22**

<u>**REFERENCES**</u>

(1) Hauck, D.; Jumde, V. R.; Crawford, C. J.; Titz, A. Optimized Henry Reaction Conditions for the Synthesis of an L -Fucose C -Glycosyl Derivative. In *Carbohydrate Chemistry*; Kosma, P., Wrodnigg, T. M., Stütz, A., Eds.; CRC Press, 2021; pp 17–21.

(2) Zahorska, E.; Kuhaudomlarp, S.; Minervini, S.; Yousaf, S.; Lepsik, M.; Kinsinger, T.; Hirsch, A. K. H.; Imberty, A.; Titz, A. A Rapid Synthesis of Low-Nanomolar Divalent LecA Inhibitors in Four Linear Steps from D -Galactose. *Chem. Commun.* **2020**, *56*, 8822–8825. https://doi.org/10.1039/d0cc03490h.

(3) Gottlieb, H. E.; Kotlyar, V.; Nudelman, A. NMR Chemical Shifts of Common Laboratory Solvents as Trace Impurities. *J. Org. Chem.* **1997**, *62* (21), 7512–7515. https://doi.org/10.1021/jo971176v.

(4) Ramos-Soriano, J.; Niss, U.; Angulo, J.; Angulo, M.; Moreno-Vargas, A. J.; Carmona, A. T.; Ohlson, S.; Robina, I. Synthesis, Biological Evaluation, Wac and NMR Studies of S-Galactosides and Non-Carbohydrate Ligands of Cholera Toxin Based on Polyhydroxyalkylfuroate Moieties. *Chem. - A Eur. J.* **2013**, *19* (52), 17989–18003. https://doi.org/10.1002/chem.201302786.

(5) Cao, S.; Meunier, S. J.; Andersson, F. O.; Letellier, M.; Roy, R. Mild Stereoselective Syntheses of Thioglycosides under PTC Conditions and Their Use as Active and Latent Glycosyl Donors. *Tetrahedron: Asymmetry* **1994**, *5* (11), 2303–2312. https://doi.org/10.1016/S0957-4166(00)86308-9.

(6) Kumar, V.; Jamie Talisman, I.; Bukhari, O.; Razzaghy, J.; Malhotra, S. V. Dual Role of Ionic Liquids as Phase Transfer Catalyst and Solvent for Glycosidation Reactions. *RSC Adv.* **2011**, *1* (9), 1721–1727. https://doi.org/10.1039/c1ra00385b.

(7) Bartolozzi, A.; Foudoulakis, H. M.; Cole, B. M. Development of a Tandem Base-Catalyzed, Triphenylphosphine-Mediated Disulfide Reduction-Michael Addition. *Synthesis (Stuttg).* **2008**, No. 13, 2023–2032. https://doi.org/10.1055/s-2008-1067102.

(8) Nakahashi, A.; Fujita, M.; Miyoshi, E.; Umeyama, T.; Naka, K.; Chujo, Y. Synthesis of Sulfur-Containing Hyperbranched Polymers by the Bisthiolation Polymerization of Diethynyl Disulfide Derivatives. *J. Polym. Sci.* **2007**, *45* (16), 3580–3587. https://doi.org/10.1002/POLA.

(9) Inkster, J. A. H.; Liu, K.; Ait-Mohand, S.; Schaffer, P.; Guérin, B.; Ruth, T. J.; Storr, T. Sulfonyl Fluoride-Based Prosthetic Compounds as Potential 18F Labelling Agents. *Chem. - A Eur. J.* **2012**, *18* (35), 11079–11087. https://doi.org/10.1002/chem.201103450.

(10) Sommer, R.; Rox, K.; Wagner, S.; Hauck, D.; Henrikus, S. S.; Newsad, S.; Arnold, T.; Ryckmans, T.; Brönstrup, M.; Imberty, A.; Varrot, A.; Hartmann, R. W.; Titz, A. Anti-Biofilm Agents against Pseudomonas Aeruginosa: A Structure-Activity Relationship Study of C-Glycosidic LecB Inhibitors. *J. Med. Chem.* **2019**, *62* (20), 9201–9216. https://doi.org/10.1021/acs.jmedchem.9b01120.

(11) Cohen, R. B.; Tsou, K.-C.; Rutenburg, S. H.; Seligman, A. M. The Colorimetric Estimation and Histochemical Demonstration of Beta-D-Galactosidase. *J. Biol. Chem.* **1952**, *195*, 239–249. https://doi.org/10.1016/S0021-9258(19)50894-8.

(12) Hudson, C. S.; Johnson, J. M. The Isomeric Tetracetates of Xylose, and Observations Regarding the Acetates of Melibiose, Trehalose and Sucrose. *J. Am. Chem. Soc.* **1915**, *37* (12), 2748–2753. https://doi.org/10.1021/ja02177a023.

(13) Šardzík, R.; Noble, G. T.; Weissenborn, M. J.; Martin, A.; Webb, S. J.; Flitsch, S. L. Preparation of Aminoethyl Glycosides for Glycoconjugation. *Beilstein J. Org. Chem.* **2010**, *6*, 699–703. https://doi.org/10.3762/bjoc.6.81.

(14) Driguez, H.; Szeja, W. Facile Synthesis of 1,2-Trans-Nitrophenyl-1-Thioglycopyranosides. *Synthesis (Stuttg).* **1994**, *12*, 1413–1414. https://doi.org/10.1055/s-1994-25704.

(15) Loison, S.; Cottet, M.; Orcel, H.; Adihou, H.; Rahmeh, R.; Lamarque, L.; Trinquet, E.; Kellenberger, E.; Hibert, M.; Durroux, T.; Mouillac, B.; Bonnet, D. Selective Fluorescent Nonpeptidic Antagonists for Vasopressin V2 GPCR: Application to Ligand Screening and Oligomerization Assays. *J. Med. Chem.* **2012**, *55* (20), 8588–8602. https://doi.org/10.1021/jm3006146.

(16) Zahorska, E.; Rosato, F.; Stober, K.; Kuhaudomlarp, S.; Meiers, J.; Hauck, D.; Reith, D.; Gillon, E.; Rox, K.; Imberty, A.; Römer, W.; Titz, A. Neutralizing the Impact of the Virulence Factor LecA from Pseudomonas Aeruginosa on Human Cells with New Glycomimetic Inhibitors. *Angew. Chemie - Int. Ed.* **2022**, *62*, e202215535. https://doi.org/10.1002/anie.202215535.

(17) Blanchard, B.; Nurisso, A.; Hollville, E.; Tétaud, C.; Wiels, J.; Pokorná, M.; Wimmerová, M.; Varrot, A.; Imberty, A. Structural Basis of the Preferential Binding for Globo-Series Glycosphingolipids Displayed by Pseudomonas Aeruginosa Lectin I. *J. Mol. Biol.* **2008**, *383* (4), 837–853. https://doi.org/10.1016/j.jmb.2008.08.028.

(18) Joachim, I.; Rikker, S.; Hauck, D.; Ponader, D.; Boden, S.; Sommer, R.; Hartmann, L.; Titz, A. Development and Optimization of a Competitive Binding Assay for the Galactophilic Low Affinity Lectin LecA from Pseudomonas Aeruginosa. *Org. Biomol. Chem.* **2016**, *14*, 7933–7948. https://doi.org/10.1039/c6ob01313a.

(19) Hauck, D.; Joachim, I.; Frommeyer, B.; Varrot, A.; Philipp, B.; Möller, H. M.; Imberty, A.; Exner, T. E.; Titz, A. Discovery of Two Classes of Potent Glycomimetic Inhibitors of Pseudomonas Aeruginosa LecB with Distinct Binding Modes. *ACS Chem. Biol.* **2013**, *8* (8), 1775–1784. https://doi.org/10.1021/cb400371r.

(20) Mitchell, E. P.; Sabin, C.; Šnajdrová, L.; Pokorná, M.; Perret, S.; Gautier, C.; Hofr, C.; Gilboa-Garber, N.; Koča, J.; Wimmerová, M.; Imberty, A. High Affinity Fucose Binding of Pseudomonas Aeruginosa Lectin PA-IIL: 1.0 Å Resolution Crystal Structure of the Complex Combined with Thermodynamics and Computational Chemistry Approaches. *Proteins Struct. Funct. Genet.* **2005**, *58* (3), 735–746. https://doi.org/10.1002/prot.20330.

(21) Beshr, G.; Sikandar, A.; Jemiller, E. M.; Klymiuk, N.; Hauck, D.; Wagner, S.; Wolf, E.; Koehnke, J.; Titz, A. Photorhabdus Luminescens Lectin A (PllA): A New Probe for Detecting -Galactoside–terminating Glycoconjugates. *J. Biol. Chem.* **2017**, *292* (48), 19935–19951. https://doi.org/10.1074/jbc.M117.812792.

(22) Turnbull, W. B.; Daranas, A. H. On the Value of c: Can Low Affinity Systems Be Studied by Isothermal Titration Calorimetry? *J. Am. Chem. Soc.* **2003**, *125* (48), 14859–14866. https://doi.org/10.1021/ja036166s.

(23) Lagendijk, E. L.; Validov, S.; Lamers, G. E. M.; De Weert, S.; Bloemberg, G. V. Genetic Tools for Tagging Gram-Negative Bacteria with mCherry for Visualization in Vitro and in Natural Habitats, Biofilm and Pathogenicity Studies. *FEMS Microbiol. Lett.* **2010**, *305* (1), 81–90. https://doi.org/10.1111/j.1574-6968.2010.01916.x.

(24) Wagner, S.; Hauck, D.; Hoffmann, M.; Sommer, R.; Joachim, I.; Müller, R.; Imberty, A.; Varrot, A.; Titz, A. Covalent Lectin Inhibition and Application in Bacterial Biofilm Imaging. *Angew. Chemie - Int. Ed.* **2017**, *56* (52), 16559–16564. https://doi.org/10.1002/anie.201709368.

(25) Ghanbari, A.; Dehghany, J.; Schwebs, T.; Müsken, M.; Häussler, S.; Meyer-Hermann, M. Inoculation Density and Nutrient Level Determine the Formation of Mushroom-Shaped Structures in Pseudomonas Aeruginosa Biofilms. *Sci. Rep.* **2016**, *6* (February), 32097. https://doi.org/10.1038/srep32097.

(26) Munder, A.; Tümmler, B. Assessing Pseudomonas Virulence Using Mammalian Models: Acute Infection Model. In *Pseudomonas Methods and Protocols*; Filloux, A., Ramos, J.-L., Eds.; Springer New York: New York, NY, 2014; pp 773–791. https://doi.org/10.1007/978-1-4939-0473-0_59.

(27) Schindelin, J.; Arganda-Carreras, I.; Frise, E.; Kaynig, V.; Longair, M.; Pietzsch, T.; Preibisch, S.; Rueden, C.; Saalfeld, S.; Schmid, B.; Tinevez, J. Y.; White, D. J.; Hartenstein, V.; Eliceiri, K.; Tomancak, P.; Cardona, A. Fiji: An Open-Source Platform for Biological-Image Analysis. *Nat. Methods* **2012**, *9* (7), 676–682. https://doi.org/10.1038/nmeth.2019.
